# Supplementary material for: Barriers and facilitators perceived by healthcare professionals for implementing lifestyle interventions in patients with osteoarthritis: a scoping review
Source: BMJ Open. 2022 Feb 1;12(2):e056831. doi: 10.1136/bmjopen-2021-056831 (PMC8808449; doi:10.1136/bmjopen-2021-056831)
Supplement: Supplementary data [file bmjopen-2021-056831supp001.pdf]

Supplemental File 1. Search strategies for the different databases

The search strategies presented below were applied in five bibliographic electronic databases on two different dates: initially on 19 February 2019 and again on 19 January 2021 to update the results. For each database it is shown below which search strategy was used in 2019 (#1) and in 2021 (#2) and how many records were retrieved using these search strategies.

PubMed

#1<br>(1798 hits)	((((("Health Personnel"[Mesh] OR "Delivery of Health Care"[Mesh] OR "Primary Health Care"[Mesh]) OR (Health personnel*[tiab] OR Health professional*[tiab] OR Medical staff*[tiab] OR Clinician*[tiab] OR Medical specialist*[tiab] OR Health care*[tiab] OR Healthcare*[tiab] OR Primary care*[tiab] OR Secondary care*[tiab] OR Allied health personnel*[tiab] OR Allied health professional*[tiab] OR Paramedic*[tiab] OR Nurse*[tiab] OR Nursing staff*[tiab] OR Nurse practit*[tiab] OR Physician assistant*[tiab] OR Physicians assistant*[tiab] OR Nutritionist*[tiab] OR Dietician*[tiab] OR Dietitian*[tiab] OR Physical therapist*[tiab] OR Physiotherapist*[tiab] OR Lifestyle coach*[tiab] OR Lifestyle counselor*[tiab] OR Physician*[tiab] OR Orthopedic surgeon*[tiab] OR Orthopedist*[tiab] OR General practitioner*[tiab] OR General practice*[tiab] OR Psychologist*[tiab])))<br>**AND**<br>((("Osteoarthritis"[Mesh]) OR (Osteoarthrit*[tiab] OR Osteoarthro*[tiab] OR Degenerative arthrit*[tiab]))) AND ((("Hip Joint"[Mesh] OR "Knee Joint"[Mesh] OR "Lower Extremity"[Mesh]) OR (Hip*[tiab] OR Cox*[tiab] OR Acetabulofemoral joint*[tiab] OR Knee*[tiab] OR Superior tibulofibular joint*[tiab] OR Patellofemoral*[tiab] OR Lower extremit*[tiab] OR Lower limb*[tiab]))))<br>**AND**<br>((((("Life Style"[Mesh] OR "Behavior"[Mesh]) OR (Life style*[tiab] OR Lifestyle*[tiab] OR Behavior*[tiab] OR Behaviour*[tiab] OR Habit*[tiab] OR Risk reduction*[tiab] OR Early therap*[tiab] OR Secondary prevention*[tiab] OR Tertiary prevention*[tiab] OR Disease prevention*[tiab]))) OR ((("Exercise"[Mesh] OR "Exercise Therapy"[Mesh] OR "Sports"[Mesh]) OR (Physical activit*[tiab] OR Physical training*[tiab] OR Physical fitness*[tiab] OR Physical condition*[tiab] OR Physical therap*[tiab] OR Physiotherap*[tiab] OR Exercis*[tiab] OR Run*[tiab] OR Jog*[tiab] OR Walk*[tiab] OR Bicycl*[tiab] OR Swim*[tiab] OR Strength*[tiab] OR Resistance*[tiab] OR Sport*[tiab] OR Athletic*[tiab] OR Train*[tiab] OR Sedentary[tiab]))) OR ((("Diet, Food, and Nutrition"[Mesh] OR "Nutrition Therapy"[Mesh] OR "Body Weight"[Mesh]) OR (Healthy diet*[tiab] OR Healthy eating*[tiab] OR Nutrition*[tiab] OR Diet*[tiab] OR Food*[tiab] OR Weight loss*[tiab] OR Weight loss program*[tiab] OR Weight reduction*[tiab] OR Weight reduction program*[tiab] OR Body weight*[tiab] OR Overweight*[tiab] OR Obesity*[tiab] OR Overnutrition*[tiab] OR Hypernutrition*[tiab])))))<br>**AND**<br>((("Attitude"[Mesh] OR "Motivation"[Mesh]) OR (Barrier*[tiab] OR Facilitator*[tiab] OR Enabler*[tiab] OR Driver*[tiab] OR Motivat*[tiab] OR Opinion*[tiab] OR View*[tiab] OR Attitude*[tiab] OR Expectation*[tiab] OR Incentive*[tiab] OR Disincentive*[tiab] OR Belief*[tiab] OR Influencing factor*[tiab] OR Experience*[tiab] OR Perspective*[tiab] OR Perception*[tiab] OR Hinder*[tiab] OR Impediment*[tiab] OR obstacle*[tiab])))))
#2<br>(467 hits)	#1 **AND** (("2019/01/01"[Date - Create] : "3000"[Date - Create]) OR ("2019/01/01"[Date - Entry] : "3000"[Date - Entry]) OR ("2019/01/01"[Date - MeSH] : "3000"[Date - MeSH]))

Embase

#1	(‘health care personnel’/exp OR ‘health care delivery’/exp OR (‘Health personnel*’ OR ‘Health professional*’ OR ‘Medical staff*’ OR ‘Clinician*’ OR

(3036 hits)	‘Medical specialist*’ OR ‘Health care*’ OR ‘Healthcare*’ OR ‘Primary care*’ OR ‘Secondary care*’ OR ‘Allied health personnel*’ OR ‘Allied health professional*’ OR ‘Paramedic*’ OR ‘Nurse*’ OR ‘Nursing staff*’ OR ‘Nurse practit*’ OR ‘Physician assistant*’ OR ‘Physicians assistant*’ OR ‘Nutritionist*’ OR ‘Dietician*’ OR ‘Dietitian*’ OR ‘Physical therapist*’ OR ‘Physiotherapist*’ OR ‘Lifestyle coach*’ OR ‘Lifestyle counselor*’ OR ‘Physician*’ OR ‘Orthopedic surgeon*’ OR ‘Orthopedist*’ OR ‘General practitioner*’ OR ‘General practice*’ OR ‘Psychologist*’):ab,ti)<br>**AND**<br>(‘osteoarthritis’/exp OR (‘Osteoarthrit*’ OR ‘Osteoarthro*’ OR ‘Degenerative arthrit*’):ab,ti) AND (‘lower limb’/exp OR (‘Hip*’ OR ‘Cox*’ OR ‘Acetabulofemoral joint*’ OR ‘Knee*’ OR ‘Superior tibulofibular joint*’ OR ‘Patellofemoral*’ OR ‘Lower extremit*’ OR ‘Lower limb*’):ab,ti)<br>**AND**<br>((‘lifestyle’/exp OR ‘lifestyle modification’/exp OR ‘behavior’/exp OR (‘Life style*’ OR ‘Lifestyle*’ OR ‘Behavior*’ OR ‘Behaviour*’ OR ‘Habit*’ OR ‘Risk reduction*’ OR ‘Early therap*’ OR ‘Secondary prevention*’ OR ‘Tertiary prevention*’ OR ‘Disease prevention*’):ab,ti) OR (‘exercise’/exp OR ‘kinesiotherapy’/exp OR ‘sport’/exp OR (‘Physical activit*’ OR ‘Physical training*’ OR ‘Physical fitness*’ OR ‘Physical condition*’ OR ‘Physical therap*’ OR ‘Physiotherap*’ OR ‘Exercis*’ OR ‘Run*’ OR ‘Jog*’ OR ‘Walk*’ OR ‘Bicycl*’ OR ‘Swim*’ OR ‘Strength*’ OR ‘Resistance*’ OR ‘Sport*’ OR ‘Athletic*’ OR ‘Train*’ OR ‘Sedentary’):ab,ti) OR (‘nutrition’/exp OR ‘diet therapy’/exp OR ‘body weight’/exp OR ‘body weight management’/exp OR (‘Healthy diet*’ OR ‘Healthy eating*’ OR ‘Nutrition*’ OR ‘Diet*’ OR ‘Food*’ OR ‘Weight loss*’ OR ‘Weight loss program*’ OR ‘Weight reduction*’ OR ‘Weight reduction program*’ OR ‘Body weight*’ OR ‘Overweight*’ OR ‘Obesity*’ OR ‘Overnutrition*’ OR ‘Hypernutrition*’):ab,ti))<br>**AND**<br>(‘attitude’/exp OR ‘motivation’/exp OR (‘Barrier*’ OR ‘Facilitator*’ OR ‘Enabler*’ OR ‘Driver*’ OR ‘Motivat*’ OR ‘Opinion*’ OR ‘View*’ OR ‘Attitude*’ OR ‘Expectation*’ OR ‘Incentive*’ OR ‘Disincentive*’ OR ‘Belief*’ OR ‘Influencing factor*’ OR ‘Experience*’ OR ‘Perspective*’ OR ‘Perception*’ OR ‘Hinder*’ OR ‘Impediment*’ OR ‘obstacle*’):ab,ti)
#2<br>(1021 hits)	#1 **AND** [1-1-2019]/sd NOT [20-1-2021]/sd

CINAHL

#1<br>(424 hits)	(MH “Health Personnel+” OR MH “Health Care Delivery+” OR TI(Health personnel* OR Health professional* OR Medical staff* OR Clinician* OR Medical specialist* OR Health care* OR Healthcare* OR Primary care* OR Secondary care* OR Allied health personnel* OR Allied health professional* OR Paramedic* OR Nurse* OR Nursing staff* OR Nurse practit* OR Physician assistant* OR Physicians assistant* OR Nutritionist* OR Dietician* OR Dietitian* OR Physical therapist* OR Physiotherapist* OR Lifestyle coach* OR Lifestyle counselor* OR Physician* OR Orthopedic surgeon* OR Orthopedist* OR General practitioner* OR General practice* OR Psychologist*) OR AB(Health personnel* OR Health professional* OR Medical staff* OR Clinician* OR Medical specialist* OR Health care* OR Healthcare* OR Primary care* OR Secondary care* OR Allied health personnel* OR Allied health professional* OR Paramedic* OR Nurse* OR Nursing staff* OR Nurse practit* OR Physician assistant* OR Physicians assistant* OR Nutritionist* OR Dietician* OR Dietitian* OR Physical therapist* OR Physiotherapist* OR Lifestyle coach* OR Lifestyle counselor* OR Physician* OR Orthopedic surgeon* OR Orthopedist* OR General practitioner* OR General practice* OR Psychologist*))<br>**AND**<br>(MH “Osteoarthritis+” OR TI(Osteoarthrit* OR Osteoarthro* OR Degenerative arthrit*) OR AB(Osteoarthrit* OR Osteoarthro* OR Degenerative arthrit*)) AND (MH “Lower Extremity+” OR MH “Hip Joint+” OR MH “Knee Joint+” OR TI(Hip* OR Cox* OR Acetabulofemoral joint* OR Knee* OR Superior tibulofibular joint* OR Patellofemoral* OR Lower extremit* OR Lower limb*) OR AB(Hip* OR Cox* OR Acetabulofemoral joint* OR Knee* OR Superior tibulofibular joint* OR Patellofemoral* OR Lower extremit* OR Lower limb*))

	**AND**<br>((MH "Life Style+" OR MH "Behavior+" OR TI(Life style* OR Lifestyle* OR Behavior* OR Behaviour* OR Habit* OR Risk reduction* OR Early therap* OR Secondary prevention* OR Tertiary prevention* OR Disease prevention*) OR AB(Life style* OR Lifestyle* OR Behavior* OR Behaviour* OR Habit* OR Risk reduction* OR Early therap* OR Secondary prevention* OR Tertiary prevention* OR Disease prevention*)) OR (MH "Exercise+" OR MH "Therapeutic Exercise+" OR MH "Sports+" OR TI(Physical activit* OR Physical training* OR Physical fitness* OR Physical condition* OR Physical therap* OR Physiotherap* OR Exercis* OR Run* OR Jog* OR Walk* OR Bicycl* OR Swim* OR Strength* OR Resistance* OR Sport* OR Athletic* OR Train* OR Sedentary) OR AB(Physical activit* OR Physical training* OR Physical fitness* OR Physical condition* OR Physical therap* OR Physiotherap* OR Exercis* OR Run* OR Jog* OR Walk* OR Bicycl* OR Swim* OR Strength* OR Resistance* OR Sport* OR Athletic* OR Train* OR Sedentary)) OR (MH "Nutrition+" OR MH "Food+" OR MH "Diet Therapy+" OR MH "Body Weight+" OR TI(Healthy diet* OR Healthy eating* OR Nutrition* OR Diet* OR Food* OR Weight loss* OR Weight loss program* OR Weight reduction* OR Weight reduction program* OR Body weight* OR Overweight* OR Obesity* OR Overnutrition* OR Hypernutrition*) OR AB(Healthy diet* OR Healthy eating* OR Nutrition* OR Diet* OR Food* OR Weight loss* OR Weight loss program* OR Weight reduction* OR Weight reduction program* OR Body weight* OR Overweight* OR Obesity* OR Overnutrition* OR Hypernutrition*)))<br>**AND**<br>(MH "Attitude+" OR MH "Motivation+" OR TI(Barrier* OR Facilitator* OR Enabler* OR Driver* OR Motivat* OR Opinion* OR View* OR Attitude* OR Expectation* OR Incentive* OR Disincentive* OR Belief* OR Influencing factor* OR Experience* OR Perspective* OR Perception* OR Hinder* OR Impediment* OR Obstacle*) OR AB(Barrier* OR Facilitator* OR Enabler* OR Driver* OR Motivat* OR Opinion* OR View* OR Attitude* OR Expectation* OR Incentive* OR Disincentive* OR Belief* OR Influencing factor* OR Experience* OR Perspective* OR Perception* OR Hinder* OR Impediment* OR Obstacle*))
#2<br>(598 hits)	#1

PsycINFO

#1<br>(49 hits)	(DE "Health Personnel" OR DE "Health Care Delivery" OR DE "Primary Health Care" OR DE "Physical Therapists" OR DE "Nurses" OR DE "Physicians" OR DE "Family Physicians" OR DE "General Practitioners" OR DE "Surgeons" OR DE "Clinical Psychologists" OR DE "Allied Health Personnel" OR DE "Caregivers" OR DE "Medical Personnel" OR DE "Mental Health Personnel" OR DE "Clinicians" OR DE "Therapists" OR DE "Psychologists" OR TI(Health personnel* OR Health professional* OR Medical staff* OR Clinician* OR Medical specialist* OR Health care* OR Healthcare* OR Primary care* OR Secondary care* OR Allied health personnel* OR Allied health professional* OR Paramedic* OR Nurse* OR Nursing staff* OR Nurse practit* OR Physician assistant* OR Physicians assistant* OR Nutritionist* OR Dietician* OR Dietitian* OR Physical therapist* OR Physiotherapist* OR Lifestyle coach* OR Lifestyle counselor* OR Physician* OR Orthopedic surgeon* OR Orthopedist* OR General practitioner* OR General practice* OR Psychologist*) OR AB(Health personnel* OR Health professional* OR Medical staff* OR Clinician* OR Medical specialist* OR Health care* OR Healthcare* OR Primary care* OR Secondary care* OR Allied health personnel* OR Allied health professional* OR Paramedic* OR Nurse* OR Nursing staff* OR Nurse practit* OR Physician assistant* OR Physicians assistant* OR Nutritionist* OR Dietician* OR Dietitian* OR Physical therapist* OR Physiotherapist* OR Lifestyle coach* OR Lifestyle counselor* OR Physician* OR Orthopedic surgeon* OR Orthopedist* OR General practitioner* OR General practice* OR Psychologist*))<br>**AND**<br>(TI(Osteoarthrit* OR Osteoarthro* OR Degenerative arthrit*) OR AB(Osteoarthrit* OR Osteoarthro* OR Degenerative arthrit*)) AND (DE "Hips" OR DE "Knee" OR TI(Hip* OR Cox* OR Acetabulofemoral joint* OR Knee* OR Superior tibulofibular joint* OR Patellofemoral* OR Lower extremit* OR Lower limb*) OR AB(Hip* OR Cox* OR Acetabulofemoral joint* OR Knee* OR Superior tibulofibular joint* OR Patellofemoral* OR Lower extremit* OR Lower

	limb*))<br>**AND**<br>((DE "Lifestyle" OR DE "Behavior" OR DE "Active Living" OR DE "Lifestyle Changes" OR DE "Activity Level" OR DE "Habits" OR DE "Behavior Change" OR DE "Readiness to Change" OR DE "Stages of Change" OR DE "Illness Behavior" OR DE "Health Behavior" OR DE "Health Attitudes" OR TI(Life style* OR Lifestyle* OR Behavior* OR Behaviour* OR Habit* OR Risk reduction* OR Early therap* OR Secondary prevention* OR Tertiary prevention* OR Disease prevention*) OR AB(Life style* OR Lifestyle* OR Behavior* OR Behaviour* OR Habit* OR Risk reduction* OR Early therap* OR Secondary prevention* OR Tertiary prevention* OR Disease prevention*)) OR (DE "Exercise" OR DE "Movement Therapy" OR DE "Sports" OR DE "Aerobic Exercise" OR DE "Physical Activity" OR DE "Physical Fitness" OR DE "Rehabilitation" OR DE "Physical Therapy" OR DE "Sedentary Behavior" OR TI(Physical activit* OR Physical training* OR Physical fitness* OR Physical condition* OR Physical therap* OR Physiotherap* OR Exercis* OR Run* OR Jog* OR Walk* OR Bicycl* OR Swim* OR Strength* OR Resistance* OR Sport* OR Athletic* OR Train* OR Sedentary) OR AB(Physical activit* OR Physical training* OR Physical fitness* OR Physical condition* OR Physical therap* OR Physiotherap* OR Exercis* OR Run* OR Jog* OR Walk* OR Bicycl* OR Swim* OR Strength* OR Resistance* OR Sport* OR Athletic* OR Train* OR Sedentary)) OR (DE "Nutrition" OR DE "Food" OR DE "Diets" OR DE "Body Weight" OR DE "Weight Control" OR DE "Calories" OR DE "Body Mass Index" OR DE "Overweight" OR DE "Weight Loss" OR DE "Obesity" OR TI(Healthy diet* OR Healthy eating* OR Nutrition* OR Diet* OR Food* OR Weight loss* OR Weight loss program* OR Weight reduction* OR Weight reduction program* OR Body weight* OR Overweight* OR Obesity* OR Overnutrition* OR Hypernutrition*) OR AB(Healthy diet* OR Healthy eating* OR Nutrition* OR Diet* OR Food* OR Weight loss* OR Weight loss program* OR Weight reduction* OR Weight reduction program* OR Body weight* OR Overweight* OR Obesity* OR Overnutrition* OR Hypernutrition*)))<br>**AND**<br>(DE "Attitudes" OR DE "Motivation" OR DE "Health Personnel Attitudes" OR DE "Therapist Attitudes" OR DE "Psychologist Attitudes" OR DE "Extrinsic Motivation" OR DE "Intrinsic Motivation" OR TI(Barrier* OR Facilitator* OR Enabler* OR Driver* OR Motivat* OR Opinion* OR View* OR Attitude* OR Expectation* OR Incentive* OR Disincentive* OR Belief* OR Influencing factor* OR Experience* OR Perspective* OR Perception* OR Hinder* OR Impediment* OR Obstacle*) OR AB(Barrier* OR Facilitator* OR Enabler* OR Driver* OR Motivat* OR Opinion* OR View* OR Attitude* OR Expectation* OR Incentive* OR Disincentive* OR Belief* OR Influencing factor* OR Experience* OR Perspective* OR Perception* OR Hinder* OR Impediment* OR Obstacle*))
#2<br>(14 hits)	#1 **AND** RD 20190101-

The Cochrane Library

#1<br>(299 hits)	((Health personnel* OR Health professional* OR Medical staff* OR Clinician* OR Medical specialist* OR Health care* OR Healthcare* OR Primary care* OR Secondary care* OR Allied health personnel* OR Allied health professional* OR Paramedic* OR Nurse* OR Nursing staff* OR Nurse practit* OR Physician assistant* OR Physicians assistant* OR Nutritionist* OR Dietician* OR Dietitian* OR Physical therapist* OR Physiotherapist* OR Lifestyle coach* OR Lifestyle counselor* OR Physician* OR Orthopedic surgeon* OR Orthopedist* OR General practitioner* OR General practice* OR Psychologist*)<br>**AND**<br>(Osteoarthrit* OR Osteoarthro* OR Degenerative arthrit*) AND (Hip* OR Cox* OR Acetabulofemoral joint* OR Knee* OR Superior tibulofibular joint* OR Patellofemoral* OR Lower extremit* OR Lower limb*)<br>**AND**<br>((Life style* OR Lifestyle* OR Behavior* OR Behaviour* OR Habit* OR Risk reduction* OR Early therap* OR Secondary prevention* OR Tertiary

	prevention* OR Disease prevention*) OR (Physical activit* OR Physical training* OR Physical fitness* OR Physical condition* OR Physical therap* OR Physiotherap* OR Exercis* OR Run* OR Jog* OR Walk* OR Bicycl* OR Swim* OR Strength* OR Resistance* OR Sport* OR Athletic* OR Train* OR Sedentary) OR (Healthy diet* OR Healthy eating* OR Nutrition* OR Diet* OR Food* OR Weight loss* OR Weight loss program* OR Weight reduction* OR Weight reduction program* OR Body weight* OR Overweight* OR Obesity* OR Overnutrition* OR Hypernutrition*))<br>**AND**<br>(Barrier* OR Facilitator* OR Enabler* OR Driver* OR Motivat* OR Opinion* OR View* OR Attitude* OR Expectation* OR Incentive* OR Disincentive* OR Belief* OR Influencing factor* OR Experience* OR Perspective* OR Perception* OR Hinder* OR Impediment* OR Obstacle*)):ti,ab,kw
#2<br>(632 hits)	#1
