# Supplementary material for: Barriers and facilitators perceived by healthcare professionals for implementing lifestyle interventions in patients with osteoarthritis: a scoping review
Source: BMJ Open. 2022 Feb 1;12(2):e056831. doi: 10.1136/bmjopen-2021-056831 (PMC8808449; doi:10.1136/bmjopen-2021-056831)
Supplement: Supplementary data [file bmjopen-2021-056831supp002.pdf]

Supplemental File 2. Quality assessment of the included studies based on the Mixed Methods Appraisal Tool[a]

**Reference[b]**	**Screening questions[c]**		**1. Qualitative[d]**					**4. Quantitative descriptive[e]**					**5. Mixed-methods[f]**				
	**S1**	**S2**	**1.1**	**1.2**	**1.3**	**1.4**	**1.5**	**4.1**	**4.2**	**4.3**	**4.4**	**4.5**	**5.1**	**5.2**	**5.3**	**5.4**	**5.5**
Allison (2019) [27]	+	+	+	+	?	+	+										
Bossen (2016) [28][g]	+	+	?	+	?	-	?										
Christiansen (2020) [29]	+	+	?	+	?	+	+										
Davis (2018) [30]	+	+	?	+	?	-	?										
De Rooij (2014) [31]	+	+	?	+	?	-	?										
Egerton (2017) [32]	+	+	?	+	?	+	+										
Egerton (2018) [33]	+	+	?	+	?	+	+										
Hinman (2016) [34]	+	+	+	+	+	+	+										
Hinman (2017) [35]	+	+	?	+	?	+	+										
Knoop (2020) [36]	+	+	?	+	?	-	?										
Law (2019) [37]	+	+	?	+	?	+	+										
Lawford (2019) [38]	+	+	?	+	?	+	+										
Lawford (2020) [39]	+	+	?	+	?	+	+										
Lawford (2021) [40]	+	+	?	+	?	+	+										
MacKay (2018) [41]	+	+	?	+	?	+	+										
MacKay (2020) [42]	+	+	?	+	?	+	+										
Mann (2011) [43]	+	+	?	+	?	+	+										
Miller (2020) [44]	+	+	?	+	?	-	+										
Nielsen (2014) [45]	+	+	?	+	?	+	+										
Okwera (2019) [46]	+	+	?	+	?	+	+										
Poitras (2010) [47]	+	+	?	+	?	-	+										
Rosemann (2006) [48]	+	+	?	+	?	-	?										
Selten (2017) [49]	+	+	?	+	?	+	+										
Tang (2020) [50]	+	+	?	+	?	+	+										
Teo (2020) [51]	+	+	?	+	?	+	+										
Wallis (2020) [52]	+	+	?	+	?	+	+										
Cottrell (2016) [53]	+	+						+	+	+	-	+					
Duarte (2019) [54]	+	+						+	?	+	+	?					
Hill (2018) [55]	+	+						+	?	+	-	+					
Hill (2018) [56]	+	+						-	?	+	?	+					
Hofstede (2016) [57]	+	+						+	?	+	-	+					
Lawford (2018) [58]	+	+						+	?	+	?	+					

Reid (2014) [59]	+	+						+	?	+	-	+					
De Rooij (2020) [60]	+	+	?	+	?	-	?	+	?	+	+	+	?	+	?	+	-
Holden (2009) [61]	+	+	?	+	?	+	+	+	?	+	-	+	+	+	+	+	-
Kloek (2020) [62]	+	+	?	+	?	+	+	+	?	+	-	+	+	+	?	-	-

[a] + = yes; - = no, ? = can't tell
[b] The numbers in brackets ('[…]') correspond to the reference numbers used in the main text of the manuscript.
[c] S1 = Are there clear research questions?; S2 = Do the collected data allow to address the research questions?
[d] 1.1 = Is the qualitative approach appropriate to answer the research question?; 1.2 = Are the qualitative data collection methods adequate to address the research question?; 1.3 = Are the findings adequately derived from the data?; 1.4 = Is the interpretation of results sufficiently substantiated by data?; 1.5 = Is there coherence between qualitative data sources, collection, analysis and interpretation?
[e] 4.1 = Is the sampling strategy relevant to address the research question?; 4.2 = Is the sample representative of the target population?; 4.3 = Are the measurements appropriate?; 4.4 = Is the risk of nonresponse bias low?; 4.5 = Is the statistical analysis appropriate to answer the research question?
[f] 5.1 = Is there an adequate rationale for using a mixed methods design to address the research question?; 5.2 = Are the different components of the study effectively integrated to answer the research question?; 5.3 = Are the outputs of the integration of qualitative and quantitative components adequately interpreted?; 5.4 = Are divergences and inconsistencies between quantitative and qualitative results adequately addressed?; 5.5 = Do the different components of the study adhere to the quality criteria of each tradition of the methods involved?
[g] The MMAT was only applied to the section regarding the pilot study on the feasibility of the blended intervention.
