# Supplementary material for: Barriers and facilitators perceived by healthcare professionals for implementing lifestyle interventions in patients with osteoarthritis: a scoping review
Source: BMJ Open. 2022 Feb 1;12(2):e056831. doi: 10.1136/bmjopen-2021-056831 (PMC8808449; doi:10.1136/bmjopen-2021-056831)
Supplement: Supplementary data [file bmjopen-2021-056831supp003.pdf]

Supplemental File 3. Full overview of all extracted factors per domain

Explanation

- In the tables below, all extracted factors per subcategory of each domain are presented. The colors of the subcategories correspond to barriers (red), facilitators (green) or unclear factors (orange).
- Column "Description": (*) at the end of the description indicates that the factor is derived from a close-ended question or attitude statement.
- Column "Reference": the numbers in brackets ('[…]') correspond to the reference numbers used in the main text of the manuscript.
- Abbreviations: ACSM: American College of Sports Medicine; BMI: body mass index; CBT: cognitive behavioral therapy; CKP: chronic knee pain; CPG: clinical practice guideline; GP: general practitioner; HCP: healthcare professional; LI: lifestyle intervention; LMP: Lifestyle Management Programme; NSAID: non-steroidal anti-inflammatory drug; NWBE: non-weight bearing quadriceps strengthening exercise; OA: osteoarthritis; PCST: pain coping skills training; PT: physiotherapist; RCT: randomized controlled trial; TKA: total knee arthroplasty; TJA: total joint arthroplasty; WBE: weight bearing functional exercise.

Domain 1: Intervention factors

Effectiveness

Description	Reference
LIs have little or no effect on OA (barrier)	
Not certain that exercise works	Christiansen (2020) [29]
Advice to exercise and lose weight does not work	Egerton (2017) [32]
Dubious about effect of exercise and weight-management advice on reducing symptoms	Egerton (2018) [33]
Surgical methods have the best outcomes	Miller (2020) [44]
Lack of confidence in clinical effectiveness of physiotherapy treatments	Okwera (2019) [46]
Limited impact of weight loss on established knee OA (more effective as a primary prevention strategy)	Poitras (2010) [47]
Questioning direct relationship between weight and knee OA (numerous other factors associated)	Poitras (2010) [47]
Less certain about effectiveness of physical therapy (benefits variable or difficult to prove)	Selten (2017) [49]
Increasing the overall activity levels does not/might not stop the knee problem getting worse (*)	Cottrell (2016) [53]
There is a paucity of evidence in regards to the effectiveness of physiotherapy treatment for OA hip and/or knee (*)	Reid (2014) [59]
Past experience has shown physiotherapy to be ineffective (*)	Reid (2014) [59]
Increasing overall activity levels does not/might not stop the knee problem getting worse (*)	Holden (2009) [61]
Increasing the strength of the muscles around the knee does not/might not stop the knee problem getting worse (*)	Holden (2009) [61]
Knee problems are not/might not be improved by general exercise (*)	Holden (2009) [61]
Potential effects of LIs are difficult to accomplish (barrier)	
Weight loss is difficult (multiplicity of factors need to be addressed, often involving change in lifestyle)	Poitras (2010) [47]

Vicious circle (pain when exercising, people move less/eat more due to frustration/sometimes depression)	Rosemann (2006) [48]
Achieving patient behavior change is difficult (*)	Cottrell (2016) [53]
Human nature (as patient-centered barrier to adherence)	Holden (2009) [61]
LIs have positive effects on affected joint(s) (facilitator)	
Functional improvements experienced by patients	Hinman (2017) [35]
Improvements in patient pain and function	Lawford (2019) [38]
Large improvements in knee pain	Lawford (2021) [40]
Treatment could improve clients’ symptoms (e.g. reduce pain, increase function)	MacKay (2018) [41]
Treatment could potentially slow progression of symptoms	MacKay (2018) [41]
Improve people’s symptoms early in treatment (to gain buy-in)	MacKay (2020) [42]
Benefits of activity on knee mobility	Poitras (2010) [47]
Activity necessary for the knee’s health	Poitras (2010) [47]
Weight loss improves pain and joint function	Poitras (2010) [47]
Benefits of weight reduction for relieving symptoms of knee/hip OA	Selten (2017) [49]
Beneficial effects of physical therapy in reducing pain/stiffness and potential effects on cartilage	Selten (2017) [49]
Exercise therapy may be effective by giving more muscular support for joints	Wallis (2020) [52]
Knee problems are improved by quadriceps strengthening exercises (*)	Cottrell (2016) [53]
Knee problems are improved by general exercise (e.g. walking or swimming) (*)	Cottrell (2016) [53]
Increasing the strength of the muscles around the knee stops the knee problem getting worse (*)	Cottrell (2016) [53]
Exercise is beneficial for OA (*)	Lawford (2018) [58]
Knee problems are improved by local strengthening exercises (*)	Holden (2009) [61]
LIs have positive effects on general health (facilitator)	
Lifestyle treatments benefited other chronic conditions	Egerton (2018) [33]
Rapid weight loss was primary driver of motivation	Lawford (2021) [40]
Benefits of activity on general wellbeing	Poitras (2010) [47]
Weight loss effective at improving mobility in general	Poitras (2010) [47]
Weight loss also benefits mobility in general	Poitras (2010) [47]
Beneficial effects of physical therapy in reducing weight and for increasing mobility/posture/coordination	Selten (2017) [49]
Community interventions are effective at achieving sufficient and sustained weight loss (*)	Hill (2018) [56]
Patients benefit from weight loss (*)	Hofstede (2016) [57]
LIs have positive mental effects (facilitator)	
Enthusiastic about the program and described the results (e.g. it was empowering)	Davis (2018) [30]
Greater confidence to exercise among patients	Hinman (2017) [35]
Reminding patients of opportunity to self-manage	Law (2019) [37]
Increased confidence to self-manage	Lawford (2019) [38]
Positive lifestyle changes (patients) (e.g. thinking differently)	Lawford (2021) [40]
Physical therapy useful in increasing patients self-management in coping with/acceptance of symptoms	Selten (2017) [49]

Exercise therapy may be effective by giving opportunity to improve confidence about activities/mobility	Wallis (2020) [52]
Non-surgical treatments motivate patients to do things themselves (*)	Hofstede (2016) [57]
LIs have positive effects (not further specified) (facilitator)	
Positive impact on patients of personalized attention from coach and from advice/education they provided	Hinman (2016) [34]
Emphasising health benefits of programme	Law (2019) [37]
Physical therapy helpful for patients most of the time	Miller (2020) [44]
Value of lifestyle advice related to knee and hip OA	Selten (2017) [49]
Non-pharmacological, non-surgical treatment was considered useful to delay surgery	Selten (2017) [49]
Positive about program (alternative approach and opportunity to avoid a joint replacement)	Wallis (2020) [52]
Improvement in the physical condition of participants	Duarte (2019) [54]
Good results of physical therapy (*)	Hofstede (2016) [57]

Safety

Description	Reference
LIs are unsafe or have negative effects (barrier)	
Being apprehensive about aggravating pain in patients	Lawford (2020) [39]
Tending to avoid pushing patients in usual clinical practice	Lawford (2020) [39]
Potential further damage to the knee due to activity	Poitras (2010) [47]
Urging caution to patients about participating in higher impact exercise/activities	Wallis (2020) [52]
Fear of increasing symptoms (as barrier to prescribing exercise)	Holden (2009) [61]
Causing disease progression, particularly through weight-bearing activities (as barrier to prescribing exercise)	Holden (2009) [61]
Exacerbating patient's comorbidities (as barrier to prescribing exercise)	Holden (2009) [61]
General exercise is not/might not be safe for everybody to do (*)	Holden (2009) [61]
Local strengthening exercises for the knee are not/might not be safe for everybody to do (*)	Holden (2009) [61]
LIs are safe (facilitator)	
Quadriceps strengthening exercises for the knee are safe for everybody to do (*)	Cottrell (2016) [53]
General exercise (e.g. walking or swimming) is safe for everybody to do (*)	Cottrell (2016) [53]
Only few drawbacks for the use of non-surgical treatments (*)	Hofstede (2016) [57]
Research environment or protocols provide a safety net (facilitator)	
Less afraid to increase training intensity (preventing adverse events by tailoring programs to individual's capacity)	De Rooij (2014) [31]
Safety net provided by research environment (e.g. patients were previously screened for comorbidities/red flags)	Hinman (2017) [35]
There was a safety net in place with the trial (each patient had been screened)	Lawford (2019) [38]
Experiences in study helped them push patients through more pain than they would have previously	Lawford (2020) [39]

Design

Description	Reference
Non-optimal content or structure of LIs (barrier)	
Structure/timing of exercise program restricted capacity to modify exercises/provide adequate follow-up	Hinman (2016) [34]
Maximum number of four sessions was considered too low in many patients	Knoop (2020) [36]
Behavioral approach in exercise therapy and advice to visit GP were considered unnecessary for most patients	Knoop (2020) [36]
Program factors (e.g. single discipline led intervention)	Wallis (2020) [52]
Services do not meet expectations (*)	Cottrell (2016) [53]
E-Exercise does not/might not contain all essential elements for the treatment of hip/knee OA (*)	Kloek (2020) [62]
The content of e-Exercise is not/might not be aligned with my opinion about treating patients with OA (*)	Kloek (2020) [62]
The intervention provided through e-Exercise is not/might not be appropriate for the average patient with OA (*)	Kloek (2020) [62]
Patients who were treated with e-Exercise were (perhaps) not generally positive about the intervention (*)	Kloek (2020) [62]
Challenges for patients during participation in LIs (barrier)	
Mental effort required for WBE program was challenging for patients	Lawford (2020) [39]
Physical challenge was the complexity of WBE program	Lawford (2020) [39]
Straight leg raise challenging in NWBE program	Lawford (2020) [39]
Volume of resources could be overwhelming/confusing for some patients	Lawford (2021) [40]
Difficulty for patients with PCST component (cognitive restructuring techniques)	Nielsen (2014) [45]
Challenges for HCPs during delivery of LIs (barrier)	
e-Exercise must be adapted for suitable integration into practice (e.g. no insight into modules patients receive)	Bossen (2016) [28]
Class required intense supervision, which was difficult to provide when most participants were new	Davis (2018) [30]
Challenges of supervision when space did not allow clear line of sight	Davis (2018) [30]
Challenges associated with cuff weights used to apply resistance in NWBE program	Lawford (2020) [39]
The lay out of the protocol does not/might not facilitate its usage in daily practice (*)	De Rooij (2020) [60]
Clarity of instruction manual and course (lack of)	Kloek (2020) [62]
Positive experiences with or suggestions for improving the content or structure of LIs (facilitator)	
Positive feedback regarding the content of e-Exercise	Bossen (2016) [28]
First education session was critical to reducing the participant's anxiety related to exercising	Davis (2018) [30]
Importance of empowering the patients rather than 'pushing' them, achieved by 'giving choices'	Davis (2018) [30]
Positive comments about the exercise regimen	Hinman (2016) [34]
Structure provided by protocol/structure of exercises (how patients included them into daily routine)	Hinman (2016) [34]
Multidisciplinary nature of LMP (whole-person, intensive and functional approach)	Law (2019) [37]
Standardization was viewed as important for monitoring and evaluation purposes	Law (2019) [37]
Helpful social impact of group-based programme	Law (2019) [37]
Long-term follow-up consultations would be beneficial	Lawford (2021) [40]
Extremely positive about educational resources provided	Lawford (2021) [40]
More information about healthy eating beyond meal replacement phase could be included	Lawford (2021) [40]
Exercise/physical activity program was an important part of intervention	Lawford (2021) [40]

Favorably comments on program content (positive way to help people be proactive about their pain)	Nielsen (2014) [45]
Importance of PCST component (cognitive restructuring techniques)	Nielsen (2014) [45]
Structure of PCST sessions (overview/practice review/covering new skill/practice planning) worked well	Nielsen (2014) [45]
A more holistic program as part of a multidisciplinary model of service was preferred	Wallis (2020) [52]
Value of program's structure and peer (group) support	Wallis (2020) [52]
Name of program (‘Good Life with OsteoArthritis’) implied optimism and positive outcome	Wallis (2020) [52]
Received positive feedback from their patients about program	Wallis (2020) [52]
Some contents of the protocol are not/might not be incorrect (*)	De Rooij (2020) [60]
The protocol is applicable to OA patients with comorbidity that I see in my clinical practice (*)	De Rooij (2020) [60]
Important to extend the intake phase to at least to 45 min	De Rooij (2020) [60]
Completeness of web-based application (exercises/assignments/information)	Kloek (2020) [62]
Perception that e-Exercise is an appropriate treatment option for subgroup of OA patients	Kloek (2020) [62]
Ease for patients during participation in LIs (facilitator)	
NWBE program was generally easier for patients to follow (mental effort)	Lawford (2020) [39]
Simplicity and convenience of meal replacements	Lawford (2021) [40]
Providing trial of sessions to assist patients to get started (suggestion for promotion and referrals)	Wallis (2020) [52]
Ease for HCPs during delivery of LIs (facilitator)	
Initial classes needed to be small with rolling recruitment very beneficial	Davis (2018) [30]
List of restrictions for exercise therapy was conveniently arranged checklist for diagnostic and treatment phases	De Rooij (2014) [31]
Suggestion to increase feasibility by reducing the protocols to three main protocols	De Rooij (2014) [31]
Requirements of treatment protocol freed therapists to notice and reflect on impact of the interventions	Hinman (2016) [34]
Structured protocol allowed to experience different OA treatment regimen/observe and learn from impact	Hinman (2016) [34]
NWBE program was easier to prescribe (mental effort)	Lawford (2020) [39]
Easier to prescribe and progress NWBE than WBE program (physical complexity)	Lawford (2020) [39]
Training workshop as good introduction to content and process of delivering PCST program	Nielsen (2014) [45]
Weekly group interaction crucial to being able to deliver intervention effectively/problem-solve issues	Nielsen (2014) [45]
Input from supervising psychologist crucial to being able to deliver intervention effectively/problem-solve issues	Nielsen (2014) [45]
Would have liked more role-playing experience prior to beginning trial treatments	Nielsen (2014) [45]
Regular group meetings were considered very important (if not essential) for delivery of PCST program	Nielsen (2014) [45]
Value of having a psychologist involved throughout the program, their professional input was helpful	Nielsen (2014) [45]
The recommendations over adapting the diagnostic phase (history taking and physical examination) in the protocol are clear and understandable (*)	De Rooij (2020) [60]
The recommendations over adapting the OA exercise therapy in the protocol are clear and understandable (*)	De Rooij (2020) [60]
Useful to plan follow up/refreshment training to repeat/discuss content of course/protocol and application	De Rooij (2020) [60]
Shortening the protocol would increase user-friendliness	De Rooij (2020) [60]
The instruction course and manual assisted me so that I knew how to work with e-Exercise (*)	Kloek (2020) [62]

Personalized treatment

Description	Reference
Insufficient ability to provide personalized treatment within LIs (barrier)	
Concerned the service would not be able to provide individualized management for a very diverse population	Egerton (2017) [32]
Requirements of RCT potentially created a barrier to responding to where the client was	Nielsen (2014) [45]
Less satisfied about the applicability of e-Exercise for only one diagnosis	Kloek (2020) [62]
I do not/might not have enough influence on the content of patients' individual e-Exercise program (*)	Kloek (2020) [62]
Ability and importance of providing personalized treatment within LIs (facilitator)	
Flexibility was valuable when tackling local participation challenges	Law (2019) [37]
Tailoring exercise programs to individual patient would overcome some challenges	Lawford (2020) [39]
Tailoring treatment to a person's goals/interests	MacKay (2020) [42]
Need to consider personal context by integrating people's home exercises into daily activities/other life demands	MacKay (2020) [42]
Some modules worked better than others (depending on the individual patient and context)	Nielsen (2014) [45]
The belief that a more flexible approach responsive to patient needs was required in their practice	Nielsen (2014) [45]
Exercise programs have to be individualized to each patient by the PT	Poitras (2010) [47]
Importance of tailored exercise program	Teo (2020) [51]
Exercise for CKP is most beneficial when it is tailored to meet individual patient needs (*)	Cottrell (2016) [53]
A standard set of exercises is not/might not be sufficient for every patient with chronic knee problems (*)	Cottrell (2016) [53]
The protocol gives the opportunity to make your own decisions regarding history taking, physical examination, and treatment (*)	De Rooij (2020) [60]
A standard set of exercises is not/might not be sufficient for every patient with knee OA (*)	Holden (2009) [61]
Exercise for knee OA is most beneficial when it is tailored to meet individual patient needs (*)	Holden (2009) [61]
More flexibility in web-based application (intervention duration, number of sets/repetitions, type of exercises)	Kloek (2020) [62]
More flexibility in intervention (more possibilities to personalize to individual needs)	Kloek (2020) [62]

Accessibility

Description	Reference
LIs are unavailable or inaccessible (barrier)	
Most of the patients did not meet study inclusion criteria	Bossen (2016) [28]
Lack of availability of support services (e.g. community-based rehabilitation programs) in remote locations	Egerton (2018) [33]
Long waiting lists for support services (e.g. community-based rehabilitation programs)	Egerton (2018) [33]
Lack of infrastructure or local programmes (particularly in rural settings)	MacKay (2018) [41]
Clients often had a waiting period before accessing care	MacKay (2018) [41]
Lack of facilities to promote continuing exercise in community	Mann (2011) [43]
Wait for physiotherapy was too long	Mann (2011) [43]
Inaccessible treatment options within organization	Miller (2020) [44]
Limitations to accessing services (e.g. lack of facilities, costs) (*)	Cottrell (2016) [53]

Weight management services are not/might not be adequately commissioned in my area (*)	Hill (2018) [56]
Lack of availability of physiotherapy	Reid (2014) [59]
Poor links to community facilities such as local leisure centres	Holden (2009) [61]
Costs of LIs to patients (barrier)	
Concern that uptake would be negatively impacted if patients were required to pay	Egerton (2017) [32]
Concerns regarding financial cost to patients when considering referral to other services	Egerton (2018) [33]
Cost was a factor in whether clients could access facilities/programmes	MacKay (2018) [41]
Costs to patients (lack of insurance coverage/high co-pays for specific services/time off work/travel expenses)	Miller (2020) [44]
Costs (extrinsic barrier for patient adherence)	Teo (2020) [51]
Cost (program access barrier)	Wallis (2020) [52]
LIs are not feasible or sustainable (barrier)	
Concern for overcomplicated system when service is not compatible/complementary with existing initiatives	Egerton (2017) [32]
Not seeing need (already adequate skills/resources to support OA patient self-management and lifestyle change)	Egerton (2017) [32]
Not seeing need (advice already given at their practice would be unhelpfully repeated)	Egerton (2017) [32]
Concern regarding long-term service sustainability	Egerton (2017) [32]
The addition of a care support team may add complexities to management	Egerton (2017) [32]
In my daily clinical practice I can (perhaps) not integrate working according to the protocol well (*)	De Rooij (2020) [60]
I do not/might not treat enough patients with knee OA and comorbidity to apply the protocol (*)	De Rooij (2020) [60]
The protocol does not/might not fit well with my working methods of daily clinical practice (*)	De Rooij (2020) [60]
Total amount of knee OA patients with comorbidity was lower than expected	De Rooij (2020) [60]
Inconvenience to patients when accessing LIs (barrier)	
Weather (extrinsic barrier for patient adherence)	Teo (2020) [51]
Transport, waiting time and parking related to attendance (program access barrier)	Wallis (2020) [52]
Geography (program access barrier)	Wallis (2020) [52]
Available session times (program access barrier)	Wallis (2020) [52]
Geographical problems (e.g. remote location, scared to walk in local area) (*)	Cottrell (2016) [53]
LIs are available or accessible, or suggestions for improvement (facilitator)	
More likely to engage with the care support team if it enabled more affordable/accessible allied health	Egerton (2017) [32]
LMP would benefit from extension of inclusion criteria (patients with less severe OA and lower BMI)	Law (2019) [37]
Benefits of having infrastructure and programmes available in their communities	MacKay (2018) [41]
Triage service (as suggestion for physiotherapy service improvement)	Okwera (2019) [46]
A web-based physiotherapy service (as suggestion for physiotherapy service improvement)	Okwera (2019) [46]
Reduced waiting times (as suggestion for physiotherapy service improvement)	Okwera (2019) [46]
Availability of non-surgical treatments (*)	Hofstede (2016) [57]
Good access to physiotherapy in area (*)	Reid (2014) [59]
LIs are feasible or sustainable (facilitator)	
Need for clarity about how the new service would integrate with existing schemes and payment structures	Egerton (2017) [32]

Seeing need (advice/recommendations may need to be reinforced/provided over several health care episodes)	Egerton (2017) [32]
Seeing need (extra time and encouragement for the patient would result in better outcomes)	Egerton (2017) [32]
Importance of broad acceptance (patients/doctors/health service funders) if new service is to continue long term	Egerton (2017) [32]
Model of stratified care easy to apply and having added value for daily practice	Knoop (2020) [36]
Appreciation of applicability of treatment protocols	Knoop (2020) [36]
Further and ongoing evaluation of the LMP would help to address current challenges	Law (2019) [37]
Interventions in physical therapists' toolbox were not static (changed over time)	MacKay (2020) [42]
The protocol is feasible in daily clinical practice (*)	De Rooij (2020) [60]
In my daily clinical practice, I work with sufficient equipment (including blood pressure meter, saturation meter) to properly apply the protocol (*)	De Rooij (2020) [60]
I have changed my working method (due to the protocol) (*)	De Rooij (2020) [60]
Intake procedure is feasible and implementable	De Rooij (2020) [60]
The more you apply the strategy in daily practice, the easier it is to integrate it in your daily working method	De Rooij (2020) [60]
Convenience for patients when accessing LIs (facilitator)	
Close, convenient locations (suggestion for promotion and referrals)	Wallis (2020) [52]
Appropriate session times for working populations (suggestion for promotion and referrals)	Wallis (2020) [52]
Provision of free parking at health service (suggestion for promotion and referrals)	Wallis (2020) [52]

Telehealth

**Description**	**Reference**
Disadvantages of telehealth in terms of effectiveness (barrier)	
Remote (telephone) delivery is not as good as face-to-face particularly in relation to exercise advice	Egerton (2017) [32]
Inability of a remote service to provide locally relevant information	Egerton (2017) [32]
An exercise program prescribed by a PT over the telephone would not/might not improve a patient's OA (*)	Lawford (2018) [58]
I do not/might not experience that e-Exercise supports patients in doing their exercises at home (*)	Kloek (2020) [62]
Telehealth is not safe for patients or patient/data privacy (barrier)	
Concerns about security of patient data and information confidentiality during the referral process	Egerton (2017) [32]
Using the telephone would not/might not be a safe way for patients to receive a PT-prescribed exercise program for their OA (*)	Lawford (2018) [58]
Challenges for HCPs regarding lack of physical/visual contact (barrier)	
Forced to modify usual habits/rely more on information shared by patients (instead of own physical assessment)	Hinman (2017) [35]
Some discomfort without hands-on assessment (no palpation of patient's knee/hands-on facilitation of exercises)	Hinman (2017) [35]
Assessment of patients could be difficult when consulting via telephone (inability to observe)	Lawford (2019) [38]
Lack of visual/physical contact would limit strategies available when teaching patients an exercise program	Lawford (2019) [38]
I would not/might not be able to adequately monitor a patient's OA over the telephone (*)	Lawford (2018) [58]
I do not/might not like that there would be no physical contact with an OA patient when consulting over the telephone (*)	Lawford (2018) [58]
I do not/might not like that there would be no physical contact with an OA patient when consulting over the internet video (*)	Lawford (2018) [58]
Reduced face-to-face contact interfered with professional autonomy	Kloek (2020) [62]

Other challenges for HCPs regarding feasibility of telehealth (barrier)	
Lack of financial incentive if blended intervention substitutes conventional visits (reduced venues per patient)	Bossen (2016) [28]
Hesitancy to embrace an unfamiliar new service	Egerton (2017) [32]
Patient flexibility could come at a cost to the therapist sometimes (allowed patients to reschedule last minute)	Hinman (2017) [35]
Skype consultations more suitable as adjunctive to usual in-clinic care (initial assessment in person preferred)	Hinman (2017) [35]
Telephone not viewed as primary mode of providing care (only for follow-up)	Lawford (2019) [38]
Some difficulty scheduling telephone consultations during usual day of face-to-face consultations	Lawford (2019) [38]
Using the telephone to consult with an OA patient and prescribe an exercise program would not/might not be easy for me (*)	Lawford (2018) [58]
I would not/might not be as satisfied talking to an OA patient over the telephone as I would be talking to the patient in person in my consulting room (*)	Lawford (2018) [58]
I would not/might not be interested in being involved in a service offering PT-prescribed exercise over the telephone for my people with OA (*)	Lawford (2018) [58]
Using the telephone would not/might not be an acceptable way for me to deliver an exercise program to patients with OA (*)	Lawford (2018) [58]
Using the telephone would not/might not be a useful (practical) way for me to deliver an exercise program to patients with OA (*)	Lawford (2018) [58]
Using the telephone would not/might not be an effective way for me to deliver an exercise program to patients with OA (*)	Lawford (2018) [58]
Technical skills (lack of)	Kloek (2020) [62]
Adaptive capacity to change treatment routines (lack of)	Kloek (2020) [62]
Absence of national e-Health guideline or standard	Kloek (2020) [62]
Loss of income due to substitution of face-to-face session	Kloek (2020) [62]
Patient-related challenges regarding feasibility of telehealth (barrier)	
Most of the patients prefer traditional face-to-face treatments	Bossen (2016) [28]
Hearing and cognitive difficulties as barriers for some patients to being able to interact with the service	Egerton (2017) [32]
Skepticism about whether many patients would embrace such a model (i.e. because of remote-delivery aspect)	Egerton (2017) [32]
Lack of technology affinity (reason for patients' non-willingness to participate in e-Exercise)	Kloek (2020) [62]
Patients preferred regular face-to-face contact	Kloek (2020) [62]
Negative aspects regarding communication and relationship using telehealth (barrier)	
Lack of face-to-face contact difficult/hampered ability to establish normal rapport/build effective relationships	Hinman (2016) [34]
Occasional technical difficulties (e.g. poor internet connection) could disrupt the flow of the consultation	Hinman (2017) [35]
Relationships with patients might be adversely impacted/could be difficult to develop rapport	Lawford (2019) [38]
Difficulties communicating might be experienced when consulting via telephone	Lawford (2019) [38]
Video consultations made it more difficult to have emotional conversations/read non-verbal cues	Lawford (2021) [40]
Benefits of telehealth in terms of effectiveness (facilitator)	
Possibility to extend physical therapy treatment in patient's home environment	Bossen (2016) [28]
Potential to enhance the adherence of home exercises	Bossen (2016) [28]
Empowering effect of home environment on patient adherence with exercise program	Hinman (2017) [35]
Using Skype distilled focus to most important and effective treatment elements to facilitate self-management	Hinman (2017) [35]
Patients more relaxed in home environment/more receptive to the information the therapists provided	Hinman (2017) [35]
Patients could be more comfortable talking about condition/engaging in exercise program from own home	Lawford (2019) [38]

Telephone-delivered care could provide increased opportunities to educate patients about OA	Lawford (2019) [38]
Patient adherence to telephone-delivered exercise program was high	Lawford (2019) [38]
An exercise program prescribed by a PT over the internet video would improve a patient's OA (*)	Lawford (2018) [58]
Added value in terms of exercise adherence (important factor to use web-based application)	Kloek (2020) [62]
Telehealth is safe for patients or patient/data privacy (facilitator)	
Home environment facilitated correct and safe exercise techniques	Hinman (2017) [35]
A patient's privacy would not be violated if I prescribed them an exercise program over the telephone (*)	Lawford (2018) [58]
A patient's privacy would not be violated if I prescribed them an exercise program over the internet video (*)	Lawford (2018) [58]
Using the internet video would be a safe way for patients to receive a PT-prescribed exercise program for their OA (*)	Lawford (2018) [58]
I believe that patient data gathered at the e-Exercise web-application is stored safely (*)	Kloek (2020) [62]
Lack of physical/visual contact not a major issue for HCPs (facilitator)	
Patients responded favorably to the exercises prescribed despite lack of hands-on assessment	Hinman (2017) [35]
Hands-off approach was physically less demanding compared to usual care/contributed to sense of satisfaction	Hinman (2017) [35]
Functional improvements were observable using Skype	Hinman (2017) [35]
Lack of physical and visual contact less of an issue than anticipated	Lawford (2019) [38]
Able to work around the lack of visual contact (erring on the side of caution)	Lawford (2019) [38]
I would get a good understanding of a patient's OA over the telephone (*)	Lawford (2018) [58]
I would get a good understanding of a patient's OA over the internet video (*)	Lawford (2018) [58]
I would be able to adequately monitor a patient's OA over the internet video (*)	Lawford (2018) [58]
Positive attitude or needs of HCPs regarding feasibility of telehealth (facilitator)	
Ease of using Skype for consultations	Hinman (2017) [35]
Quality of technology suitable for providing instructions/prescribing exercises/receiving instantaneous feedback	Hinman (2017) [35]
More effective communication skills would be needed to consult via telephone	Lawford (2019) [38]
It would be necessary to provide patients with pictures or videos of each exercise when consulting via telephone	Lawford (2019) [38]
Experiences providing telephone-delivered care exceeded expectations, resulting in new enthusiasm	Lawford (2019) [38]
Written materials provided to patients helped to prescribe exercises effectively	Lawford (2019) [38]
Training in communication and/or health coaching important to effectively deliver care over telephone	Lawford (2019) [38]
Video consultations were easy and convenient	Lawford (2021) [40]
Using the internet video to consult with an OA patient and prescribe an exercise program would be easy for me (*)	Lawford (2018) [58]
I would be as satisfied talking to an OA patient over the internet video as I would be talking to the patient in person in my consulting room (*)	Lawford (2018) [58]
I would be interested in being involved in a service offering PT-prescribed exercise over the internet video for my people with OA (*)	Lawford (2018) [58]
Using the internet video would be an acceptable way for me to deliver an exercise program to patients with OA (*)	Lawford (2018) [58]
Using the internet video would be a useful (practical) way for me to deliver an exercise program to patients with OA (*)	Lawford (2018) [58]
Using the internet video would be an effective way for me to deliver an exercise program to patients with OA (*)	Lawford (2018) [58]
Advantage of reducing number of treatments	Kloek (2020) [62]
Offering an innovative intervention attracted new patients	Kloek (2020) [62]
That it results in less income is not/might not be a major disadvantage of e-Exercise (*)	Kloek (2020) [62]

Our physiotherapy practice has the intention to use e-Health innovations (*)	Kloek (2020) [62]
Patient-related benefits regarding feasibility of telehealth (facilitator)	
24/7 availability of information and exercises	Bossen (2016) [28]
Service could increase access to support for rural patients	Egerton (2017) [32]
Skype-delivered care convenient for patients (time efficiency/flexibility/access)	Hinman (2017) [35]
Telephone-delivered care would be convenient for patients	Lawford (2019) [38]
Telephone-delivered care could reduce patient costs associated with accessing physiotherapy services	Lawford (2019) [38]
Telephone-delivered care could allow wider variety of patients to access physiotherapy	Lawford (2019) [38]
Noticeable shift in patients' expectations of physiotherapy care (more willing to self-manage their condition)	Lawford (2019) [38]
Telephone-delivered care was convenient for patients	Lawford (2019) [38]
An exercise program prescribed by a PT over the telephone would save a patient money (*)	Lawford (2018) [58]
An exercise program prescribed by a PT over the internet video would save a patient money (*)	Lawford (2018) [58]
Receiving an exercise program from a PT over the telephone would be a convenient form of health care for an OA patient (*)	Lawford (2018) [58]
Receiving an exercise program from a PT over the internet video would be a convenient form of health care for an OA patient (*)	Lawford (2018) [58]
Receiving an exercise program from a PT over the telephone would save the patient time (*)	Lawford (2018) [58]
Receiving an exercise program from a PT over the internet video would save the patient time (*)	Lawford (2018) [58]
Using the telephone would be an affordable way for patients to receive a PT-prescribed exercise program for their OA (*)	Lawford (2018) [58]
Using the internet video would be an affordable way for patients to receive a PT-prescribed exercise program for their OA (*)	Lawford (2018) [58]
Positive aspects regarding communication and relationship using telehealth (facilitator)	
Developed a strong rapport with patients over the telephone	Lawford (2019) [38]
Consulting via telephone forced to focus on effective conversations with patients (more personal level)	Lawford (2019) [38]
Pleasantly surprised by experience with video consultations (had some of the best conversations)	Lawford (2021) [40]

Domain 2: Individual HCP factors

Expertise

**Description**	**Reference**
Lack of knowledge or skills around LIs or promoting behavioral change (barrier)	
Lack of knowledge around appropriate interventions for weight loss	Allison (2019) [27]
Uncertainty about how to enact their understanding of relationship between weight and knee OA	Allison (2019) [27]
Limited knowledge of exercise prescription (uncertainty of what exercise to recommend/how much)	Christiansen (2020) [29]
Not received sufficient training on exercise/lack of education	Christiansen (2020) [29]
Knowledge of exercise and weight-loss treatments is sometimes inaccurate or inadequate	Egerton (2018) [33]
Reduced confidence with providing suitable exercise and weight loss advice	Egerton (2018) [33]
Lack of skills in promoting readiness and motivation for lifestyle treatments	Egerton (2018) [33]
Variability in confidence to provide weight management (not confident)	MacKay (2018) [41]

Not confident in knowledge about weight management	MacKay (2020) [42]
Concerns about capacity to learn/not having skills to fulfill study expectations/deal with challenging patients	Nielsen (2014) [45]
Not have sufficient skills to present PCST component (cognitive restructuring techniques) effectively	Nielsen (2014) [45]
Some process skills were dissimilar to pre-existing clinical communication skills and challenging to use	Nielsen (2014) [45]
Lack of knowledge about CBT (necessary to participate in training/RCT to fully appreciate value of CBT to practice)	Nielsen (2014) [45]
Unclear on amount and type of activity necessary to obtain benefits without further damaging the knee	Poitras (2010) [47]
Uncertainties about dosage/frequency/type of physical activity	Selten (2017) [49]
Unaware about practice guidelines in relation to aerobic exercise prescription/weight loss/pain management	Tang (2020) [50]
Knowledge about BMI/weight management was particularly poor (e.g. relying on visual estimations)	Tang (2020) [50]
Limited knowledge of how to address weight management	Tang (2020) [50]
Less awareness about aerobic exercise prescription	Tang (2020) [50]
Reduced confidence with recommending individual weight/pain management plans (discuss in general terms)	Tang (2020) [50]
Lack of confidence/knowledge/skills in implementing evidence into practice (e.g. weight management)	Teo (2020) [51]
I don't have/might not have the required knowledge and training around obesity care (*)	Hill (2018) [56]
I do not/might not have sufficient knowledge about knee OA exercise therapy and comorbidity to apply the protocol in daily clinical practice (*)	De Rooij (2020) [60]
Gaps in knowledge/skills (including how to facilitate behavior change, particularly with less motivated patients)	Holden (2009) [61]
Lack of knowledge or skills around OA care in general (barrier)	
General lack of expertise/interest in OA (that could lead to inappropriate referral/suboptimal access to services)	Mann (2011) [43]
Lack of physician education on OA care	Miller (2020) [44]
Only two GPs had clear understanding of clinical guidelines on OA	Okwera (2019) [46]
No knowledge about treatment	Rosemann (2006) [48]
Lack of knowledge or skills around specific resources (barrier)	
The issue is not a lack of suitable patient resources but awareness of them	Egerton (2018) [33]
Inability to discuss specific details of ACSM guideline	Tang (2020) [50]
I do not/might not have sufficient skills to apply the protocol in daily clinical practice (*)	De Rooij (2020) [60]
I do not/might not read the protocol sufficiently to remember any of its contents (*)	De Rooij (2020) [60]
Having or improving knowledge or skills around LIs or promoting behavioral change (facilitator)	
Importance of having highly effective communication skills	Egerton (2018) [33]
Value of monitoring/encouraging patients to develop own understanding of links between exercise/pain	Hinman (2016) [34]
Confident in capabilities/skills to use strategies they believed to be effective within scope of practice	MacKay (2018) [41]
Variability in confidence to provide weight management (confident)	MacKay (2018) [41]
Clinical experience helped to read the person’s situation (identify approach to motivate them)	MacKay (2020) [42]
Postgraduate continuing professional development courses to expand toolkit of therapeutic interventions	MacKay (2020) [42]
Confidence in addressing weight management	MacKay (2020) [42]
Opportunity to review PCST skills and learn more structured/deliberate ways of incorporating these into practice	Nielsen (2014) [45]
Increasing confidence in using PCST skills over the course of the study	Nielsen (2014) [45]
Improved interpersonal skills with general clinical patients as a result of participating in the study	Nielsen (2014) [45]

Reasonable understanding of role physiotherapy plays in management of lower-limb OA	Okwera (2019) [46]
Knowledge/confidence in providing treatments related to strengthening and range of motion	Tang (2020) [50]
Confident in providing justifications for non-routinely adhering to guidelines (range of motion exercises)	Tang (2020) [50]
Being able to describe how they will manage pain during strengthening exercise	Tang (2020) [50]
Having or improving knowledge or skills around OA care in general (facilitator)	
Need for tailored GP education to improve confidence	Egerton (2018) [33]
Importance of provider knowledge regarding OA management	Miller (2020) [44]
Physician education on OA management can affect both provider and patient attitudes	Miller (2020) [44]
Training sessions (as suggestion for physiotherapy service improvement)	Okwera (2019) [46]
Knowledge about treatment	Rosemann (2006) [48]
Available resources might improve knowledge and decision-making (facilitator)	
Protocols offered guidance in setting up treatment/making clinical decisions/adapting treatment to comorbidity	De Rooij (2014) [31]
List of restrictions was helpful in process of clinical decision making	De Rooij (2014) [31]
Being aware about ACSM guidelines	Tang (2020) [50]
The protocol supports me in clinical reasoning (*)	De Rooij (2020) [60]
The protocol is supporting the improvement of my knowledge regarding knee OA exercise therapy and comorbidity (*)	De Rooij (2020) [60]
The protocol is supportive in which comorbidity-related symptoms I need to monitor before, during and after treatment (*)	De Rooij (2020) [60]
More insight into exercise tolerance/more background knowledge to make clinical decision by using strategy	De Rooij (2020) [60]
Clinical experience (unclear factor)	
Driven by their professional experience of what does and doesn’t work/trial and error	MacKay (2020) [42]
Treatments were based more on what works clinically (opposed to scientific evidence)	MacKay (2020) [42]

Attitude

Description	Reference
Negative attitude toward LIs (barrier)	
Assigning low priority to exercise as treatment	Christiansen (2020) [29]
GP does not prioritise exercise (*)	Cottrell (2016) [53]
It is not/might not be important that people with knee OA increase their overall activity levels (*)	Holden (2009) [61]
Negative attitude toward guidelines or protocols (barrier)	
Frustrations about lack of autonomy with decision-making	Okwera (2019) [46]
Negativity toward guidelines (clinical reasoning more important)	Okwera (2019) [46]
Positive attitude toward LIs (facilitator)	
Acknowledging that weight loss (when someone is overweight) is important	Egerton (2018) [33]
Identifying weight management as important	MacKay (2018) [41]
Perception that exercise and physical activity were central to management	MacKay (2020) [42]
Acknowledging that weight management was a component of management	MacKay (2020) [42]

Expecting to utilize/continue integrating PCST in general clinical work as physical therapist (beyond the study)	Nielsen (2014) [45]
There is place for each (self-management programs/physiotherapy/orthopedic consultants) in OA management	Okwera (2019) [46]
Many interventions should be used before resorting to medication (including physiotherapy)	Poitras (2010) [47]
Other interventions (including physiotherapy) should be used before paracetamol	Poitras (2010) [47]
NSAIDs alone are not sufficient to appropriately treat inflammation and have to be combined with physiotherapy	Poitras (2010) [47]
Patients should be encouraged to resume/maintain daily activities	Poitras (2010) [47]
Knowing the importance of weight management for knee OA	Tang (2020) [50]
Aware that being overweight/obese is risk factor for knee OA/losing weight is important	Teo (2020) [51]
Physiotherapy (referral) needs to be prioritised	Cottrell (2016) [53]
It is important that people with CKP increase their overall activity levels (*)	Cottrell (2016) [53]
Exercise for CKP should (perhaps) preferably be used before drug treatment has been tried (*)	Cottrell (2016) [53]
Weight loss should be the first-line treatment in the management of obese patients with symptomatic knee OA (*)	Hill (2018) [55]
Obese patients with symptomatic knee OA should be referred to a specialist weight management service before orthopaedic assessment (*)	Hill (2018) [55]
Weight loss should be the first-line treatment in the management of symptomatic knee OA in obesity (*)	Hill (2018) [56]
Support for creation of regional centres where orthopaedic surgeons and bariatric surgeons, with their respective teams, could assess obese patients with symptomatic knee pain (*)	Hill (2018) [56]
Important to try non-surgical treatments first (*)	Hofstede (2016) [57]
Important to delay a surgery as long as possible (*)	Hofstede (2016) [57]
Conservative treatment is (perhaps) an important part of OA management (*)	Reid (2014) [59]
Positive attitude toward guidelines or protocols (facilitator)	
Perceived professional responsibility to adhere to evidence-based guideline	Allison (2019) [27]
Important to follow guidelines (*)	Hofstede (2016) [57]
In general, I do not/might not feel resistance towards working according to protocols (*)	De Rooij (2020) [60]
Autonomy (unclear factor)	
Autonomy affects referral considerations	Law (2019) [37]

Role

**Description**	**Reference**
Perception of own role potentially impeding prescription or follow-up of LIs (barrier)	
Referring patients to other health care providers and for other treatments rather than recommending exercise	Christiansen (2020) [29]
Referring patients to those with specialized knowledge rather than treating themselves (outside scope of practice)	Christiansen (2020) [29]
Paternalistic approach to care (low level of engagement in providing exercise and weight management advice)	Egerton (2018) [33]
Lack of confidence/uncertainty related to role in weight management	MacKay (2020) [42]
Perception that discussions related to diet were not part of their scope of practice	MacKay (2020) [42]
Changing own practice style remained as barrier after OA training	Miller (2020) [44]
Most GPs believed their contribution was essentially limited to diagnosis of condition and medication	Poitras (2010) [47]

Not focusing on increasing patients' motivation for behavioural change, but just giving general recommendations	Rosemann (2006) [48]
Mentioning benefits of weight reduction, but not actively coaching or referring patients	Selten (2017) [49]
Not perceiving weight reduction advice as their responsibility	Selten (2017) [49]
Uncertainty over scope of practice/questioning whether weight and pain management fall outside scope	Tang (2020) [50]
Describing own role as prepping patients for knee surgery when they were referred for physiotherapy	Teo (2020) [51]
Advice about how to lose weight was limited to brief general advice	Teo (2020) [51]
Considering weight loss to be outside own scope of practice (role of a dietician)	Teo (2020) [51]
Comfortable suggesting surgery to patients who responded poorly to conservative management	Teo (2020) [51]
GPs should (perhaps) not follow-up patients to monitor extent of continuation of exercises (*)	Cottrell (2016) [53]
It is the patient's own responsibility to continue doing their exercise programme (*)	Cottrell (2016) [53]
Not (or perhaps not) interested in being the orthopedic surgeon in an ortho-bariatric centre (*)	Hill (2018) [55]
Therapist's role seen as assessment/exercise prescription/education (relatively short-term responsibilities)	Holden (2009) [61]
Patient's role to follow prescribed exercise program over long term/get on board with treatment	Holden (2009) [61]
Physical therapists should (perhaps) not prescribe general exercise for every patient with knee OA (*)	Holden (2009) [61]
It is the patient's own responsibility to continue doing their exercise program (*)	Holden (2009) [61]
It is not/might not be the physiotherapist's responsibility to make sure that the patient will continue doing their exercise program (*)	Holden (2009) [61]
Negative consequences for own role when referring patients to LIs (barrier)	
Job satisfaction may be diminished when handing over care of their patients to third party with no involvement	Egerton (2017) [32]
The addition of a care support team may lead them feeling disconnected with their patient's care	Egerton (2017) [32]
Perception of own role potentially stimulating prescription or follow-up of LIs (facilitator)	
Patient-centred approach (high level of engagement in providing exercise and weight management advice)	Egerton (2018) [33]
Viewing themselves as having an important role in supporting clients to participate in management	MacKay (2018) [41]
Routinely including education about weight management	MacKay (2020) [42]
Playing a role in promoting engagement in management	MacKay (2020) [42]
Necessity of physiotherapy to effectively rehabilitate knee OA patients (because of knowledge/availability)	Poitras (2010) [47]
Importance of PT's role in educating patients with regards to NSAIDs/alternatives (including physiotherapy)	Poitras (2010) [47]
Necessity of PT involvement in managing activity (because potentially detrimental if excessive)	Poitras (2010) [47]
Exercise planning is usually PT's role (rather than GP's)	Poitras (2010) [47]
Necessity of PT follow-up sessions to assess and encourage patient adherence	Poitras (2010) [47]
PT's role to individualize patients' activity according to needs and capacity	Poitras (2010) [47]
Although agreeing with active patient participation, it is ultimately PT's role to appropriately manage patients	Poitras (2010) [47]
Desire to be more involved in life style counselling (upgrade of profession)	Rosemann (2006) [48]
Perceiving exercise prescription to be their main role	Teo (2020) [51]
Advising patients against surgery for as long as possible (last option)	Teo (2020) [51]
Implementing several strategies to boost adherence	Teo (2020) [51]
Not their role to advise the patient about knee surgery, opting not to discuss surgery at all	Teo (2020) [51]
Advising patients against knee arthroscopy if specifically asked about this procedure	Teo (2020) [51]

It is part of my job to reassure patients about the safety of exercise for CKP (*)	Cottrell (2016) [53]
GPs should educate patients with CKP about how to change their lifestyle for the better (*)	Cottrell (2016) [53]
GPs should prescribe quadriceps strengthening exercises to every patient with CKP (*)	Cottrell (2016) [53]
GPs should prescribe general exercise (e.g. walking or swimming) for every patient with CKP (*)	Cottrell (2016) [53]
There was a BMI threshold above which they would not perform a TKA at all (*)	Hill (2018) [55]
There was a BMI threshold above which they would not perform a TKA until the patient had attended a weight management program (*)	Hill (2018) [55]
Intention to refer patients to an ortho-bariatric centre if it existed (*)	Hill (2018) [56]
Recognizing potential influence on exercise adherence, sharing responsibility of exercise adherence with patient	Holden (2009) [61]
Physical therapists should prescribe local strengthening exercise for every patient with knee OA (*)	Holden (2009) [61]
Physiotherapists should educate chronic patients with knee OA about how to change their lifestyle for the better (*)	Holden (2009) [61]
Positive consequences for own role when referring patients to LIs (facilitator)	
Some appeal for a lessening of their own responsibility in terms of managing this condition	Egerton (2017) [32]

Domain 3: Patient factors

Health status

**Description**	**Reference**
Severity of disease and symptoms (barrier)	
Level of disease severity (i.e. whether people with very mild or very severe joint disease would benefit)	Egerton (2017) [32]
Patients delay care until they are highly symptomatic (missing opportunities to slow disease progression)	Miller (2020) [44]
Rehabilitation potential depended on length of disability (less potential with late management)	Poitras (2010) [47]
Paracetamol could mask pain/underlying physical problem (reducing opportunity to assess/manage problem)	Poitras (2010) [47]
Difficult to obtain effective analgesia with some patients	Poitras (2010) [47]
Potential to create unrealistic expectations and discouragement in patients that were too disabled	Poitras (2010) [47]
Questioning capacity to perform regular exercise because of severity of disability	Poitras (2010) [47]
Knee pain restricts activities in general (which makes weight loss difficult)	Poitras (2010) [47]
Pain (main barrier resulting in reduced dosage prescription of strengthening exercises)	Tang (2020) [50]
Patient's ability to exercise (main barrier resulting in reduced dosage prescription of strengthening exercises)	Tang (2020) [50]
Pain (key barrier to prescription of exercise as recommended by CPGs)	Tang (2020) [50]
Pain (barrier to prescription of aerobic exercise)	Tang (2020) [50]
Osteoarthritis severity (mild/severe) (patient-related barrier)	Wallis (2020) [52]
Effectiveness related to severity of joint damage/pain level (less effective when more damage/pain)	Holden (2009) [61]
Reluctant to promote exercise in the presence of pain	Holden (2009) [61]
Pain (as patient-centered barrier to adherence)	Holden (2009) [61]
Exercises are not/might not be effective for patients if an X-ray shows severe knee osteoarthritis (*)	Holden (2009) [61]
Negative impact of comorbidities (barrier)	

Difficulty with managing multiple conditions/tendency to prioritize other conditions over OA	Christiansen (2020) [29]
Significant impact of other health problems on patients' ability to commit fully to exercise program	Lawford (2020) [39]
Patient body weight (overweight/obese) (impedes exercise/makes visits to services more difficult)	Miller (2020) [44]
Questioning capacity to perform regular exercise because of general health	Poitras (2010) [47]
Depression as important barrier to motivate patients to physical exercise	Rosemann (2006) [48]
Comorbidities (often more severe pain, hampering ability to exercise or be physically active)	Teo (2020) [51]
Existing comorbidities (patient-related barrier)	Wallis (2020) [52]
Other patient characteristics (barrier)	
Person had been sedentary throughout life	Poitras (2010) [47]
Questioning capacity to perform regular exercise because of age	Poitras (2010) [47]
Patients with knee OA tended to be older/less active/with slower metabolism (which makes weight loss difficult)	Poitras (2010) [47]
Older age (patient-related barrier)	Wallis (2020) [52]
Poor rate of previous (physiotherapy) success (*)	Reid (2014) [59]
Severity of disease and symptoms (facilitator)	
Most patients tolerated a lot more than was expected (amount of exercise)	Lawford (2020) [39]
Rehabilitation potential depended on length of disability (better outcomes with early management)	Poitras (2010) [47]
Effective analgesia necessary for patients to be able to accomplish activities	Poitras (2010) [47]
Exercise is effective for patients if an X-ray shows severe knee OA (*)	Cottrell (2016) [53]
Referring patients to physiotherapy if they had high levels of pain/disability and where radiographic evidence of OA was present (*)	Reid (2014) [59]
Effectiveness related to severity of joint damage/pain level (more effective when less damage/pain)	Holden (2009) [61]
Exercises are effective for patients if an X-ray shows moderate knee osteoarthritis (*)	Holden (2009) [61]
Exercises are effective for patients if an X-ray shows mild knee osteoarthritis (*)	Holden (2009) [61]
Other patient characteristics (facilitator)	
Clients' pre-existing activity level (e.g. active person)	MacKay (2018) [41]
Importance of evaluating a patient's overall functional ability (rather than only knee signs/symptoms)	Teo (2020) [51]
Referring patients to physiotherapy if they were of a younger age (*)	Reid (2014) [59]
Severity of disease and symptoms (unclear factor)	
Treatment decisions depended on people's symptoms/findings of physical assessment	MacKay (2020) [42]
Management strategies depended on how bad the knee is	Okwera (2019) [46]
Exercise does not/might not work just as well for everybody, regardless of the amount of pain they have (*)	Cottrell (2016) [53]
Exercise does not/might not work just as well for everybody, regardless of the amount of pain they have (*)	Holden (2009) [61]
Other patient characteristics (unclear factor)	
Clients' general health	MacKay (2018) [41]

Treatment expectations and preferences

Description	Reference

Negative attitude toward LIs (barrier)	
Patients' lack of motivation to exercise/patients want passive treatment approach or quick fix	Christiansen (2020) [29]
Patients often have own ideas on management (problematic if primarily passive treatments)	Egerton (2018) [33]
Reluctance from patients to talk about physical activity (physical therapist's role, not the coach's role)	Hinman (2016) [34]
Interdisciplinary consult with dietician could not always take place because patients refused to visit dietician	Knoop (2020) [36]
People that don't particularly like exercise	Lawford (2020) [39]
Disconnect between PTs' recommendations for treatment and clients' expectations or preferences	MacKay (2018) [41]
Prior experiences with physical therapy influenced client expectations of clinical encounter	MacKay (2018) [41]
People's preferences were at odds with physical therapists' beliefs about management	MacKay (2020) [42]
Doubts about patients' willingness to make behavioral changes	Mann (2011) [43]
Patients don't want to expend effort towards lifestyle change	Miller (2020) [44]
Public expectation of what physical therapy treatment should be (e.g. didn't come to have thinking challenged)	Nielsen (2014) [45]
Feeling that patients tended to prefer treatment administered to them	Okwera (2019) [46]
Negative comments about patient reports of a lack of "hands-on" physiotherapy	Okwera (2019) [46]
Benefits obtained in the long term, which often conflicted with patient expectations for short-term benefits	Poitras (2010) [47]
Lack of patient motivation in remaining active despite knee OA	Poitras (2010) [47]
Patient views and expectations rarely matched patient needs	Poitras (2010) [47]
Questioning capacity to perform regular exercise because of motivation	Poitras (2010) [47]
Success rate in motivating patients too low (distinctly resignated regarding their impact on patients' life style)	Rosemann (2006) [48]
Frustration about impact of information (e.g. self-help groups) (lot of patients find excuses not to participate)	Rosemann (2006) [48]
Self-motivation (intrinsic barrier for patient adherence)	Teo (2020) [51]
Patient expectations (not keen to participate in exercise/play active role in management, desire for quick fix)	Teo (2020) [51]
Lack of motivation to participate active lifestyle interventions (patient-related barrier)	Wallis (2020) [52]
Existing relationships with physiotherapists (as barrier to referral if patient already had treating physiotherapist)	Wallis (2020) [52]
Exercise does not match patient needs/expectations (*)	Cottrell (2016) [53]
The patients are not/might not be cooperative in applying the protocol in daily clinical practice (*)	De Rooij (2020) [60]
Patients with knee OA and comorbidity are not always motivated to perform exercises	De Rooij (2020) [60]
Lack of motivation or laziness (as patient-centered barrier to adherence)	Holden (2009) [61]
Negative treatment expectations (as patient-centered barrier to adherence)	Holden (2009) [61]
Positive attitude toward TJA (barrier)	
Patients' ideas about whether they wanted surgery influenced making referrals to the LMP	Law (2019) [37]
Unrealistic expectations of the outcome of joint replacement among patients	Mann (2011) [43]
Feeling pressure by patients to refer them to specialist	Rosemann (2006) [48]
Make use of patients' preference for TJA within LIs (facilitator)	
Suggestion to relist patients completing the programme further up the waiting list (for surgery)	Law (2019) [37]
Using bargaining techniques centering on implications of LMP for replacement surgery (put patient on the list)	Law (2019) [37]
Patients' preferences (unclear factor)	

Management strategies depended on what the person wants	Okwera (2019) [46]

Active participation

Description	Reference
Low patient adherence or engagement (barrier)	
Shifting patients' mind-sets to active participation/making lifestyle changes was challenging/time consuming	Egerton (2018) [33]
How well they felt the individual patient would engage with programme influenced making referrals to the LMP	Law (2019) [37]
Challenging work to get clients to initiate management and maintain it over the long term	MacKay (2018) [41]
Getting buy-in (engaging people in management) often portrayed as challenge	MacKay (2020) [42]
Lack of compliance with home exercise regimes and advice given to patients was common	Okwera (2019) [46]
Patients' adherence to management recommendations was limited (because of fatalism)	Poitras (2010) [47]
Patients' unsatisfactory adherence to exercise programs	Teo (2020) [51]
Main concern was participant adherence to physical activity routines after end of program	Duarte (2019) [54]
Negative perceptions of patients' levels of exercise adherence	Holden (2009) [61]
High patient adherence or engagement (facilitator)	
Positive impact of information, education, and structured monitoring on patients' adherence to exercise	Hinman (2016) [34]
Patients adherent/easy to work with when they engaged in exercise program/started seeing improvements	Lawford (2020) [39]
Cohort of patients, in general, was highly motivated (remained interested/motivated for entirety of 6 months)	Lawford (2021) [40]
Enthusiastic participation of the participants	Duarte (2019) [54]
Importance of high patient adherence or engagement for effectiveness of LIs (facilitator)	
Exercise progression was most effective when the participant requested progression	Davis (2018) [30]
Client participation in management was critical to see improvement in symptoms	MacKay (2018) [41]
Getting buy-in (engaging people in management) critical to improving outcomes	MacKay (2020) [42]
Necessity of patients' active participation in knee OA management (to achieve significant outcomes)	Poitras (2010) [47]
How well a patient complies with their exercise programme determines how effective it will be (*)	Cottrell (2016) [53]
Importance of exercise adherence/link between level of adherence and clinical outcomes (dose-response effect)	Holden (2009) [61]
How well a patient complies with their exercise program determines how effective it will be (*)	Holden (2009) [61]

Capabilities

Description	Reference
Low health literacy (barrier)	
Poor health literacy in chronic disease management negatively influenced discussing exercise/weight management	Egerton (2018) [33]
Diagnosis can foster fear-avoidance (e.g. reduced activity) due to belief activity/exercise will cause further damage	Egerton (2018) [33]
Pessimistic about patients' abilities to make lifestyle changes to address their knee OA (not capable)	Egerton (2018) [33]
Lack of information and patient-professional discussion at point of referral may hinder uptake/retention of LMP	Law (2019) [37]

Patients were sceptical about safety and benefits of strengthening exercise for OA	Lawford (2020) [39]
Fear (patients required a lot of encouragement and reassurance)	Lawford (2020) [39]
Patients were apprehensive about managing weight by themselves	Lawford (2021) [40]
Some clients had misconceptions about OA (nothing they could do/normalising it as part of ageing)	MacKay (2018) [41]
Some clients feared participation in exercise (concerns for further degeneration)	MacKay (2018) [41]
Accepting diagnosis of OA could be particularly challenging for people with early OA	MacKay (2018) [41]
Clients' language (e.g. haven't mastered English/French)	MacKay (2018) [41]
Clients' lifestyle (e.g. coping, attitude towards pain)	MacKay (2018) [41]
Insufficient information for OA patients (e.g. not providing leaflets)	Mann (2011) [43]
Difficulty convincing patients to consider non-surgical, non-medication treatments	Miller (2020) [44]
Lack of patient self-efficacy (regarding lifestyle changes)	Miller (2020) [44]
Lack of knowledge about OA (patient barrier)	Miller (2020) [44]
Most patients demonstrated fatalism/inadequate knowledge and beliefs related to knee OA management	Poitras (2010) [47]
Ambivalent about patients' ability to lose weight (not able to succeed in making lifestyle changes)	Selten (2017) [49]
The belief that patients are not capable of losing weight	Selten (2017) [49]
Fear of falling (intrinsic barrier for patient adherence)	Teo (2020) [51]
Fear of pain (intrinsic barrier for patient adherence)	Teo (2020) [51]
Language/different cultural backgrounds (patient-related barrier)	Wallis (2020) [52]
Patients do not always believe in/may lack knowledge about effectiveness of exercise therapy	De Rooij (2020) [60]
Fear of harm (as patient-centered barrier to adherence)	Holden (2009) [61]
Limited financial resources (barrier)	
Clients' socioeconomic status (e.g. great poverty, shelter system)	MacKay (2018) [41]
Costs related to weight loss can be prohibitive for patients with limited resources (financial burdens)	Miller (2020) [44]
Other responsibilities (barrier)	
Clients' family responsibilities (e.g. busy, lot going on)	MacKay (2018) [41]
Work/other commitments precluding exercise-therapy (patient-related barrier)	Wallis (2020) [52]
High health literacy or importance of education (facilitator)	
Importance of pain education and reassurance about safety and benefits of exercise	Lawford (2020) [39]
Education contributed to buy-in to treatment (pathology, consequences, treatments)	MacKay (2020) [42]
Need for early education about OA/self-management and treatment options and opportunity to discuss these	Mann (2011) [43]
Ambivalent about patients' ability to lose weight (able)	Selten (2017) [49]
Education focused on self-management strategies	Teo (2020) [51]
Inform patients with knee OA and comorbidity better about benefits of exercise therapy	De Rooij (2020) [60]
Social support (facilitator)	
Level of support patients had from family/people close to them seemed to make a big difference	Lawford (2021) [40]
Integrating patients' social system into treatment	Rosemann (2006) [48]
Health literacy (unclear factor)	

Management strategies depended on what the person can cope with	Okwera (2019) [46]
Other responsibilities (unclear factor)	
Clients' occupation	MacKay (2018) [41]

Domain 4: Professional interactions

Collaboration

**Description**	**Reference**
Non-optimal interdisciplinary collaboration or healthcare provision (barrier)	
Cautious not to encroach on other HCPs' territory	Allison (2019) [27]
Potential for confusion about the treatment plan	Egerton (2017) [32]
Potential for issues resulting from incongruence of patient advice and information	Egerton (2017) [32]
Second professional not necessary to fulfill health coach role (part of own professional role as physical therapists)	Hinman (2016) [34]
Overlapping roles of physical therapist and coach could be source of conflict if not working from same set of goals	Hinman (2016) [34]
Necessary teamwork less likely if coach/physical therapist did not recognize/support each other's goals	Hinman (2016) [34]
Physicians who did not make timely referrals to physical therapy	MacKay (2018) [41]
Physicians' attitudes could influence clients' perceptions and level of buy-in to physical therapy	MacKay (2018) [41]
Lack of provision for patients who were not candidates for surgery (too long without help)	Mann (2011) [43]
Patients lacked proactive follow-up to support self-management	Mann (2011) [43]
Lack of coordination between leisure, social and health services	Mann (2011) [43]
Insufficient (physiotherapy) intervention when patients were seen	Mann (2011) [43]
Belief that physiotherapists did not find it rewarding/interesting to treat OA patients	Mann (2011) [43]
Criticizing the decision to centralize musculoskeletal physiotherapy service (useful to have somebody in team)	Okwera (2019) [46]
Frustrations about lack of continuity regarding team of physiotherapists within clinic	Okwera (2019) [46]
Not working closely with physiotherapists/frustrations about working relationship	Okwera (2019) [46]
Dissatisfaction about loss of coherent working since centralizing musculoskeletal physiotherapy service	Okwera (2019) [46]
Disagreement on effective exercise parameters	Poitras (2010) [47]
Disagreement on optimal design of exercise programs to increase adherence	Poitras (2010) [47]
Mistrust in interventions dieticians use to help patients' with weight reduction attempts	Selten (2017) [49]
Negative views about physical therapists who provided non-evidence-based treatments	Selten (2017) [49]
Mistrust because they observed huge differences in quality of care delivered by physical therapists	Selten (2017) [49]
Occupational therapists, podiatrists and physical therapists do not work together optimally in OA care	Selten (2017) [49]
Role of rheumatologist in knee/hip OA care perceived as unclear/limited	Selten (2017) [49]
Agreement that orthopedic surgeon's primary task is to assess whether patient is eligible for surgery	Selten (2017) [49]
Orthopedic surgeons were perceived negatively by several healthcare providers	Selten (2017) [49]
Unclear what physio offers (*)	Cottrell (2016) [53]

My colleagues in physiotherapy are not/might not be cooperative in applying the protocol in daily clinical practice (*)	De Rooij (2020) [60]
The general practitioners or other physicians are not/might not be collaborative regarding the application of the protocol in daily clinical practice (*)	De Rooij (2020) [60]
Suboptimal collaboration with general practitioners and physicians	De Rooij (2020) [60]
Referring physicians do not always believe in/may lack knowledge about effectiveness of exercise therapy	De Rooij (2020) [60]
No access to other HCPs (barrier)	
Lack of access to other healthcare providers (e.g. physicians with expertise in OA)	MacKay (2018) [41]
Good interdisciplinary collaboration or healthcare provision, or suggestions for improvement (facilitator)	
Perceived status of physical therapists within health care team and wider community	Allison (2019) [27]
Importance of clearly understanding roles and functions of service, care support team, and themselves	Egerton (2017) [32]
Importance of having confidence in (the skills of) the staff of a new service to deliver on promises	Egerton (2017) [32]
Having a personal relationship with the people providing the service/a desire to work closely with service staff	Egerton (2017) [32]
Appreciation how their participation afforded physical therapists and coaches opportunities to collaborate	Hinman (2016) [34]
Importance of teamwork in delivering the integrated intervention	Hinman (2016) [34]
Reinforcement of health messages from another clinician could be valuable	Hinman (2016) [34]
Having a separate coach freed therapists up to focus on other treatment aspects	Hinman (2016) [34]
Multidisciplinary nature of LMP (ability to utilize expertise from other professionals)	Law (2019) [37]
Importance of good working relationships	MacKay (2018) [41]
Physicians who expressed support for physical therapy/exercise and referred clients to physical therapy early	MacKay (2018) [41]
Patients would be better served by long-term condition model of care (e.g. diabetes mellitus)	Mann (2011) [43]
Patients should initiate own follow-up when needed (as better use of time/health care resources)	Mann (2011) [43]
Allow patients, after initial referral, to use direct access system to service (no need for re-referral)	Mann (2011) [43]
Utilising clinic health educator who met with patients for weight loss discussions and followed up by phone	Miller (2020) [44]
Employing a multi-pronged approach to engage patients in weight loss	Miller (2020) [44]
Overall positive experience of physiotherapy service and therapists	Okwera (2019) [46]
GPs believed PT involvement was necessary to motivate the patient and manage the exercise program	Poitras (2010) [47]
Gate keeper role for GPs could reduce patients' pressure to refer to orthopaedics/decrease performed x-rays	Rosemann (2006) [48]
Involvement of practice nurses is imaginable in the area of life style counselling and advice giving	Rosemann (2006) [48]
Dieticians are helpful for patients trying to lose weight	Selten (2017) [49]
Need for physical therapists to provide evidence-based exercises instead of non-evidence-based modalities	Selten (2017) [49]
Non-pharmacological, non-surgical OA care can and should be provided in a primary care setting	Selten (2017) [49]
GPs have coordinating role (diagnose/monitor, refer when necessary, lifestyle education, long-term coach)	Selten (2017) [49]
Physical therapists can guide patients/provide lifestyle advice (more time compared with GPs)	Selten (2017) [49]
Perceiving rheumatologists' role as valuable (giving injections, providing lifestyle/medication advice, refer)	Selten (2017) [49]
Knowledge that program was delivered by well-trained and trusted physiotherapist	Wallis (2020) [52]
Exercise for CKP is more effectively provided by physiotherapists than GPs (*)	Cottrell (2016) [53]
Obese patients with symptomatic knee OA should be assessed by a specialist multidisciplinary service, which should include an orthopaedic surgeon (*)	Hill (2018) [55]

Support for creation of regional centres where orthopedic surgeons and bariatric surgeons, with their respective teams, could assess obese patients with symptomatic knee pain (*)	Hill (2018) [55]
Obese patients with symptomatic knee OA should be referred to a specialist weight management service before orthopaedic assessment (*)	Hill (2018) [56]
Obese patients with symptomatic knee OA should be assessed by a specialist multidisciplinary service (*)	Hill (2018) [56]
Agreements with colleagues about the content of the care trajectory (*)	Hofstede (2016) [57]
Positive attitudes of colleagues about non-surgical treatments (*)	Hofstede (2016) [57]
Agreements/ deliberations with primary care (GP, physical therapist, dietician) (*)	Hofstede (2016) [57]
Physiotherapists do not/might not lack expertise in OA management (*)	Reid (2014) [59]
Working with the protocol invites me to discuss more with experts in the field of the comorbidity (*)	De Rooij (2020) [60]
Inform referrers better about benefits of exercise therapy in patients with knee OA and comorbidity	De Rooij (2020) [60]
Optimize collaboration with orthopaedic surgeons and other health care providers	De Rooij (2020) [60]
Support from colleagues	Kloek (2020) [62]
Access to other HCPs (facilitator)	
Potential benefit of increased access to OA specialists	Egerton (2017) [32]
Access to a team on-site/a network of healthcare providers they trusted	MacKay (2018) [41]
Those with access to other clinicians recommended to consult another clinician for advice on diet as needed	MacKay (2020) [42]
Care could be improved if every GP practice contained an individual who took a particular interest in OA	Mann (2011) [43]
There should be OA specialist clinicians (all relevant allied health professions) providing services in community	Mann (2011) [43]
In-house physiotherapy (as suggestion for physiotherapy service improvement)	Okwera (2019) [46]

Communication and referral

**Description**	**Reference**
Lack of communication between HCPs (barrier)	
Frustrations about lack of contact/communication involved in the referral and discharge process	Okwera (2019) [46]
Dissatisfaction about loss of communication since centralizing musculoskeletal physiotherapy service	Okwera (2019) [46]
Specialist did not take time to explain what they had examined/x-rays he had taken	Rosemann (2006) [48]
Physicians are not always collaborating in discussing medical conditions of patients	De Rooij (2020) [60]
Challenges of communication and referral procedures (barrier)	
Necessary teamwork less likely if communication processes not clearly prescribed/structure not used	Hinman (2016) [34]
Different views were expressed about the preferred medium of communication	Hinman (2016) [34]
Interdisciplinary consult with dietician could not always take place because of problems with contacting dietician	Knoop (2020) [36]
Challenges when coordinating multimodal care (including difficulties with the referral system)	Miller (2020) [44]
Frustrations about restrictive referral pathways	Okwera (2019) [46]
Referral process was convoluted and at times irrelevant	Okwera (2019) [46]
Requesting medical information about patients from specialists takes a lot of time	De Rooij (2020) [60]
Improving communication between HCPs (facilitator)	

GPs wanted to be updated on the advice given and plan made so they know what has been said to their patient	Egerton (2017) [32]
Communication needed to be collaborative, patient-centered and consistent for integrated care to be effective	Hinman (2016) [34]
Need for improving communication (quality of referrals, information at discharge)	Okwera (2019) [46]
Better communication with specialists could increase efficacy of treatment	Rosemann (2006) [48]
Receiving communication back from program physiotherapist about patient outcomes	Wallis (2020) [52]
Clarity on what the patient has done at the physical therapist (*)	Hofstede (2016) [57]
Needs regarding communication and referral procedures (facilitator)	
Need to ensure referral procedures are streamlined in order to minimize impact on their busy schedules	Egerton (2017) [32]
Need for effective, useful and timely channels of communication between the GP and the care support team	Egerton (2017) [32]
Importance of effective mechanisms to communicate	MacKay (2018) [41]
Streamlining the physiotherapy referral process (as suggestion for physiotherapy service improvement)	Okwera (2019) [46]
Straightforward, easy and quick lines of communication among different disciplines in healthcare center	Selten (2017) [49]
Simple, streamlined referral process (suggestion for promotion and referrals)	Wallis (2020) [52]

Domain 5: Incentives and resources

Time

Description	Reference
Lack of time within patient consultations (barrier)	
Time pressure (unable to individualise weight management/develop exercise plans within appointment time)	Egerton (2018) [33]
Restricted in amount of time they could allot per patient	MacKay (2018) [41]
Lack of time to give patients sufficient opportunity to discuss their condition	Mann (2011) [43]
Lifestyle counseling is huge time commitment	Miller (2020) [44]
Appointment times too short to address all of patient's issues and provide lifestyle counseling	Miller (2020) [44]
Time required to teach PCST skills to patients	Nielsen (2014) [45]
Weight reduction advice takes too much time in a consultation	Selten (2017) [49]
Insufficient time in consultations (*)	Cottrell (2016) [53]
Time constraints prevent GPs from providing advice on individual exercises for CKP (*)	Cottrell (2016) [53]
Limited time to review individual patients reduced opportunities to facilitate behavior change	Holden (2009) [61]
Large caseloads and pressure of waiting lists reduced the number of treatment sessions provided	Holden (2009) [61]
Limited opportunity to provide follow-up sessions after discharge	Holden (2009) [61]
Lack of time due to other demands (or not further specified) (barrier)	
The addition of a care support team may increase paperwork	Egerton (2017) [32]
Lack of time to monitor attendance/provide support was compounded by increasing administrative demands	Law (2019) [37]
Wait lists as a burden	MacKay (2018) [41]
Lack of time	Rosemann (2006) [48]

Less satisfied about time needed to get used to e-Exercise during high work pressure/administrative burden	Kloek (2020) [62]
Perceiving web-based application as an additional burden	Kloek (2020) [62]
Busy work schedules and administrative burden hindered testing/using e-Exercise in their practice	Kloek (2020) [62]
Adequate duration of patient consultations (facilitator)	
Importance of longer consultations	Egerton (2018) [33]
Having adequate time to spend with clients	MacKay (2018) [41]
Adequate duration of specific interventions or protocols (facilitator)	
Idea of having some of the burden of managing this patient group (e.g. time) taken away appealing	Egerton (2017) [32]
Incorporating selected PCST components on as-needed basis most practical way within current environment	Nielsen (2014) [45]
Working according to the protocol is not/might not be too time-consuming (*)	De Rooij (2020) [60]
Perceiving web-based application as time-saving	Kloek (2020) [62]
I do not/might not have insufficient time available to get familiar with e-Exercise and to use the web-application (*)	Kloek (2020) [62]

Financial resources

**Description**	**Reference**
Limited financial resources within organization (barrier)	
Concern about capacity to recover costs of incorporating CBT into practice	Nielsen (2014) [45]
Treatments (e.g. physiotherapy) prescribed less frequently due to decreasing financial resources	Rosemann (2006) [48]
Financial reward for implementing LIs (facilitator)	
Financial incentivisation	Egerton (2017) [32]
Payment system has to be changed to upgrade conservative treatments and conversation with patient	Rosemann (2006) [48]
Interventions performed by practice nurses have to be reinsured sufficiently	Rosemann (2006) [48]
Working according to the protocol should be financially rewarded (*)	De Rooij (2020) [60]

Information resources

**Description**	**Reference**
Lack of information resources (barrier)	
Absence of clear guidelines for weight loss	Allison (2019) [27]
Lack of information about scheme hindered referral	Law (2019) [37]
Lack of resources for face-to-face patient education and patient reference	Miller (2020) [44]
Lack of information about self-help groups/offers on community level	Rosemann (2006) [48]
Missing information about offers e.g. in the community	Rosemann (2006) [48]
Challenges in accessing information resources (barrier)	
Challenges in accessing scientific papers	MacKay (2018) [41]
Difficulty finding high quality, patient-friendly OA educational materials	Miller (2020) [44]

Frustration that material found on Internet or provided by friends/family was frequently inaccurate	Miller (2020) [44]
Cannot access necessary resources (*)	Cottrell (2016) [53]
Availability of information resources (facilitator)	
Clear preference for concrete guidelines or tools for engaging in weight management	Allison (2019) [27]
Recommending informational materials for patients (to mitigate delays in OA care)	Miller (2020) [44]
Standardised flowsheet on OA management (as guide for providers/tool for patient discussions)	Miller (2020) [44]
Specific information about program (suggestion for promotion and referrals)	Wallis (2020) [52]
Clear referral criteria/guideline (*)	Hofstede (2016) [57]
Access to information resources (facilitator)	
Having access to customizable, printable patient resources	Egerton (2018) [33]
Access to current evidence	MacKay (2018) [41]
Professional networks/community of practice as mechanism to facilitate sharing of information	MacKay (2018) [41]
Integrating scientific evidence from studies into their approach to management	MacKay (2020) [42]
Including links on websites of partners (suggestion for promotion and referrals)	Wallis (2020) [52]

Facilities

**Description**	**Reference**
Negative attitude toward information technology (barrier)	
Sceptical about benefit of clinical practice information technology	Egerton (2018) [33]
Potential use of information technology (facilitator)	
Changes to clinical practice information technology (e.g. prompts into clinic software)	Egerton (2018) [33]
Having patient resources embedded within current practice software or routines	Egerton (2018) [33]
Electronic reminders for physicians on how to locate OA treatment information and resources	Miller (2020) [44]
Benefits of working in health centers (facilitator)	
Collaboration among multiple disciplines could be facilitated by working in a health center	Selten (2017) [49]

Domain 6: Capacity for organizational change

Professional paradigm

**Description**	**Reference**
Adequate professional paradigm or suggestions for expansion (facilitator)	
Nature of the physical therapy paradigm (in relation to weight management)	Allison (2019) [27]
Physical therapy scope of practice was adequate to manage clients with perceived early knee OA	MacKay (2018) [41]
Suggestion that it would be useful to expand scope of practice to include ordering diagnostic imaging	MacKay (2018) [41]
Value of increasing profession's explicit understanding/use of PCST skills (practice model may be required)	Nielsen (2014) [45]

Value of incorporating aspects of PCST mind-set into professional training (entry-level vs. postgraduate level)	Nielsen (2014) [45]

Monitoring

**Description**	**Reference**
Audit (facilitator)	
Peer review/audit of professional association (*)	Hofstede (2016) [57]

Support within the organization

**Description**	**Reference**
Management not supportive (barrier)	
The management of my practice is not/might not be collaborative regarding the application of the protocol in daily clinical practice (*)	De Rooij (2020) [60]

Domain 7: Social, political, and legal factors

Healthcare system

**Description**	**Reference**
Restrictions due to health insurance (barrier)	
Lack of funding prevented clients from accessing services/seeking help/getting full course of treatment	MacKay (2018) [41]
Treatments (e.g. physiotherapy) prescribed less frequently due to increasing restrictions by health insurances	Rosemann (2006) [48]
The number of treatments that the patient receives from their insurance company is a barrier in using the protocol (*)	De Rooij (2020) [60]
Number of treatment sessions patients receive from insurance companies restricted application of the strategy	De Rooij (2020) [60]
Benefits of good health insurance (facilitator)	
Patients who are well insured have improved access to services (e.g. physical therapy)	Miller (2020) [44]
Positivity toward private sector (patients will get seen a lot quicker)	Okwera (2019) [46]
Private healthcare supplementation (as suggestion for physiotherapy service improvement)	Okwera (2019) [46]
In complex patients insurance companies should reimburse more treatment sessions	De Rooij (2020) [60]
Government subsidies (facilitator)	
Government-subsidised allied health visits to facilitate utilisation of services that support exercise/weight loss	Egerton (2018) [33]

Domain 8: Patient and HCP interactions

Therapeutic alliance

**Description**	**Reference**

Potential negative influence of implementing LIs to relationship (barrier)	
Feelings of guilt when referring to LMP (dooming patients to a longer wait for surgery)	Law (2019) [37]
Importance of communication and relationship (facilitator)	
Strong therapeutic relationship with patients	Lawford (2020) [39]
Having positive attitude/being encouraging of small changes/being hopeful about OA management	MacKay (2020) [42]
More openly address psychological complaints of patients	Rosemann (2006) [48]
Good communication with patient may help in delaying surgery	Selten (2017) [49]
Importance of having trust	Selten (2017) [49]

Lifestyle as conversation topic

**Description**	**Reference**
Challenges of discussing weight (barrier)	
Perceived impact of own weight during weight discussions (not being overweight)	Allison (2019) [27]
Apparent discomfort with having conversations about weight	Allison (2019) [27]
Concern about how weight conversations might threaten patient rapport	Allison (2019) [27]
Weight loss is sensitive topic (afraid of upsetting their patients results in temptation to avoid discussion)	Egerton (2018) [33]
Weight was touchy/sensitive subject to discuss	MacKay (2020) [42]
Difficulties in communicating with patients about being overweight	Selten (2017) [49]
Viewing weight as sensitive subject/feeling uncomfortable discussing it	Tang (2020) [50]
Factors that could ease the way to discussing weight (facilitator)	
Perceived impact of own weight during weight discussions (being overweight)	Allison (2019) [27]
Feeling comfortable discussing role of physical activity in maintaining weight control	MacKay (2020) [42]
Developing rapport with people made it easier to discuss weight management	MacKay (2020) [42]
Reframe discussions around exercise and weight loss (e.g. not blaming/discouraging people)	Miller (2020) [44]
Relationship with patients, developed through numerous sessions, facilitated influence for lifestyle modifications	Poitras (2010) [47]
Having a relationship with patient built on mutual trust/respect would ease way to discussing weight reduction	Selten (2017) [49]

Domain 9: Disease factors

Image

**Description**	**Reference**
OA seen as low priority (barrier)	
Assigning low priority to OA as disease	Christiansen (2020) [29]
Concern about providing this service for a condition perceived as low priority	Egerton (2017) [32]
OA was not given enough attention, symptoms were often dismissed/minimized in health care	Mann (2011) [43]

Medical professionals saw OA as low priority with respect to managing their workload	Okwera (2019) [46]
Knee OA more often diagnosed as an unanticipated comorbidity (rarely primary reason for consultation)	Poitras (2010) [47]
Not enough emphasis put on primary prevention of knee OA	Poitras (2010) [47]
Knee OA management seen as unchallenging routine	Poitras (2010) [47]
Belief that nobody is willing to change lifestyle due to OA, disease has to be a lot worse	Rosemann (2006) [48]
OA seen as untreatable and local condition (wear-and-tear) (barrier)	
Describing OA as simply a problem of cartilage degeneration/joint space narrowing (on x-ray)/wear and tear	Egerton (2018) [33]
Belief that symptoms will progress, and that surgery is inevitable	Egerton (2018) [33]
Assumption that patients would have negative connotations associated with the label knee OA	Egerton (2018) [33]
No effective treatment options	Miller (2020) [44]
Incurable nature and negative prognosis of OA	Okwera (2019) [46]
Knee OA seen as uninteresting health problem on which they had limited impact and could not cure	Poitras (2010) [47]
Knee OA was perceived as a degenerative (wear and tear)	Teo (2020) [51]
Using negative language to describe OA (wear-and-tear/joint damage/bone-on-bone/degenerative condition)	Wallis (2020) [52]
Biomedical perspective on knee OA, attributing signs and symptoms to local knee pathology or wear and tear	Holden (2009) [61]
OA seen as chronic degenerative condition that would progressively worsen over time (only cure being surgery)	Holden (2009) [61]
Optimistic views toward OA (facilitator)	
Belief that knee OA is condition that can be successfully managed	Egerton (2018) [33]
Importance of conveying to patients that diagnosis is not all negative/delivering a relatively positive prognosis	Egerton (2018) [33]
Knee OA seen as technically challenging condition	Poitras (2010) [47]
