# Supplementary material for: Barriers and facilitators perceived by healthcare professionals for implementing lifestyle interventions in patients with osteoarthritis: a scoping review
Source: BMJ Open. 2022 Feb 1;12(2):e056831. doi: 10.1136/bmjopen-2021-056831 (PMC8808449; doi:10.1136/bmjopen-2021-056831)
Supplement: Supplementary data [file bmjopen-2021-056831supp004.pdf]

Supplemental File 4. Full overview of all extracted factors per article

Explanation

- In the tables below, all extracted factors per included article are presented. The number in brackets ('[…]') displayed after each article corresponds to the reference number used in the main text of the manuscript.
- Column "Number": the capital letter and color used refer to barriers (B/red), facilitators (F/green) or unclear factors (U/orange).
- Column "Description": (*) at the end of the description indicates that the factor is derived from a close-ended question or attitude statement.
- Column "Subcategory (domain)": the relevant subcategory is displayed first, followed by the number of the domain to which this subcategory belongs. The domain numbers refer to the domains as described in the main text of the manuscript: (1) intervention factors; (2) individual HCP factors; (3) patient factors; (4) professional interactions; (5) incentives and resources; (6) capacity for organizational change; (7) social, political, and legal factors; (8) patient and HCP interactions; (9) disease factors.
- Abbreviations: ACSM: American College of Sports Medicine; BMI: body mass index; CBT: cognitive behavioral therapy; CKP: chronic knee pain; CPG: clinical practice guideline; GP: general practitioner; HCP: healthcare professional; LI: lifestyle intervention; LMP: Lifestyle Management Programme; NSAID: non-steroidal anti-inflammatory drug; NWBE: non-weight bearing quadriceps strengthening exercise; OA: osteoarthritis; PCST: pain coping skills training; PT: physiotherapist; RCT: randomized controlled trial; TKA: total knee arthroplasty; TJA: total joint arthroplasty; WBE: weight bearing functional exercise.

Allison (2019) [27]

Number	Description	Subcategory (domain)
B1	Cautious not to encroach on other HCPs' territory	Non-optimal interdisciplinary collaboration or healthcare provision (4)
B2	Lack of knowledge around appropriate interventions for weight loss	Lack of knowledge or skills around LIs or promoting behavioral change (2)
B3	Uncertainty about how to enact their understanding of relationship between weight and knee OA	Lack of knowledge or skills around LIs or promoting behavioral change (2)
B4	Absence of clear guidelines for weight loss	Lack of information resources (5)
B5	Perceived impact of own weight during weight discussions (not being overweight)	Challenges of discussing weight (8)
B6	Apparent discomfort with having conversations about weight	Challenges of discussing weight (8)
B7	Concern about how weight conversations might threaten patient rapport	Challenges of discussing weight (8)
F1	Nature of the physical therapy paradigm (in relation to weight management)	Adequate professional paradigm or suggestions for expansion (6)
F2	Perceived status of physical therapists within health care team and wider community	Good interdisciplinary collaboration or healthcare provision, or suggestions for improvement (4)
F3	Perceived professional responsibility to adhere to evidence-based guideline	Positive attitude toward guidelines or protocols (2)
F4	Clear preference for concrete guidelines or tools for engaging in weight management	Availability of information resources (5)

F5	Perceived impact of own weight during weight discussions (being overweight)	Factors that could ease the way to discussing weight (8)

Bossen (2016) [28]

Number	Description	Subcategory (domain)
B1	Lack of financial incentive if blended intervention substitutes conventional visits (reduced venues per patient)	Other challenges for HCPs regarding feasibility of telehealth (1)
B2	Most of the patients prefer traditional face-to-face treatments	Patient-related challenges regarding feasibility of telehealth (1)
B3	Most of the patients did not meet study inclusion criteria	LIs are unavailable or inaccessible (1)
B4	e-Exercise must be adapted for suitable integration into practice (e.g. no insight into modules patients receive)	Challenges for HCPs during delivery of LIs (1)
F1	24/7 availability of information and exercises	Patient-related benefits regarding feasibility of telehealth (1)
F2	Possibility to extend physical therapy treatment in patient's home environment	Benefits of telehealth in terms of effectiveness (1)
F3	Potential to enhance the adherence of home exercises	Benefits of telehealth in terms of effectiveness (1)
F4	Positive feedback regarding the content of e-Exercise	Positive experiences with or suggestions to improve the content or structure of LIs (1)

Christiansen (2020) [29]

Number	Description	Subcategory (domain)
B1	Assigning low priority to OA as disease	OA seen as low priority (9)
B2	Assigning low priority to exercise as treatment	Negative attitude toward LIs (2)
B3	Difficulty with managing multiple conditions/tendency to prioritize other conditions over OA	Negative impact of comorbidities (3)
B4	Not certain that exercise works	LIs have little or no effect on OA (1)
B5	Referring patients to other health care providers and for other treatments rather than recommending exercise	Perception of own role potentially impeding prescription or follow-up of LIs (2)
B6	Limited knowledge of exercise prescription (uncertainty of what exercise to recommend/how much)	Lack of knowledge or skills around LIs or promoting behavioral change (2)
B7	Referring patients to those with specialized knowledge rather than treating themselves (outside scope of practice)	Perception of own role potentially impeding prescription or follow-up of LIs (2)
B8	Not received sufficient training on exercise/lack of education	Lack of knowledge or skills around LIs or promoting behavioral change (2)
B9	Patients' lack of motivation to exercise/patients want passive treatment approach or quick fix	Negative attitude toward LIs (3)

Davis (2018) [30]

Number	Description	Subcategory (domain)

B1	Class required intense supervision, which was difficult to provide when most participants were new	Challenges for HCPs during delivery of LIs (1)
B2	Challenges of supervision when space did not allow clear line of sight	Challenges for HCPs during delivery of LIs (1)
F1	Enthusiastic about the program and described the results (e.g. it was empowering)	LIs have positive mental effects (1)
F2	Initial classes needed to be small with rolling recruitment very beneficial	Ease for HCPs during delivery of LIs (1)
F3	First education session was critical to reducing the participant's anxiety related to exercising	Positive experiences with or suggestions to improve the content or structure of LIs (1)
F4	Importance of empowering the patients rather than 'pushing' them, achieved by 'giving choices'	Positive experiences with or suggestions to improve the content or structure of LIs (1)
F5	Exercise progression was most effective when the participant requested progression	Importance of high patient adherence or engagement for effectiveness of LIs (3)

De Rooij (2014) [31]

Number	Description	Subcategory (domain)
F1	Protocols offered guidance in setting up treatment/making clinical decisions/adapting treatment to comorbidity	Available resources might improve knowledge and decision-making (2)
F2	List of restrictions for exercise therapy was conveniently arranged checklist for diagnostic and treatment phases	Ease for HCPs during delivery of LIs (1)
F3	List of restrictions was helpful in process of clinical decision making	Available resources might improve knowledge and decision-making (2)
F4	Suggestion to increase feasibility by reducing the protocols to three main protocols	Ease for HCPs during delivery of LIs (1)
F5	Less afraid to increase training intensity (preventing adverse events by tailoring programs to individual's capacity)	Research environment or protocols provide a safety net (1)

Egerton (2017) [32]

Number	Description	Subcategory (domain)
B1	Concern that uptake would be negatively impacted if patients were required to pay	Costs of LIs to patients (1)
B2	Concern for overcomplicated system when service is not compatible/complementary with existing initiatives	LIs are not feasible or sustainable (1)
B3	Not seeing need (already adequate skills/resources to support OA patient self-management and lifestyle change)	LIs are not feasible or sustainable (1)
B4	Concern about providing this service for a condition perceived as low priority	OA seen as low priority (9)
B5	Not seeing need (advice already given at their practice would be unhelpfully repeated)	LIs are not feasible or sustainable (1)
B6	Remote (telephone) delivery is not as good as face-to-face particularly in relation to exercise advice	Disadvantages of telehealth in terms of effectiveness (1)
B7	Advice to exercise and lose weight does not work	LIs have little or no effect on OA (1)

B8	Hesitancy to embrace an unfamiliar new service	Other challenges for HCPs regarding feasibility of telehealth (1)
B9	Concern regarding long-term service sustainability	LIs are not feasible or sustainable (1)
B10	Concerns about security of patient data and information confidentiality during the referral process	Telehealth is not safe for patients or patient/data privacy (1)
B11	Job satisfaction may be diminished when handing over care of their patients to third party with no involvement	Negative consequences for own role when referring patients to LIs (2)
B12	The addition of a care support team may add complexities to management	LIs are not feasible or sustainable (1)
B13	The addition of a care support team may increase paperwork	Lack of time due to other demands (or not further specified) (5)
B14	The addition of a care support team may lead them feeling disconnected with their patient's care	Negative consequences for own role when referring patients to LIs (2)
B15	Potential for confusion about the treatment plan	Non-optimal interdisciplinary collaboration or healthcare provision (4)
B16	Potential for issues resulting from incongruence of patient advice and information	Non-optimal interdisciplinary collaboration or healthcare provision (4)
B17	Concerned the service would not be able to provide individualized management for a very diverse population	Insufficient ability to provide personalized treatment within LIs (1)
B18	Hearing and cognitive difficulties as barriers for some patients to being able to interact with the service	Patient-related challenges regarding feasibility of telehealth (1)
B19	Level of disease severity (i.e. whether people with very mild or very severe joint disease would benefit)	Severity of disease and symptoms (3)
B20	Inability of a remote service to provide locally relevant information	Disadvantages of telehealth in terms of effectiveness (1)
B21	Skepticism about whether many patients would embrace such a model (i.e. because of remote-delivery aspect)	Patient-related challenges regarding feasibility of telehealth (1)
F1	More likely to engage with the care support team if it enabled more affordable/accessible allied health	LIs are available or accessible, or suggestions for improvement (1)
F2	Need to ensure referral procedures are streamlined in order to minimize impact on their busy schedules	Needs regarding communication and referral procedures (4)
F3	Need for effective, useful and timely channels of communication between the GP and the care support team	Needs regarding communication and referral procedures (4)
F4	GPs wanted to be updated on the advice given and plan made so they know what has been said to their patient	Improving communication between HCPs (4)
F5	Need for clarity about how the new service would integrate with existing schemes and payment structures	LIs are feasible or sustainable (1)
F6	Seeing need (advice/recommendations may need to be reinforced/provided over several health care episodes)	LIs are feasible or sustainable (1)
F7	Seeing need (extra time and encouragement for the patient would result in better outcomes)	LIs are feasible or sustainable (1)
F8	Potential benefit of increased access to OA specialists	Access to other HCPs (4)
F9	Importance of clearly understanding roles and functions of service, care support team, and themselves	Good interdisciplinary collaboration or healthcare provision, or suggestions for improvement (4)

F10	Importance of broad acceptance (patients/doctors/health service funders) if new service is to continue long term	LIs are feasible or sustainable (1)
F11	Importance of having confidence in (the skills of) the staff of a new service to deliver on promises	Good interdisciplinary collaboration or healthcare provision, or suggestions for improvement (4)
F12	Having a personal relationship with the people providing the service/a desire to work closely with service staff	Good interdisciplinary collaboration or healthcare provision, or suggestions for improvement (4)
F13	Idea of having some of the burden of managing this patient group (e.g. time) taken away appealing	Adequate duration of specific interventions or protocols (5)
F14	Some appeal for a lessening of their own responsibility in terms of managing this condition	Positive consequences for own role when referring patients to LIs (2)
F15	Service could increase access to support for rural patients	Patient-related benefits regarding feasibility of telehealth (1)
F16	Financial incentivisation	Financial reward for implementing LIs (5)

Egerton (2018) [33]

Number	Description	Subcategory (domain)
B1	Describing OA as simply a problem of cartilage degeneration/joint space narrowing (on x-ray)/wear and tear	OA seen as untreatable and local condition (wear-and-tear) (9)
B2	Belief that symptoms will progress, and that surgery is inevitable	OA seen as untreatable and local condition (wear-and-tear) (9)
B3	Knowledge of exercise and weight-loss treatments is sometimes inaccurate or inadequate	Lack of knowledge or skills around LIs or promoting behavioral change (2)
B4	Dubious about effect of exercise and weight-management advice on reducing symptoms	LIs have little or no effect on OA (1)
B5	Reduced confidence with providing suitable exercise and weight loss advice	Lack of knowledge or skills around LIs or promoting behavioral change (2)
B6	Lack of skills in promoting readiness and motivation for lifestyle treatments	Lack of knowledge or skills around LIs or promoting behavioral change (2)
B7	Time pressure (unable to individualise weight management/develop exercise plans within appointment time)	Lack of time within patient consultations (5)
B8	Concerns regarding financial cost to patients when considering referral to other services	Costs of LIs to patients (1)
B9	Lack of availability of support services (e.g. community-based rehabilitation programs) in remote locations	LIs are unavailable or inaccessible (1)
B10	Long waiting lists for support services (e.g. community-based rehabilitation programs)	LIs are unavailable or inaccessible (1)
B11	Sceptical about benefit of clinical practice information technology	Negative attitude toward information technology (5)
B12	The issue is not a lack of suitable patient resources but awareness of them	Lack of knowledge or skills around specific resources (2)
B13	Poor health literacy in chronic disease management negatively influenced discussing exercise/weight management	Low health literacy (3)
B14	Patients often have own ideas on management (problematic if primarily passive treatments)	Negative attitude toward LIs (3)

B15	Shifting patients' mind-sets to active participation/making lifestyle changes was challenging/time consuming	Low patient adherence or engagement (3)
B16	Paternalistic approach to care (low level of engagement in providing exercise and weight management advice)	Perception of own role potentially impeding prescription or follow-up of LIs (2)
B17	Assumption that patients would have negative connotations associated with the label knee OA	OA seen as untreatable and local condition (wear-and-tear) (9)
B18	Diagnosis can foster fear-avoidance (e.g. reduced activity) due to belief activity/exercise will cause further damage	Low health literacy (3)
B19	Pessimistic about patients' abilities to make lifestyle changes to address their knee OA (not capable)	Low health literacy (3)
B20	Weight loss is sensitive topic (afraid of upsetting their patients results in temptation to avoid discussion)	Challenges of discussing weight (8)
F1	Need for tailored GP education to improve confidence	Having or improving knowledge or skills around OA care in general (2)
F2	Importance of having highly effective communication skills	Having or improving knowledge or skills around LIs or promoting behavioral change (2)
F3	Lifestyle treatments benefited other chronic conditions	LIs have positive effects on general health (1)
F4	Importance of longer consultations	Adequate duration of patient consultations (5)
F5	Government-subsidised allied health visits to facilitate utilisation of services that support exercise/weight loss	Government subsidies (7)
F6	Changes to clinical practice information technology (e.g. prompts into clinic software)	Potential use of information technology (5)
F7	Having access to customizable, printable patient resources	Access to information resources (5)
F8	Having patient resources embedded within current practice software or routines	Potential use of information technology (5)
F9	Patient-centred approach (high level of engagement in providing exercise and weight management advice)	Perception of own role potentially stimulating prescription or follow-up of LIs (2)
F10	Belief that knee OA is condition that can be successfully managed	Optimistic views toward OA (9)
F11	Importance of conveying to patients that diagnosis is not all negative/delivering a relatively positive prognosis	Optimistic views toward OA (9)
F12	Acknowledging that weight loss (when someone is overweight) is important	Positive attitude toward LIs (2)

Hinman (2016) [34]

Number	Description	Subcategory (domain)
B1	Structure/timing of exercise program restricted capacity to modify exercises/provide adequate follow-up	Non-optimal content or structure of LIs (1)
B2	Lack of face-to-face contact difficult/hampered ability to establish normal rapport/build effective relationships	Negative aspects regarding communication and relationship using telehealth (1)
B3	Second professional not necessary to fulfill health coach role (part of own professional	Non-optimal interdisciplinary collaboration or healthcare provision (4)

	role as physical therapists)	
B4	Overlapping roles of physical therapist and coach could be source of conflict if not working from same set of goals	Non-optimal interdisciplinary collaboration or healthcare provision (4)
B5	Reluctance from patients to talk about physical activity (physical therapist's role, not the coach's role)	Negative attitude toward LIs (3)
B6	Necessary teamwork less likely if communication processes not clearly prescribed/structure not used	Challenges of communication and referral procedures (4)
B7	Necessary teamwork less likely if coach/physical therapist did not recognize/support each other's goals	Non-optimal interdisciplinary collaboration or healthcare provision (4)
B8	Different views were expressed about the preferred medium of communication	Challenges of communication and referral procedures (4)
F1	Appreciation how their participation afforded physical therapists and coaches opportunities to collaborate	Good interdisciplinary collaboration or healthcare provision, or suggestions for improvement (4)
F2	Positive impact on patients of personalized attention from coach and from advice/education they provided	LIs have positive effects (not further specified) (1)
F3	Value of monitoring/encouraging patients to develop own understanding of links between exercise/pain	Having or improving knowledge or skills around LIs or promoting behavioral change (2)
F4	Positive impact of information, education, and structured monitoring on patients' adherence to exercise	High patient adherence or engagement (3)
F5	Requirements of treatment protocol freed therapists to notice and reflect on impact of the interventions	Ease for HCPs during delivery of LIs (1)
F6	Positive comments about the exercise regimen	Positive experiences with or suggestions to improve the content or structure of LIs (1)
F7	Structured protocol allowed to experience different OA treatment regimen/observe and learn from impact	Ease for HCPs during delivery of LIs (1)
F8	Structure provided by protocol/structure of exercises (how patients included them into daily routine)	Positive experiences with or suggestions to improve the content or structure of LIs (1)
F9	Importance of teamwork in delivering the integrated intervention	Good interdisciplinary collaboration or healthcare provision, or suggestions for improvement (4)
F10	Reinforcement of health messages from another clinician could be valuable	Good interdisciplinary collaboration or healthcare provision, or suggestions for improvement (4)
F11	Having a separate coach freed therapists up to focus on other treatment aspects	Good interdisciplinary collaboration or healthcare provision, or suggestions for improvement (4)
F12	Communication needed to be collaborative, patient-centered and consistent for integrated care to be effective	Improving communication between HCPs (4)

Hinman (2017) [35]

**Number**	**Description**	**Subcategory (domain)**

B1	Occasional technical difficulties (e.g. poor internet connection) could disrupt the flow of the consultation	Negative aspects regarding communication and relationship using telehealth (1)
B2	Patient flexibility could come at a cost to the therapist sometimes (allowed patients to reschedule last minute)	Other challenges for HCPs regarding feasibility of telehealth (1)
B3	Forced to modify usual habits/rely more on information shared by patients (instead of own physical assessment)	Challenges for HCPs regarding lack of physical/visual contact (1)
B4	Some discomfort without hands-on assessment (no palpation of patient's knee/hands-on facilitation of exercises)	Challenges for HCPs regarding lack of physical/visual contact (1)
B5	Skype consultations more suitable as adjunctive to usual in-clinic care (initial assessment in person preferred)	Other challenges for HCPs regarding feasibility of telehealth (1)
F1	Ease of using Skype for consultations	Positive attitude or needs of HCPs regarding feasibility of telehealth (1)
F2	Quality of technology suitable for providing instructions/prescribing exercises/receiving instantaneous feedback	Positive attitude or needs of HCPs regarding feasibility of telehealth (1)
F3	Skype-delivered care convenient for patients (time efficiency/flexibility/access)	Patient-related benefits regarding feasibility of telehealth (1)
F4	Empowering effect of home environment on patient adherence with exercise program	Benefits of telehealth in terms of effectiveness (1)
F5	Home environment facilitated correct and safe exercise techniques	Telehealth is safe for patients or patient/data privacy (1)
F6	Using Skype distilled focus to most important and effective treatment elements to facilitate self-management	Benefits of telehealth in terms of effectiveness (1)
F7	Patients more relaxed in home environment/more receptive to the information the therapists provided	Benefits of telehealth in terms of effectiveness (1)
F8	Patients responded favorably to the exercises prescribed despite lack of hands-on assessment	Lack of physical/visual contact not a major issue for HCPs (1)
F9	Safety net provided by research environment (e.g. patients were previously screened for comorbidities/red flags)	Research environment or protocols provide a safety net (1)
F10	Hands-off approach was physically less demanding compared to usual care/contributed to sense of satisfaction	Lack of physical/visual contact not a major issue for HCPs (1)
F11	Functional improvements experienced by patients	LIs have positive effects on affected joint(s) (1)
F12	Functional improvements were observable using Skype	Lack of physical/visual contact not a major issue for HCPs (1)
F13	Greater confidence to exercise among patients	LIs have positive mental effects (1)

Knoop (2020) [36]

Number	Description	Subcategory (domain)
B1	Maximum number of four sessions was considered too low in many patients	Non-optimal content or structure of LIs (1)
B2	Behavioral approach in exercise therapy and advice to visit GP were considered unnecessary for most patients	Non-optimal content or structure of LIs (1)
B3	Interdisciplinary consult with dietician could not always take place because of problems with contacting dietician	Challenges of communication and referral procedures (4)

B4	Interdisciplinary consult with dietician could not always take place because patients refused to visit dietician	Negative attitude toward LIs (3)
F1	Model of stratified care easy to apply and having added value for daily practice	LIs are feasible or sustainable (1)
F2	Appreciation of applicability of treatment protocols	LIs are feasible or sustainable (1)

Law (2019) [37]

Number	Description	Subcategory (domain)
B1	Patients' ideas about whether they wanted surgery influenced making referrals to the LMP	Positive attitude toward TJA (3)
B2	How well they felt the individual patient would engage with programme influenced making referrals to the LMP	Low patient adherence or engagement (3)
B3	Lack of information about scheme hindered referral	Lack of information resources (5)
B4	Feelings of guilt when referring to LMP (dooming patients to a longer wait for surgery)	Potential negative influence of implementing LIs to relationship (8)
B5	Lack of information and patient-professional discussion at point of referral may hinder uptake/retention of LMP	Low health literacy (3)
B6	Lack of time to monitor attendance/provide support was compounded by increasing administrative demands	Lack of time due to other demands (or not further specified) (5)
F1	Multidisciplinary nature of LMP (whole-person, intensive and functional approach)	Positive experiences with or suggestions to improve the content or structure of LIs (1)
F2	Multidisciplinary nature of LMP (ability to utilize expertise from other professionals)	Good interdisciplinary collaboration or healthcare provision, or suggestions for improvement (4)
F3	Suggestion to relist patients completing the programme further up the waiting list (for surgery)	Make use of patients' preference for TJA within LIs (3)
F4	LMP would benefit from extension of inclusion criteria (patients with less severe OA and lower BMI)	LIs are available or accessible, or suggestions for improvement (1)
F5	Emphasising health benefits of programme	LIs have positive effects (not further specified) (1)
F6	Reminding patients of opportunity to self-manage	LIs have positive mental effects (1)
F7	Using bargaining techniques centering on implications of LMP for replacement surgery (put patient on the list)	Make use of patients' preference for TJA within LIs (3)
F8	Standardization was viewed as important for monitoring and evaluation purposes	Positive experiences with or suggestions to improve the content or structure of LIs (1)
F9	Flexibility was valuable when tackling local participation challenges	Ability and importance of providing personalized treatment within LIs (1)
F10	Helpful social impact of group-based programme	Positive experiences with or suggestions to improve the content or structure of LIs (1)
F11	Further and ongoing evaluation of the LMP would help to address current challenges	LIs are feasible or sustainable (1)

U1	Autonomy affects referral considerations	Autonomy (2)

Lawford (2019) [38]

Number	Description	Subcategory (domain)
B1	Telephone not viewed as primary mode of providing care (only for follow-up)	Other challenges for HCPs regarding feasibility of telehealth (1)
B2	Assessment of patients could be difficult when consulting via telephone (inability to observe)	Challenges for HCPs regarding lack of physical/visual contact (1)
B3	Relationships with patients might be adversely impacted/could be difficult to develop rapport	Negative aspects regarding communication and relationship using telehealth (1)
B4	Difficulties communicating might be experienced when consulting via telephone	Negative aspects regarding communication and relationship using telehealth (1)
B5	Lack of visual/physical contact would limit strategies available when teaching patients an exercise program	Challenges for HCPs regarding lack of physical/visual contact (1)
B6	Some difficulty scheduling telephone consultations during usual day of face-to-face consultations	Other challenges for HCPs regarding feasibility of telehealth (1)
F1	Telephone-delivered care would be convenient for patients	Patient-related benefits regarding feasibility of telehealth (1)
F2	Patients could be more comfortable talking about condition/engaging in exercise program from own home	Benefits of telehealth in terms of effectiveness (1)
F3	Telephone-delivered care could reduce patient costs associated with accessing physiotherapy services	Patient-related benefits regarding feasibility of telehealth (1)
F4	Telephone-delivered care could provide increased opportunities to educate patients about OA	Benefits of telehealth in terms of effectiveness (1)
F5	Telephone-delivered care could allow wider variety of patients to access physiotherapy	Patient-related benefits regarding feasibility of telehealth (1)
F6	More effective communication skills would be needed to consult via telephone	Positive attitude or needs of HCPs regarding feasibility of telehealth (1)
F7	It would be necessary to provide patients with pictures or videos of each exercise when consulting via telephone	Positive attitude or needs of HCPs regarding feasibility of telehealth (1)
F8	Experiences providing telephone-delivered care exceeded expectations, resulting in new enthusiasm	Positive attitude or needs of HCPs regarding feasibility of telehealth (1)
F9	Lack of physical and visual contact less of an issue than anticipated	Lack of physical/visual contact not a major issue for HCPs (1)
F10	Developed a strong rapport with patients over the telephone	Positive aspects regarding communication and relationship using telehealth (1)
F11	Patient adherence to telephone-delivered exercise program was high	Benefits of telehealth in terms of effectiveness (1)
F12	Consulting via telephone forced to focus on effective conversations with patients (more personal level)	Positive aspects regarding communication and relationship using telehealth (1)
F13	Noticeable shift in patients' expectations of physiotherapy care (more willing to self-manage their condition)	Patient-related benefits regarding feasibility of telehealth (1)
F14	Improvements in patient pain and function	LIs have positive effects on affected joint(s) (1)
F15	Increased confidence to self-manage	LIs have positive mental effects (1)

F16	Telephone-delivered care was convenient for patients	Patient-related benefits regarding feasibility of telehealth (1)
F17	Able to work around the lack of visual contact (erring on the side of caution)	Lack of physical/visual contact not a major issue for HCPs (1)
F18	Written materials provided to patients helped to prescribe exercises effectively	Positive attitude or needs of HCPs regarding feasibility of telehealth (1)
F19	There was a safety net in place with the trial (each patient had been screened)	Research environment or protocols provide a safety net (1)
F20	Training in communication and/or health coaching important to effectively deliver care over telephone	Positive attitude or needs of HCPs regarding feasibility of telehealth (1)

Lawford (2020) [39]

Number	Description	Subcategory (domain)
B1	Patients were sceptical about safety and benefits of strengthening exercise for OA	Low health literacy (3)
B2	Fear (patients required a lot of encouragement and reassurance)	Low health literacy (3)
B3	Being apprehensive about aggravating pain in patients	LIs are unsafe or have negative effects (1)
B4	People that don't particularly like exercise	Negative attitude toward LIs (3)
B5	Mental effort required for WBE program was challenging for patients	Challenges for patients during participation in LIs (1)
B6	Tending to avoid pushing patients in usual clinical practice	LIs are unsafe or have negative effects (1)
B7	Physical challenge was the complexity of WBE program	Challenges for patients during participation in LIs (1)
B8	Challenges associated with cuff weights used to apply resistance in NWBE program	Challenges for HCPs during delivery of LIs (1)
B9	Straight leg raise challenging in NWBE program	Challenges for patients during participation in LIs (1)
B10	Significant impact of other health problems on patients' ability to commit fully to exercise program	Negative impact of comorbidities (3)
F1	Experiences in study helped them push patients through more pain than they would have previously	Research environment or protocols provide a safety net (1)
F2	NWBE program was generally easier for patients to follow (mental effort)	Ease for patients during participation in LIs (1)
F3	NWBE program was easier to prescribe (mental effort)	Ease for HCPs during delivery of LIs (1)
F4	Most patients tolerated a lot more than was expected (amount of exercise)	Severity of disease and symptoms (3)
F5	Easier to prescribe and progress NWBE than WBE program (physical complexity)	Ease for HCPs during delivery of LIs (1)
F6	Patients adherent/easy to work with when they engaged in exercise program/started seeing improvements	High patient adherence or engagement (3)
F7	Strong therapeutic relationship with patients	Importance of communication and relationship (8)
F8	Importance of pain education and reassurance about safety and benefits of exercise	High health literacy or importance of education (3)
F9	Tailoring exercise programs to individual patient would overcome some challenges	Ability and importance of providing personalized treatment within LIs (1)

Lawford (2021) [40]

Number	Description	Subcategory (domain)
B1	Video consultations made it more difficult to have emotional conversations/read non-verbal cues	Negative aspects regarding communication and relationship using telehealth (1)
B2	Patients were apprehensive about managing weight by themselves	Low health literacy (3)
B3	Volume of resources could be overwhelming/confusing for some patients	Challenges for patients during participation in LIs (1)
F1	Simplicity and convenience of meal replacements	Ease for patients during participation in LIs (1)
F2	Video consultations were easy and convenient	Positive attitude or needs of HCPs regarding feasibility of telehealth (1)
F3	Pleasantly surprised by experience with video consultations (had some of the best conversations)	Positive aspects regarding communication and relationship using telehealth (1)
F4	Level of support patients had from family/people close to them seemed to make a big difference	Social support (3)
F5	Long-term follow-up consultations would be beneficial	Positive experiences with or suggestions to improve the content or structure of LIs (1)
F6	Cohort of patients, in general, was highly motivated (remained interested/motivated for entirety of 6 months)	High patient adherence or engagement (3)
F7	Rapid weight loss was primary driver of motivation	LIs have positive effects on general health (1)
F8	Extremely positive about educational resources provided	Positive experiences with or suggestions to improve the content or structure of LIs (1)
F9	More information about healthy eating beyond meal replacement phase could be included	Positive experiences with or suggestions to improve the content or structure of LIs (1)
F10	Exercise/physical activity program was an important part of intervention	Positive experiences with or suggestions to improve the content or structure of LIs (1)
F11	Large improvements in knee pain	LIs have positive effects on affected joint(s) (1)
F12	Positive lifestyle changes (patients) (e.g. thinking differently)	LIs have positive mental effects (1)

MacKay (2018) [41]

Number	Description	Subcategory (domain)
B1	Lack of infrastructure or local programmes (particularly in rural settings)	LIs are unavailable or inaccessible (1)
B2	Cost was a factor in whether clients could access facilities/programmes	Costs of LIs to patients (1)
B3	Lack of funding prevented clients from accessing services/seeking help/getting full course of treatment	Restrictions due to health insurance (7)
B4	Clients often had a waiting period before accessing care	LIs are unavailable or inaccessible (1)
B5	Wait lists as a burden	Lack of time due to other demands (or not further specified) (5)
B6	Lack of access to other healthcare providers (e.g. physicians with expertise in OA)	No access to other HCPs (4)
B7	Variability in confidence to provide weight management (not confident)	Lack of knowledge or skills around LIs or promoting behavioral change (2)

B8	Physicians who did not make timely referrals to physical therapy	Non-optimal interdisciplinary collaboration or healthcare provision (4)
B9	Physicians' attitudes could influence clients' perceptions and level of buy-in to physical therapy	Non-optimal interdisciplinary collaboration or healthcare provision (4)
B10	Restricted in amount of time they could allot per patient	Lack of time within patient consultations (5)
B11	Challenges in accessing scientific papers	Challenges in accessing information resources (5)
B12	Challenging work to get clients to initiate management and maintain it over the long term	Low patient adherence or engagement (3)
B13	Disconnect between PTs' recommendations for treatment and clients' expectations or preferences	Negative attitude toward LIs (3)
B14	Prior experiences with physical therapy influenced client expectations of clinical encounter	Negative attitude toward LIs (3)
B15	Some clients had misconceptions about OA (nothing they could do/normalising it as part of ageing)	Low health literacy (3)
B16	Some clients feared participation in exercise (concerns for further degeneration)	Low health literacy (3)
B17	Accepting diagnosis of OA could be particularly challenging for people with early OA	Low health literacy (3)
B18	Clients' socioeconomic status (e.g. great poverty, shelter system)	Limited financial resources (3)
B19	Clients' language (e.g. haven't mastered English/French)	Low health literacy (3)
B20	Clients' family responsibilities (e.g. busy, lot going on)	Other responsibilities (3)
B21	Clients' lifestyle (e.g. coping, attitude towards pain)	Low health literacy (3)
F1	Benefits of having infrastructure and programmes available in their communities	LIs are available or accessible, or suggestions for improvement (1)
F2	Access to a team on-site/a network of healthcare providers they trusted	Access to other HCPs (4)
F3	Importance of good working relationships	Good interdisciplinary collaboration or healthcare provision, or suggestions for improvement (4)
F4	Importance of effective mechanisms to communicate	Needs regarding communication and referral procedures (4)
F5	Confident in capabilities/skills to use strategies they believed to be effective within scope of practice	Having or improving knowledge or skills around LIs or promoting behavioral change (2)
F6	Identifying weight management as important	Positive attitude toward LIs (2)
F7	Variability in confidence to provide weight management (confident)	Having or improving knowledge or skills around LIs or promoting behavioral change (2)
F8	Treatment could improve clients' symptoms (e.g. reduce pain, increase function)	LIs have positive effects on affected joint(s) (1)
F9	Treatment could potentially slow progression of symptoms	LIs have positive effects on affected joint(s) (1)
F10	Physicians who expressed support for physical therapy/exercise and referred clients to physical therapy early	Good interdisciplinary collaboration or healthcare provision, or suggestions for improvement (4)
F11	Physical therapy scope of practice was adequate to manage clients with perceived early knee OA	Adequate professional paradigm or suggestions for expansion (6)
F12	Suggestion that it would be useful to expand scope of practice to include ordering diagnostic imaging	Adequate professional paradigm or suggestions for expansion (6)

F13	Having adequate time to spend with clients	Adequate duration of patient consultations (5)
F14	Access to current evidence	Access to information resources (5)
F15	Professional networks/community of practice as mechanism to facilitate sharing of information	Access to information resources (5)
F16	Client participation in management was critical to see improvement in symptoms	Importance of high patient adherence or engagement for effectiveness of LIs (3)
F17	Viewing themselves as having an important role in supporting clients to participate in management	Perception of own role potentially stimulating prescription or follow-up of LIs (2)
F18	Clients' pre-existing activity level (e.g. active person)	Other patient characteristics (3)
U1	Clients' general health	Other patient characteristics (3)
U2	Clients' occupation	Other responsibilities (3)

MacKay (2020) [42]

Number	Description	Subcategory (domain)
B1	Lack of confidence/uncertainty related to role in weight management	Perception of own role potentially impeding prescription or follow-up of LIs (2)
B2	Weight was touchy/sensitive subject to discuss	Challenges of discussing weight (8)
B3	Not confident in knowledge about weight management	Lack of knowledge or skills around LIs or promoting behavioral change (2)
B4	Perception that discussions related to diet were not part of their scope of practice	Perception of own role potentially impeding prescription or follow-up of LIs (2)
B5	Getting buy-in (engaging people in management) often portrayed as challenge	Low patient adherence or engagement (3)
B6	People's preferences were at odds with physical therapists' beliefs about management	Negative attitude toward LIs (3)
F1	Clinical experience helped to read the person's situation (identify approach to motivate them)	Having or improving knowledge or skills around LIs or promoting behavioral change (2)
F2	Integrating scientific evidence from studies into their approach to management	Access to information resources (5)
F3	Postgraduate continuing professional development courses to expand toolkit of therapeutic interventions	Having or improving knowledge or skills around LIs or promoting behavioral change (2)
F4	Interventions in physical therapists' toolbox were not static (changed over time)	LIs are feasible or sustainable (1)
F5	Perception that exercise and physical activity were central to management	Positive attitude toward LIs (2)
F6	Acknowledging that weight management was a component of management	Positive attitude toward LIs (2)
F7	Confidence in addressing weight management	Having or improving knowledge or skills around LIs or promoting behavioral change (2)
F8	Routinely including education about weight management	Perception of own role potentially stimulating prescription or follow-up of LIs (2)
F9	Feeling comfortable discussing role of physical activity in maintaining weight control	Factors that could ease the way to discussing weight (8)
F10	Those with access to other clinicians recommended to consult another clinician for advice	Access to other HCPs (4)

	on diet as needed	
F11	Developing rapport with people made it easier to discuss weight management	Factors that could ease the way to discussing weight (8)
F12	Getting buy-in (engaging people in management) critical to improving outcomes	Importance of high patient adherence or engagement for effectiveness of LIs (3)
F13	Playing a role in promoting engagement in management	Perception of own role potentially stimulating prescription or follow-up of LIs (2)
F14	Education contributed to buy-in to treatment (pathology, consequences, treatments)	High health literacy or importance of education (3)
F15	Tailoring treatment to a person's goals/interests	Ability and importance of providing personalized treatment within LIs (1)
F16	Need to consider personal context by integrating people's home exercises into daily activities/other life demands	Ability and importance of providing personalized treatment within LIs (1)
F17	Improve people's symptoms early in treatment (to gain buy-in)	LIs have positive effects on affected joint(s) (1)
F18	Having positive attitude/being encouraging of small changes/being hopeful about OA management	Importance of communication and relationship (8)
U1	Driven by their professional experience of what does and doesn't work/trial and error	Clinical experience (2)
U2	Treatments were based more on what works clinically (opposed to scientific evidence)	Clinical experience (2)
U3	Treatment decisions depended on people's symptoms/findings of physical assessment	Severity of disease and symptoms (3)

Mann (2011) [43]

Number	Description	Subcategory (domain)
B1	Insufficient information for OA patients (e.g. not providing leaflets)	Low health literacy (3)
B2	Doubts about patients' willingness to make behavioral changes	Negative attitude toward LIs (3)
B3	Unrealistic expectations of the outcome of joint replacement among patients	Positive attitude toward TJA (3)
B4	OA was not given enough attention, symptoms were often dismissed/minimized in health care	OA seen as low priority (9)
B5	Lack of provision for patients who were not candidates for surgery (too long without help)	Non-optimal interdisciplinary collaboration or healthcare provision (4)
B6	Patients lacked proactive follow-up to support self-management	Non-optimal interdisciplinary collaboration or healthcare provision (4)
B7	Lack of time to give patients sufficient opportunity to discuss their condition	Lack of time within patient consultations (5)
B8	General lack of expertise/interest in OA (that could lead to inappropriate referral/suboptimal access to services)	Lack of knowledge or skills around OA care in general (2)
B9	Lack of facilities to promote continuing exercise in community	LIs are unavailable or inaccessible (1)
B10	Lack of coordination between leisure, social and health services	Non-optimal interdisciplinary collaboration or healthcare provision (4)
B11	Wait for physiotherapy was too long	LIs are unavailable or inaccessible (1)
B12	Insufficient (physiotherapy) intervention when patients were seen	Non-optimal interdisciplinary collaboration or healthcare provision (4)
B13	Belief that physiotherapists did not find it rewarding/interesting to treat OA patients	Non-optimal interdisciplinary collaboration or healthcare provision (4)

F1	Need for early education about OA/self-management and treatment options and opportunity to discuss these	High health literacy or importance of education (3)
F2	Patients would be better served by long-term condition model of care (e.g. diabetes mellitus)	Good interdisciplinary collaboration or healthcare provision, or suggestions for improvement (4)
F3	Patients should initiate own follow-up when needed (as better use of time/health care resources)	Good interdisciplinary collaboration or healthcare provision, or suggestions for improvement (4)
F4	Allow patients, after initial referral, to use direct access system to service (no need for re-referral)	Good interdisciplinary collaboration or healthcare provision, or suggestions for improvement (4)
F5	Care could be improved if every GP practice contained an individual who took a particular interest in OA	Access to other HCPs (4)
F6	There should be OA specialist clinicians (all relevant allied health professions) providing services in community	Access to other HCPs (4)

Miller (2020) [44]

Number	Description	Subcategory (domain)
B1	No effective treatment options	OA seen as untreatable and local condition (wear-and-tear) (9)
B2	Patients don't want to expend effort towards lifestyle change	Negative attitude toward LIs (3)
B3	Lifestyle counseling is huge time commitment	Lack of time within patient consultations (5)
B4	Difficulty convincing patients to consider non-surgical, non-medication treatments	Low health literacy (3)
B5	Lack of physician education on OA care	Lack of knowledge or skills around OA care in general (2)
B6	Patient body weight (overweight/obese) (impedes exercise/makes visits to services more difficult)	Negative impact of comorbidities (3)
B7	Costs related to weight loss can be prohibitive for patients with limited resources (financial burdens)	Limited financial resources (3)
B8	Lack of patient self-efficacy (regarding lifestyle changes)	Low health literacy (3)
B9	Patients delay care until they are highly symptomatic (missing opportunities to slow disease progression)	Severity of disease and symptoms (3)
B10	Lack of knowledge about OA (patient barrier)	Low health literacy (3)
B11	Costs to patients (lack of insurance coverage/high co-pays for specific services/time off work/travel expenses)	Costs of LIs to patients (1)
B12	Inaccessible treatment options within organization	LIs are unavailable or inaccessible (1)
B13	Lack of resources for face-to-face patient education and patient reference	Lack of information resources (5)
B14	Challenges when coordinating multimodal care (including difficulties with the referral system)	Challenges of communication and referral procedures (4)
B15	Appointment times too short to address all of patient's issues and provide lifestyle counseling	Lack of time within patient consultations (5)
B16	Difficulty finding high quality, patient-friendly OA educational materials	Challenges in accessing information resources (5)

B17	Frustration that material found on Internet or provided by friends/family was frequently inaccurate	Challenges in accessing information resources (5)
B18	Surgical methods have the best outcomes	LIs have little or no effect on OA (1)
B19	Changing own practice style remained as barrier after OA training	Perception of own role potentially impeding prescription or follow-up of LIs (2)
F1	Importance of provider knowledge regarding OA management	Having or improving knowledge or skills around OA care in general (2)
F2	Physical therapy helpful for patients most of the time	LIs have positive effects (not further specified) (1)
F3	Utilising clinic health educator who met with patients for weight loss discussions and followed up by phone	Good interdisciplinary collaboration or healthcare provision, or suggestions for improvement (4)
F4	Employing a multi-pronged approach to engage patients in weight loss	Good interdisciplinary collaboration or healthcare provision, or suggestions for improvement (4)
F5	Patients who are well insured have improved access to services (e.g. physical therapy)	Benefits of good health insurance (7)
F6	Physician education on OA management can affect both provider and patient attitudes	Having or improving knowledge or skills around OA care in general (2)
F7	Reframe discussions around exercise and weight loss (e.g. not blaming/discouraging people)	Factors that could ease the way to discussing weight (8)
F8	Recommending informational materials for patients (to mitigate delays in OA care)	Availability of information resources (5)
F9	Standardised flowsheet on OA management (as guide for providers/tool for patient discussions)	Availability of information resources (5)
F10	Electronic reminders for physicians on how to locate OA treatment information and resources	Potential use of information technology (5)

Nielsen (2014) [45]

Number	Description	Subcategory (domain)
B1	Concerns about capacity to learn/not having skills to fulfill study expectations/deal with challenging patients	Lack of knowledge or skills around LIs or promoting behavioral change (2)
B2	Requirements of RCT potentially created a barrier to responding to where the client was	Insufficient ability to provide personalized treatment within LIs (1)
B3	Difficulty for patients with PCST component (cognitive restructuring techniques)	Challenges for patients during participation in LIs (1)
B4	Not have sufficient skills to present PCST component (cognitive restructuring techniques) effectively	Lack of knowledge or skills around LIs or promoting behavioral change (2)
B5	Some process skills were dissimilar to pre-existing clinical communication skills and challenging to use	Lack of knowledge or skills around LIs or promoting behavioral change (2)
B6	Time required to teach PCST skills to patients	Lack of time within patient consultations (5)
B7	Concern about capacity to recover costs of incorporating CBT into practice	Limited financial resources within organization (5)
B8	Lack of knowledge about CBT (necessary to participate in training/RCT to fully appreciate value of CBT to practice)	Lack of knowledge or skills around LIs or promoting behavioral change (2)
B9	Public expectation of what physical therapy treatment should be (e.g. didn't come to have thinking challenged)	Negative attitude toward LIs (3)

F1	Training workshop as good introduction to content and process of delivering PCST program	Ease for HCPs during delivery of LIs (1)
F2	Weekly group interaction crucial to being able to deliver intervention effectively/problem-solve issues	Ease for HCPs during delivery of LIs (1)
F3	Input from supervising psychologist crucial to being able to deliver intervention effectively/problem-solve issues	Ease for HCPs during delivery of LIs (1)
F4	Would have liked more role-playing experience prior to beginning trial treatments	Ease for HCPs during delivery of LIs (1)
F5	Favorably comments on program content (positive way to help people be proactive about their pain)	Positive experiences with or suggestions to improve the content or structure of LIs (1)
F6	Opportunity to review PCST skills and learn more structured/deliberate ways of incorporating these into practice	Having or improving knowledge or skills around LIs or promoting behavioral change (2)
F7	Some modules worked better than others (depending on the individual patient and context)	Ability and importance of providing personalized treatment within LIs (1)
F8	Importance of PCST component (cognitive restructuring techniques)	Positive experiences with or suggestions to improve the content or structure of LIs (1)
F9	Structure of PCST sessions (overview/practice review/covering new skill/practice planning) worked well	Positive experiences with or suggestions to improve the content or structure of LIs (1)
F10	Regular group meetings were considered very important (if not essential) for delivery of PCST program	Ease for HCPs during delivery of LIs (1)
F11	Value of having a psychologist involved throughout the program, their professional input was helpful	Ease for HCPs during delivery of LIs (1)
F12	Expecting to utilize/continue integrating PCST in general clinical work as physical therapist (beyond the study)	Positive attitude toward LIs (2)
F13	The belief that a more flexible approach responsive to patient needs was required in their practice	Ability and importance of providing personalized treatment within LIs (1)
F14	Increasing confidence in using PCST skills over the course of the study	Having or improving knowledge or skills around LIs or promoting behavioral change (2)
F15	Improved interpersonal skills with general clinical patients as a result of participating in the study	Having or improving knowledge or skills around LIs or promoting behavioral change (2)
F16	Value of increasing profession's explicit understanding/use of PCST skills (practice model may be required)	Adequate professional paradigm or suggestions for expansion (6)
F17	Incorporating selected PCST components on as-needed basis most practical way within current environment	Adequate duration of specific interventions or protocols (5)
F18	Value of incorporating aspects of PCST mind-set into professional training (entry-level vs. postgraduate level)	Adequate professional paradigm or suggestions for expansion (6)

Okwera (2019) [46]

Number	Description	Subcategory (domain)
B1	Incurable nature and negative prognosis of OA	OA seen as untreatable and local condition (wear-and-tear) (9)
B2	Medical professionals saw OA as low priority with respect to managing their workload	OA seen as low priority (9)
B3	Frustrations about restrictive referral pathways	Challenges of communication and referral procedures (4)
B4	Frustrations about lack of autonomy with decision-making	Negative attitude toward guidelines or protocols (2)
B5	Only two GPs had clear understanding of clinical guidelines on OA	Lack of knowledge or skills around OA care in general (2)
B6	Negativity toward guidelines (clinical reasoning more important)	Negative attitude toward guidelines or protocols (2)
B7	Feeling that patients tended to prefer treatment administered to them	Negative attitude toward LIs (3)
B8	Lack of compliance with home exercise regimes and advice given to patients was common	Low patient adherence or engagement (3)
B9	Lack of confidence in clinical effectiveness of physiotherapy treatments	LIs have little or no effect on OA (1)
B10	Negative comments about patient reports of a lack of “hands-on” physiotherapy	Negative attitude toward LIs (3)
B11	Criticizing the decision to centralize musculoskeletal physiotherapy service (useful to have somebody in team)	Non-optimal interdisciplinary collaboration or healthcare provision (4)
B12	Frustrations about lack of continuity regarding team of physiotherapists within clinic	Non-optimal interdisciplinary collaboration or healthcare provision (4)
B13	Frustrations about lack of contact/communication involved in the referral and discharge process	Lack of communication between HCPs (4)
B14	Referral process was convoluted and at times irrelevant	Challenges of communication and referral procedures (4)
B15	Not working closely with physiotherapists/frustrations about working relationship	Non-optimal interdisciplinary collaboration or healthcare provision (4)
B16	Dissatisfaction about loss of communication since centralizing musculoskeletal physiotherapy service	Lack of communication between HCPs (4)
B17	Dissatisfaction about loss of coherent working since centralizing musculoskeletal physiotherapy service	Non-optimal interdisciplinary collaboration or healthcare provision (4)
F1	There is place for each (self-management programs/physiotherapy/orthopedic consultants) in OA management	Positive attitude toward LIs (2)
F2	Positivity toward private sector (patients will get seen a lot quicker)	Benefits of good health insurance (7)
F3	Reasonable understanding of role physiotherapy plays in management of lower-limb OA	Having or improving knowledge or skills around LIs or promoting behavioral change (2)
F4	Overall positive experience of physiotherapy service and therapists	Good interdisciplinary collaboration or healthcare provision, or suggestions for improvement (4)
F5	Need for improving communication (quality of referrals, information at discharge)	Improving communication between HCPs (4)
F6	In-house physiotherapy (as suggestion for physiotherapy service improvement)	Access to other HCPs (4)
F7	Streamlining the physiotherapy referral process (as suggestion for physiotherapy service improvement)	Needs regarding communication and referral procedures (4)
F8	Training sessions (as suggestion for physiotherapy service improvement)	Having or improving knowledge or skills around OA care in general (2)
F9	Triage service (as suggestion for physiotherapy service improvement)	LIs are available or accessible, or suggestions for improvement (1)
F10	Private healthcare supplementation (as suggestion for physiotherapy service improvement)	Benefits of good health insurance (7)

F11	A web-based physiotherapy service (as suggestion for physiotherapy service improvement)	LIs are available or accessible, or suggestions for improvement (1)
F12	Reduced waiting times (as suggestion for physiotherapy service improvement)	LIs are available or accessible, or suggestions for improvement (1)
U1	Management strategies depended on what the person wants	Patients' preferences (3)
U2	Management strategies depended on what the person can cope with	Health literacy (3)
U3	Management strategies depended on how bad the knee is	Severity of disease and symptoms (3)

Poitras (2010) [47]

Number	Description	Subcategory (domain)
B1	Knee OA seen as uninteresting health problem on which they had limited impact and could not cure	OA seen as untreatable and local condition (wear-and-tear) (9)
B2	Knee OA more often diagnosed as an unanticipated comorbidity (rarely primary reason for consultation)	OA seen as low priority (9)
B3	Not enough emphasis put on primary prevention of knee OA	OA seen as low priority (9)
B4	Most GPs believed their contribution was essentially limited to diagnosis of condition and medication	Perception of own role potentially impeding prescription or follow-up of LIs (2)
B5	Knee OA management seen as unchallenging routine	OA seen as low priority (9)
B6	Rehabilitation potential depended on length of disability (less potential with late management)	Severity of disease and symptoms (3)
B7	Benefits obtained in the long term, which often conflicted with patient expectations for short-term benefits	Negative attitude toward LIs (3)
B8	Paracetamol could mask pain/underlying physical problem (reducing opportunity to assess/manage problem)	Severity of disease and symptoms (3)
B9	Potential further damage to the knee due to activity	LIs are unsafe or have negative effects (1)
B10	Difficult to obtain effective analgesia with some patients	Severity of disease and symptoms (3)
B11	Lack of patient motivation in remaining active despite knee OA	Negative attitude toward LIs (3)
B12	Person had been sedentary throughout life	Other patient characteristics (3)
B13	Potential to create unrealistic expectations and discouragement in patients that were too disabled	Severity of disease and symptoms (3)
B14	Patient views and expectations rarely matched patient needs	Negative attitude toward LIs (3)
B15	Questioning capacity to perform regular exercise because of severity of disability	Severity of disease and symptoms (3)
B16	Questioning capacity to perform regular exercise because of age	Other patient characteristics (3)
B17	Questioning capacity to perform regular exercise because of general health	Negative impact of comorbidities (3)
B18	Questioning capacity to perform regular exercise because of motivation	Negative attitude toward LIs (3)
B19	Disagreement on effective exercise parameters	Non-optimal interdisciplinary collaboration or healthcare provision (4)
B20	Disagreement on optimal design of exercise programs to increase adherence	Non-optimal interdisciplinary collaboration or healthcare provision (4)

B21	Unclear on amount and type of activity necessary to obtain benefits without further damaging the knee	Lack of knowledge or skills around LIs or promoting behavioral change (2)
B22	Most patients demonstrated fatalism/inadequate knowledge and beliefs related to knee OA management	Low health literacy (3)
B23	Patients' adherence to management recommendations was limited (because of fatalism)	Low patient adherence or engagement (3)
B24	Limited impact of weight loss on established knee OA (more effective as a primary prevention strategy)	LIs have little or no effect on OA (1)
B25	Questioning direct relationship between weight and knee OA (numerous other factors associated)	LIs have little or no effect on OA (1)
B26	Knee pain restricts activities in general (which makes weight loss difficult)	Severity of disease and symptoms (3)
B27	Patients with knee OA tended to be older/less active/with slower metabolism (which makes weight loss difficult)	Other patient characteristics (3)
B28	Weight loss is difficult (multiplicity of factors need to be addressed, often involving change in lifestyle)	Potential effects of LIs are difficult to accomplish (1)
F1	Knee OA seen as technically challenging condition	Optimistic views toward OA (9)
F2	Necessity of physiotherapy to effectively rehabilitate knee OA patients (because of knowledge/availability)	Perception of own role potentially stimulating prescription or follow-up of LIs (2)
F3	Rehabilitation potential depended on length of disability (better outcomes with early management)	Severity of disease and symptoms (3)
F4	Many interventions should be used before resorting to medication (including physiotherapy)	Positive attitude toward LIs (2)
F5	Other interventions (including physiotherapy) should be used before paracetamol	Positive attitude toward LIs (2)
F6	NSAIDs alone are not sufficient to appropriately treat inflammation and have to be combined with physiotherapy	Positive attitude toward LIs (2)
F7	Importance of PT's role in educating patients with regards to NSAIDs/alternatives (including physiotherapy)	Perception of own role potentially stimulating prescription or follow-up of LIs (2)
F8	Benefits of activity on knee mobility	LIs have positive effects on affected joint(s) (1)
F9	Benefits of activity on general wellbeing	LIs have positive effects on general health (1)
F10	Effective analgesia necessary for patients to be able to accomplish activities	Severity of disease and symptoms (3)
F11	Patients should be encouraged to resume/maintain daily activities	Positive attitude toward LIs (2)
F12	Necessity of PT involvement in managing activity (because potentially detrimental if excessive)	Perception of own role potentially stimulating prescription or follow-up of LIs (2)
F13	GPs believed PT involvement was necessary to motivate the patient and manage the exercise program	Good interdisciplinary collaboration or healthcare provision, or suggestions for improvement (4)
F14	Exercise planning is usually PT's role (rather than GP's)	Perception of own role potentially stimulating prescription or follow-up of LIs (2)
F15	Exercise programs have to be individualized to each patient by the PT	Ability and importance of providing personalized treatment within LIs (1)

F16	Necessity of PT follow-up sessions to assess and encourage patient adherence	Perception of own role potentially stimulating prescription or follow-up of LIs (2)
F17	Activity necessary for the knee’s health	LIs have positive effects on affected joint(s) (1)
F18	PT's role to individualize patients’ activity according to needs and capacity	Perception of own role potentially stimulating prescription or follow-up of LIs (2)
F19	Necessity of patients’ active participation in knee OA management (to achieve significant outcomes)	Importance of high patient adherence or engagement for effectiveness of LIs (3)
F20	Although agreeing with active patient participation, it is ultimately PT's role to appropriately manage patients	Perception of own role potentially stimulating prescription or follow-up of LIs (2)
F21	Weight loss effective at improving mobility in general	LIs have positive effects on general health (1)
F22	Weight loss improves pain and joint function	LIs have positive effects on affected joint(s) (1)
F23	Relationship with patients, developed through numerous sessions, facilitated influence for lifestyle modifications	Factors that could ease the way to discussing weight (8)
F24	Weight loss also benefits mobility in general	LIs have positive effects on general health (1)

Rosemann (2006) [48]

Number	Description	Subcategory (domain)
B1	Depression as important barrier to motivate patients to physical exercise	Negative impact of comorbidities (3)
B2	Feeling pressure by patients to refer them to specialist	Positive attitude toward TJA (3)
B3	Specialist did not take time to explain what they had examined/x-rays he had taken	Lack of communication between HCPs (4)
B4	Treatments (e.g. physiotherapy) prescribed less frequently due to decreasing financial resources	Limited financial resources within organization (5)
B5	Treatments (e.g. physiotherapy) prescribed less frequently due to increasing restrictions by health insurances	Restrictions due to health insurance (7)
B6	Not focusing on increasing patients’ motivation for behavioural change, but just giving general recommendations	Perception of own role potentially impeding prescription or follow-up of LIs (2)
B7	Success rate in motivating patients too low (distinctly resignated regarding their impact on patients’ life style)	Negative attitude toward LIs (3)
B8	Vicious circle (pain when exercising, people move less/eat more due to frustration/sometimes depression)	Potential effects of LIs are difficult to accomplish (1)
B9	Belief that nobody is willing to change lifestyle due to OA, disease has to be a lot worse	OA seen as low priority (9)
B10	Lack of information about self-help groups/offers on community level	Lack of information resources (5)
B11	Frustration about impact of information (e.g. self-help groups) (lot of patients find excuses not to participate)	Negative attitude toward LIs (3)
B12	Missing information about offers e.g. in the community	Lack of information resources (5)
B13	Lack of time	Lack of time due to other demands (or not further specified) (5)

B14	No knowledge about treatment	Lack of knowledge or skills around OA care in general (2)
F1	Gate keeper role for GPs could reduce patients' pressure to refer to orthopaedics/decrease performed x-rays	Good interdisciplinary collaboration or healthcare provision, or suggestions for improvement (4)
F2	Better communication with specialists could increase efficacy of treatment	Improving communication between HCPs (4)
F3	Payment system has to be changed to upgrade conservative treatments and conversation with patient	Financial reward for implementing LIs (5)
F4	Involvement of practice nurses is imaginable in the area of life style counselling and advice giving	Good interdisciplinary collaboration or healthcare provision, or suggestions for improvement (4)
F5	Interventions performed by practice nurses have to be reinsured sufficiently	Financial reward for implementing LIs (5)
F6	Desire to be more involved in life style counselling (upgrade of profession)	Perception of own role potentially stimulating prescription or follow-up of LIs (2)
F7	Knowledge about treatment	Having or improving knowledge or skills around OA care in general (2)
F8	Integrating patients' social system into treatment	Social support (3)
F9	More openly address psychological complaints of patients	Importance of communication and relationship (8)

Selten (2017) [49]

Number	Description	Subcategory (domain)
B1	Ambivalent about patients' ability to lose weight (not able to succeed in making lifestyle changes)	Low health literacy (3)
B2	Mistrust in interventions dieticians use to help patients' with weight reduction attempts	Non-optimal interdisciplinary collaboration or healthcare provision (4)
B3	Mentioning benefits of weight reduction, but not actively coaching or referring patients	Perception of own role potentially impeding prescription or follow-up of LIs (2)
B4	The belief that patients are not capable of losing weight	Low health literacy (3)
B5	Weight reduction advice takes too much time in a consultation	Lack of time within patient consultations (5)
B6	Not perceiving weight reduction advice as their responsibility	Perception of own role potentially impeding prescription or follow-up of LIs (2)
B7	Difficulties in communicating with patients about being overweight	Challenges of discussing weight (8)
B8	Uncertainties about dosage/frequency/type of physical activity	Lack of knowledge or skills around LIs or promoting behavioral change (2)
B9	Less certain about effectiveness of physical therapy (benefits variable or difficult to prove)	LIs have little or no effect on OA (1)
B10	Negative views about physical therapists who provided non-evidence-based treatments	Non-optimal interdisciplinary collaboration or healthcare provision (4)
B11	Mistrust because they observed huge differences in quality of care delivered by physical therapists	Non-optimal interdisciplinary collaboration or healthcare provision (4)
B12	Occupational therapists, podiatrists and physical therapists do not work together optimally in OA care	Non-optimal interdisciplinary collaboration or healthcare provision (4)
B13	Role of rheumatologist in knee/hip OA care perceived as unclear/limited	Non-optimal interdisciplinary collaboration or healthcare provision (4)

B14	Agreement that orthopedic surgeon's primary task is to assess whether patient is eligible for surgery	Non-optimal interdisciplinary collaboration or healthcare provision (4)
B15	Orthopedic surgeons were perceived negatively by several healthcare providers	Non-optimal interdisciplinary collaboration or healthcare provision (4)
F1	Benefits of weight reduction for relieving symptoms of knee/hip OA	LIs have positive effects on affected joint(s) (1)
F2	Ambivalent about patients' ability to lose weight (able)	High health literacy or importance of education (3)
F3	Dieticians are helpful for patients trying to lose weight	Good interdisciplinary collaboration or healthcare provision, or suggestions for improvement (4)
F4	Having a relationship with patient built on mutual trust/respect would ease way to discussing weight reduction	Factors that could ease the way to discussing weight (8)
F5	Value of lifestyle advice related to knee and hip OA	LIs have positive effects (not further specified) (1)
F6	Beneficial effects of physical therapy in reducing pain/stiffness and potential effects on cartilage	LIs have positive effects on affected joint(s) (1)
F7	Beneficial effects of physical therapy in reducing weight and for increasing mobility/posture/coordination	LIs have positive effects on general health (1)
F8	Physical therapy useful in increasing patients self-management in coping with/acceptance of symptoms	LIs have positive mental effects (1)
F9	Need for physical therapists to provide evidence-based exercises instead of non-evidence-based modalities	Good interdisciplinary collaboration or healthcare provision, or suggestions for improvement (4)
F10	Non-pharmacological, non-surgical treatment was considered useful to delay surgery	LIs have positive effects (not further specified) (1)
F11	Good communication with patient may help in delaying surgery	Importance of communication and relationship (8)
F12	Straightforward, easy and quick lines of communication among different disciplines in healthcare center	Needs regarding communication and referral procedures (4)
F13	Collaboration among multiple disciplines could be facilitated by working in a health center	Benefits of working in health centers (5)
F14	Non-pharmacological, non-surgical OA care can and should be provided in a primary care setting	Good interdisciplinary collaboration or healthcare provision, or suggestions for improvement (4)
F15	GPs have coordinating role (diagnose/monitor, refer when necessary, lifestyle education, long-term coach)	Good interdisciplinary collaboration or healthcare provision, or suggestions for improvement (4)
F16	Importance of having trust	Importance of communication and relationship (8)
F17	Physical therapists can guide patients/provide lifestyle advice (more time compared with GPs)	Good interdisciplinary collaboration or healthcare provision, or suggestions for improvement (4)
F18	Perceiving rheumatologists' role as valuable (giving injections, providing lifestyle/medication advice, refer)	Good interdisciplinary collaboration or healthcare provision, or suggestions for improvement (4)

Tang (2020) [50]

Number	Description	Subcategory (domain)
B1	Inability to discuss specific details of ACSM guideline	Lack of knowledge or skills around specific resources (2)

B2	Pain (main barrier resulting in reduced dosage prescription of strengthening exercises)	Severity of disease and symptoms (3)
B3	Patient's ability to exercise (main barrier resulting in reduced dosage prescription of strengthening exercises)	Severity of disease and symptoms (3)
B4	Unaware about practice guidelines in relation to aerobic exercise prescription/weight loss/pain management	Lack of knowledge or skills around LIs or promoting behavioral change (2)
B5	Knowledge about BMI/weight management was particularly poor (e.g. relying on visual estimations)	Lack of knowledge or skills around LIs or promoting behavioral change (2)
B6	Limited knowledge of how to address weight management	Lack of knowledge or skills around LIs or promoting behavioral change (2)
B7	Pain (key barrier to prescription of exercise as recommended by CPGs)	Severity of disease and symptoms (3)
B8	Less awareness about aerobic exercise prescription	Lack of knowledge or skills around LIs or promoting behavioral change (2)
B9	Pain (barrier to prescription of aerobic exercise)	Severity of disease and symptoms (3)
B10	Uncertainty over scope of practice/questioning whether weight and pain management fall outside scope	Perception of own role potentially impeding prescription or follow-up of LIs (2)
B11	Reduced confidence with recommending individual weight/pain management plans (discuss in general terms)	Lack of knowledge or skills around LIs or promoting behavioral change (2)
B12	Viewing weight as sensitive subject/feeling uncomfortable discussing it	Challenges of discussing weight (8)
F1	Knowledge/confidence in providing treatments related to strengthening and range of motion	Having or improving knowledge or skills around LIs or promoting behavioral change (2)
F2	Being aware about ACSM guidelines	Available resources might improve knowledge and decision-making (2)
F3	Confident in providing justifications for non-routinely adhering to guidelines (range of motion exercises)	Having or improving knowledge or skills around LIs or promoting behavioral change (2)
F4	Knowing the importance of weight management for knee OA	Positive attitude toward LIs (2)
F5	Being able to describe how they will manage pain during strengthening exercise	Having or improving knowledge or skills around LIs or promoting behavioral change (2)

Teo (2020) [51]

Number	Description	Subcategory (domain)
B1	Knee OA was perceived as a degenerative (wear and tear)	OA seen as untreatable and local condition (wear-and-tear) (9)
B2	Describing own role as prepping patients for knee surgery when they were referred for physiotherapy	Perception of own role potentially impeding prescription or follow-up of LIs (2)
B3	Comorbidities (often more severe pain, hampering ability to exercise or be physically active)	Negative impact of comorbidities (3)
B4	Patients' unsatisfactory adherence to exercise programs	Low patient adherence or engagement (3)
B5	Self-motivation (intrinsic barrier for patient adherence)	Negative attitude toward LIs (3)

B6	Fear of falling (intrinsic barrier for patient adherence)	Low health literacy (3)
B7	Fear of pain (intrinsic barrier for patient adherence)	Low health literacy (3)
B8	Costs (extrinsic barrier for patient adherence)	Costs of LIs to patients (1)
B9	Weather (extrinsic barrier for patient adherence)	Inconvenience to patients when accessing LIs (1)
B10	Patient expectations (not keen to participate in exercise/play active role in management, desire for quick fix)	Negative attitude toward LIs (3)
B11	Lack of confidence/knowledge/skills in implementing evidence into practice (e.g. weight management)	Lack of knowledge or skills around LIs or promoting behavioral change (2)
B12	Advice about how to lose weight was limited to brief general advice	Perception of own role potentially impeding prescription or follow-up of LIs (2)
B13	Considering weight loss to be outside own scope of practice (role of a dietician)	Perception of own role potentially impeding prescription or follow-up of LIs (2)
B14	Comfortable suggesting surgery to patients who responded poorly to conservative management	Perception of own role potentially impeding prescription or follow-up of LIs (2)
F1	Education focused on self-management strategies	High health literacy or importance of education (3)
F2	Importance of evaluating a patient's overall functional ability (rather than only knee signs/symptoms)	Other patient characteristics (3)
F3	Perceiving exercise prescription to be their main role	Perception of own role potentially stimulating prescription or follow-up of LIs (2)
F4	Importance of tailored exercise program	Ability and importance of providing personalized treatment within LIs (1)
F5	Advising patients against surgery for as long as possible (last option)	Perception of own role potentially stimulating prescription or follow-up of LIs (2)
F6	Implementing several strategies to boost adherence	Perception of own role potentially stimulating prescription or follow-up of LIs (2)
F7	Aware that being overweight/obese is risk factor for knee OA/losing weight is important	Positive attitude toward LIs (2)
F8	Not their role to advise the patient about knee surgery, opting not to discuss surgery at all	Perception of own role potentially stimulating prescription or follow-up of LIs (2)
F9	Advising patients against knee arthroscopy if specifically asked about this procedure	Perception of own role potentially stimulating prescription or follow-up of LIs (2)

Wallis (2020) [52]

Number	Description	Subcategory (domain)
B1	Cost (program access barrier)	Costs of LIs to patients (1)
B2	Transport, waiting time and parking related to attendance (program access barrier)	Inconvenience to patients when accessing LIs (1)
B3	Geography (program access barrier)	Inconvenience to patients when accessing LIs (1)
B4	Available session times (program access barrier)	Inconvenience to patients when accessing LIs (1)

B5	Using negative language to describe OA (wear-and-tear/joint damage/bone-on-bone/degenerative condition)	OA seen as untreatable and local condition (wear-and-tear) (9)
B6	Existing comorbidities (patient-related barrier)	Negative impact of comorbidities (3)
B7	Osteoarthritis severity (mild/severe) (patient-related barrier)	Severity of disease and symptoms (3)
B8	Lack of motivation to participate active lifestyle interventions (patient-related barrier)	Negative attitude toward LIs (3)
B9	Older age (patient-related barrier)	Other patient characteristics (3)
B10	Language/different cultural backgrounds (patient-related barrier)	Low health literacy (3)
B11	Work/other commitments precluding exercise-therapy (patient-related barrier)	Other responsibilities (3)
B12	Program factors (e.g. single discipline led intervention)	Non-optimal content or structure of LIs (1)
B13	Existing relationships with physiotherapists (as barrier to referral if patient already had treating physiotherapist)	Negative attitude toward LIs (3)
B14	Urging caution to patients about participating in higher impact exercise/activities	LIs are unsafe or have negative effects (1)
F1	A more holistic program as part of a multidisciplinary model of service was preferred	Positive experiences with or suggestions to improve the content or structure of LIs (1)
F2	Knowledge that program was delivered by well-trained and trusted physiotherapist	Good interdisciplinary collaboration or healthcare provision, or suggestions for improvement (4)
F3	Receiving communication back from program physiotherapist about patient outcomes	Improving communication between HCPs (4)
F4	Positive about program (alternative approach and opportunity to avoid a joint replacement)	LIs have positive effects (not further specified) (1)
F5	Exercise therapy may be effective by giving more muscular support for joints	LIs have positive effects on affected joint(s) (1)
F6	Exercise therapy may be effective by giving opportunity to improve confidence about activities/mobility	LIs have positive mental effects (1)
F7	Value of program's structure and peer (group) support	Positive experiences with or suggestions to improve the content or structure of LIs (1)
F8	Name of program (‘Good Life with OsteoArthritis’) implied optimism and positive outcome	Positive experiences with or suggestions to improve the content or structure of LIs (1)
F9	Received positive feedback from their patients about program	Positive experiences with or suggestions to improve the content or structure of LIs (1)
F10	Including links on websites of partners (suggestion for promotion and referrals)	Access to information resources (5)
F11	Simple, streamlined referral process (suggestion for promotion and referrals)	Needs regarding communication and referral procedures (4)
F12	Close, convenient locations (suggestion for promotion and referrals)	Convenience for patients when accessing LIs (1)
F13	Appropriate session times for working populations (suggestion for promotion and referrals)	Convenience for patients when accessing LIs (1)
F14	Specific information about program (suggestion for promotion and referrals)	Availability of information resources (5)
F15	Providing trial of sessions to assist patients to get started (suggestion for promotion and referrals)	Ease for patients during participation in LIs (1)
F16	Provision of free parking at health service (suggestion for promotion and referrals)	Convenience for patients when accessing LIs (1)

Cottrell (2016) [53]

Number	Description	Subcategory (domain)
B1	Insufficient time in consultations (*)	Lack of time within patient consultations (5)
B2	Limitations to accessing services (e.g. lack of facilities, costs) (*)	LIs are unavailable or inaccessible (1)
B3	Services do not meet expectations (*)	Non-optimal content or structure of LIs (1)
B4	Geographical problems (e.g. remote location, scared to walk in local area) (*)	Inconvenience to patients when accessing LIs (1)
B5	Cannot access necessary resources (*)	Challenges in accessing information resources (5)
B6	GP does not prioritise exercise (*)	Negative attitude toward LIs (2)
B7	Unclear what physio offers (*)	Non-optimal interdisciplinary collaboration or healthcare provision (4)
B8	Exercise does not match patient needs/expectations (*)	Negative attitude toward LIs (3)
B9	Achieving patient behavior change is difficult (*)	Potential effects of LIs are difficult to accomplish (1)
B10	GPs should (perhaps) not follow-up patients to monitor extent of continuation of exercises (*)	Perception of own role potentially impeding prescription or follow-up of LIs (2)
B11	It is the patient's own responsibility to continue doing their exercise programme (*)	Perception of own role potentially impeding prescription or follow-up of LIs (2)
B12	Increasing the overall activity levels does not/might not stop the knee problem getting worse (*)	LIs have little or no effect on OA (1)
B13	Time constraints prevent GPs from providing advice on individual exercises for CKP (*)	Lack of time within patient consultations (5)
F1	Physiotherapy (referral) needs to be prioritised	Positive attitude toward LIs (2)
F2	It is part of my job to reassure patients about the safety of exercise for CKP (*)	Perception of own role potentially stimulating prescription or follow-up of LIs (2)
F3	Exercise for CKP is most beneficial when it is tailored to meet individual patient needs (*)	Ability and importance of providing personalized treatment within LIs (1)
F4	A standard set of exercises is not/might not be sufficient for every patient with chronic knee problems (*)	Ability and importance of providing personalized treatment within LIs (1)
F5	GPs should educate patients with CKP about how to change their lifestyle for the better (*)	Perception of own role potentially stimulating prescription or follow-up of LIs (2)
F6	It is important that people with CKP increase their overall activity levels (*)	Positive attitude toward LIs (2)
F7	How well a patient complies with their exercise programme determines how effective it will be (*)	Importance of high patient adherence or engagement for effectiveness of LIs (3)
F8	GPs should prescribe quadriceps strengthening exercises to every patient with CKP (*)	Perception of own role potentially stimulating prescription or follow-up of LIs (2)
F9	GPs should prescribe general exercise (e.g. walking or swimming) for every patient with CKP (*)	Perception of own role potentially stimulating prescription or follow-up of LIs (2)
F10	Knee problems are improved by quadriceps strengthening exercises (*)	LIs have positive effects on affected joint(s) (1)
F11	Knee problems are improved by general exercise (e.g. walking or swimming) (*)	LIs have positive effects on affected joint(s) (1)

F12	Quadriceps strengthening exercises for the knee are safe for everybody to do (*)	LIs are safe (1)
F13	General exercise (e.g. walking or swimming) is safe for everybody to do (*)	LIs are safe (1)
F14	Exercise is effective for patients if an X-ray shows severe knee OA (*)	Severity of disease and symptoms (3)
F15	Increasing the strength of the muscles around the knee stops the knee problem getting worse (*)	LIs have positive effects on affected joint(s) (1)
F16	Exercise for CKP should (perhaps) preferably be used before drug treatment has been tried (*)	Positive attitude toward LIs (2)
F17	Exercise for CKP is more effectively provided by physiotherapists than GPs (*)	Good interdisciplinary collaboration or healthcare provision, or suggestions for improvement (4)
U1	Exercise does not/might not work just as well for everybody, regardless of the amount of pain they have (*)	Severity of disease and symptoms (3)

Duarte (2019) [54]

Number	Description	Subcategory (domain)
B1	Main concern was participant adherence to physical activity routines after end of program	Low patient adherence or engagement (3)
F1	Improvement in the physical condition of participants	LIs have positive effects (not further specified) (1)
F2	Enthusiastic participation of the participants	High patient adherence or engagement (3)

Hill (2018) [55]

Number	Description	Subcategory (domain)
B1	Not (or perhaps not) interested in being the orthopedic surgeon in an ortho-bariatric centre (*)	Perception of own role potentially impeding prescription or follow-up of LIs (2)
F1	There was a BMI threshold above which they would not perform a TKA at all (*)	Perception of own role potentially stimulating prescription or follow-up of LIs (2)
F2	There was a BMI threshold above which they would not perform a TKA until the patient had attended a weight management program (*)	Perception of own role potentially stimulating prescription or follow-up of LIs (2)
F3	Weight loss should be the first-line treatment in the management of obese patients with symptomatic knee OA (*)	Positive attitude toward LIs (2)
F4	Obese patients with symptomatic knee OA should be referred to a specialist weight management service before orthopaedic assessment (*)	Positive attitude toward LIs (2)
F5	Obese patients with symptomatic knee OA should be assessed by a specialist multidisciplinary service, which should include an orthopaedic surgeon (*)	Good interdisciplinary collaboration or healthcare provision, or suggestions for improvement (4)
F6	Support for creation of regional centres where orthopedic surgeons and bariatric surgeons, with their respective teams, could assess obese patients with symptomatic knee pain (*)	Good interdisciplinary collaboration or healthcare provision, or suggestions for improvement (4)

Hill (2018) [56]

Number	Description	Subcategory (domain)
B1	I don't have/might not have the required knowledge and training around obesity care (*)	Lack of knowledge or skills around LIs or promoting behavioral change (2)
B2	Weight management services are not/might not be adequately commissioned in my area (*)	LIs are unavailable or inaccessible (1)
F1	Weight loss should be the first-line treatment in the management of symptomatic knee OA in obesity (*)	Positive attitude toward LIs (2)
F2	Community interventions are effective at achieving sufficient and sustained weight loss (*)	LIs have positive effects on general health (1)
F3	Obese patients with symptomatic knee OA should be referred to a specialist weight management service before orthopaedic assessment (*)	Good interdisciplinary collaboration or healthcare provision, or suggestions for improvement (4)
F4	Obese patients with symptomatic knee OA should be assessed by a specialist multidisciplinary service (*)	Good interdisciplinary collaboration or healthcare provision, or suggestions for improvement (4)
F5	Support for creation of regional centres where orthopaedic surgeons and bariatric surgeons, with their respective teams, could assess obese patients with symptomatic knee pain (*)	Positive attitude toward LIs (2)
F6	Intention to refer patients to an ortho-bariatric centre if it existed (*)	Perception of own role potentially stimulating prescription or follow-up of LIs (2)

Hofstede (2016) [57]

Number	Description	Subcategory (domain)
F1	Clear referral criteria/guideline (*)	Availability of information resources (5)
F2	Important to follow guidelines (*)	Positive attitude toward guidelines or protocols (2)
F3	Important to try non-surgical treatments first (*)	Positive attitude toward LIs (2)
F4	Only few drawbacks for the use of non-surgical treatments (*)	LIs are safe (1)
F5	Patients benefit from weight loss (*)	LIs have positive effects on general health (1)
F6	Non-surgical treatments motivate patients to do things themselves (*)	LIs have positive mental effects (1)
F7	Good results of physical therapy (*)	LIs have positive effects (not further specified) (1)
F8	Important to delay a surgery as long as possible (*)	Positive attitude toward LIs (2)
F9	Agreements with colleagues about the content of the care trajectory (*)	Good interdisciplinary collaboration or healthcare provision, or suggestions for improvement (4)
F10	Peer review/audit of professional association (*)	Audit (6)
F11	Positive attitudes of colleagues about non-surgical treatments (*)	Good interdisciplinary collaboration or healthcare provision, or suggestions for improvement (4)
F12	Clarity on what the patient has done at the physical therapist (*)	Improving communication between HCPs (4)
F13	Agreements/ deliberations with primary care (GP, physical therapist, dietician) (*)	Good interdisciplinary collaboration or healthcare provision, or suggestions for improvement (4)

F14	Availability of non-surgical treatments (*)	LIs are available or accessible, or suggestions for improvement (1)

Lawford (2018) [58]

Number	Description	Subcategory (domain)
B1	Using the telephone to consult with an OA patient and prescribe an exercise program would not/might not be easy for me (*)	Other challenges for HCPs regarding feasibility of telehealth (1)
B2	I would not/might not be as satisfied talking to an OA patient over the telephone as I would be talking to the patient in person in my consulting room (*)	Other challenges for HCPs regarding feasibility of telehealth (1)
B3	An exercise program prescribed by a PT over the telephone would not/might not improve a patient's OA (*)	Disadvantages of telehealth in terms of effectiveness (1)
B4	I would not/might not be able to adequately monitor a patient's OA over the telephone (*)	Challenges for HCPs regarding lack of physical/visual contact (1)
B5	I do not/might not like that there would be no physical contact with an OA patient when consulting over the telephone (*)	Challenges for HCPs regarding lack of physical/visual contact (1)
B6	I do not/might not like that there would be no physical contact with an OA patient when consulting over the internet video (*)	Challenges for HCPs regarding lack of physical/visual contact (1)
B7	I would not/might not be interested in being involved in a service offering PT-prescribed exercise over the telephone for my people with OA (*)	Other challenges for HCPs regarding feasibility of telehealth (1)
B8	Using the telephone would not/might not be an acceptable way for me to deliver an exercise program to patients with OA (*)	Other challenges for HCPs regarding feasibility of telehealth (1)
B9	Using the telephone would not/might not be a useful (practical) way for me to deliver an exercise program to patients with OA (*)	Other challenges for HCPs regarding feasibility of telehealth (1)
B10	Using the telephone would not/might not be an effective way for me to deliver an exercise program to patients with OA (*)	Other challenges for HCPs regarding feasibility of telehealth (1)
B11	Using the telephone would not/might not be a safe way for patients to receive a PT-prescribed exercise program for their OA (*)	Telehealth is not safe for patients or patient/data privacy (1)
F1	Exercise is beneficial for OA (*)	LIs have positive effects on affected joint(s) (1)
F2	I would get a good understanding of a patient's OA over the telephone (*)	Lack of physical/visual contact not a major issue for HCPs (1)
F3	I would get a good understanding of a patient's OA over the internet video (*)	Lack of physical/visual contact not a major issue for HCPs (1)
F4	A patient's privacy would not be violated if I prescribed them an exercise program over the telephone (*)	Telehealth is safe for patients or patient/data privacy (1)
F5	A patient's privacy would not be violated if I prescribed them an exercise program over the internet video (*)	Telehealth is safe for patients or patient/data privacy (1)
F6	Using the internet video to consult with an OA patient and prescribe an exercise program would be easy for me (*)	Positive attitude or needs of HCPs regarding feasibility of telehealth (1)

F7	I would be as satisfied talking to an OA patient over the internet video as I would be talking to the patient in person in my consulting room (*)	Positive attitude or needs of HCPs regarding feasibility of telehealth (1)
F8	An exercise program prescribed by a PT over the internet video would improve a patient's OA (*)	Benefits of telehealth in terms of effectiveness (1)
F9	An exercise program prescribed by a PT over the telephone would save a patient money (*)	Patient-related benefits regarding feasibility of telehealth (1)
F10	An exercise program prescribed by a PT over the internet video would save a patient money (*)	Patient-related benefits regarding feasibility of telehealth (1)
F11	I would be able to adequately monitor a patient's OA over the internet video (*)	Lack of physical/visual contact not a major issue for HCPs (1)
F12	Receiving an exercise program from a PT over the telephone would be a convenient form of health care for an OA patient (*)	Patient-related benefits regarding feasibility of telehealth (1)
F13	Receiving an exercise program from a PT over the internet video would be a convenient form of health care for an OA patient (*)	Patient-related benefits regarding feasibility of telehealth (1)
F14	Receiving an exercise program from a PT over the telephone would save the patient time (*)	Patient-related benefits regarding feasibility of telehealth (1)
F15	Receiving an exercise program from a PT over the internet video would save the patient time (*)	Patient-related benefits regarding feasibility of telehealth (1)
F16	I would be interested in being involved in a service offering PT-prescribed exercise over the internet video for my people with OA (*)	Positive attitude or needs of HCPs regarding feasibility of telehealth (1)
F17	Using the internet video would be an acceptable way for me to deliver an exercise program to patients with OA (*)	Positive attitude or needs of HCPs regarding feasibility of telehealth (1)
F18	Using the internet video would be a useful (practical) way for me to deliver an exercise program to patients with OA (*)	Positive attitude or needs of HCPs regarding feasibility of telehealth (1)
F19	Using the internet video would be an effective way for me to deliver an exercise program to patients with OA (*)	Positive attitude or needs of HCPs regarding feasibility of telehealth (1)
F20	Using the telephone would be an affordable way for patients to receive a PT-prescribed exercise program for their OA (*)	Patient-related benefits regarding feasibility of telehealth (1)
F21	Using the internet video would be an affordable way for patients to receive a PT-prescribed exercise program for their OA (*)	Patient-related benefits regarding feasibility of telehealth (1)
F22	Using the internet video would be a safe way for patients to receive a PT-prescribed exercise program for their OA (*)	Telehealth is safe for patients or patient/data privacy (1)

Reid (2014) [59]

Number	Description	Subcategory (domain)
B1	Lack of availability of physiotherapy	LIs are unavailable or inaccessible (1)
B2	Poor rate of previous (physiotherapy) success (*)	Other patient characteristics (3)
B3	There is a paucity of evidence in regards to the effectiveness of physiotherapy treatment for	LIs have little or no effect on OA (1)

	OA hip and/or knee (*)	
B4	Past experience has shown physiotherapy to be ineffective (*)	LIs have little or no effect on OA (1)
F1	Referring patients to physiotherapy if they had high levels of pain/disability and where radiographic evidence of OA was present (*)	Severity of disease and symptoms (3)
F2	Referring patients to physiotherapy if they were of a younger age (*)	Other patient characteristics (3)
F3	Good access to physiotherapy in area (*)	LIs are available or accessible, or suggestions for improvement (1)
F4	Physiotherapists do not/might not lack expertise in OA management (*)	Good interdisciplinary collaboration or healthcare provision, or suggestions for improvement (4)
F5	Conservative treatment is (perhaps) an important part of OA management (*)	Positive attitude toward LIs (2)

De Rooij (2020) [60]

Number	Description	Subcategory (domain)
B1	In my daily clinical practice I can (perhaps) not integrate working according to the protocol well (*)	LIs are not feasible or sustainable (1)
B2	The lay out of the protocol does not/might not facilitate its usage in daily practice (*)	Challenges for HCPs during delivery of LIs (1)
B3	I do not/might not treat enough patients with knee OA and comorbidity to apply the protocol (*)	LIs are not feasible or sustainable (1)
B4	The protocol does not/might not fit well with my working methods of daily clinical practice (*)	LIs are not feasible or sustainable (1)
B5	I do not/might not have sufficient knowledge about knee OA exercise therapy and comorbidity to apply the protocol in daily clinical practice (*)	Lack of knowledge or skills around LIs or promoting behavioral change (2)
B6	I do not/might not have sufficient skills to apply the protocol in daily clinical practice (*)	Lack of knowledge or skills around specific resources (2)
B7	I do not/might not read the protocol sufficiently to remember any of its contents (*)	Lack of knowledge or skills around specific resources (2)
B8	The number of treatments that the patient receives from their insurance company is a barrier in using the protocol (*)	Restrictions due to health insurance (7)
B9	The patients are not/might not be cooperative in applying the protocol in daily clinical practice (*)	Negative attitude toward LIs (3)
B10	My colleagues in physiotherapy are not/might not be cooperative in applying the protocol in daily clinical practice (*)	Non-optimal interdisciplinary collaboration or healthcare provision (4)
B11	The management of my practice is not/might not be collaborative regarding the application of the protocol in daily clinical practice (*)	Management not supportive (6)
B12	The general practitioners or other physicians are not/might not be collaborative regarding the application of the protocol in daily clinical practice (*)	Non-optimal interdisciplinary collaboration or healthcare provision (4)
B13	Suboptimal collaboration with general practitioners and physicians	Non-optimal interdisciplinary collaboration or healthcare provision (4)
B14	Referring physicians do not always believe in/may lack knowledge about effectiveness of exercise therapy	Non-optimal interdisciplinary collaboration or healthcare provision (4)
B15	Patients do not always believe in/may lack knowledge about effectiveness of exercise therapy	Low health literacy (3)
B16	Total amount of knee OA patients with comorbidity was lower than expected	LIs are not feasible or sustainable (1)

B17	Number of treatment sessions patients receive from insurance companies restricted application of the strategy	Restrictions due to health insurance (7)
B18	Patients with knee OA and comorbidity are not always motivated to perform exercises	Negative attitude toward LIs (3)
B19	Requesting medical information about patients from specialists takes a lot of time	Challenges of communication and referral procedures (4)
B20	Physicians are not always collaborating in discussing medical conditions of patients	Lack of communication between HCPs (4)
F1	The protocol is feasible in daily clinical practice (*)	LIs are feasible or sustainable (1)
F2	The protocol supports me in clinical reasoning (*)	Available resources might improve knowledge and decision-making (2)
F3	The protocol gives the opportunity to make your own decisions regarding history taking, physical examination, and treatment (*)	Ability and importance of providing personalized treatment within LIs (1)
F4	Some contents of the protocol are not/might not be incorrect (*)	Positive experiences with or suggestions to improve the content or structure of LIs (1)
F5	In my daily clinical practice, I work with sufficient equipment (including blood pressure meter, saturation meter) to properly apply the protocol (*)	LIs are feasible or sustainable (1)
F6	The protocol is supporting the improvement of my knowledge regarding knee OA exercise therapy and comorbidity (*)	Available resources might improve knowledge and decision-making (2)
F7	The recommendations over adapting the diagnostic phase (history taking and physical examination) in the protocol are clear and understandable (*)	Ease for HCPs during delivery of LIs (1)
F8	The recommendations over adapting the OA exercise therapy in the protocol are clear and understandable (*)	Ease for HCPs during delivery of LIs (1)
F9	The protocol is supportive in which comorbidity-related symptoms I need to monitor before, during and after treatment (*)	Available resources might improve knowledge and decision-making (2)
F10	Working with the protocol invites me to discuss more with experts in the field of the comorbidity (*)	Good interdisciplinary collaboration or healthcare provision, or suggestions for improvement (4)
F11	In general, I do not/might not feel resistance towards working according to protocols (*)	Positive attitude toward guidelines or protocols (2)
F12	I have changed my working method (due to the protocol) (*)	LIs are feasible or sustainable (1)
F13	Working according to the protocol is not/might not be too time-consuming (*)	Adequate duration of specific interventions or protocols (5)
F14	Working according to the protocol should be financially rewarded (*)	Financial reward for implementing LIs (5)
F15	The protocol is applicable to OA patients with comorbidity that I see in my clinical practice (*)	Positive experiences with or suggestions to improve the content or structure of LIs (1)
F16	Intake procedure is feasible and implementable	LIs are feasible or sustainable (1)
F17	Important to extend the intake phase to at least to 45 min	Positive experiences with or suggestions to improve the content or structure of LIs (1)
F18	The more you apply the strategy in daily practice, the easier it is to integrate it in your daily working method	LIs are feasible or sustainable (1)
F19	More insight into exercise tolerance/more background knowledge to make clinical decision by using strategy	Available resources might improve knowledge and decision-making (2)
F20	Inform referrers better about benefits of exercise therapy in patients with knee OA and	Good interdisciplinary collaboration or healthcare provision, or

	comorbidity	suggestions for improvement (4)
F21	Inform patients with knee OA and comorbidity better about benefits of exercise therapy	High health literacy or importance of education (3)
F22	Optimize collaboration with orthopaedic surgeons and other health care providers	Good interdisciplinary collaboration or healthcare provision, or suggestions for improvement (4)
F23	In complex patients insurance companies should reimburse more treatment sessions	Benefits of good health insurance (7)
F24	Useful to plan follow up/refreshment training to repeat/discuss content of course/protocol and application	Ease for HCPs during delivery of LIs (1)
F25	Shortening the protocol would increase user-friendliness	Ease for HCPs during delivery of LIs (1)

Holden (2009) [61]

Number	Description	Subcategory (domain)
B1	Biomedical perspective on knee OA, attributing signs and symptoms to local knee pathology or wear and tear	OA seen as untreatable and local condition (wear-and-tear) (9)
B2	OA seen as chronic degenerative condition that would progressively worsen over time (only cure being surgery)	OA seen as untreatable and local condition (wear-and-tear) (9)
B3	Effectiveness related to severity of joint damage/pain level (less effective when more damage/pain)	Severity of disease and symptoms (3)
B4	Fear of increasing symptoms (as barrier to prescribing exercise)	LIs are unsafe or have negative effects (1)
B5	Causing disease progression, particularly through weight-bearing activities (as barrier to prescribing exercise)	LIs are unsafe or have negative effects (1)
B6	Exacerbating patient's comorbidities (as barrier to prescribing exercise)	LIs are unsafe or have negative effects (1)
B7	Reluctant to promote exercise in the presence of pain	Severity of disease and symptoms (3)
B8	Negative perceptions of patients' levels of exercise adherence	Low patient adherence or engagement (3)
B9	Lack of motivation or laziness (as patient-centered barrier to adherence)	Negative attitude toward LIs (3)
B10	Human nature (as patient-centered barrier to adherence)	Potential effects of LIs are difficult to accomplish (1)
B11	Pain (as patient-centered barrier to adherence)	Severity of disease and symptoms (3)
B12	Fear of harm (as patient-centered barrier to adherence)	Low health literacy (3)
B13	Negative treatment expectations (as patient-centered barrier to adherence)	Negative attitude toward LIs (3)
B14	Therapist's role seen as assessment/exercise prescription/education (relatively short-term responsibilities)	Perception of own role potentially impeding prescription or follow-up of LIs (2)
B15	Patient's role to follow prescribed exercise program over long term/get on board with treatment	Perception of own role potentially impeding prescription or follow-up of LIs (2)
B16	Limited time to review individual patients reduced opportunities to facilitate behavior change	Lack of time within patient consultations (5)
B17	Large caseloads and pressure of waiting lists reduced the number of treatment sessions provided	Lack of time within patient consultations (5)
B18	Limited opportunity to provide follow-up sessions after discharge	Lack of time within patient consultations (5)
B19	Poor links to community facilities such as local leisure centres	LIs are unavailable or inaccessible (1)

B20	Gaps in knowledge/skills (including how to facilitate behavior change, particularly with less motivated patients)	Lack of knowledge or skills around LIs or promoting behavioral change (2)
B21	Exercises are not/might not be effective for patients if an X-ray shows severe knee osteoarthritis (*)	Severity of disease and symptoms (3)
B22	Increasing overall activity levels does not/might not stop the knee problem getting worse (*)	LIs have little or no effect on OA (1)
B23	Increasing the strength of the muscles around the knee does not/might not stop the knee problem getting worse (*)	LIs have little or no effect on OA (1)
B24	General exercise is not/might not be safe for everybody to do (*)	LIs are unsafe or have negative effects (1)
B25	Local strengthening exercises for the knee are not/might not be safe for everybody to do (*)	LIs are unsafe or have negative effects (1)
B26	Knee problems are not/might not be improved by general exercise (*)	LIs have little or no effect on OA (1)
B27	Physical therapists should (perhaps) not prescribe general exercise for every patient with knee OA (*)	Perception of own role potentially impeding prescription or follow-up of LIs (2)
B28	It is the patient's own responsibility to continue doing their exercise program (*)	Perception of own role potentially impeding prescription or follow-up of LIs (2)
B29	It is not/might not be the physiotherapist's responsibility to make sure that the patient will continue doing their exercise program (*)	Perception of own role potentially impeding prescription or follow-up of LIs (2)
B30	It is not/might not be important that people with knee OA increase their overall activity levels (*)	Negative attitude toward LIs (2)
F1	Effectiveness related to severity of joint damage/pain level (more effective when less damage/pain)	Severity of disease and symptoms (3)
F2	Importance of exercise adherence/link between level of adherence and clinical outcomes (dose-response effect)	Importance of high patient adherence or engagement for effectiveness of LIs (3)
F3	Recognizing potential influence on exercise adherence, sharing responsibility of exercise adherence with patient	Perception of own role potentially stimulating prescription or follow-up of LIs (2)
F4	Exercises are effective for patients if an X-ray shows moderate knee osteoarthritis (*)	Severity of disease and symptoms (3)
F5	Exercises are effective for patients if an X-ray shows mild knee osteoarthritis (*)	Severity of disease and symptoms (3)
F6	Knee problems are improved by local strengthening exercises (*)	LIs have positive effects on affected joint(s) (1)
F7	Physical therapists should prescribe local strengthening exercise for every patient with knee OA (*)	Perception of own role potentially stimulating prescription or follow-up of LIs (2)
F8	How well a patient complies with their exercise program determines how effective it will be (*)	Importance of high patient adherence or engagement for effectiveness of LIs (3)
F9	Physiotherapists should educate chronic patients with knee OA about how to change their lifestyle for the better (*)	Perception of own role potentially stimulating prescription or follow-up of LIs (2)
F10	A standard set of exercises is not/might not be sufficient for every patient with knee OA (*)	Ability and importance of providing personalized treatment within LIs (1)
F11	Exercise for knee OA is most beneficial when it is tailored to meet individual patient needs (*)	Ability and importance of providing personalized treatment within LIs (1)
U1	Exercise does not/might not work just as well for everybody, regardless of the amount of pain they have (*)	Severity of disease and symptoms (3)

Kloek (2020) [62]

Number	Description	Subcategory (domain)
B1	Less satisfied about the applicability of e-Exercise for only one diagnosis	Insufficient ability to provide personalized treatment within LIs (1)
B2	Less satisfied about time needed to get used to e-Exercise during high work pressure/administrative burden	Lack of time due to other demands (or not further specified) (5)
B3	Lack of technology affinity (reason for patients' non-willingness to participate in e-Exercise)	Patient-related challenges regarding feasibility of telehealth (1)
B4	Patients preferred regular face-to-face contact	Patient-related challenges regarding feasibility of telehealth (1)
B5	Perceiving web-based application as an additional burden	Lack of time due to other demands (or not further specified) (5)
B6	Technical skills (lack of)	Other challenges for HCPs regarding feasibility of telehealth (1)
B7	Clarity of instruction manual and course (lack of)	Challenges for HCPs during delivery of LIs (1)
B8	Adaptive capacity to change treatment routines (lack of)	Other challenges for HCPs regarding feasibility of telehealth (1)
B9	Busy work schedules and administrative burden hindered testing/using e-Exercise in their practice	Lack of time due to other demands (or not further specified) (5)
B10	Reduced face-to-face contact interfered with professional autonomy	Challenges for HCPs regarding lack of physical/visual contact (1)
B11	Absence of national e-Health guideline or standard	Other challenges for HCPs regarding feasibility of telehealth (1)
B12	Loss of income due to substitution of face-to-face session	Other challenges for HCPs regarding feasibility of telehealth (1)
B13	E-Exercise does not/might not contain all essential elements for the treatment of hip/knee OA (*)	Non-optimal content or structure of LIs (1)
B14	I do not/might not have enough influence on the content of patients' individual e-Exercise program (*)	Insufficient ability to provide personalized treatment within LIs (1)
B15	The content of e-Exercise is not/might not be aligned with my opinion about treating patients with OA (*)	Non-optimal content or structure of LIs (1)
B16	The intervention provided through e-Exercise is not/might not be appropriate for the average patient with OA (*)	Non-optimal content or structure of LIs (1)
B17	I do not/might not experience that e-Exercise supports patients in doing their exercises at home (*)	Disadvantages of telehealth in terms of effectiveness (1)
B18	Patients who were treated with e-Exercise were (perhaps) not generally positive about the intervention (*)	Non-optimal content or structure of LIs (1)
F1	More flexibility in web-based application (intervention duration, number of sets/repetitions, type of exercises)	Ability and importance of providing personalized treatment within LIs (1)
F2	Completeness of web-based application (exercises/assignments/information)	Positive experiences with or suggestions to improve the content or structure of LIs (1)
F3	Perception that e-Exercise is an appropriate treatment option for subgroup of OA patients	Positive experiences with or suggestions to improve the content or structure of LIs (1)
F4	Added value in terms of exercise adherence (important factor to use web-based	Benefits of telehealth in terms of effectiveness (1)

	application)	
F5	Perceiving web-based application as time-saving	Adequate duration of specific interventions or protocols (5)
F6	More flexibility in intervention (more possibilities to personalize to individual needs)	Ability and importance of providing personalized treatment within LIs (1)
F7	Support from colleagues	Good interdisciplinary collaboration or healthcare provision, or suggestions for improvement (4)
F8	Advantage of reducing number of treatments	Positive attitude or needs of HCPs regarding feasibility of telehealth (1)
F9	Offering an innovative intervention attracted new patients	Positive attitude or needs of HCPs regarding feasibility of telehealth (1)
F10	The instruction course and manual assisted me so that I knew how to work with e-Exercise (*)	Ease for HCPs during delivery of LIs (1)
F11	That it results in less income is not/might not be a major disadvantage of e-Exercise (*)	Positive attitude or needs of HCPs regarding feasibility of telehealth (1)
F12	Our physiotherapy practice has the intention to use e-Health innovations (*)	Positive attitude or needs of HCPs regarding feasibility of telehealth (1)
F13	I do not/might not have insufficient time available to get familiar with e-Exercise and to use the web-application (*)	Adequate duration of specific interventions or protocols (5)
F14	I believe that patient data gathered at the e-Exercise web-application is stored safely (*)	Telehealth is safe for patients or patient/data privacy (1)
