# Supplementary material for: Barriers and facilitators perceived by healthcare professionals for implementing lifestyle interventions in patients with osteoarthritis: a scoping review
Source: BMJ Open. 2022 Feb 1;12(2):e056831. doi: 10.1136/bmjopen-2021-056831 (PMC8808449; doi:10.1136/bmjopen-2021-056831)
Supplement: Author's manuscript [file bmjopen-2021-056831.draft_revisions.pdf]

BMJ Open is committed to open peer review. As part of this commitment we make the peer review history of every article we publish publicly available.

When an article is published we post the peer reviewers' comments and the authors' responses online. We also post the versions of the paper that were used during peer review. These are the versions that the peer review comments apply to.

The versions of the paper that follow are the versions that were submitted during the peer review process. They are not the versions of record or the final published versions. They should not be cited or distributed as the published version of this manuscript.

BMJ Open is an open access journal and the full, final, typeset and author-corrected version of record of the manuscript is available on our site with no access controls, subscription charges or pay-per-view fees (http://bmjopen.bmj.com).

If you have any questions on BMJ Open's open peer review process please email info.bmjopen@bmj.com

BMJ Open

Barriers and facilitators perceived by healthcare professionals for implementing lifestyle interventions in patients with osteoarthritis: a systematic scoping review

Journal:	*BMJ Open*
Manuscript ID	bmjopen-2021-056831
Article Type:	Original research
Date Submitted by the Author:	30-Aug-2021
Complete List of Authors:	Bouma, Sjoukje; University of Groningen, University Medical Center Groningen, Department of Orthopedics<br>van Beek, Juliette; University of Groningen, University Medical Center Groningen, Department of Orthopedics; University of Groningen, University Medical Center Groningen, Center for Human Movement Sciences<br>Diercks, Ron; University of Groningen, University Medical Center Groningen, Department of Orthopedics<br>van der Woude, Lucas; University of Groningen, University Medical Center Groningen, Center for Human Movement Sciences; University of Groningen, University Medical Center Groningen, Department of Rehabilitation Medicine<br>Stevens, Martin; University of Groningen, University Medical Center Groningen, Department of Orthopedics<br>van den Akker-Scheek, Inge; University of Groningen, University Medical Center Groningen, Department of Orthopedics
Keywords:	ORTHOPAEDIC & TRAUMA SURGERY, Hip < ORTHOPAEDIC & TRAUMA SURGERY, Knee < ORTHOPAEDIC & TRAUMA SURGERY, Musculoskeletal disorders < ORTHOPAEDIC & TRAUMA SURGERY, REHABILITATION MEDICINE, SPORTS MEDICINE

SCHOLARONE™
Manuscripts

I, the Submitting Author has the right to grant and does grant on behalf of all authors of the Work (as defined in the below author licence), an exclusive licence and/or a non-exclusive licence for contributions from authors who are: i) UK Crown employees; ii) where BMJ has agreed a CC-BY licence shall apply, and/or iii) in accordance with the terms applicable for US Federal Government officers or employees acting as part of their official duties; on a worldwide, perpetual, irrevocable, royalty-free basis to BMJ Publishing Group Ltd ("BMJ") its licensees and where the relevant Journal is co-owned by BMJ to the co-owners of the Journal, to publish the Work in this journal and any other BMJ products and to exploit all rights, as set out in our licence.

The Submitting Author accepts and understands that any supply made under these terms is made by BMJ to the Submitting Author unless you are acting as an employee on behalf of your employer or a postgraduate student of an affiliated institution which is paying any applicable article publishing charge ("APC") for Open Access articles. Where the Submitting Author wishes to make the Work available on an Open Access basis (and intends to pay the relevant APC), the terms of reuse of such Open Access shall be governed by a Creative Commons licence – details of these licences and which Creative Commons licence will apply to this Work are set out in our licence referred to above.

Other than as permitted in any relevant BMJ Author's Self Archiving Policies, I confirm this Work has not been accepted for publication elsewhere, is not being considered for publication elsewhere and does not duplicate material already published. I confirm all authors consent to publication of this Work and authorise the granting of this licence.

For peer review only

Title: "Barriers and facilitators perceived by healthcare professionals for implementing lifestyle interventions in patients with osteoarthritis: a systematic scoping review"

S.E. (Sjoukje) Bouma[1], J.F.E. (Juliette) van Beek[1,2], R.L. (Ron) Diercks[1], L.H.V. (Lucas) van der Woude[2,3,4], M. (Martin) Stevens[1*], I. (Inge) van den Akker-Scheek[1*]

[1] University of Groningen, University Medical Center Groningen, Department of Orthopedics, Groningen, The Netherlands

[2] University of Groningen, University Medical Center Groningen, Center for Human Movement Sciences, Groningen, The Netherlands

[3] University of Groningen, University Medical Center Groningen, Department of Rehabilitation Medicine, Groningen, The Netherlands

[4] Peter Harrison Centre for Disability Sport, School of Sport, Exercise and Health Sciences, Loughborough University, United Kingdom

[*] These authors contributed equally to this work.

Corresponding author:

S.E. (Sjoukje) Bouma

University of Groningen, University Medical Center Groningen

Department of Orthopedics (BB51)

PO Box 30.001

9700 RB Groningen, The Netherlands

E-mail: s.e.bouma@umcg.nl

For peer review only - http://bmjopen.bmj.com/site/about/guidelines.xhtml

Abstract

Objective: To provide an overview of barriers and facilitators that healthcare professionals (HCPs) perceive regarding the implementation of lifestyle interventions (LIs) in patients with hip and/or knee osteoarthritis (OA).

Design: Systematic scoping review.

Methods: The databases PubMed, Embase, CINAHL, PsycINFO, and the Cochrane Library were searched up to January 2021. Primary research articles with a quantitative, qualitative or mixed-methods design were eligible for inclusion if they reported: (1) perceptions of primary and/or secondary HCPs (*population*); (2) on implementing LIs with physical activity and/or weight management as key components (*concept*); (3) on conservative management of hip and/or knee OA (*context*). Barriers and facilitators were extracted by two researchers independently and linked to a framework based on the Tailored Implementation for Chronic Diseases checklist.

Results: Thirty-six articles were included. In total, 809 factors were extracted and subdivided into nine domains: intervention factors (n=315); individual HCP factors (n=144); patient factors (n=137); professional interactions (n=101); incentives and resources (n=56); capacity for organizational change (n=7); social, political and legal factors (n=9); patient and HCP interactions (n=19); and disease factors (n=21).

Conclusions: Multiple individual and environmental factors influence the implementation of LIs by HCPs in patients with hip and/or knee OA. The resulting overview of barriers and facilitators can guide future research on the implementation of LIs within OA care. To investigate whether factor frequency is related to the relevance of each domain, further research should assess the relative importance of the identified factors involving all relevant disciplines of primary and secondary HCPs.

PROSPERO registration number: CRD42019129348.

For peer review only

For peer review only - http://bmjopen.bmj.com/site/about/guidelines.xhtml

Abstract word count: 251 (max. 300)

Manuscript word count: 4176 (max. 4000)

Strengths and limitations of this study

- This is the first systematic scoping review to classify barriers and facilitators for implementing lifestyle interventions as conservative treatment for hip and/or knee osteoarthritis from the perspective of primary and secondary healthcare professionals.
- Both qualitative and quantitative data were included, providing broad insight into the topic.
- Given the broad definition of "implementing lifestyle interventions", the identified barriers and facilitators provide insight into the full spectrum of influencing factors rather than being applicable to every single way of implementing lifestyle interventions.
- Grey literature was not included in the search and selection process.

For peer review only

For peer review only - http://bmjopen.bmj.com/site/about/guidelines.xhtml

Introduction

Regular physical activity and weight management are recommended by national and international clinical guidelines for the conservative management of osteoarthritis (OA)[1-5]. Previous studies have demonstrated that lifestyle interventions (LIs) focusing on exercise, alone or combined with dietary weight loss, are able to reduce OA-related disability and to postpone or even prevent total joint arthroplasty[6-10]. However, these positive results are not always transferred from research settings to daily practice, which means that LIs are underutilized[11]. This suboptimal implementation of LIs as treatment for OA can result from factors related to the patient, the healthcare professional (HCP) or the societal context[12]. Research on adhering to LIs has so far focused mainly on identifying barriers and facilitators at the patient level. However, these studies have also shown that HCPs can have a facilitating role in the lifestyle behavior of their patients, for example by providing advice, education, encouragement, and instructions[13,14].

Some research has already been conducted investigating the perspective of HCPs and the implementation of LIs in their daily practice. This knowledge is needed in order to enhance the implementation of LIs. As far as the authors know, no (systematic) literature review has previously been performed that identified and/or classified barriers and facilitators for implementing LIs in the conservative treatment of OA from the perspective of HCPs. One systematic review focused on the views toward OA management based on recommendations in clinical practice guidelines of HCPs working in primary care[15]. However, HCPs working in secondary care are also involved in the treatment of patients with OA, which draws attention to the importance of collaboration and communication between primary and secondary care practitioners[16].

For peer review only - http://bmjopen.bmj.com/site/about/guidelines.xhtml

A scoping review can be considered a suitable methodology to summarize existing literature on barriers and facilitators for implementing LIs in OA and to identify potential gaps in the current literature on participation of primary and secondary HCPs[17]. To this end, a systematic scoping review was conducted aiming to provide a comprehensive overview of barriers and facilitators perceived by primary and secondary HCPs regarding the implementation of LIs in patients with hip and/or knee OA. The Tailored Implementation for Chronic Diseases (TICD) checklist was used to guide data synthesis[18].

Method

Study design

We conducted this systematic scoping review according to the framework developed by Arksey and O'Malley[19]. Five stages were followed successively: (1) identifying the research question; (2) identifying relevant studies; (3) study selection; (4) charting the data; and (5) collating, summarizing and reporting the results[19]. The PRISMA Extension for Scoping Reviews (PRISMA-ScR) checklist was used as reporting guideline[20]. The review protocol was registered in the PROSPERO database (registration number: CRD42019129348).

Data sources and searches

A search strategy was developed consisting of four components: search terms related to (1) primary and secondary HCPs; (2) hip and/or knee OA; (3) LIs; and (4) barriers and facilitators. This search strategy was applied in five bibliographic electronic databases (i.e. PubMed, Embase, CINAHL, PsycINFO, and the Cochrane Library) to identify relevant articles up to 19 January 2021. A detailed search strategy for each of the databases can be

For peer review only - http://bmjopen.bmj.com/site/about/guidelines.xhtml

found in Supplemental File 1. Reference lists of included articles were manually searched for additional relevant articles. Primary research articles with a quantitative, qualitative or mixed-methods design were eligible for inclusion; study protocols, reviews, abstracts, and commentaries were excluded. Articles written in English, German, or Dutch were eligible for inclusion. No restrictions were applied regarding publication period.

Study selection

Eligibility criteria were described according to the Population-Concept-Context framework[21]. First, the study population was defined as all primary and secondary HCPs who are involved in the conservative treatment of patients with hip and/or knee OA. Articles focusing solely on the perspective of patients with hip and/or knee OA were excluded. Second, the concepts central to this review were barriers and facilitators for implementing LIs. Barriers and facilitators were defined as any belief, experience, factor, opinion, reason, or view reported by an HCP that potentially influences (either impedes or facilitates) implementation of LIs in patients with hip and/or knee OA. Implementing LIs was broadly defined, ranging from mentioning or discussing a healthy lifestyle to recommending or running specific lifestyle programs, as long as it was clearly described that physical activity and/or weight management were key components. This definition includes physiotherapeutic exercise interventions (aerobic, functional, or strengthening programs), dietary interventions, and self-management programs. Physiotherapeutic modalities such as acupuncture, manual therapy, and massage, and self-management programs whose content was not specified were not considered LIs and were therefore excluded. Articles not primarily focusing on implementing LIs (e.g. development and evaluation of clinical guidelines, general management of hip and/or knee OA, general patient-practitioner relationship or shared decision-making) also fell outside the scope of this review. Lastly, the context of this review was the conservative treatment of hip

For peer review only - http://bmjopen.bmj.com/site/about/guidelines.xhtml

and/or knee OA in both primary and secondary healthcare settings. Articles focusing on preoperative or postoperative treatment of hip and/or knee OA were excluded. Two researchers (SB together with AJ or JvB) independently assessed the eligibility of the identified articles based on the above criteria in three consecutive rounds: based on (1) title; (2) abstract; and (3) full-text of the article. Any disagreements among the researchers were resolved in consensus meetings.

Data extraction and quality assessment

A data extraction form was created and pilot-tested in order to systematically record study characteristics (first author, year of publication, country of origin, aims/purpose, study design, data collection method, data analysis method, theoretical basis, study population, setting, recruitment method, type of LI, patient population) and outcomes (barriers, facilitators, and/or unclear factors (i.e. an influencing factor, but not clearly defined as barrier or facilitator)). Study quality was assessed with the Mixed Methods Appraisal Tool (MMAT). The MMAT is a critical appraisal tool that can be used in reviews of mixed studies to assess the methodological quality of different study design categories: mixed-methods, qualitative, and quantitative studies (randomized controlled trials, non-randomized studies, and descriptive studies)[22,23]. Since calculating a total score is discouraged[23], it was chosen to present the ratings of the individual criteria.

Data extraction was performed in two stages. The first stage consisted of filling in the data extraction form and the MMAT for each article, done by two researchers (SB/JvB) independently. Regarding barriers and facilitators, both researchers extracted the relevant units of text and/or descriptive statistics from the Results sections. Any discrepancies between the researchers in this first stage were resolved in consensus meetings. During the second

For peer review only - http://bmjopen.bmj.com/site/about/guidelines.xhtml

stage, the extraction of barriers and facilitators was discussed among the research team (SB/MS/IvdAS) and the process was further refined for both quantitative and qualitative data. Regarding quantitative data, factors were only extracted if ≥50% of participants indicated that the factor influenced the implementation of LIs[24,25]. For close-ended questions or attitude statements with multiple answer options, participants were classified as being "in agreement" or "not in agreement". If this classification had not yet been made by the authors of the original article, it was made based on the possible answer options, with "(strongly) agree", "to a reasonable/large extent" and "yes" indicating agreement, and "neither disagree or agree", "don't know", "neutral", "a little bit/not at all", "(strongly) disagree", and "no" indicating not in agreement. Next, the factor was classified as barrier or facilitator depending on the formulation of the question and which of the two groups ("in agreement" versus "not in agreement") comprised ≥50% of the participants. In case of open-ended questions, all mentioned factors were extracted. Regarding qualitative data, if the authors of the original study did not explicitly identify a factor as barrier or facilitator, the description in the text or the participants' quotes were used to classify the factor as barrier (i.e. impeding/negative/problem/lack), facilitator (i.e. facilitating/positive/solution/need), or unclear (i.e. insufficient information). In addition, all unclear factors were re-discussed with a third researcher (IvdAS) to assess whether these factors could nevertheless be classified as barrier or facilitator. At the end of the second stage, final data extraction based on the above criteria was performed by one researcher (SB), who also checked the consistency of the entire data extraction process.

Data synthesis and analysis

A narrative synthesis of the data was undertaken, based on the TICD checklist developed by Flottorp et al.[18]. This checklist aims to assist in identifying key determinants of professional

For peer review only - http://bmjopen.bmj.com/site/about/guidelines.xhtml

practice, defined as factors that might prevent or enable healthcare improvements, and is intended for use in research on implementation and quality improvement in healthcare. It consists of seven domains: (1) guideline factors; (2) individual health professional factors; (3) patient factors; (4) professional interactions; (5) incentives and resources; (6) capacity for organizational change; and (7) social, political, and legal factors. The authors of the current study have previously used the TICD checklist in the analysis of focus group data on the same topic, revealing two additional domains: (8) patient and HCP interactions; and (9) disease factors (data not yet published). One researcher (SB) assigned all extracted factors to one of these nine domains and then summarized the factors within different categories and subcategories per domain. The resulting classification of factors and corresponding conclusions were subsequently discussed among the research team (SB/MS/IvdAS).

Patient and public involvement

Patients or the public were not involved in this study as the study aim did not concern patients but HCPs.

Results

Study selection

A flowchart of the study selection process is presented in Figure 1. A total of 8338 articles were retrieved. After removal of duplicates and exclusion of articles based on title or abstract, 93 potentially relevant articles remained for full-text screening. Ultimately, 36 articles were included in the qualitative synthesis[26-61].

[**Figure 1.** Flowchart of the study selection process]

For peer review only - http://bmjopen.bmj.com/site/about/guidelines.xhtml

Study characteristics

General characteristics of the included studies are presented in Table 1. The majority of studies were conducted in Australia (36%), the Netherlands (19%), the United Kingdom (19%) and Canada (11%). Qualitative data were extracted in 26 studies (72%), quantitative data in 7 studies (19%), and both qualitative and quantitative data in the remaining 3 studies (8%). Individual interviews were most commonly used as qualitative data collection method, while the quantitative studies were all based on cross-sectional surveys. Most studies included physiotherapists or general practitioners (or physicians) as study population. Other participants were dieticians, exercise professionals, a nurse practitioner, an occupational therapist, orthopedic surgeons, practice nurses, program instructors, rheumatologists, telephone coaches, and triaging clinicians.

[Table 1 near here]

Quality assessment

Findings of the quality assessment of the included studies based on the MMAT are shown in Supplemental File 2. Regarding the qualitative data assessments, only one study had the maximum of five positive ratings. Seven studies had a negative rating for the item on substantiating the interpretation of results, as no or a limited number of participant quotes were presented. In addition, many unknown ratings were given due to a lack of information about the applied qualitative approach and/or data analysis methods and their rationale. Regarding the quantitative data assessments, most studies had a negative or unknown rating for the risk of non-response bias due to low response rates or a lack of information about the response rate and/or reasons for non-response. In addition, the item on representativeness of

For peer review only - http://bmjopen.bmj.com/site/about/guidelines.xhtml

the sample was often given an unknown rating because insufficient information about the sample and/or non-responders was presented. Finally, all three mixed-methods studies had a negative rating since the qualitative and quantitative components did not adhere to their specific quality criteria. For the other four mixed-methods criteria, only one of these three studies obtained positive ratings.

Synthesis of results

A total of 809 factors were extracted from the 36 included articles. Table 2 presents the distribution of factors from the individual studies across the aforementioned nine domains, which were largely based on the TICD checklist. The highest number of factors was assigned to intervention factors (n=315), followed by individual HCP factors (n=144), and patient factors (n=137). The lowest number of factors was assigned to capacity for organizational change (n=7), followed by social, political, and legal factors (n=9), and patient and HCP interactions (n=19). In Table 3 the content of the nine domains is further explained by presenting an overview of the created categories and subcategories of factors that potentially influence the implementation of LIs by HCPs within each domain. In addition, each domain is briefly discussed below. A full overview of all extracted factors can be found in Supplemental File 3 (presented per domain) and Supplemental File 4 (presented per article).

[Table 2 near here]

[Table 3 near here]

Domain 1: Intervention factors

This domain consists of factors related to the outcomes, design and accessibility of LIs. Regarding outcomes, perceptions that LIs have no or even negative effects were identified as

For peer review only - http://bmjopen.bmj.com/site/about/guidelines.xhtml

barriers. By contrast, other HCPs reported that LIs have positive effects on multiple levels and are safe to implement. Regarding design, barriers and facilitators were identified for the content or structure of LIs, the effort required for patients participating in LIs, and HCPs delivering LIs. The inability and ability to provide personalized treatment within LIs were identified as barrier and facilitator, respectively. Regarding accessibility, barriers and facilitators were found in the field of availability, associated costs, feasibility and sustainability, and convenience for patients. A separate category was created for factors specific to delivering LIs via telehealth, consisting of factors related to effectiveness, safety, feasibility for HCPs and patients, and the interaction between HCPs and patients.

Domain 2: Individual HCP factors

Within this domain, three categories related to individual primary and secondary HCPs were established: expertise, attitude, and role. A lack of knowledge and skills, a negative attitude toward LIs or guidelines/protocols, and "negative" perceptions about the HCP's own role in the implementation of LIs (e.g. no/limited role) were found to be barriers. Adequate knowledge and skills, a positive attitude toward LIs or guidelines/protocols, and "positive" perceptions about the HCP's own role (e.g. active role in the prescription or follow-up of LIs) were identified as facilitators.

Domain 3: Patient factors

This domain includes factors related to patients with hip and/or knee OA as perceived by HCPs. The first category within this domain is health status, consisting of the influence of OA severity, potential comorbidities, and other patient characteristics. The second category is treatment expectations and preferences, in which the perception that patients are not open to LIs was mainly seen as barrier. The third category concerns active participation, which refers

For peer review only - http://bmjopen.bmj.com/site/about/guidelines.xhtml

to degree of patient adherence or engagement and its influence on the effectiveness of LIs. The fourth and last category within this domain is capabilities, referring to the capacity and possibilities of patients to change their lifestyle, which might depend on their level of health literacy, financial resources, social support, and other responsibilities.

Domain 4: Professional interactions

Factors related to interactions between primary and secondary HCPs are included in this domain, divided into two categories: collaboration, and communication and referral. Regarding collaboration, non-optimal interdisciplinary collaboration or healthcare provision and no access to other HCPs were identified as barriers, while good collaboration with and adequate access to other HCPs were found to be facilitators. With regard to communication and referral, both the quantity (i.e. amount of communication) and quality (i.e. procedures used for communication and referral) were established as influencing factors.

Domain 5: Incentives and resources

This domain consists of factors related to the availability of incentives and resources for primary and secondary HCPs. First, the amount of time was identified as influencing factor, including both time for patient consultations and other activities/demands. In addition, barriers and facilitators were found related to financial resources and information resources. Lastly, the influence of facilities such as information technology or a health center was reported.

Domain 6: Capacity for organizational change

This domain includes factors related to the organization where primary and secondary HCPs work, both in the narrow sense (i.e. specific healthcare institution) and the broad sense (i.e.

For peer review only - http://bmjopen.bmj.com/site/about/guidelines.xhtml

general professional group). Two categories were identified including only facilitators: professional paradigm and monitoring. The third category consists of solely one barrier, namely lack of support from the management of the organization.

Domain 7: Social, political, and legal factors

Within this domain, which includes factors related to the social, political, and legal context, one category was found: the healthcare system. Health insurance could be perceived as both barrier and facilitator, depending on the associated possibilities for reimbursement and access to services. Subsidies provided by the government were identified as facilitator.

Domain 8: Patient and HCP interactions

Two categories were established in this domain of interactions between patients with hip and/or knee OA and primary and secondary HCPs. First, therapeutic alliance refers to the potential negative influence of implementing LIs to the relationship between patients and HCPs as barrier, and to the importance of communication and having a good relationship as facilitator. Second, lifestyle as conversation topic includes both barriers and facilitators in relation to discussing weight management.

Domain 9: Disease factors

This final domain consists of factors related to the disease of OA, grouped in a category named image. Barriers within this category were perceptions that OA has a low priority and that it is an untreatable and local condition, often described as "wear and tear". An optimistic attitude toward the prognosis of OA and the conservative treatment options was identified as facilitator.

For peer review only - http://bmjopen.bmj.com/site/about/guidelines.xhtml

Discussion

The aim of this review was to provide an overview of barriers and facilitators that primary and secondary HCPs perceive for implementing LIs in patients with hip and/or knee OA. By linking the identified factors to a framework that was largely based on the TICD checklist[18], a comprehensive overview of influencing factors was created that could serve as a basis for improving the implementation of LIs within primary and secondary OA care. The variety of domains within this framework shows that multiple levels (i.e. both the level of the individual HCP and several environmental levels) should be considered in order to achieve this.

A relatively large number of studies were included, a majority of which was published in recent years. From these 36 studies, a total of 809 influencing factors were extracted. Although all nine domains were covered, the total number of factors identified within each domain differed greatly, ranging from 7 *(capacity for organizational change)* to 315 *(intervention factors)*. In addition to the domain of intervention factors, most factors were assigned to the domains of individual HCP factors and patient factors. However, the fact that we found the highest number of factors within these domains does not necessarily mean that these are the most important or relevant domains. It could also be an indication that studies to date have mainly focused on these domains, and that the other domains are still underexposed in the available literature. The quality assessment of the included studies showed many unknown ratings due to a lack of information about, for example, the applied methods and their rationale. This finding does not have to mean that the studies are of low quality, but it does emphasize the importance of accurate and complete reporting of research using design-specific reporting guidelines.

For peer review only - http://bmjopen.bmj.com/site/about/guidelines.xhtml

Our results reflect those of a previous systematic review conducted by Egerton et al.[15], in which the authors synthesized qualitative evidence only on primary care clinicians' views on providing recommended management of OA up to August 2016. In addition to exercise and weight loss, recommended management included education, self-management support, and medication. The authors identified four barriers as main themes: (1) "OA is not that serious"; (2) "clinicians are, or perceive they are, underprepared"; (3) "personal beliefs at odds with providing recommended practice"; and (4) "dissonant patient expectations". A few system-related factors (e.g. time, payment system) were mentioned, but these were not found to be themes across multiple studies. In the current review, on the other hand, factors related to interdisciplinary collaboration and the organizational and societal context were in fact identified. Although these domains were relatively small in terms of number of factors, the current review shows that these factors can also influence the implementation of LIs and thus offers an even broader perspective on the implementation status of LIs within OA care.

In addition to summarizing the existing literature on barriers and facilitators for implementing LIs, this review aimed to identify potential gaps in literature on the participation of HCPs. Although we aimed to include perceptions of various primary and secondary HCPs, the results show that studies to date have mainly focused on the views of physiotherapists and general practitioners. These primary HCPs may well be the first point of contact for patients within the care pathway, yet we recommend that other relevant disciplines − like dieticians, lifestyle counselors, practice nurses, and orthopedic clinicians − be more involved in follow-up research, allowing for a more complete understanding of the patient journey in OA care.

The resulting overview of barriers and facilitators can be used to improve the implementation of LIs in daily practice. This overview presents factors that are relevant for individual HCPs,

For peer review only - http://bmjopen.bmj.com/site/about/guidelines.xhtml

as well as for policymakers, who can facilitate the organizational and societal context in which primary and secondary HCPs work. When developing implementation strategies, possible interactions between the various domains should also be considered. For instance, more time *(domain 5)* can be used in various ways by HCPs: for their own education *(domain 2)*, provision of information to patients *(domain 3),* or interdisciplinary consultation *(domain 4)*. Another example is that societal changes in health insurance or payment structures *(domain 7)* can lead to increased accessibility of LIs *(domain 1)*, and that limited financial resources might be less of an obstacle for patients *(domain 3)*. Hence changes related to the established factors can have positive effects on multiple levels.

Within the domain of intervention factors, a separate category was created for factors specific to delivering LIs via telehealth. Attention for this modality of healthcare provision has been growing for some time[62]. In addition, during the course of the current review the COVID-19 pandemic emerged, which meant that many HCPs actually had to use telehealth in their daily practice[63]. Although telehealth was not a specific focus of this review, it could be interesting to further investigate the experiences with telehealth and its value for long-term counseling of patients with hip and/or knee OA on behavioral change[64].

To the best of our knowledge, this is the first review to focus specifically on the implementation of LIs as conservative treatment for hip and/or knee OA while taking into account the perceptions of both primary and secondary HCPs. Both qualitative and quantitative data were included, providing broad insight into the topic. All included studies were conducted in North America, Europe and Oceania. Given that the majority of these studies were conducted quite recently, our results are expected to be representative of the current situation in these continents.

For peer review only - http://bmjopen.bmj.com/site/about/guidelines.xhtml

There are also a few limitations to acknowledge. First, "implementing LIs" was defined very broadly and can be seen as an umbrella term, ranging from mentioning a healthy lifestyle to running specific lifestyle programs. Consequently, the identified barriers and facilitators may not fit with every single way of implementing LIs, but may rather provide insight into the full spectrum of influencing factors. Although data synthesis was not performed separately for physical activity and weight management, the created overview gives us the overall impression that barriers and facilitators related to these two lifestyle components are quite similar. One barrier that seems to be unique to weight management is the perception of it being a difficult or sensitive subject to discuss. Regarding physical activity, the perception that it is unsafe or has negative effects seems to be a unique barrier. Second, although data extraction and quality assessment were performed by two researchers independently, data analysis was performed primarily by one researcher. By discussing the resulting classification of factors and any doubts during the process with members of the research team, we aimed to increase the reliability of our findings. Lastly, as we did not search grey literature there is a slight chance that relevant studies may have been missed.

The comprehensive overview of barriers and facilitators for implementing LIs in patients with hip and/or knee OA by HCPs resulting from this review can serve as a basis for further research and the development of implementation strategies that focus on both the individual and the environmental context of HCPs. However, what the relative importance of the identified factors is and whether differences exist between the various types of primary and secondary HCPs with respect to these factors are not known yet. Further research is required to provide more insight into this relative importance and therewith the most relevant targets for change in daily practice.

For peer review only - http://bmjopen.bmj.com/site/about/guidelines.xhtml

Conclusion

This review has shown that multiple factors influence whether or not HCPs implement LIs when treating patients with hip and/or knee OA. Data analysis has resulted in a comprehensive overview of influencing factors, where barriers and facilitators have been subdivided into nine domains, both at an individual and at several environmental levels. The review contributes to existing knowledge about the implementation of LIs by identifying multiple factors related to the intervention, interdisciplinary collaboration, and the organizational and societal context. The broad inventory created in this review can be a first step toward an improved implementation of LIs by HCPs in OA care. Future research in this area should focus on determining the relative importance of the identified factors involving all relevant disciplines of primary and secondary HCPs.

Acknowledgements

The authors would like to thank Truus van Ittersum for her advice on search strategies and Annemarie Jenks for her contribution to the study selection process.

Author contributions

Conception and design: SB, RD, LvdW, MS, IvdAS. Collection and assembly of data: SB, JvB. Analysis and interpretation of the data: SB, MS, IvdAS. Drafting of the article: SB. Critical revision of the article for important intellectual content: SB, JvB, RD, LvdW, MS, IvdAS. Final approval of the article: SB, JvB, RD, LvdW, MS, IvdAS. Obtaining of funding: SB, RD, LvdW, MS, IvdAS.

Role of the funding source

For peer review only - http://bmjopen.bmj.com/site/about/guidelines.xhtml

This study is supported by a scholarship from the University of Groningen/University Medical Center Groningen (grant/award number: not applicable). The funders played no role in the study design, collection, analysis and interpretation of data, writing of the manuscript, or the decision to submit the manuscript for publication.

Conflict of interest

The authors report no potential conflicts of interest.

Data sharing statement

Data sharing is not applicable to this article as no new data were created or analyzed in this study.

References

1. Koninklijk Nederlands Genootschap voor Fysiotherapie (KNGF). KNGF-richtlijn Artrose heup-knie. Conservatieve, pre- en postoperatieve behandeling [Internet]. 2018 [cited 2020 Aug 25]. Available from: https://www.kngf.nl/kennisplatform/richtlijnen/artrose-heup-knie.

2. Nederlands Huisartsen Genootschap (NHG). NHG-Standaard Niet-traumatische knieklachten [Internet]. 2016 [cited 2020 Aug 25]. Available from: https://www.nhg.org/standaarden/samenvatting/niet-traumatische-knieklachten.

3. Nederlandse Orthopaedische Vereniging (NOV). Conservatieve behandeling van artrose in heup of knie [Internet]. 2018 [cited 2020 Aug 25]. Available from: https://richtlijnendatabase.nl/richtlijn/artrose_in_heup_of_knie/startpagina_-_heup-_of_knieartrose.html.

For peer review only - http://bmjopen.bmj.com/site/about/guidelines.xhtml

4. Bannuru RR, Osani MC, Vaysbrot EE, Arden NK, Bennell K, Bierma-Zeinstra SMA, et al. OARSI guidelines for the non-surgical management of knee, hip, and polyarticular osteoarthritis. Osteoarthr Cartilage 2019; 27: 1578-89.

5. Fernandes L, Hagen KB, Bijlsma JWJ, Andreassen O, Christensen P, Conaghan PG, et al. EULAR recommendations for the non-pharmacological core management of hip and knee osteoarthritis. Ann Rheum Dis 2013; 72: 1125-35.

6. Fransen M, McConnell S, Hernandez-Molina G, Reichenbach S. Exercise for osteoarthritis of the hip. Cochrane Database Syst Rev. 2014(4):CD007912. DOI:10.1002/14651858.CD007912.pub2.

7. Fransen M, McConnell S, Harmer AR, Van der Esch M, Simic M, Bennell KL. Exercise for osteoarthritis of the knee: a Cochrane systematic review. Br J Sports Med 2015; 49: 1554-7.

8. Messier SP, Loeser RF, Miller GD, Morgan TM, Rejeski WJ, Sevick MA, et al. Exercise and dietary weight loss in overweight and obese older adults with knee osteoarthritis: the Arthritis, Diet, and Activity Promotion Trial. Arthritis Rheum 2004; 50: 1501-10.

9. Paans N, van den Akker-Scheek I, Dilling RG, Bos M, van der Meer K, Bulstra SK, et al. Effect of exercise and weight loss in people who have hip osteoarthritis and are overweight or obese: a prospective cohort study. Phys Ther 2013; 93: 137-46.

10. Svege I, Nordsletten L, Fernandes L, Risberg MA. Exercise therapy may postpone total hip replacement surgery in patients with hip osteoarthritis: a long-term follow-up of a randomised trial. Ann Rheum Dis 2015; 74: 164-9.

11. Snijders GF, den Broeder AA, van Riel PL, Straten VH, de Man FH, van den Hoogen FH, et al. Evidence-based tailored conservative treatment of knee and hip osteoarthritis: between knowing and doing. Scand J Rheumatol 2011; 40: 225-31.

For peer review only - http://bmjopen.bmj.com/site/about/guidelines.xhtml

12. Bennell KL, Dobson F, Hinman RS. Exercise in osteoarthritis: moving from prescription to adherence. Best Pract Res Clin Rheumatol 2014; 28: 93-117.

13. Dobson F, Bennell KL, French SD, Nicolson PJ, Klaasman RN, Holden MA, et al. Barriers and facilitators to exercise participation in people with hip and/or knee osteoarthritis: synthesis of the literature using behavior change theory. Am J Phys Med Rehabil 2016; 95: 372-89.

14. Kanavaki AM, Rushton A, Efstathiou N, Alrushud A, Klocke R, Abhishek A, et al. Barriers and facilitators of physical activity in knee and hip osteoarthritis: a systematic review of qualitative evidence. BMJ Open. 2017;7(12):e017042. DOI:10.1136/bmjopen-2017-017042

15. Egerton T, Diamond LE, Buchbinder R, Bennell KL, Slade SC. A systematic review and evidence synthesis of qualitative studies to identify primary care clinicians' barriers and enablers to the management of osteoarthritis. Osteoarthr Cartilage 2017; 25: 625-38.

16. Landelijke Huisartsen Vereniging (LHV)/Nederlands Huisartsen Genootschap (NHG). Toekomstvisie Huisartsenzorg 2022 [Internet]. 2012 [cited 2020 Dec 21]. Available from: https://www.nhg.org/toekomstvisie.

17. Colquhoun HL, Levac D, O'Brien KK, Straus S, Tricco AC, Perrier L, et al. Scoping reviews: time for clarity in definition, methods, and reporting. J Clin Epidemiol 2014; 67: 1291-4.

18. Flottorp SA, Oxman AD, Krause J, Musila NR, Wensing M, Godycki-Cwirko M, et al. A checklist for identifying determinants of practice: a systematic review and synthesis of frameworks and taxonomies of factors that prevent or enable improvements in healthcare professional practice. Implement Sci. 2013;8:35. DOI:10.1186/1748-5908-8-35

For peer review only

For peer review only - http://bmjopen.bmj.com/site/about/guidelines.xhtml

19. Arksey H, O'Malley L. Scoping studies: towards a methodological framework. Int J Soc Res Method 2005; 8: 19-32.

20. Tricco AC, Lillie E, Zarin W, O'Brien KK, Colquhoun H, Levac D, et al. PRISMA Extension for Scoping Reviews (PRISMA-ScR): Checklist and Explanation. Ann Intern Med 2018; 169: 467-73.

21. Peters MDJ, Godfrey CM, McInerney P, Baldini Soares C, Khalil H, Parker D. The Joanna Briggs Institute Reviewers' Manual 2015: Methodology for JBI scoping reviews. Adelaide: The Joanna Briggs Institute 2015.

22. Pluye P, Gagnon M-P, Griffiths F, Johnson-Lafleur J. A scoring system for appraising mixed methods research, and concomitantly appraising qualitative, quantitative and mixed methods primary studies in Mixed Studies Reviews. Int J Nurs Stud 2009; 46: 529-46.

23. Hong QN, Pluye P, Fàbregues S, Bartlett G, Boardman F, Cargo M, et al. Mixed Methods Appraisal Tool (MMAT), version 2018. Registration of Copyright (#1148552), Canadian Intellectual Property Office, Industry Canada 2018.

24. Garne-Dalgaard A, Mann S, Bredahl TVG, Stochkendahl MJ. Implementation strategies, and barriers and facilitators for implementation of physical activity at work: a scoping review. Chiropr Man Therap. 2019;27. DOI:10.1186/s12998-019-0268-5

25. Weatherson KA, Gainforth HL, Jung ME. A theoretical analysis of the barriers and facilitators to the implementation of school-based physical activity policies in Canada: a mixed methods scoping review. Implementation Sci. 2017;12. DOI:10.1186/s13012-017-0570-3

26. Allison K, Setchell J, Egerton T, Delany C, Bennell KL. In Theory, Yes; in Practice, Uncertain: A Qualitative Study Exploring Physical Therapists' Attitudes Toward Their

For peer review only

For peer review only - http://bmjopen.bmj.com/site/about/guidelines.xhtml

Roles in Weight Management for People With Knee Osteoarthritis. Phys Ther 2019; 99: 601-11.

27. Bossen D, Kloek C, Snippe HW, Dekker J, de Bakker D, Veenhof C. A Blended Intervention for Patients With Knee and Hip Osteoarthritis in the Physical Therapy Practice: Development and a Pilot Study. JMIR Res Protoc 2016; 5: e32.

28. Christiansen MB, White DK, Christian J, Waugh E, Gakhal N, King L, et al. "It ... doesn't always make it [to] the top of the list" Primary care physicians' experiences with prescribing exercise for knee osteoarthritis. Canadian Family Physician 2020; 66: e14-20.

29. Davis AM, Kennedy D, Wong R, Robarts S, Skou ST, McGlasson R, et al. Cross-cultural adaptation and implementation of Good Life with osteoarthritis in Denmark (GLA:D™): group education and exercise for hip and knee osteoarthritis is feasible in Canada. Osteoarthritis Cartilage 2018; 26: 211-9.

30. de Rooij M, van der Leeden M, Avezaat E, Häkkinen A, Klaver R, Maas T, et al. Development of comorbidity-adapted exercise protocols for patients with knee osteoarthritis. Clin Interv Aging 2014; 9: 829-42.

31. Egerton T, Nelligan R, Setchell J, Atkins L, Bennell KL. General practitioners' perspectives on a proposed new model of service delivery for primary care management of knee osteoarthritis: a qualitative study. BMC Fam Pract 2017; 18: 85.

32. Egerton T, Nelligan RK, Setchell J, Atkins L, Bennell KL. General practitioners' views on managing knee osteoarthritis: a thematic analysis of factors influencing clinical practice guideline implementation in primary care. BMC Rheumatol 2018; 2: 30.

For peer review only

33. Hinman RS, Delany CM, Campbell PK, Gale J, Bennell KL. Physical Therapists, Telephone Coaches, and Patients With Knee Osteoarthritis: Qualitative Study About Working Together to Promote Exercise Adherence. Phys Ther 2016; 96: 479-93.

34. Hinman RS, Nelligan RK, Bennell KL, Delany C. "Sounds a Bit Crazy, But It Was Almost More Personal:" A Qualitative Study of Patient and Clinician Experiences of Physical Therapist-Prescribed Exercise For Knee Osteoarthritis Via Skype. Arthritis Care Res (Hoboken) 2017; 69: 1834-44.

35. Knoop J, van der Leeden M, van der Esch M, de Rooij M, Peter WF, Bennell KL, et al. Is a model of stratified exercise therapy by physical therapists in primary care feasible in patients with knee osteoarthritis? : a mixed methods study. Physiotherapy 2020; 106: 101-10.

36. Law RJ, Nafees S, Hiscock J, Wynne C, Williams NH. A lifestyle management programme focused on exercise, diet and physiotherapy support for patients with hip or knee osteoarthritis and a body mass index over 35: A qualitative study. Musculoskeletal Care 2019; 17: 145-51.

37. Lawford BJ, Delany C, Bennell KL, Hinman RS. "I Was Really Pleasantly Surprised": Firsthand Experience and Shifts in Physical Therapist Perceptions of Telephone-Delivered Exercise Therapy for Knee Osteoarthritis-A Qualitative Study. Arthritis Care Res (Hoboken) 2019; 71: 545-57.

38. Lawford BJ, Bennell KL, Allison K, Schwartz S, Hinman RS. Challenges with strengthening exercises for people with knee osteoarthritis and comorbid obesity: a qualitative study with patients and physiotherapists. Arthritis Care Res (Hoboken). 2020. DOI:10.1002/acr.24439

39. Lawford BJ, Bennell KL, Jones SE, Keating C, Brown C, Hinman RS. "It's the single best thing I've done in the last 10 years": a qualitative study exploring patient and

For peer review only

dietitian experiences with, and perceptions of, a multi-component dietary weight loss program for knee osteoarthritis. Osteoarthritis Cartilage 2021; 29: 507-17.

40. MacKay C, Hawker GA, Jaglal SB. Qualitative study exploring the factors influencing physical therapy management of early knee osteoarthritis in Canada. BMJ Open 2018; 8: e023457.

41. MacKay C, Hawker GA, Jaglal SB. How Do Physical Therapists Approach Management of People With Early Knee Osteoarthritis? A Qualitative Study. Phys Ther 2020; 100: 295-306.

42. Mann C, Gooberman-Hill R. Health care provision for osteoarthritis: concordance between what patients would like and what health professionals think they should have. Arthritis Care Res (Hoboken) 2011; 63: 963-72.

43. Miller KA, Osman F, Baier Manwell L. Patient and physician perceptions of knee and hip osteoarthritis care: A qualitative study. Int J Clin Pract 2020; 74: e13627.

44. Nielsen M, Keefe FJ, Bennell K, Jull GA. Physical therapist-delivered cognitive-behavioral therapy: a qualitative study of physical therapists' perceptions and experiences. Phys Ther 2014; 94: 197-209.

45. Okwera A, May S. Views of general practitioners toward physiotherapy management of osteoarthritis-a qualitative study. Physiother Theory Pract 2019; 35: 940-6.

46. Poitras S, Rossignol M, Avouac J, Avouac B, Cedraschi C, Nordin M, et al. Management recommendations for knee osteoarthritis: how usable are they? Joint Bone Spine 2010; 77: 458-65.

47. Rosemann T, Wensing M, Joest K, Backenstrass M, Mahler C, Szecsenyi J. Problems and needs for improving primary care of osteoarthritis patients: the views of patients, general practitioners and practice nurses. BMC Musculoskel Dis 2006; 7: 48.

For peer review only - http://bmjopen.bmj.com/site/about/guidelines.xhtml

48. Selten EMH, Vriezekolk JE, Nijhof MW, Schers HJ, van der Meulen-Dilling RG, van der Laan WH, et al. Barriers Impeding the Use of Non-pharmacological, Non-surgical Care in Hip and Knee Osteoarthritis: The Views of General Practitioners, Physical Therapists, and Medical Specialists. J Clin Rheumatol 2017; 23: 405-10.

49. Tang CY, Pile R, Croft A, Watson NJ. Exploring Physical Therapist Adherence to Clinical Guidelines When Treating Patients With Knee Osteoarthritis in Australia: A Mixed Methods Study. Phys Ther 2020; 100: 1084-93.

50. Teo PL, Bennell KL, Lawford BJ, Egerton T, Dziedzic KS, Hinman RS. Physiotherapists may improve management of knee osteoarthritis through greater psychosocial focus, being proactive with advice, and offering longer-term reviews: a qualitative study. J Physiother 2020; 66: 256-65.

51. Wallis JA, Ackerman IN, Brusco NK, Kemp JL, Sherwood J, Young K, et al. Barriers and enablers to uptake of a contemporary guideline-based management program for hip and knee osteoarthritis: A qualitative study. Osteoarthritis and Cartilage Open 2020; 2.

52. Cottrell E, Roddy E, Rathod T, Porcheret M, Foster NE. What influences general practitioners' use of exercise for patients with chronic knee pain? Results from a national survey. BMC Fam Pract 2016; 17: 172.

53. Duarte N, Hughes SL, Paúl C. Cultural adaptation and specifics of the Fit & Strong! program in Portugal. Transl Behav Med 2019; 9: 67-75.

54. Hill DS, Freudmann M, Sergeant JC, Board T. Management of symptomatic knee osteoarthritis in obesity: a survey of orthopaedic surgeons' opinions and practice. Eur J Orthop Surg Traumatol 2018; 28: 967-74.

For peer review only - http://bmjopen.bmj.com/site/about/guidelines.xhtml

55. Hill D, Boyd A, Board T. Management of symptomatic knee osteoarthritis in obesity: a survey of general practitioners' opinions and practice. Eur J Orthop Surg Traumatol 2018; 28: 1183-9.

56. Hofstede SN, Marang-van de Mheen PJ, Vliet Vlieland TP, van den Ende CH, Nelissen RG, van Bodegom-Vos L. Barriers and Facilitators Associated with Non-Surgical Treatment Use for Osteoarthritis Patients in Orthopaedic Practice. PLoS One 2016; 11: e0147406.

57. Lawford BJ, Bennell KL, Kasza J, Hinman RS. Physical Therapists' Perceptions of Telephone- and Internet Video-Mediated Service Models for Exercise Management of People With Osteoarthritis. Arthritis Care Res (Hoboken) 2018; 70: 398-408.

58. Reid DA, Potts G, Burnett M, Konings B. Physiotherapy management of knee and hip osteoarthritis: a survey of patient and medicai practitioners' expectations, experiences and perceptions of effectiveness of treatment. New Zeal J Physiother 2014; 42: 118-25.

59. de Rooij M, van der Leeden M, van der Esch M, Lems WF, Meesters JJL, Peter WF, et al. Evaluation of an educational course for primary care physiotherapists on comorbidity-adapted exercise therapy in knee osteoarthritis: an observational study. Musculoskeletal Care 2020; 18: 122-33.

60. Holden MA, Nicholls EE, Young J, Hay EM, Foster NE. UK-based physical therapists' attitudes and beliefs regarding exercise and knee osteoarthritis: findings from a mixed-methods study. Arthritis Rheum 2009; 61: 1511-21.

61. Kloek CJJ, Bossen D, de Vries HJ, de Bakker DH, Veenhof C, Dekker J. Physiotherapists' experiences with a blended osteoarthritis intervention: a mixed methods study. Physiother Theory Pract 2020; 36: 572-9.

For peer review only - http://bmjopen.bmj.com/site/about/guidelines.xhtml

62. Slater H, Dear BF, Merolli MA, Li LC, Briggs AM. Use of eHealth technologies to enable the implementation of musculoskeletal Models of Care: Evidence and practice. Best Pract Res Clin Rheumatol 2016; 30: 483-502.

63. Loeb AE, Rao SS, Ficke JR, Morris CD, Riley LH, Levin AS. Departmental Experience and Lessons Learned With Accelerated Introduction of Telemedicine During the COVID-19 Crisis. J Am Acad Orthop Surg 2020; 28: e469-76.

64. Piga M, Cangemi I, Mathieu A, Cauli A. Telemedicine for patients with rheumatic diseases: Systematic review and proposal for research agenda. Semin Arthritis Rheum 2017; 47: 121-8.

For peer review only

Table 1. Overview of included studies[a]

Reference	Country and health setting	Study focus	Type of data extracted	Data collection method	Data analysis method	Participants
Allison[26] (2019)	Australia (private primary care and public hospital care or community health)	Attitudes and perceptions toward role in weight management (knee OA)	Qualitative	Individual interviews	Inductive thematic analysis	PT (n=13, 61% female, age range 27-61 years)
Bossen[27] (2016)	The Netherlands (private practice)	Development and feasibility of the blended exercise therapy intervention "e-Exercise" (hip and/or knee OA)	Qualitative	1) Focus group<br>2) Individual interviews	1) Summarizing<br>2) Thematic trend analysis	1) PT (n=7)<br>2) PT (n=5)
Christiansen[28] (2020)	Canada (academic and community family health practice)	Experiences with and barriers to prescribing exercise (knee OA)	Qualitative	Individual interviews	Constant comparison approach	Physician (n=11)
Davis[29] (2018)	Canada (single assessment center)	Implementation of the "Good Life with osteoArthritis in Denmark (GLA:D^{TM}) Canada" program (hip and/or knee OA)	Qualitative	Individual interviews	Thematic content analysis	PT (n=3)
De Rooij[30] (2014)	The Netherlands (rehabilitation center)	Development of comorbidity-adapted exercise protocols (knee OA)	Qualitative	Individual interviews	Analyzing notes	PT (n=3)
Egerton[31] (2017)[b]	Australia (primary care)	Perspectives on potential barriers and facilitators to engagement with a proposed model of service delivery for primary care management (knee OA)	Qualitative	Individual interviews	Interpretive thematic analysis	GP (n=11, 64% female, mean age 50.8 years (range: 34-67))
Egerton[32] (2018)[b]	Australia (primary care)	Barriers and facilitators influencing clinical practice guideline implementation in	Qualitative	Individual interviews	Interpretive thematic analysis	GP (n=11, 64% female)

For peer review only - http://bmjopen.bmj.com/site/about/guidelines.xhtml

		primary care (knee OA)				
Hinman[33] (2016)	Australia (private practice)	Experiences of being involved in delivering an integrated program of PT-supervised exercise and telephone coaching (knee OA)	Qualitative	Individual interviews	Thematic analysis informed by grounded theory	PT (n=10, 50% female, mean age 43 years (SD: 13))<br><br>Telephone coach (n=4; 100% female, mean age 42 years (SD: 11))
Hinman[34] (2017)	Australia (not specified)	Experiences using Skype as a service delivery model for PT-prescribed exercise management (knee OA)	Qualitative	Individual interviews	Thematic and constant comparative analytical approach	PT (n=8, 50% female, mean age 39 years (SD: 9))
Knoop[35] (2020)	The Netherlands (primary care)	Feasibility of a newly developed model of stratified exercise therapy in primary care (knee OA)	Qualitative	1) Individual interviews<br><br>2) Focus group	Analyzed descriptively	1) PT (n=9)<br><br>2) PT (n=14)
Law[36] (2019)	United Kingdom (leisure center)	Experiences and views of referring and delivering professionals regarding the "Lifestyle Management Program" (hip and/or knee OA)	Qualitative	1) Focus groups<br><br>2) Individual interviews	Framework analysis method	1) Dietician (n=2)<br><br>Exercise professional (n=3)<br><br>PT (n=4)<br><br>Triaging clinician (n=1)<br><br>2) GP (n=3)<br><br>Total group: 46% female

For peer review only - http://bmjopen.bmj.com/site/about/guidelines.xhtml

Lawford[37] (2019)	Australia (private and public practice)	Pre- and post-intervention perceptions of telephone-delivered exercise therapy (knee OA)	Qualitative	Individual interviews	Thematic analysis approach	PT (n=8, 50% female)
Lawford[38] (2020)	Australia (private and public practice)	Experiences and perceptions with prescribing a strengthening exercise program for people with comorbid obesity (knee OA)	Qualitative	Individual interviews	Inductive thematic approach	PT (n=7, 14% female)
Lawford[39] (2021)	Australia (private and public practice)	Experiences with a multi-component dietary weight loss program (knee OA)	Qualitative	Individual interviews	Thematic approach informed by grounded theory	Dietician (n=5, 100% female)
MacKay[40] (2018)[c]	Canada (community-based and outpatient setting)	Factors influencing physical therapy management (knee OA)	Qualitative	Individual interviews	Inductive thematic analysis	PT (n=33, 76% female)
MacKay[41] (2020)[c]	Canada (community-based and outpatient setting)	Perceptions related to physical therapy management (knee OA)	Qualitative	Individual interviews	Inductive thematic analysis	PT (n=33, 76% female)
Mann[42] (2011)	United Kingdom (primary and secondary care)	Perceptions of current service provision and possible service improvements (hip and/or knee OA)	Qualitative	Individual interviews	Framework method	GP (n=2)<br>Nurse practitioner (n=1)<br>Occupational therapist (n=1)<br>OS (n=2)<br>Practice nurse (n=3)<br>PT (n=2)

For peer review only - http://bmjopen.bmj.com/site/about/guidelines.xhtml

						RH (n=1)
Miller[43] (2020)	United States of America (large academic medical center)	Barriers and facilitators to guideline-based treatment (hip and/or knee OA)	Qualitative	Individual interviews	Conventional content analysis	Physician (n=6, 50% female)
Nielsen[44] (2014)	Australia (not specified)	Perspectives on and experiences with an intervention of exercise combined with cognitive behavioral therapy (Pain Coping Skills Training) and the implementation process (knee OA)	Qualitative	Individual interviews	Framework analysis	PT (n=8, 88% female, age range 35-58 years)
Okwera[45] (2019)	United Kingdom (general practice within NHS)	Beliefs on physiotherapy management in primary care (hip and/or knee OA)	Qualitative	Individual interviews	Framework analysis	GP (n=8, 50% female, age range 31-60 years)
Poitras[46] (2010)	France (general practice; work setting PTs not specified)	Barriers to use of conservative management recommendations (knee OA)	Qualitative	Focus groups	Thematic content analysis	GP (n=7, 29% female, median age 53 years (range: 48-77))<br>PT (n=10, 40% female, median age 46.5 years (range: 24-69))
Rosemann[47] (2006)	Germany (general practice)	Problems and needs for improving primary care (hip and/or knee OA)	Qualitative	Individual interviews	Description of coding process, but no specific method reported	GP (n=20, 20% female, mean age 43.5 years (range: 33-57))<br>Practice nurse (n=20, 100% female, mean age 41.3 years (range: 29-56))
Selten[48] (2017)	The Netherlands	Views on non-pharmacological, non-	Qualitative	Individual	Thematic analysis	GP (n=5)

For peer review only - http://bmjopen.bmj.com/site/about/guidelines.xhtml

	(general practice; work setting PTs, OSs and RHs not specified)	surgical management (hip and/or knee OA)		interviews		OS (n=7)<br>PT (n=7)<br>RH (n=5)<br>Total group: 50% female, age range 24-64 years
Tang[49] (2020)	Australia (large metropolitan public health service)	Application of clinical practice guidelines (knee OA)	Qualitative	Individual interviews	Thematic analysis	PT (n=18)
Teo[50] (2020)	Australia (private practice and tertiary or non-tertiary hospitals)	Experiences with delivering care (knee OA)	Qualitative	Individual interviews	Inductive thematic approach	PT (n=22, 50% female, mean age 34 years (SD: 8, range: 24-54))
Wallis[51] (2020)	Australia (general practice; OSs and RHs working in private and public hospitals)	Perceptions about management including barriers and enablers for referral to the "Good Life with osteoArthritis in Denmark (GLA:D®) Australia" program (hip and/or knee OA)	Qualitative	Individual interviews	Inductive thematic analysis	GP (n=5)<br>OS (n=6)<br>RH (n=4)<br>Total group: mean age 52 years (SD: 12)
Cottrell[52] (2016)	United Kingdom (general practice)	Attitudes and beliefs regarding exercise (knee OA)	Quantitative	Survey (RR: 17%)	Descriptive statistics (frequency)	GP (n=835, 51% female)
Duarte[53] (2019)	Portugal (not specified)	Development and acceptability of the Portuguese version of the "Fit & Strong!" program (hip and/or knee OA)	Quantitative	Survey (RR: 100%)	Not reported	Program instructor (n=2)
Hill[54] (2018)	United Kingdom	Opinions and practices regarding the	Quantitative	Survey (RR:	Descriptive statistics	OS (n=205)

For peer review only - http://bmjopen.bmj.com/site/about/guidelines.xhtml

	(specialist practice in knee surgery)	management of symptomatic OA in obesity (knee OA)		52%)	(frequency)	
Hill[55] (2018)	United Kingdom (general practice)	Opinions and practices regarding the management of symptomatic OA in obesity (knee OA)	Quantitative	Survey (RR: 75%)	Descriptive statistics (frequency)	GP (n=130)
Hofstede[56] (2016)	The Netherlands (52% of OSs worked at a general hospital)	Barriers and facilitators associated with prescription of different non-surgical treatments (hip and/or knee OA)	Quantitative	Survey (RR: 36%)	Descriptive statistics (frequency)	OS (n=172, 9% female, mean age 48.4 years (SD: 8.6))
Lawford[57] (2018)	Australia (private and public practice)	Perceptions of remotely-delivered service models for exercise management (hip and/or knee OA)	Quantitative	Survey (RR: unknown)	Descriptive statistics (frequency and level of agreement)	PT (n=217, 72% female)
Reid[58] (2014)	New Zealand (general practice; work setting OSs not specified)	Self-reported behavior, experiences, expectations and perceptions regarding physiotherapy referral and management (hip and/or knee OA)	Quantitative	Survey (RR: 46% (GP) and 26% (OS))	Descriptive statistics (frequency)	GP (n=24)<br><br>OS (n=20)<br><br>Total group: 34% female, mean age 52.2 years (SD: 8.5)
De Rooij[59] (2020)	The Netherlands (primary care)	Facilitators and barriers for usage of a strategy for exercise prescription in patients with comorbidity (knee OA)	Mixed-methods	1) Survey (RR: 100%)<br><br>2) Individual interviews	1) Descriptive statistics (frequency)<br><br>2) Summarizing notes	1) PT (n=34, 68% female, mean age 43.7 years (SD: 11.1))<br><br>2) PT (n=10)
Holden[60] (2009)	United Kingdom (NHS and non-NHS)	Attitudes and beliefs regarding exercise (knee OA)	Mixed-methods	1) Survey (RR: 58%)<br><br>2) Individual interviews	1) Descriptive statistics (level of agreement)<br><br>2) Thematic analysis	1) PT (n=538, 87% female)<br><br>2) PT (n=24, 67% female)

For peer review only - http://bmjopen.bmj.com/site/about/guidelines.xhtml

Kloek[61] (2020)	The Netherlands (primary care practice)	Experiences with and determinants related to the usage of the blended physiotherapy intervention "e-Exercise" (hip and/or knee OA)	Mixed-methods	1) Survey (RR: 40%)<br><br>2) Individual interviews	1) Descriptive statistics (frequency)<br>2) Grounded theory methodology	1) PT (n=49)<br><br>2) PT (n=9, 33% female, median age 52 years (range: 24-59))

694 [a] GP: general practitioner; NHS: National Health Service; OA: osteoarthritis; OS: orthopedic surgeon; PT: physiotherapist; RH: rheumatologist; RR: response rate; SD:
695 standard deviation.
696 [b,c] Data for both studies were collected during the same interview.

For peer review only

Table 2. Distribution of the extracted factors per included article across the domains, which were largely based on the Tailored Implementation of Chronic Diseases checklist[a]

Reference	Domain 1: Intervention factors	Domain 2: Individual HCP factors	Domain 3: Patient factors	Domain 4: Professional interactions	Domain 5: Incentives and resources	Domain 6: Capacity for organizational change	Domain 7: Social, political, and legal factors	Domain 8: Patient and HCP interactions	Domain 9: Disease factors	Total number of factors in article
Allison[26] (2019)		3		2	2	1		4		12
Bossen[27] (2016)	8									8
Christiansen[28] (2020)	1	5	2						1	9
Davis[29] (2018)	6		1							7
De Rooij[30] (2014)	3	2								5
Egerton[31] (2017)	20	3	1	9	3				1	37
Egerton[32] (2018)	5	9	5		6		1	1	5	32
Hinman[33] (2016)	7	1	2	10						20
Hinman[34]	18									18

For peer review only

(2017)										
Knoop[35] (2020)	4		1	1						6
Law[36] (2019)	8	1	5	1	2			1		18
Lawford[37] (2019)	26									26
Lawford[38] (2020)	11		7					1		19
Lawford[39] (2021)	12		3							15
MacKay[40] (2018)	6	5	14	7	6	2	1			41
MacKay[41] (2020)	4	12	5	1	1			4		27
Mann[42] (2011)	2	1	4	10	1				1	19
Miller[43] (2020)	4	4	7	3	8		1	1	1	29
Nielsen[44] (2014)	13	8	1		3	2				27
Okwera[45] (2019)	4	6	6	12			2		2	32
Poitras[46]	11	13	19	3				1	5	52

For peer review only

(2010)										
Rosemann[47] (2006)	1	4	5	4	6		1	1	1	23
Selten[48] (2017)	7	3	3	14	2			4		33
Tang[49] (2020)		12	4					1		17
Teo[50] (2020)	3	11	8						1	23
Wallis[51] (2020)	17		7	3	2				1	30
Cottrell[52] (2016)	12	10	4	2	3					31
Duarte[53] (2019)	1		2							3
Hill[54] (2018)		5		2						7
Hill[55] (2018)	2	4		2						8
Hofstede[56] (2016)	5	3		4	1	1				14
Lawford[57] (2018)	33									33
Reid[58] (2014)	4	1	3	1						9
De Rooij[59] (2020)	18	8	4	9	2	1	3			45

For peer review only

Holden[60] (2009)	13	10	14		3				2	42
Kloek[61] (2020)	26			1	5					32
**Total number of factors in domain**	**315**	**144**	**137**	**101**	**56**	**7**	**9**	**19**	**21**	**809**

[a] HCP: healthcare professional.

For peer review only

For peer review only - http://bmjopen.bmj.com/site/about/guidelines.xhtml

Table 3. Overview of barriers, facilitators, and unclear factors that influence the implementation of LIs as perceived by HCPs for all domains, which were largely based on the Tailored Implementation of Chronic Diseases checklist[a]

Category	Barriers	Facilitators	Unclear factors
Domain 1: Intervention factors			
Effectiveness	- LIs have little or no effect on OA[28,31,32,43,45,46,48,52,58,60]<br>- Potential effects of LIs are difficult to accomplish[46,47,52,60]	- LIs have positive effects on affected joint(s)[34,37,39-41,46,48,51,52,57,60]<br>- LIs have positive effects on general health[32,39,46,48,55,56]<br>- LIs have positive mental effects[29,34,36,37,39,48,51,56]<br>- LIs have positive effects (not further specified)[33,36,43,48,51,53,56]	
Safety	- LIs are unsafe or have negative effects[38,46,51,60]	- LIs are safe[52,56]<br>- Research environment or protocols provide a safety net[30,34,37,38]	
Design	- Non-optimal content or structure of LIs[33,35,51,52,61]<br>- Challenges for patients during participation in LIs[38,39,44]<br>- Challenges for HCPs during delivery of LIs[27,29,38,59,61]	- Positive experiences with or suggestions to improve the content or structure of LIs[27,29,33,36,39,44,51,59,61]<br>- Ease for patients during participation in LIs[38,39,51]<br>- Ease for HCPs during delivery of LIs[29,30,33,38,44,59,61]	
Personalized treatment	- Insufficient ability to provide personalized treatment within LIs[31,44,61]	- Ability and importance of providing personalized treatment within LIs[36,38,41,44,46,50,52,59-61]	
Accessibility	- LIs are unavailable or inaccessible[27,32,40,42,43,52,55,58,60]	- LIs are available or accessible, or suggestions for improvement[31,36,40,45,56,58]	

For peer review only - http://bmjopen.bmj.com/site/about/guidelines.xhtml

	- Costs of LIs to patients[31,32,40,43,50,51]<br><br>- LIs are not feasible or sustainable[31,59]<br><br>- Inconvenience to patients when accessing LIs[50-52]	- LIs are feasible or sustainable[31,35,36,41,59]<br><br>- Convenience for patients when accessing LIs[51]	
Telehealth	- Disadvantages of telehealth in terms of effectiveness[31,57,61]<br><br>- Telehealth is not safe for patients or patient/data privacy[31,57]<br><br>- Challenges for HCPs regarding lack of physical/visual contact[34,37,57,61]<br><br>- Other challenges for HCPs regarding feasibility of telehealth[27,31,34,37,57,61]<br><br>- Patient-related challenges regarding feasibility of telehealth[27,31,61]<br><br>- Negative aspects regarding communication and relationship using telehealth[33,34,37,39]	- Benefits of telehealth in terms of effectiveness[27,34,37,57,61]<br><br>- Telehealth is safe for patients or patient/data privacy[34,57,61]<br><br>- Lack of physical/visual contact not a major issue for HCPs[34,37,57]<br><br>- Positive attitude or needs of HCPs regarding feasibility of telehealth[34,37,39,57,61]<br><br>- Patient-related benefits regarding feasibility of telehealth[27,31,34,37,57]<br><br>- Positive aspects regarding communication and relationship using telehealth[37,39]	
Domain 2: Individual HCP factors			
Expertise	- Lack of knowledge or skills around LIs or promoting behavioral change[26,28,32,40,41,44,46,48-50,55,59,60]<br><br>- Lack of knowledge or skills around OA care in general[42,43,45,47]<br><br>- Lack of knowledge or skills around specific resources[32,49,59]	- Having or improving knowledge or skills around LIs or promoting behavioral change[32,33,40,41,44,45,49]<br><br>- Having or improving knowledge or skills around OA care in general[32,43,45,47]<br><br>- Available resources might improve knowledge and decision-making[30,49,59]	- Clinical experience[41]

For peer review only - http://bmjopen.bmj.com/site/about/guidelines.xhtml

Attitude	- Negative attitude toward LIs[28,52,60]<br><br>- Negative attitude toward guidelines or protocols[45]	- Positive attitude toward LIs[32,40,41,44-46,49,50,52,54-56,58]<br><br>- Positive attitude toward guidelines or protocols[26,56,59]	- Autonomy[36]
Role	- Perception of own role potentially impeding prescription or follow-up of LIs[28,32,41,43,46-50,52,54,60]<br><br>- Negative consequences for own role when referring patients to LIs[31]	- Perception of own role potentially stimulating prescription or follow-up of LIs[32,40,41,46,47,50,52,54,55,60]<br><br>- Positive consequences for own role when referring patients to LIs[31]	
Domain 3: Patient factors			
Health status	- Severity of disease and symptoms[31,43,46,49,51,60]<br><br>- Negative impact of comorbidities[28,38,43,46,47,50,51]<br><br>- Other patient characteristics[46,51,58]	- Severity of disease and symptoms[38,46,52,58,60]<br><br>- Other patient characteristics[40,50,58]	- Severity of disease and symptoms[41,45,52,60]<br><br>- Other patient characteristics[40]
Treatment expectations and preferences	- Negative attitude toward LIs[28,32,33,35,38,40-47,50-52,59,60]<br><br>- Positive attitude toward TJA[36,42,47]	- Make use of patients' preference for TJA within LIs[36]	- Patients' preferences[45]
Active participation	- Low patient adherence or engagement[32,36,40,41,45,46,50,53,60]	- High patient adherence or engagement[33,38,39,53]<br><br>- Importance of high patient adherence or engagement for effectiveness of LIs[29,40,41,46,52,60]	
Capabilities	- Low health literacy[32,36,38-40,42,43,46,48,50,51,59,60]<br><br>- Limited financial resources[40,43]<br><br>- Other responsibilities[40,51]	- High health literacy or importance of education[38,41,42,48,50,59]<br><br>- Social support[39,47]	- Health literacy[45]<br><br>- Other responsibilities[40]
Domain 4: Professional interactions			
Collaboration	- Non-optimal interdisciplinary collaboration or	- Good interdisciplinary collaboration or healthcare provision, or suggestions for improvement[26,31,33,36,40,42,43,45-48,51,52,54-]	

For peer review only - http://bmjopen.bmj.com/site/about/guidelines.xhtml

	healthcare provision[26,31,33,40,42,45,46,48,52,59]	56,58,59,61
	- No access to other HCPs[40]	- Access to other HCPs[31,40-42,45]
Communication and referral	- Lack of communication between HCPs[45,47,59]<br>- Challenges of communication and referral procedures[33,35,43,45,59]	- Improving communication between HCPs[31,33,45,47,51,56]<br>- Needs regarding communication and referral procedures[31,40,45,48,51]
Domain 5: Incentives and resources		
Time	- Lack of time within patient consultations[32,40,42-44,48,52,60]<br>- Lack of time due to other demands (or not further specified)[31,36,40,47,61]	- Adequate duration of patient consultations[32,40]<br>- Adequate duration of specific interventions or protocols[31,44,59,61]
Financial resources	- Limited financial resources within organization[44,47]	- Financial reward for implementing LIs[31,47,59]
Information resources	- Lack of information resources[26,36,43,47]<br>- Challenges in accessing information resources[40,43,52]	- Availability of information resources[26,43,51,56]<br>- Access to information resources[32,40,41,51]
Facilities	- Negative attitude toward information technology[32]	- Potential use of information technology[32,43]<br>- Benefits of working in health centers[48]
Domain 6: Capacity for organizational change		
Professional paradigm		- Adequate professional paradigm or suggestions for expansion[26,40,44]
Monitoring		- Audit[56]
Support within the	- Management not supportive[59]	

For peer review only - http://bmjopen.bmj.com/site/about/guidelines.xhtml

organization		
Domain 7: Social, political, and legal factors		
Healthcare system	- Restrictions due to health insurance[40,47,59]	- Benefits of good health insurance[43,45,59]<br><br>- Government subsidies[32]
Domain 8: Patient and HCP interactions		
Therapeutic alliance	- Potential negative influence of implementing LIs to relationship[36]	- Importance of communication and relationship[38,41,47,48]
Lifestyle as conversation topic	- Challenges of discussing weight[26,32,41,48,49]	- Factors that could ease the way to discussing weight[26,41,43,46,48]
Domain 9: Disease factors		
Image	- OA seen as low priority[28,31,42,45-47]<br><br>- OA seen as untreatable and local condition (wear-and-tear)[32,43,45,46,50,51,60]	- Optimistic views toward OA[32,46]

702 [a] HCP: healthcare professional; LI: lifestyle intervention; OA: osteoarthritis; TJA: total joint arthroplasty.

For peer review only - http://bmjopen.bmj.com/site/about/guidelines.xhtml

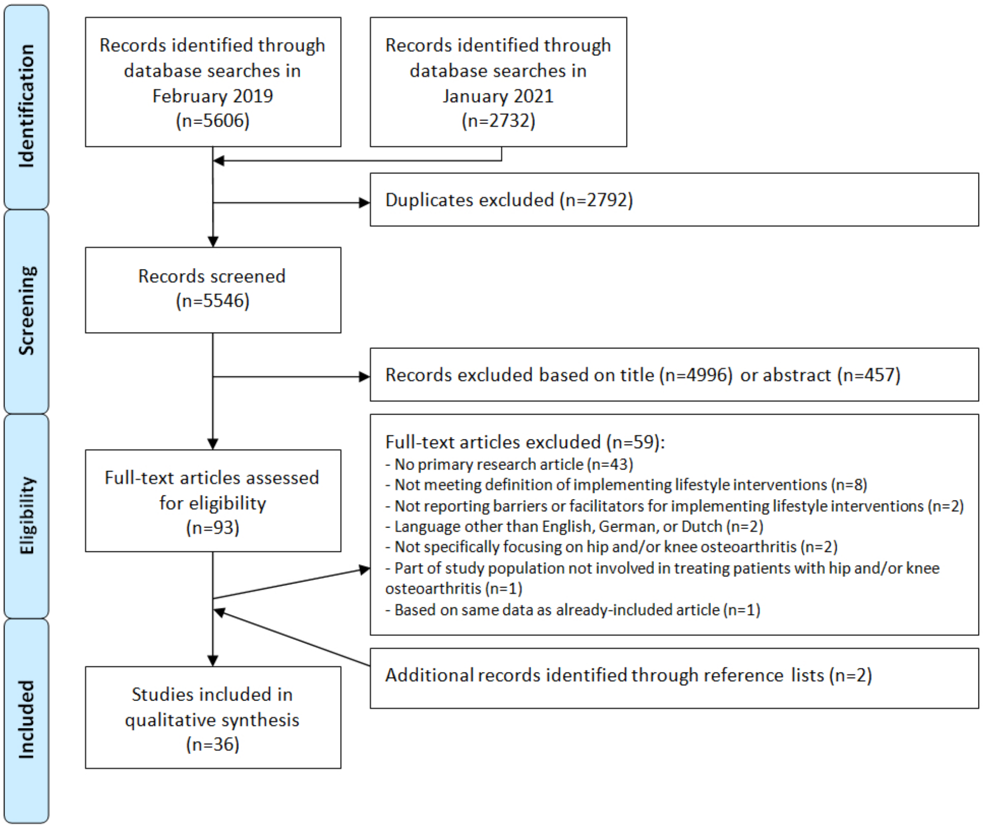

Figure 1. Flowchart of the study selection process

260x217mm (96 x 96 DPI)

Supplemental File 1. Search strategies for the different databases

The search strategies presented below were applied in five bibliographic electronic databases on two different dates: initially on 19 February 2019 and again on 19 January 2021 to update the results. For each database it is shown below which search strategy was used in 2019 (#1) and in 2021 (#2) and how many records were retrieved using these search strategies.

PubMed

#1 (1798 hits)	(((((("Health Personnel"[Mesh] OR "Delivery of Health Care"[Mesh] OR "Primary Health Care"[Mesh]) OR (Health personnel*[tiab] OR Health professional*[tiab] OR Medical staff*[tiab] OR Clinician*[tiab] OR Medical specialist*[tiab] OR Health care*[tiab] OR Healthcare*[tiab] OR Primary care*[tiab] OR Secondary care*[tiab] OR Allied health personnel*[tiab] OR Allied health professional*[tiab] OR Paramedic*[tiab] OR Nurse*[tiab] OR Nursing staff*[tiab] OR Nurse practit*[tiab] OR Physician assistant*[tiab] OR Physicians assistant*[tiab] OR Nutritionist*[tiab] OR Dietician*[tiab] OR Dietitian*[tiab] OR Physical therapist*[tiab] OR Physiotherapist*[tiab] OR Lifestyle coach*[tiab] OR Lifestyle counselor*[tiab] OR Physician*[tiab] OR Orthopedic surgeon*[tiab] OR Orthopedist*[tiab] OR General practitioner*[tiab] OR General practice*[tiab] OR Psychologist*[tiab]))) **AND** ((("Osteoarthritis"[Mesh]) OR (Osteoarthrit*[tiab] OR Osteoarthro*[tiab] OR Degenerative arthrit*[tiab]))) AND ((("Hip Joint"[Mesh] OR "Knee Joint"[Mesh] OR "Lower Extremity"[Mesh]) OR (Hip*[tiab] OR Cox*[tiab] OR Acetabulofemoral joint*[tiab] OR Knee*[tiab] OR Superior tibulofibular joint*[tiab] OR Patellofemoral*[tiab] OR Lower extremit*[tiab] OR Lower limb*[tiab])))) **AND** ((((("Life Style"[Mesh] OR "Behavior"[Mesh]) OR (Life style*[tiab] OR Lifestyle*[tiab] OR Behavior*[tiab] OR Behaviour*[tiab] OR Habit*[tiab] OR Risk reduction*[tiab] OR Early therap*[tiab] OR Secondary prevention*[tiab] OR Tertiary prevention*[tiab] OR Disease prevention*[tiab]))) OR ((("Exercise"[Mesh] OR "Exercise Therapy"[Mesh] OR "Sports"[Mesh]) OR (Physical activit*[tiab] OR Physical training*[tiab] OR Physical fitness*[tiab] OR Physical condition*[tiab] OR Physical therap*[tiab] OR Physiotherap*[tiab] OR Exercis*[tiab] OR Run*[tiab] OR Jog*[tiab] OR Walk*[tiab] OR Bicycl*[tiab] OR Swim*[tiab] OR Strength*[tiab] OR Resistance*[tiab] OR Sport*[tiab] OR Athletic*[tiab] OR Train*[tiab] OR Sedentary[tiab]))) OR ((("Diet, Food, and Nutrition"[Mesh] OR "Nutrition Therapy"[Mesh] OR "Body Weight"[Mesh]) OR (Healthy diet*[tiab] OR Healthy eating*[tiab] OR Nutrition*[tiab] OR Diet*[tiab] OR Food*[tiab] OR Weight loss*[tiab] OR Weight loss program*[tiab] OR Weight reduction*[tiab] OR Weight reduction program*[tiab] OR Body weight*[tiab] OR Overweight*[tiab] OR Obesity*[tiab] OR Overnutrition*[tiab] OR Hypernutrition*[tiab]))))) **AND** ((("Attitude"[Mesh] OR "Motivation"[Mesh]) OR (Barrier*[tiab] OR Facilitator*[tiab] OR Enabler*[tiab] OR Driver*[tiab] OR Motivat*[tiab] OR Opinion*[tiab] OR View*[tiab] OR Attitude*[tiab] OR Expectation*[tiab] OR Incentive*[tiab] OR Disincentive*[tiab] OR Belief*[tiab] OR Influencing factor*[tiab] OR Experience*[tiab] OR Perspective*[tiab] OR Perception*[tiab] OR Hinder*[tiab] OR Impediment*[tiab] OR obstacle*[tiab]))))))
#2 (467 hits)	#1 **AND** (("2019/01/01"[Date - Create] : "3000"[Date - Create]) OR ("2019/01/01"[Date - Entry] : "3000"[Date - Entry]) OR ("2019/01/01"[Date - MeSH] : "3000"[Date - MeSH]))

Embase

#1	(‘health care personnel’/exp OR ‘health care delivery’/exp OR (‘Health personnel*’ OR ‘Health professional*’ OR ‘Medical staff*’ OR ‘Clinician*’ OR

(3036 hits)	'Medical specialist*' OR 'Health care*' OR 'Healthcare*' OR 'Primary care*' OR 'Secondary care*' OR 'Allied health personnel*' OR 'Allied health professional*' OR 'Paramedic*' OR 'Nurse*' OR 'Nursing staff*' OR 'Nurse practit*' OR 'Physician assistant*' OR 'Physicians assistant*' OR 'Nutritionist*' OR 'Dietician*' OR 'Dietitian*' OR 'Physical therapist*' OR 'Physiotherapist*' OR 'Lifestyle coach*' OR 'Lifestyle counselor*' OR 'Physician*' OR 'Orthopedic surgeon*' OR 'Orthopedist*' OR 'General practitioner*' OR 'General practice*' OR 'Psychologist*'):ab,ti)<br>**AND**<br>('osteoarthritis'/exp OR ('Osteoarthrit*' OR 'Osteoarthro*' OR 'Degenerative arthrit*'):ab,ti) AND ('lower limb'/exp OR ('Hip*' OR 'Cox*' OR 'Acetabulofemoral joint*' OR 'Knee*' OR 'Superior tibulofibular joint*' OR 'Patellofemoral*' OR 'Lower extremit*' OR 'Lower limb*'):ab,ti)<br>**AND**<br>(('lifestyle'/exp OR 'lifestyle modification'/exp OR 'behavior'/exp OR ('Life style*' OR 'Lifestyle*' OR 'Behavior*' OR 'Behaviour*' OR 'Habit*' OR 'Risk reduction*' OR 'Early therap*' OR 'Secondary prevention*' OR 'Tertiary prevention*' OR 'Disease prevention*'):ab,ti) OR ('exercise'/exp OR 'kinesiotherapy'/exp OR 'sport'/exp OR ('Physical activit*' OR 'Physical training*' OR 'Physical fitness*' OR 'Physical condition*' OR 'Physical therap*' OR 'Physiotherap*' OR 'Exercis*' OR 'Run*' OR 'Jog*' OR 'Walk*' OR 'Bicycl*' OR 'Swim*' OR 'Strength*' OR 'Resistance*' OR 'Sport*' OR 'Athletic*' OR 'Train*' OR 'Sedentary'):ab,ti) OR ('nutrition'/exp OR 'diet therapy'/exp OR 'body weight'/exp OR 'body weight management'/exp OR ('Healthy diet*' OR 'Healthy eating*' OR 'Nutrition*' OR 'Diet*' OR 'Food*' OR 'Weight loss*' OR 'Weight loss program*' OR 'Weight reduction*' OR 'Weight reduction program*' OR 'Body weight*' OR 'Overweight*' OR 'Obesity*' OR 'Overnutrition*' OR 'Hypernutrition*'):ab,ti))<br>**AND**<br>('attitude'/exp OR 'motivation'/exp OR ('Barrier*' OR 'Facilitator*' OR 'Enabler*' OR 'Driver*' OR 'Motivat*' OR 'Opinion*' OR 'View*' OR 'Attitude*' OR 'Expectation*' OR 'Incentive*' OR 'Disincentive*' OR 'Belief*' OR 'Influencing factor*' OR 'Experience*' OR 'Perspective*' OR 'Perception*' OR 'Hinder*' OR 'Impediment*' OR 'obstacle*'):ab,ti)
#2<br>(1021 hits)	#1 **AND** [1-1-2019]/sd NOT [20-1-2021]/sd

<u>CINAHL</u>

#1<br>(424 hits)	(MH "Health Personnel+" OR MH "Health Care Delivery+" OR TI(Health personnel* OR Health professional* OR Medical staff* OR Clinician* OR Medical specialist* OR Health care* OR Healthcare* OR Primary care* OR Secondary care* OR Allied health personnel* OR Allied health professional* OR Paramedic* OR Nurse* OR Nursing staff* OR Nurse practit* OR Physician assistant* OR Physicians assistant* OR Nutritionist* OR Dietician* OR Dietitian* OR Physical therapist* OR Physiotherapist* OR Lifestyle coach* OR Lifestyle counselor* OR Physician* OR Orthopedic surgeon* OR Orthopedist* OR General practitioner* OR General practice* OR Psychologist*) OR AB(Health personnel* OR Health professional* OR Medical staff* OR Clinician* OR Medical specialist* OR Health care* OR Healthcare* OR Primary care* OR Secondary care* OR Allied health personnel* OR Allied health professional* OR Paramedic* OR Nurse* OR Nursing staff* OR Nurse practit* OR Physician assistant* OR Physicians assistant* OR Nutritionist* OR Dietician* OR Dietitian* OR Physical therapist* OR Physiotherapist* OR Lifestyle coach* OR Lifestyle counselor* OR Physician* OR Orthopedic surgeon* OR Orthopedist* OR General practitioner* OR General practice* OR Psychologist*))<br>**AND**<br>(MH "Osteoarthritis+" OR TI(Osteoarthrit* OR Osteoarthro* OR Degenerative arthrit*) OR AB(Osteoarthrit* OR Osteoarthro* OR Degenerative arthrit*)) AND (MH "Lower Extremity+" OR MH "Hip Joint+" OR MH "Knee Joint+" OR TI(Hip* OR Cox* OR Acetabulofemoral joint* OR Knee* OR Superior tibulofibular joint* OR Patellofemoral* OR Lower extremit* OR Lower limb*) OR AB(Hip* OR Cox* OR Acetabulofemoral joint* OR Knee* OR Superior tibulofibular joint* OR Patellofemoral* OR Lower extremit* OR Lower limb*))

	**AND**<br>((MH "Life Style+" OR MH "Behavior+" OR TI(Life style* OR Lifestyle* OR Behavior* OR Behaviour* OR Habit* OR Risk reduction* OR Early therap* OR Secondary prevention* OR Tertiary prevention* OR Disease prevention*) OR AB(Life style* OR Lifestyle* OR Behavior* OR Behaviour* OR Habit* OR Risk reduction* OR Early therap* OR Secondary prevention* OR Tertiary prevention* OR Disease prevention*)) OR (MH "Exercise+" OR MH "Therapeutic Exercise+" OR MH "Sports+" OR TI(Physical activit* OR Physical training* OR Physical fitness* OR Physical condition* OR Physical therap* OR Physiotherap* OR Exercis* OR Run* OR Jog* OR Walk* OR Bicycl* OR Swim* OR Strength* OR Resistance* OR Sport* OR Athletic* OR Train* OR Sedentary) OR AB(Physical activit* OR Physical training* OR Physical fitness* OR Physical condition* OR Physical therap* OR Physiotherap* OR Exercis* OR Run* OR Jog* OR Walk* OR Bicycl* OR Swim* OR Strength* OR Resistance* OR Sport* OR Athletic* OR Train* OR Sedentary)) OR (MH "Nutrition+" OR MH "Food+" OR MH "Diet Therapy+" OR MH "Body Weight+" OR TI(Healthy diet* OR Healthy eating* OR Nutrition* OR Diet* OR Food* OR Weight loss* OR Weight loss program* OR Weight reduction* OR Weight reduction program* OR Body weight* OR Overweight* OR Obesity* OR Overnutrition* OR Hypernutrition*) OR AB(Healthy diet* OR Healthy eating* OR Nutrition* OR Diet* OR Food* OR Weight loss* OR Weight loss program* OR Weight reduction* OR Weight reduction program* OR Body weight* OR Overweight* OR Obesity* OR Overnutrition* OR Hypernutrition*)))<br>**AND**<br>(MH "Attitude+" OR MH "Motivation+" OR TI(Barrier* OR Facilitator* OR Enabler* OR Driver* OR Motivat* OR Opinion* OR View* OR Attitude* OR Expectation* OR Incentive* OR Disincentive* OR Belief* OR Influencing factor* OR Experience* OR Perspective* OR Perception* OR Hinder* OR Impediment* OR Obstacle*) OR AB(Barrier* OR Facilitator* OR Enabler* OR Driver* OR Motivat* OR Opinion* OR View* OR Attitude* OR Expectation* OR Incentive* OR Disincentive* OR Belief* OR Influencing factor* OR Experience* OR Perspective* OR Perception* OR Hinder* OR Impediment* OR Obstacle*))
#2<br>(598 hits)	#1

PsycINFO

#1<br>(49 hits)	(DE "Health Personnel" OR DE "Health Care Delivery" OR DE "Primary Health Care" OR DE "Physical Therapists" OR DE "Nurses" OR DE "Physicians" OR DE "Family Physicians" OR DE "General Practitioners" OR DE "Surgeons" OR DE "Clinical Psychologists" OR DE "Allied Health Personnel" OR DE "Caregivers" OR DE "Medical Personnel" OR DE "Mental Health Personnel" OR DE "Clinicians" OR DE "Therapists" OR DE "Psychologists" OR TI(Health personnel* OR Health professional* OR Medical staff* OR Clinician* OR Medical specialist* OR Health care* OR Healthcare* OR Primary care* OR Secondary care* OR Allied health personnel* OR Allied health professional* OR Paramedic* OR Nurse* OR Nursing staff* OR Nurse practit* OR Physician assistant* OR Physicians assistant* OR Nutritionist* OR Dietician* OR Dietitian* OR Physical therapist* OR Physiotherapist* OR Lifestyle coach* OR Lifestyle counselor* OR Physician* OR Orthopedic surgeon* OR Orthopedist* OR General practitioner* OR General practice* OR Psychologist*) OR AB(Health personnel* OR Health professional* OR Medical staff* OR Clinician* OR Medical specialist* OR Health care* OR Healthcare* OR Primary care* OR Secondary care* OR Allied health personnel* OR Allied health professional* OR Paramedic* OR Nurse* OR Nursing staff* OR Nurse practit* OR Physician assistant* OR Physicians assistant* OR Nutritionist* OR Dietician* OR Dietitian* OR Physical therapist* OR Physiotherapist* OR Lifestyle coach* OR Lifestyle counselor* OR Physician* OR Orthopedic surgeon* OR Orthopedist* OR General practitioner* OR General practice* OR Psychologist*))<br>**AND**<br>(TI(Osteoarthrit* OR Osteoarthro* OR Degenerative arthrit*) OR AB(Osteoarthrit* OR Osteoarthro* OR Degenerative arthrit*)) AND (DE "Hips" OR DE "Knee" OR TI(Hip* OR Cox* OR Acetabulofemoral joint* OR Knee* OR Superior tibulofibular joint* OR Patellofemoral* OR Lower extremit* OR Lower limb*) OR AB(Hip* OR Cox* OR Acetabulofemoral joint* OR Knee* OR Superior tibulofibular joint* OR Patellofemoral* OR Lower extremit* OR Lower

For peer review only

	limb*))<br>**AND**<br>((DE “Lifestyle” OR DE “Behavior” OR DE "Active Living" OR DE "Lifestyle Changes" OR DE "Activity Level” OR DE "Habits” OR DE "Behavior Change" OR DE "Readiness to Change" OR DE "Stages of Change" OR DE "Illness Behavior" OR DE "Health Behavior" OR DE "Health Attitudes" OR TI(Life style* OR Lifestyle* OR Behavior* OR Behaviour* OR Habit* OR Risk reduction* OR Early therap* OR Secondary prevention* OR Tertiary prevention* OR Disease prevention*) OR AB(Life style* OR Lifestyle* OR Behavior* OR Behaviour* OR Habit* OR Risk reduction* OR Early therap* OR Secondary prevention* OR Tertiary prevention* OR Disease prevention*)) OR (DE “Exercise” OR DE “Movement Therapy” OR DE “Sports” OR DE "Aerobic Exercise" OR DE "Physical Activity" OR DE "Physical Fitness" OR DE "Rehabilitation" OR DE "Physical Therapy" OR DE "Sedentary Behavior" OR TI(Physical activit* OR Physical training* OR Physical fitness* OR Physical condition* OR Physical therap* OR Physiotherap* OR Exercis* OR Run* OR Jog* OR Walk* OR Bicycl* OR Swim* OR Strength* OR Resistance* OR Sport* OR Athletic* OR Train* OR Sedentary) OR AB(Physical activit* OR Physical training* OR Physical fitness* OR Physical condition* OR Physical therap* OR Physiotherap* OR Exercis* OR Run* OR Jog* OR Walk* OR Bicycl* OR Swim* OR Strength* OR Resistance* OR Sport* OR Athletic* OR Train* OR Sedentary)) OR (DE “Nutrition” OR DE “Food” OR DE “Diets” OR DE “Body Weight” OR DE "Weight Control" OR DE "Calories" OR DE "Body Mass Index" OR DE "Overweight" OR DE "Weight Loss" OR DE "Obesity" OR TI(Healthy diet* OR Healthy eating* OR Nutrition* OR Diet* OR Food* OR Weight loss* OR Weight loss program* OR Weight reduction* OR Weight reduction program* OR Body weight* OR Overweight* OR Obesity* OR Overnutrition* OR Hypernutrition*) OR AB(Healthy diet* OR Healthy eating* OR Nutrition* OR Diet* OR Food* OR Weight loss* OR Weight loss program* OR Weight reduction* OR Weight reduction program* OR Body weight* OR Overweight* OR Obesity* OR Overnutrition* OR Hypernutrition*)))<br>**AND**<br>(DE “Attitudes” OR DE “Motivation” OR DE "Health Personnel Attitudes" OR DE "Therapist Attitudes" OR DE "Psychologist Attitudes" OR DE "Extrinsic Motivation" OR DE "Intrinsic Motivation" OR TI(Barrier* OR Facilitator* OR Enabler* OR Driver* OR Motivat* OR Opinion* OR View* OR Attitude* OR Expectation* OR Incentive* OR Disincentive* OR Belief* OR Influencing factor* OR Experience* OR Perspective* OR Perception* OR Hinder* OR Impediment* OR Obstacle*) OR AB(Barrier* OR Facilitator* OR Enabler* OR Driver* OR Motivat* OR Opinion* OR View* OR Attitude* OR Expectation* OR Incentive* OR Disincentive* OR Belief* OR Influencing factor* OR Experience* OR Perspective* OR Perception* OR Hinder* OR Impediment* OR Obstacle*))
#2<br>(14 hits)	#1 **AND** RD 20190101-

<u>The Cochrane Library</u>

#1<br>(299 hits)	((Health personnel* OR Health professional* OR Medical staff* OR Clinician* OR Medical specialist* OR Health care* OR Healthcare* OR Primary care* OR Secondary care* OR Allied health personnel* OR Allied health professional* OR Paramedic* OR Nurse* OR Nursing staff* OR Nurse practit* OR Physician assistant* OR Physicians assistant* OR Nutritionist* OR Dietician* OR Dietitian* OR Physical therapist* OR Physiotherapist* OR Lifestyle coach* OR Lifestyle counselor* OR Physician* OR Orthopedic surgeon* OR Orthopedist* OR General practitioner* OR General practice* OR Psychologist*)<br>**AND**<br>(Osteoarthrit* OR Osteoarthro* OR Degenerative arthrit*) AND (Hip* OR Cox* OR Acetabulofemoral joint* OR Knee* OR Superior tibulofibular joint* OR Patellofemoral* OR Lower extremit* OR Lower limb*)<br>**AND**<br>((Life style* OR Lifestyle* OR Behavior* OR Behaviour* OR Habit* OR Risk reduction* OR Early therap* OR Secondary prevention* OR Tertiary

	prevention* OR Disease prevention*) OR (Physical activit* OR Physical training* OR Physical fitness* OR Physical condition* OR Physical therap* OR Physiotherap* OR Exercis* OR Run* OR Jog* OR Walk* OR Bicycl* OR Swim* OR Strength* OR Resistance* OR Sport* OR Athletic* OR Train* OR Sedentary) OR (Healthy diet* OR Healthy eating* OR Nutrition* OR Diet* OR Food* OR Weight loss* OR Weight loss program* OR Weight reduction* OR Weight reduction program* OR Body weight* OR Overweight* OR Obesity* OR Overnutrition* OR Hypernutrition*))<br>**AND**<br>(Barrier* OR Facilitator* OR Enabler* OR Driver* OR Motivat* OR Opinion* OR View* OR Attitude* OR Expectation* OR Incentive* OR Disincentive* OR Belief* OR Influencing factor* OR Experience* OR Perspective* OR Perception* OR Hinder* OR Impediment* OR Obstacle*)):ti,ab,kw
#2<br>(632 hits)	#1

For peer review only

Supplemental File 2. Quality assessment of the included studies based on the Mixed Methods Appraisal Tool[a]

Reference[b]	Screening questions[c]		1. Qualitative[d]					4. Quantitative descriptive[e]					5. Mixed-methods[f]				
	S1	S2	1.1	1.2	1.3	1.4	1.5	4.1	4.2	4.3	4.4	4.5	5.1	5.2	5.3	5.4	5.5
Allison (2019) [26]	+	+	+	+	?	+	+										
Bossen (2016) [27][g]	+	+	?	+	?	-	?										
Christiansen (2020) [28]	+	+	?	+	?	+	+										
Davis (2018) [29]	+	+	?	+	?	-	?										
De Rooij (2014) [30]	+	+	?	+	?	-	?										
Egerton (2017) [31]	+	+	?	+	?	+	+										
Egerton (2018) [32]	+	+	?	+	?	+	+										
Hinman (2016) [33]	+	+	+	+	+	+	+										
Hinman (2017) [34]	+	+	?	+	?	+	+										
Knoop (2020) [35]	+	+	?	+	?	-	?										
Law (2019) [36]	+	+	?	+	?	+	+										
Lawford (2019) [37]	+	+	?	+	?	+	+										
Lawford (2020) [38]	+	+	?	+	?	+	+										
Lawford (2021) [39]	+	+	?	+	?	+	+										
MacKay (2018) [40]	+	+	?	+	?	+	+										
MacKay (2020) [41]	+	+	?	+	?	+	+										
Mann (2011) [42]	+	+	?	+	?	+	+										
Miller (2020) [43]	+	+	?	+	?	-	+										
Nielsen (2014) [44]	+	+	?	+	?	+	+										
Okwera (2019) [45]	+	+	?	+	?	+	+										
Poitras (2010) [46]	+	+	?	+	?	-	+										
Rosemann (2006) [47]	+	+	?	+	?	-	?										
Selten (2017) [48]	+	+	?	+	?	+	+										
Tang (2020) [49]	+	+	?	+	?	+	+										
Teo (2020) [50]	+	+	?	+	?	+	+										
Wallis (2020) [51]	+	+	?	+	?	+	+										
Cottrell (2016) [52]	+	+						+	+	+	-	+					
Duarte (2019) [53]	+	+						+	?	+	+	?					
Hill (2018) [54]	+	+						+	?	+	-	+					
Hill (2018) [55]	+	+						-	?	+	?	+					
Hofstede (2016) [56]	+	+						+	?	+	-	+					
Lawford (2018) [57]	+	+						+	?	+	?	+					

For peer review only

Reid (2014) [58]	+	+						+	?	+	-	+					
De Rooij (2020) [59]	+	+	?	+	?	-	?	+	?	+	+	+	?	+	?	+	-
Holden (2009) [60]	+	+	?	+	?	+	+	+	?	+	-	+	+	+	+	+	-
Kloek (2020) [61]	+	+	?	+	?	+	+	+	?	+	-	+	+	+	?	-	-

[a] + = yes; - = no, ? = can't tell

[b] The numbers in brackets ('[…]') correspond to the reference numbers used in the main text of the manuscript.

[c] S1 = Are there clear research questions?; S2 = Do the collected data allow to address the research questions?

[d] 1.1 = Is the qualitative approach appropriate to answer the research question?; 1.2 = Are the qualitative data collection methods adequate to address the research question?; 1.3 = Are the findings adequately derived from the data?; 1.4 = Is the interpretation of results sufficiently substantiated by data?; 1.5 = Is there coherence between qualitative data sources, collection, analysis and interpretation?

[e] 4.1 = Is the sampling strategy relevant to address the research question?; 4.2 = Is the sample representative of the target population?; 4.3 = Are the measurements appropriate?; 4.4 = Is the risk of nonresponse bias low?; 4.5 = Is the statistical analysis appropriate to answer the research question?

[f] 5.1 = Is there an adequate rationale for using a mixed methods design to address the research question?; 5.2 = Are the different components of the study effectively integrated to answer the research question?; 5.3 = Are the outputs of the integration of qualitative and quantitative components adequately interpreted?; 5.4 = Are divergences and inconsistencies between quantitative and qualitative results adequately addressed?; 5.5 = Do the different components of the study adhere to the quality criteria of each tradition of the methods involved?

[g] The MMAT was only applied to the section regarding the pilot study on the feasibility of the blended intervention.

For peer review only

Supplemental File 3. Full overview of all extracted factors per domain

Explanation

- In the tables below, all extracted factors per subcategory of each domain are presented. The colors of the subcategories correspond to barriers (red), facilitators (green) or unclear factors (orange).
- Column "Description": (*) at the end of the description indicates that the factor is derived from a close-ended question or attitude statement.
- Column "Reference": the numbers in brackets ('[…]') correspond to the reference numbers used in the main text of the manuscript.
- Abbreviations: ACSM: American College of Sports Medicine; BMI: body mass index; CBT: cognitive behavioral therapy; CKP: chronic knee pain; CPG: clinical practice guideline; GP: general practitioner; HCP: healthcare professional; LI: lifestyle intervention; LMP: Lifestyle Management Programme; NSAID: non-steroidal anti-inflammatory drug; NWBE: non-weight bearing quadriceps strengthening exercise; OA: osteoarthritis; PCST: pain coping skills training; PT: physiotherapist; RCT: randomized controlled trial; TKA: total knee arthroplasty; TJA: total joint arthroplasty; WBE: weight bearing functional exercise.

Domain 1: Intervention factors

Effectiveness

Description	Reference
LIs have little or no effect on OA (barrier)	
Not certain that exercise works	Christiansen (2020) [28]
Advice to exercise and lose weight does not work	Egerton (2017) [31]
Dubious about effect of exercise and weight-management advice on reducing symptoms	Egerton (2018) [32]
Surgical methods have the best outcomes	Miller (2020) [43]
Lack of confidence in clinical effectiveness of physiotherapy treatments	Okwera (2019) [45]
Limited impact of weight loss on established knee OA (more effective as a primary prevention strategy)	Poitras (2010) [46]
Questioning direct relationship between weight and knee OA (numerous other factors associated)	Poitras (2010) [46]
Less certain about effectiveness of physical therapy (benefits variable or difficult to prove)	Selten (2017) [48]
Increasing the overall activity levels does not/might not stop the knee problem getting worse (*)	Cottrell (2016) [52]
There is a paucity of evidence in regards to the effectiveness of physiotherapy treatment for OA hip and/or knee (*)	Reid (2014) [58]
Past experience has shown physiotherapy to be ineffective (*)	Reid (2014) [58]
Increasing overall activity levels does not/might not stop the knee problem getting worse (*)	Holden (2009) [60]
Increasing the strength of the muscles around the knee does not/might not stop the knee problem getting worse (*)	Holden (2009) [60]
Knee problems are not/might not be improved by general exercise (*)	Holden (2009) [60]
Potential effects of LIs are difficult to accomplish (barrier)	
Weight loss is difficult (multiplicity of factors need to be addressed, often involving change in lifestyle)	Poitras (2010) [46]

For peer review only - http://bmjopen.bmj.com/site/about/guidelines.xhtml

Vicious circle (pain when exercising, people move less/eat more due to frustration/sometimes depression)	Rosemann (2006) [47]
Achieving patient behavior change is difficult (*)	Cottrell (2016) [52]
Human nature (as patient-centered barrier to adherence)	Holden (2009) [60]
LIs have positive effects on affected joint(s) (facilitator)	
Functional improvements experienced by patients	Hinman (2017) [34]
Improvements in patient pain and function	Lawford (2019) [37]
Large improvements in knee pain	Lawford (2021) [39]
Treatment could improve clients' symptoms (e.g. reduce pain, increase function)	MacKay (2018) [40]
Treatment could potentially slow progression of symptoms	MacKay (2018) [40]
Improve people's symptoms early in treatment (to gain buy-in)	MacKay (2020) [41]
Benefits of activity on knee mobility	Poitras (2010) [46]
Activity necessary for the knee's health	Poitras (2010) [46]
Weight loss improves pain and joint function	Poitras (2010) [46]
Benefits of weight reduction for relieving symptoms of knee/hip OA	Selten (2017) [48]
Beneficial effects of physical therapy in reducing pain/stiffness and potential effects on cartilage	Selten (2017) [48]
Exercise therapy may be effective by giving more muscular support for joints	Wallis (2020) [51]
Knee problems are improved by quadriceps strengthening exercises (*)	Cottrell (2016) [52]
Knee problems are improved by general exercise (e.g. walking or swimming) (*)	Cottrell (2016) [52]
Increasing the strength of the muscles around the knee stops the knee problem getting worse (*)	Cottrell (2016) [52]
Exercise is beneficial for OA (*)	Lawford (2018) [57]
Knee problems are improved by local strengthening exercises (*)	Holden (2009) [60]
LIs have positive effects on general health (facilitator)	
Lifestyle treatments benefited other chronic conditions	Egerton (2018) [32]
Rapid weight loss was primary driver of motivation	Lawford (2021) [39]
Benefits of activity on general wellbeing	Poitras (2010) [46]
Weight loss effective at improving mobility in general	Poitras (2010) [46]
Weight loss also benefits mobility in general	Poitras (2010) [46]
Beneficial effects of physical therapy in reducing weight and for increasing mobility/posture/coordination	Selten (2017) [48]
Community interventions are effective at achieving sufficient and sustained weight loss (*)	Hill (2018) [55]
Patients benefit from weight loss (*)	Hofstede (2016) [56]
LIs have positive mental effects (facilitator)	
Enthusiastic about the program and described the results (e.g. it was empowering)	Davis (2018) [29]
Greater confidence to exercise among patients	Hinman (2017) [34]
Reminding patients of opportunity to self-manage	Law (2019) [36]
Increased confidence to self-manage	Lawford (2019) [37]
Positive lifestyle changes (patients) (e.g. thinking differently)	Lawford (2021) [39]
Physical therapy useful in increasing patients self-management in coping with/acceptance of symptoms	Selten (2017) [48]

For peer review only

Exercise therapy may be effective by giving opportunity to improve confidence about activities/mobility	Wallis (2020) [51]
Non-surgical treatments motivate patients to do things themselves (*)	Hofstede (2016) [56]
LIs have positive effects (not further specified) (facilitator)	
Positive impact on patients of personalized attention from coach and from advice/education they provided	Hinman (2016) [33]
Emphasising health benefits of programme	Law (2019) [36]
Physical therapy helpful for patients most of the time	Miller (2020) [43]
Value of lifestyle advice related to knee and hip OA	Selten (2017) [48]
Non-pharmacological, non-surgical treatment was considered useful to delay surgery	Selten (2017) [48]
Positive about program (alternative approach and opportunity to avoid a joint replacement)	Wallis (2020) [51]
Improvement in the physical condition of participants	Duarte (2019) [53]
Good results of physical therapy (*)	Hofstede (2016) [56]

Safety

**Description**	**Reference**
LIs are unsafe or have negative effects (barrier)	
Being apprehensive about aggravating pain in patients	Lawford (2020) [38]
Tending to avoid pushing patients in usual clinical practice	Lawford (2020) [38]
Potential further damage to the knee due to activity	Poitras (2010) [46]
Urging caution to patients about participating in higher impact exercise/activities	Wallis (2020) [51]
Fear of increasing symptoms (as barrier to prescribing exercise)	Holden (2009) [60]
Causing disease progression, particularly through weight-bearing activities (as barrier to prescribing exercise)	Holden (2009) [60]
Exacerbating patient's comorbidities (as barrier to prescribing exercise)	Holden (2009) [60]
General exercise is not/might not be safe for everybody to do (*)	Holden (2009) [60]
Local strengthening exercises for the knee are not/might not be safe for everybody to do (*)	Holden (2009) [60]
LIs are safe (facilitator)	
Quadriceps strengthening exercises for the knee are safe for everybody to do (*)	Cottrell (2016) [52]
General exercise (e.g. walking or swimming) is safe for everybody to do (*)	Cottrell (2016) [52]
Only few drawbacks for the use of non-surgical treatments (*)	Hofstede (2016) [56]
Research environment or protocols provide a safety net (facilitator)	
Less afraid to increase training intensity (preventing adverse events by tailoring programs to individual's capacity)	De Rooij (2014) [30]
Safety net provided by research environment (e.g. patients were previously screened for comorbidities/red flags)	Hinman (2017) [34]
There was a safety net in place with the trial (each patient had been screened)	Lawford (2019) [37]
Experiences in study helped them push patients through more pain than they would have previously	Lawford (2020) [38]

Design

For peer review only

Description	Reference
Non-optimal content or structure of LIs (barrier)	
Structure/timing of exercise program restricted capacity to modify exercises/provide adequate follow-up	Hinman (2016) [33]
Maximum number of four sessions was considered too low in many patients	Knoop (2020) [35]
Behavioral approach in exercise therapy and advice to visit GP were considered unnecessary for most patients	Knoop (2020) [35]
Program factors (e.g. single discipline led intervention)	Wallis (2020) [51]
Services do not meet expectations (*)	Cottrell (2016) [52]
E-Exercise does not/might not contain all essential elements for the treatment of hip/knee OA (*)	Kloek (2020) [61]
The content of e-Exercise is not/might not be aligned with my opinion about treating patients with OA (*)	Kloek (2020) [61]
The intervention provided through e-Exercise is not/might not be appropriate for the average patient with OA (*)	Kloek (2020) [61]
Patients who were treated with e-Exercise were (perhaps) not generally positive about the intervention (*)	Kloek (2020) [61]
Challenges for patients during participation in LIs (barrier)	
Mental effort required for WBE program was challenging for patients	Lawford (2020) [38]
Physical challenge was the complexity of WBE program	Lawford (2020) [38]
Straight leg raise challenging in NWBE program	Lawford (2020) [38]
Volume of resources could be overwhelming/confusing for some patients	Lawford (2021) [39]
Difficulty for patients with PCST component (cognitive restructuring techniques)	Nielsen (2014) [44]
Challenges for HCPs during delivery of LIs (barrier)	
e-Exercise must be adapted for suitable integration into practice (e.g. no insight into modules patients receive)	Bossen (2016) [27]
Class required intense supervision, which was difficult to provide when most participants were new	Davis (2018) [29]
Challenges of supervision when space did not allow clear line of sight	Davis (2018) [29]
Challenges associated with cuff weights used to apply resistance in NWBE program	Lawford (2020) [38]
The lay out of the protocol does not/might not facilitate its usage in daily practice (*)	De Rooij (2020) [59]
Clarity of instruction manual and course (lack of)	Kloek (2020) [61]
Positive experiences with or suggestions for improving the content or structure of LIs (facilitator)	
Positive feedback regarding the content of e-Exercise	Bossen (2016) [27]
First education session was critical to reducing the participant's anxiety related to exercising	Davis (2018) [29]
Importance of empowering the patients rather than 'pushing' them, achieved by 'giving choices'	Davis (2018) [29]
Positive comments about the exercise regimen	Hinman (2016) [33]
Structure provided by protocol/structure of exercises (how patients included them into daily routine)	Hinman (2016) [33]
Multidisciplinary nature of LMP (whole-person, intensive and functional approach)	Law (2019) [36]
Standardization was viewed as important for monitoring and evaluation purposes	Law (2019) [36]
Helpful social impact of group-based programme	Law (2019) [36]
Long-term follow-up consultations would be beneficial	Lawford (2021) [39]
Extremely positive about educational resources provided	Lawford (2021) [39]
More information about healthy eating beyond meal replacement phase could be included	Lawford (2021) [39]
Exercise/physical activity program was an important part of intervention	Lawford (2021) [39]

For peer review only

Favorably comments on program content (positive way to help people be proactive about their pain)	Nielsen (2014) [44]
Importance of PCST component (cognitive restructuring techniques)	Nielsen (2014) [44]
Structure of PCST sessions (overview/practice review/covering new skill/practice planning) worked well	Nielsen (2014) [44]
A more holistic program as part of a multidisciplinary model of service was preferred	Wallis (2020) [51]
Value of program's structure and peer (group) support	Wallis (2020) [51]
Name of program ('Good Life with OsteoArthritis') implied optimism and positive outcome	Wallis (2020) [51]
Received positive feedback from their patients about program	Wallis (2020) [51]
Some contents of the protocol are not/might not be incorrect (*)	De Rooij (2020) [59]
The protocol is applicable to OA patients with comorbidity that I see in my clinical practice (*)	De Rooij (2020) [59]
Important to extend the intake phase to at least to 45 min	De Rooij (2020) [59]
Completeness of web-based application (exercises/assignments/information)	Kloek (2020) [61]
Perception that e-Exercise is an appropriate treatment option for subgroup of OA patients	Kloek (2020) [61]
Ease for patients during participation in LIs (facilitator)	
NWBE program was generally easier for patients to follow (mental effort)	Lawford (2020) [38]
Simplicity and convenience of meal replacements	Lawford (2021) [39]
Providing trial of sessions to assist patients to get started (suggestion for promotion and referrals)	Wallis (2020) [51]
Ease for HCPs during delivery of LIs (facilitator)	
Initial classes needed to be small with rolling recruitment very beneficial	Davis (2018) [29]
List of restrictions for exercise therapy was conveniently arranged checklist for diagnostic and treatment phases	De Rooij (2014) [30]
Suggestion to increase feasibility by reducing the protocols to three main protocols	De Rooij (2014) [30]
Requirements of treatment protocol freed therapists to notice and reflect on impact of the interventions	Hinman (2016) [33]
Structured protocol allowed to experience different OA treatment regimen/observe and learn from impact	Hinman (2016) [33]
NWBE program was easier to prescribe (mental effort)	Lawford (2020) [38]
Easier to prescribe and progress NWBE than WBE program (physical complexity)	Lawford (2020) [38]
Training workshop as good introduction to content and process of delivering PCST program	Nielsen (2014) [44]
Weekly group interaction crucial to being able to deliver intervention effectively/problem-solve issues	Nielsen (2014) [44]
Input from supervising psychologist crucial to being able to deliver intervention effectively/problem-solve issues	Nielsen (2014) [44]
Would have liked more role-playing experience prior to beginning trial treatments	Nielsen (2014) [44]
Regular group meetings were considered very important (if not essential) for delivery of PCST program	Nielsen (2014) [44]
Value of having a psychologist involved throughout the program, their professional input was helpful	Nielsen (2014) [44]
The recommendations over adapting the diagnostic phase (history taking and physical examination) in the protocol are clear and understandable (*)	De Rooij (2020) [59]
The recommendations over adapting the OA exercise therapy in the protocol are clear and understandable (*)	De Rooij (2020) [59]
Useful to plan follow up/refreshment training to repeat/discuss content of course/protocol and application	De Rooij (2020) [59]
Shortening the protocol would increase user-friendliness	De Rooij (2020) [59]
The instruction course and manual assisted me so that I knew how to work with e-Exercise (*)	Kloek (2020) [61]

Personalized treatment

For peer review only

Description	Reference
Insufficient ability to provide personalized treatment within LIs (barrier)	
Concerned the service would not be able to provide individualized management for a very diverse population	Egerton (2017) [31]
Requirements of RCT potentially created a barrier to responding to where the client was	Nielsen (2014) [44]
Less satisfied about the applicability of e-Exercise for only one diagnosis	Kloek (2020) [61]
I do not/might not have enough influence on the content of patients' individual e-Exercise program (*)	Kloek (2020) [61]
Ability and importance of providing personalized treatment within LIs (facilitator)	
Flexibility was valuable when tackling local participation challenges	Law (2019) [36]
Tailoring exercise programs to individual patient would overcome some challenges	Lawford (2020) [38]
Tailoring treatment to a person's goals/interests	MacKay (2020) [41]
Need to consider personal context by integrating people's home exercises into daily activities/other life demands	MacKay (2020) [41]
Some modules worked better than others (depending on the individual patient and context)	Nielsen (2014) [44]
The belief that a more flexible approach responsive to patient needs was required in their practice	Nielsen (2014) [44]
Exercise programs have to be individualized to each patient by the PT	Poitras (2010) [46]
Importance of tailored exercise program	Teo (2020) [50]
Exercise for CKP is most beneficial when it is tailored to meet individual patient needs (*)	Cottrell (2016) [52]
A standard set of exercises is not/might not be sufficient for every patient with chronic knee problems (*)	Cottrell (2016) [52]
The protocol gives the opportunity to make your own decisions regarding history taking, physical examination, and treatment (*)	De Rooij (2020) [59]
A standard set of exercises is not/might not be sufficient for every patient with knee OA (*)	Holden (2009) [60]
Exercise for knee OA is most beneficial when it is tailored to meet individual patient needs (*)	Holden (2009) [60]
More flexibility in web-based application (intervention duration, number of sets/repetitions, type of exercises)	Kloek (2020) [61]
More flexibility in intervention (more possibilities to personalize to individual needs)	Kloek (2020) [61]

Accessibility

Description	Reference
LIs are unavailable or inaccessible (barrier)	
Most of the patients did not meet study inclusion criteria	Bossen (2016) [27]
Lack of availability of support services (e.g. community-based rehabilitation programs) in remote locations	Egerton (2018) [32]
Long waiting lists for support services (e.g. community-based rehabilitation programs)	Egerton (2018) [32]
Lack of infrastructure or local programmes (particularly in rural settings)	MacKay (2018) [40]
Clients often had a waiting period before accessing care	MacKay (2018) [40]
Lack of facilities to promote continuing exercise in community	Mann (2011) [42]
Wait for physiotherapy was too long	Mann (2011) [42]
Inaccessible treatment options within organization	Miller (2020) [43]
Limitations to accessing services (e.g. lack of facilities, costs) (*)	Cottrell (2016) [52]

For peer review only - http://bmjopen.bmj.com/site/about/guidelines.xhtml

Weight management services are not/might not be adequately commissioned in my area (*)	Hill (2018) [55]
Lack of availability of physiotherapy	Reid (2014) [58]
Poor links to community facilities such as local leisure centres	Holden (2009) [60]
Costs of LIs to patients (barrier)	
Concern that uptake would be negatively impacted if patients were required to pay	Egerton (2017) [31]
Concerns regarding financial cost to patients when considering referral to other services	Egerton (2018) [32]
Cost was a factor in whether clients could access facilities/programmes	MacKay (2018) [40]
Costs to patients (lack of insurance coverage/high co-pays for specific services/time off work/travel expenses)	Miller (2020) [43]
Costs (extrinsic barrier for patient adherence)	Teo (2020) [50]
Cost (program access barrier)	Wallis (2020) [51]
LIs are not feasible or sustainable (barrier)	
Concern for overcomplicated system when service is not compatible/complementary with existing initiatives	Egerton (2017) [31]
Not seeing need (already adequate skills/resources to support OA patient self-management and lifestyle change)	Egerton (2017) [31]
Not seeing need (advice already given at their practice would be unhelpfully repeated)	Egerton (2017) [31]
Concern regarding long-term service sustainability	Egerton (2017) [31]
The addition of a care support team may add complexities to management	Egerton (2017) [31]
In my daily clinical practice I can (perhaps) not integrate working according to the protocol well (*)	De Rooij (2020) [59]
I do not/might not treat enough patients with knee OA and comorbidity to apply the protocol (*)	De Rooij (2020) [59]
The protocol does not/might not fit well with my working methods of daily clinical practice (*)	De Rooij (2020) [59]
Total amount of knee OA patients with comorbidity was lower than expected	De Rooij (2020) [59]
Inconvenience to patients when accessing LIs (barrier)	
Weather (extrinsic barrier for patient adherence)	Teo (2020) [50]
Transport, waiting time and parking related to attendance (program access barrier)	Wallis (2020) [51]
Geography (program access barrier)	Wallis (2020) [51]
Available session times (program access barrier)	Wallis (2020) [51]
Geographical problems (e.g. remote location, scared to walk in local area) (*)	Cottrell (2016) [52]
LIs are available or accessible, or suggestions for improvement (facilitator)	
More likely to engage with the care support team if it enabled more affordable/accessible allied health	Egerton (2017) [31]
LMP would benefit from extension of inclusion criteria (patients with less severe OA and lower BMI)	Law (2019) [36]
Benefits of having infrastructure and programmes available in their communities	MacKay (2018) [40]
Triage service (as suggestion for physiotherapy service improvement)	Okwera (2019) [45]
A web-based physiotherapy service (as suggestion for physiotherapy service improvement)	Okwera (2019) [45]
Reduced waiting times (as suggestion for physiotherapy service improvement)	Okwera (2019) [45]
Availability of non-surgical treatments (*)	Hofstede (2016) [56]
Good access to physiotherapy in area (*)	Reid (2014) [58]
LIs are feasible or sustainable (facilitator)	
Need for clarity about how the new service would integrate with existing schemes and payment structures	Egerton (2017) [31]

For peer review only

Seeing need (advice/recommendations may need to be reinforced/provided over several health care episodes)	Egerton (2017) [31]
Seeing need (extra time and encouragement for the patient would result in better outcomes)	Egerton (2017) [31]
Importance of broad acceptance (patients/doctors/health service funders) if new service is to continue long term	Egerton (2017) [31]
Model of stratified care easy to apply and having added value for daily practice	Knoop (2020) [35]
Appreciation of applicability of treatment protocols	Knoop (2020) [35]
Further and ongoing evaluation of the LMP would help to address current challenges	Law (2019) [36]
Interventions in physical therapists' toolbox were not static (changed over time)	MacKay (2020) [41]
The protocol is feasible in daily clinical practice (*)	De Rooij (2020) [59]
In my daily clinical practice, I work with sufficient equipment (including blood pressure meter, saturation meter) to properly apply the protocol (*)	De Rooij (2020) [59]
I have changed my working method (due to the protocol) (*)	De Rooij (2020) [59]
Intake procedure is feasible and implementable	De Rooij (2020) [59]
The more you apply the strategy in daily practice, the easier it is to integrate it in your daily working method	De Rooij (2020) [59]
Convenience for patients when accessing LIs (facilitator)	
Close, convenient locations (suggestion for promotion and referrals)	Wallis (2020) [51]
Appropriate session times for working populations (suggestion for promotion and referrals)	Wallis (2020) [51]
Provision of free parking at health service (suggestion for promotion and referrals)	Wallis (2020) [51]

Telehealth

**Description**	**Reference**
Disadvantages of telehealth in terms of effectiveness (barrier)	
Remote (telephone) delivery is not as good as face-to-face particularly in relation to exercise advice	Egerton (2017) [31]
Inability of a remote service to provide locally relevant information	Egerton (2017) [31]
An exercise program prescribed by a PT over the telephone would not/might not improve a patient's OA (*)	Lawford (2018) [57]
I do not/might not experience that e-Exercise supports patients in doing their exercises at home (*)	Kloek (2020) [61]
Telehealth is not safe for patients or patient/data privacy (barrier)	
Concerns about security of patient data and information confidentiality during the referral process	Egerton (2017) [31]
Using the telephone would not/might not be a safe way for patients to receive a PT-prescribed exercise program for their OA (*)	Lawford (2018) [57]
Challenges for HCPs regarding lack of physical/visual contact (barrier)	
Forced to modify usual habits/rely more on information shared by patients (instead of own physical assessment)	Hinman (2017) [34]
Some discomfort without hands-on assessment (no palpation of patient's knee/hands-on facilitation of exercises)	Hinman (2017) [34]
Assessment of patients could be difficult when consulting via telephone (inability to observe)	Lawford (2019) [37]
Lack of visual/physical contact would limit strategies available when teaching patients an exercise program	Lawford (2019) [37]
I would not/might not be able to adequately monitor a patient's OA over the telephone (*)	Lawford (2018) [57]
I do not/might not like that there would be no physical contact with an OA patient when consulting over the telephone (*)	Lawford (2018) [57]
I do not/might not like that there would be no physical contact with an OA patient when consulting over the internet video (*)	Lawford (2018) [57]
Reduced face-to-face contact interfered with professional autonomy	Kloek (2020) [61]

For peer review only

Other challenges for HCPs regarding feasibility of telehealth (barrier)	
Lack of financial incentive if blended intervention substitutes conventional visits (reduced venues per patient)	Bossen (2016) [27]
Hesitancy to embrace an unfamiliar new service	Egerton (2017) [31]
Patient flexibility could come at a cost to the therapist sometimes (allowed patients to reschedule last minute)	Hinman (2017) [34]
Skype consultations more suitable as adjunctive to usual in-clinic care (initial assessment in person preferred)	Hinman (2017) [34]
Telephone not viewed as primary mode of providing care (only for follow-up)	Lawford (2019) [37]
Some difficulty scheduling telephone consultations during usual day of face-to-face consultations	Lawford (2019) [37]
Using the telephone to consult with an OA patient and prescribe an exercise program would not/might not be easy for me (*)	Lawford (2018) [57]
I would not/might not be as satisfied talking to an OA patient over the telephone as I would be talking to the patient in person in my consulting room (*)	Lawford (2018) [57]
I would not/might not be interested in being involved in a service offering PT-prescribed exercise over the telephone for my people with OA (*)	Lawford (2018) [57]
Using the telephone would not/might not be an acceptable way for me to deliver an exercise program to patients with OA (*)	Lawford (2018) [57]
Using the telephone would not/might not be a useful (practical) way for me to deliver an exercise program to patients with OA (*)	Lawford (2018) [57]
Using the telephone would not/might not be an effective way for me to deliver an exercise program to patients with OA (*)	Lawford (2018) [57]
Technical skills (lack of)	Kloek (2020) [61]
Adaptive capacity to change treatment routines (lack of)	Kloek (2020) [61]
Absence of national e-Health guideline or standard	Kloek (2020) [61]
Loss of income due to substitution of face-to-face session	Kloek (2020) [61]
Patient-related challenges regarding feasibility of telehealth (barrier)	
Most of the patients prefer traditional face-to-face treatments	Bossen (2016) [27]
Hearing and cognitive difficulties as barriers for some patients to being able to interact with the service	Egerton (2017) [31]
Skepticism about whether many patients would embrace such a model (i.e. because of remote-delivery aspect)	Egerton (2017) [31]
Lack of technology affinity (reason for patients' non-willingness to participate in e-Exercise)	Kloek (2020) [61]
Patients preferred regular face-to-face contact	Kloek (2020) [61]
Negative aspects regarding communication and relationship using telehealth (barrier)	
Lack of face-to-face contact difficult/hampered ability to establish normal rapport/build effective relationships	Hinman (2016) [33]
Occasional technical difficulties (e.g. poor internet connection) could disrupt the flow of the consultation	Hinman (2017) [34]
Relationships with patients might be adversely impacted/could be difficult to develop rapport	Lawford (2019) [37]
Difficulties communicating might be experienced when consulting via telephone	Lawford (2019) [37]
Video consultations made it more difficult to have emotional conversations/read non-verbal cues	Lawford (2021) [39]
Benefits of telehealth in terms of effectiveness (facilitator)	
Possibility to extend physical therapy treatment in patient's home environment	Bossen (2016) [27]
Potential to enhance the adherence of home exercises	Bossen (2016) [27]
Empowering effect of home environment on patient adherence with exercise program	Hinman (2017) [34]
Using Skype distilled focus to most important and effective treatment elements to facilitate self-management	Hinman (2017) [34]
Patients more relaxed in home environment/more receptive to the information the therapists provided	Hinman (2017) [34]
Patients could be more comfortable talking about condition/engaging in exercise program from own home	Lawford (2019) [37]

For peer review only

Telephone-delivered care could provide increased opportunities to educate patients about OA	Lawford (2019) [37]
Patient adherence to telephone-delivered exercise program was high	Lawford (2019) [37]
An exercise program prescribed by a PT over the internet video would improve a patient's OA (*)	Lawford (2018) [57]
Added value in terms of exercise adherence (important factor to use web-based application)	Kloek (2020) [61]
Telehealth is safe for patients or patient/data privacy (facilitator)	
Home environment facilitated correct and safe exercise techniques	Hinman (2017) [34]
A patient's privacy would not be violated if I prescribed them an exercise program over the telephone (*)	Lawford (2018) [57]
A patient's privacy would not be violated if I prescribed them an exercise program over the internet video (*)	Lawford (2018) [57]
Using the internet video would be a safe way for patients to receive a PT-prescribed exercise program for their OA (*)	Lawford (2018) [57]
I believe that patient data gathered at the e-Exercise web-application is stored safely (*)	Kloek (2020) [61]
Lack of physical/visual contact not a major issue for HCPs (facilitator)	
Patients responded favorably to the exercises prescribed despite lack of hands-on assessment	Hinman (2017) [34]
Hands-off approach was physically less demanding compared to usual care/contributed to sense of satisfaction	Hinman (2017) [34]
Functional improvements were observable using Skype	Hinman (2017) [34]
Lack of physical and visual contact less of an issue than anticipated	Lawford (2019) [37]
Able to work around the lack of visual contact (erring on the side of caution)	Lawford (2019) [37]
I would get a good understanding of a patient's OA over the telephone (*)	Lawford (2018) [57]
I would get a good understanding of a patient's OA over the internet video (*)	Lawford (2018) [57]
I would be able to adequately monitor a patient's OA over the internet video (*)	Lawford (2018) [57]
Positive attitude or needs of HCPs regarding feasibility of telehealth (facilitator)	
Ease of using Skype for consultations	Hinman (2017) [34]
Quality of technology suitable for providing instructions/prescribing exercises/receiving instantaneous feedback	Hinman (2017) [34]
More effective communication skills would be needed to consult via telephone	Lawford (2019) [37]
It would be necessary to provide patients with pictures or videos of each exercise when consulting via telephone	Lawford (2019) [37]
Experiences providing telephone-delivered care exceeded expectations, resulting in new enthusiasm	Lawford (2019) [37]
Written materials provided to patients helped to prescribe exercises effectively	Lawford (2019) [37]
Training in communication and/or health coaching important to effectively deliver care over telephone	Lawford (2019) [37]
Video consultations were easy and convenient	Lawford (2021) [39]
Using the internet video to consult with an OA patient and prescribe an exercise program would be easy for me (*)	Lawford (2018) [57]
I would be as satisfied talking to an OA patient over the internet video as I would be talking to the patient in person in my consulting room (*)	Lawford (2018) [57]
I would be interested in being involved in a service offering PT-prescribed exercise over the internet video for my people with OA (*)	Lawford (2018) [57]
Using the internet video would be an acceptable way for me to deliver an exercise program to patients with OA (*)	Lawford (2018) [57]
Using the internet video would be a useful (practical) way for me to deliver an exercise program to patients with OA (*)	Lawford (2018) [57]
Using the internet video would be an effective way for me to deliver an exercise program to patients with OA (*)	Lawford (2018) [57]
Advantage of reducing number of treatments	Kloek (2020) [61]
Offering an innovative intervention attracted new patients	Kloek (2020) [61]
That it results in less income is not/might not be a major disadvantage of e-Exercise (*)	Kloek (2020) [61]

For peer review only

Our physiotherapy practice has the intention to use e-Health innovations (*)	Kloek (2020) [61]
Patient-related benefits regarding feasibility of telehealth (facilitator)	
24/7 availability of information and exercises	Bossen (2016) [27]
Service could increase access to support for rural patients	Egerton (2017) [31]
Skype-delivered care convenient for patients (time efficiency/flexibility/access)	Hinman (2017) [34]
Telephone-delivered care would be convenient for patients	Lawford (2019) [37]
Telephone-delivered care could reduce patient costs associated with accessing physiotherapy services	Lawford (2019) [37]
Telephone-delivered care could allow wider variety of patients to access physiotherapy	Lawford (2019) [37]
Noticeable shift in patients' expectations of physiotherapy care (more willing to self-manage their condition)	Lawford (2019) [37]
Telephone-delivered care was convenient for patients	Lawford (2019) [37]
An exercise program prescribed by a PT over the telephone would save a patient money (*)	Lawford (2018) [57]
An exercise program prescribed by a PT over the internet video would save a patient money (*)	Lawford (2018) [57]
Receiving an exercise program from a PT over the telephone would be a convenient form of health care for an OA patient (*)	Lawford (2018) [57]
Receiving an exercise program from a PT over the internet video would be a convenient form of health care for an OA patient (*)	Lawford (2018) [57]
Receiving an exercise program from a PT over the telephone would save the patient time (*)	Lawford (2018) [57]
Receiving an exercise program from a PT over the internet video would save the patient time (*)	Lawford (2018) [57]
Using the telephone would be an affordable way for patients to receive a PT-prescribed exercise program for their OA (*)	Lawford (2018) [57]
Using the internet video would be an affordable way for patients to receive a PT-prescribed exercise program for their OA (*)	Lawford (2018) [57]
Positive aspects regarding communication and relationship using telehealth (facilitator)	
Developed a strong rapport with patients over the telephone	Lawford (2019) [37]
Consulting via telephone forced to focus on effective conversations with patients (more personal level)	Lawford (2019) [37]
Pleasantly surprised by experience with video consultations (had some of the best conversations)	Lawford (2021) [39]

Domain 2: Individual HCP factors

Expertise

Description	Reference
Lack of knowledge or skills around LIs or promoting behavioral change (barrier)	
Lack of knowledge around appropriate interventions for weight loss	Allison (2019) [26]
Uncertainty about how to enact their understanding of relationship between weight and knee OA	Allison (2019) [26]
Limited knowledge of exercise prescription (uncertainty of what exercise to recommend/how much)	Christiansen (2020) [28]
Not received sufficient training on exercise/lack of education	Christiansen (2020) [28]
Knowledge of exercise and weight-loss treatments is sometimes inaccurate or inadequate	Egerton (2018) [32]
Reduced confidence with providing suitable exercise and weight loss advice	Egerton (2018) [32]
Lack of skills in promoting readiness and motivation for lifestyle treatments	Egerton (2018) [32]
Variability in confidence to provide weight management (not confident)	MacKay (2018) [40]

For peer review only

Not confident in knowledge about weight management	MacKay (2020) [41]
Concerns about capacity to learn/not having skills to fulfill study expectations/deal with challenging patients	Nielsen (2014) [44]
Not have sufficient skills to present PCST component (cognitive restructuring techniques) effectively	Nielsen (2014) [44]
Some process skills were dissimilar to pre-existing clinical communication skills and challenging to use	Nielsen (2014) [44]
Lack of knowledge about CBT (necessary to participate in training/RCT to fully appreciate value of CBT to practice)	Nielsen (2014) [44]
Unclear on amount and type of activity necessary to obtain benefits without further damaging the knee	Poitras (2010) [46]
Uncertainties about dosage/frequency/type of physical activity	Selten (2017) [48]
Unaware about practice guidelines in relation to aerobic exercise prescription/weight loss/pain management	Tang (2020) [49]
Knowledge about BMI/weight management was particularly poor (e.g. relying on visual estimations)	Tang (2020) [49]
Limited knowledge of how to address weight management	Tang (2020) [49]
Less awareness about aerobic exercise prescription	Tang (2020) [49]
Reduced confidence with recommending individual weight/pain management plans (discuss in general terms)	Tang (2020) [49]
Lack of confidence/knowledge/skills in implementing evidence into practice (e.g. weight management)	Teo (2020) [50]
I don't have/might not have the required knowledge and training around obesity care (*)	Hill (2018) [55]
I do not/might not have sufficient knowledge about knee OA exercise therapy and comorbidity to apply the protocol in daily clinical practice (*)	De Rooij (2020) [59]
Gaps in knowledge/skills (including how to facilitate behavior change, particularly with less motivated patients)	Holden (2009) [60]
Lack of knowledge or skills around OA care in general (barrier)	
General lack of expertise/interest in OA (that could lead to inappropriate referral/suboptimal access to services)	Mann (2011) [42]
Lack of physician education on OA care	Miller (2020) [43]
Only two GPs had clear understanding of clinical guidelines on OA	Okwera (2019) [45]
No knowledge about treatment	Rosemann (2006) [47]
Lack of knowledge or skills around specific resources (barrier)	
The issue is not a lack of suitable patient resources but awareness of them	Egerton (2018) [32]
Inability to discuss specific details of ACSM guideline	Tang (2020) [49]
I do not/might not have sufficient skills to apply the protocol in daily clinical practice (*)	De Rooij (2020) [59]
I do not/might not read the protocol sufficiently to remember any of its contents (*)	De Rooij (2020) [59]
Having or improving knowledge or skills around LIs or promoting behavioral change (facilitator)	
Importance of having highly effective communication skills	Egerton (2018) [32]
Value of monitoring/encouraging patients to develop own understanding of links between exercise/pain	Hinman (2016) [33]
Confident in capabilities/skills to use strategies they believed to be effective within scope of practice	MacKay (2018) [40]
Variability in confidence to provide weight management (confident)	MacKay (2018) [40]
Clinical experience helped to read the person's situation (identify approach to motivate them)	MacKay (2020) [41]
Postgraduate continuing professional development courses to expand toolkit of therapeutic interventions	MacKay (2020) [41]
Confidence in addressing weight management	MacKay (2020) [41]
Opportunity to review PCST skills and learn more structured/deliberate ways of incorporating these into practice	Nielsen (2014) [44]
Increasing confidence in using PCST skills over the course of the study	Nielsen (2014) [44]
Improved interpersonal skills with general clinical patients as a result of participating in the study	Nielsen (2014) [44]

Reasonable understanding of role physiotherapy plays in management of lower-limb OA	Okwera (2019) [45]
Knowledge/confidence in providing treatments related to strengthening and range of motion	Tang (2020) [49]
Confident in providing justifications for non-routinely adhering to guidelines (range of motion exercises)	Tang (2020) [49]
Being able to describe how they will manage pain during strengthening exercise	Tang (2020) [49]
Having or improving knowledge or skills around OA care in general (facilitator)	
Need for tailored GP education to improve confidence	Egerton (2018) [32]
Importance of provider knowledge regarding OA management	Miller (2020) [43]
Physician education on OA management can affect both provider and patient attitudes	Miller (2020) [43]
Training sessions (as suggestion for physiotherapy service improvement)	Okwera (2019) [45]
Knowledge about treatment	Rosemann (2006) [47]
Available resources might improve knowledge and decision-making (facilitator)	
Protocols offered guidance in setting up treatment/making clinical decisions/adapting treatment to comorbidity	De Rooij (2014) [30]
List of restrictions was helpful in process of clinical decision making	De Rooij (2014) [30]
Being aware about ACSM guidelines	Tang (2020) [49]
The protocol supports me in clinical reasoning (*)	De Rooij (2020) [59]
The protocol is supporting the improvement of my knowledge regarding knee OA exercise therapy and comorbidity (*)	De Rooij (2020) [59]
The protocol is supportive in which comorbidity-related symptoms I need to monitor before, during and after treatment (*)	De Rooij (2020) [59]
More insight into exercise tolerance/more background knowledge to make clinical decision by using strategy	De Rooij (2020) [59]
Clinical experience (unclear factor)	
Driven by their professional experience of what does and doesn't work/trial and error	MacKay (2020) [41]
Treatments were based more on what works clinically (opposed to scientific evidence)	MacKay (2020) [41]

Attitude

**Description**	**Reference**
Negative attitude toward LIs (barrier)	
Assigning low priority to exercise as treatment	Christiansen (2020) [28]
GP does not prioritise exercise (*)	Cottrell (2016) [52]
It is not/might not be important that people with knee OA increase their overall activity levels (*)	Holden (2009) [60]
Negative attitude toward guidelines or protocols (barrier)	
Frustrations about lack of autonomy with decision-making	Okwera (2019) [45]
Negativity toward guidelines (clinical reasoning more important)	Okwera (2019) [45]
Positive attitude toward LIs (facilitator)	
Acknowledging that weight loss (when someone is overweight) is important	Egerton (2018) [32]
Identifying weight management as important	MacKay (2018) [40]
Perception that exercise and physical activity were central to management	MacKay (2020) [41]
Acknowledging that weight management was a component of management	MacKay (2020) [41]

For peer review only - http://bmjopen.bmj.com/site/about/guidelines.xhtml

Expecting to utilize/continue integrating PCST in general clinical work as physical therapist (beyond the study)	Nielsen (2014) [44]
There is place for each (self-management programs/physiotherapy/orthopedic consultants) in OA management	Okwera (2019) [45]
Many interventions should be used before resorting to medication (including physiotherapy)	Poitras (2010) [46]
Other interventions (including physiotherapy) should be used before paracetamol	Poitras (2010) [46]
NSAIDs alone are not sufficient to appropriately treat inflammation and have to be combined with physiotherapy	Poitras (2010) [46]
Patients should be encouraged to resume/maintain daily activities	Poitras (2010) [46]
Knowing the importance of weight management for knee OA	Tang (2020) [49]
Aware that being overweight/obese is risk factor for knee OA/losing weight is important	Teo (2020) [50]
Physiotherapy (referral) needs to be prioritised	Cottrell (2016) [52]
It is important that people with CKP increase their overall activity levels (*)	Cottrell (2016) [52]
Exercise for CKP should (perhaps) preferably be used before drug treatment has been tried (*)	Cottrell (2016) [52]
Weight loss should be the first-line treatment in the management of obese patients with symptomatic knee OA (*)	Hill (2018) [54]
Obese patients with symptomatic knee OA should be referred to a specialist weight management service before orthopaedic assessment (*)	Hill (2018) [54]
Weight loss should be the first-line treatment in the management of symptomatic knee OA in obesity (*)	Hill (2018) [55]
Support for creation of regional centres where orthopaedic surgeons and bariatric surgeons, with their respective teams, could assess obese patients with symptomatic knee pain (*)	Hill (2018) [55]
Important to try non-surgical treatments first (*)	Hofstede (2016) [56]
Important to delay a surgery as long as possible (*)	Hofstede (2016) [56]
Conservative treatment is (perhaps) an important part of OA management (*)	Reid (2014) [58]
Positive attitude toward guidelines or protocols (facilitator)	
Perceived professional responsibility to adhere to evidence-based guideline	Allison (2019) [26]
Important to follow guidelines (*)	Hofstede (2016) [56]
In general, I do not/might not feel resistance towards working according to protocols (*)	De Rooij (2020) [59]
Autonomy (unclear factor)	
Autonomy affects referral considerations	Law (2019) [36]

Role

**Description**	**Reference**
Perception of own role potentially impeding prescription or follow-up of LIs (barrier)	
Referring patients to other health care providers and for other treatments rather than recommending exercise	Christiansen (2020) [28]
Referring patients to those with specialized knowledge rather than treating themselves (outside scope of practice)	Christiansen (2020) [28]
Paternalistic approach to care (low level of engagement in providing exercise and weight management advice)	Egerton (2018) [32]
Lack of confidence/uncertainty related to role in weight management	MacKay (2020) [41]
Perception that discussions related to diet were not part of their scope of practice	MacKay (2020) [41]
Changing own practice style remained as barrier after OA training	Miller (2020) [43]
Most GPs believed their contribution was essentially limited to diagnosis of condition and medication	Poitras (2010) [46]

For peer review only

Not focusing on increasing patients' motivation for behavioural change, but just giving general recommendations	Rosemann (2006) [47]
Mentioning benefits of weight reduction, but not actively coaching or referring patients	Selten (2017) [48]
Not perceiving weight reduction advice as their responsibility	Selten (2017) [48]
Uncertainty over scope of practice/questioning whether weight and pain management fall outside scope	Tang (2020) [49]
Describing own role as prepping patients for knee surgery when they were referred for physiotherapy	Teo (2020) [50]
Advice about how to lose weight was limited to brief general advice	Teo (2020) [50]
Considering weight loss to be outside own scope of practice (role of a dietician)	Teo (2020) [50]
Comfortable suggesting surgery to patients who responded poorly to conservative management	Teo (2020) [50]
GPs should (perhaps) not follow-up patients to monitor extent of continuation of exercises (*)	Cottrell (2016) [52]
It is the patient's own responsibility to continue doing their exercise programme (*)	Cottrell (2016) [52]
Not (or perhaps not) interested in being the orthopedic surgeon in an ortho-bariatric centre (*)	Hill (2018) [54]
Therapist's role seen as assessment/exercise prescription/education (relatively short-term responsibilities)	Holden (2009) [60]
Patient's role to follow prescribed exercise program over long term/get on board with treatment	Holden (2009) [60]
Physical therapists should (perhaps) not prescribe general exercise for every patient with knee OA (*)	Holden (2009) [60]
It is the patient's own responsibility to continue doing their exercise program (*)	Holden (2009) [60]
It is not/might not be the physiotherapist's responsibility to make sure that the patient will continue doing their exercise program (*)	Holden (2009) [60]
Negative consequences for own role when referring patients to LIs (barrier)	
Job satisfaction may be diminished when handing over care of their patients to third party with no involvement	Egerton (2017) [31]
The addition of a care support team may lead them feeling disconnected with their patient's care	Egerton (2017) [31]
Perception of own role potentially stimulating prescription or follow-up of LIs (facilitator)	
Patient-centred approach (high level of engagement in providing exercise and weight management advice)	Egerton (2018) [32]
Viewing themselves as having an important role in supporting clients to participate in management	MacKay (2018) [40]
Routinely including education about weight management	MacKay (2020) [41]
Playing a role in promoting engagement in management	MacKay (2020) [41]
Necessity of physiotherapy to effectively rehabilitate knee OA patients (because of knowledge/availability)	Poitras (2010) [46]
Importance of PT's role in educating patients with regards to NSAIDs/alternatives (including physiotherapy)	Poitras (2010) [46]
Necessity of PT involvement in managing activity (because potentially detrimental if excessive)	Poitras (2010) [46]
Exercise planning is usually PT's role (rather than GP's)	Poitras (2010) [46]
Necessity of PT follow-up sessions to assess and encourage patient adherence	Poitras (2010) [46]
PT's role to individualize patients' activity according to needs and capacity	Poitras (2010) [46]
Although agreeing with active patient participation, it is ultimately PT's role to appropriately manage patients	Poitras (2010) [46]
Desire to be more involved in life style counselling (upgrade of profession)	Rosemann (2006) [47]
Perceiving exercise prescription to be their main role	Teo (2020) [50]
Advising patients against surgery for as long as possible (last option)	Teo (2020) [50]
Implementing several strategies to boost adherence	Teo (2020) [50]
Not their role to advise the patient about knee surgery, opting not to discuss surgery at all	Teo (2020) [50]
Advising patients against knee arthroscopy if specifically asked about this procedure	Teo (2020) [50]

It is part of my job to reassure patients about the safety of exercise for CKP (*)	Cottrell (2016) [52]
GPs should educate patients with CKP about how to change their lifestyle for the better (*)	Cottrell (2016) [52]
GPs should prescribe quadriceps strengthening exercises to every patient with CKP (*)	Cottrell (2016) [52]
GPs should prescribe general exercise (e.g. walking or swimming) for every patient with CKP (*)	Cottrell (2016) [52]
There was a BMI threshold above which they would not perform a TKA at all (*)	Hill (2018) [54]
There was a BMI threshold above which they would not perform a TKA until the patient had attended a weight management program (*)	Hill (2018) [54]
Intention to refer patients to an ortho-bariatric centre if it existed (*)	Hill (2018) [55]
Recognizing potential influence on exercise adherence, sharing responsibility of exercise adherence with patient	Holden (2009) [60]
Physical therapists should prescribe local strengthening exercise for every patient with knee OA (*)	Holden (2009) [60]
Physiotherapists should educate chronic patients with knee OA about how to change their lifestyle for the better (*)	Holden (2009) [60]
Positive consequences for own role when referring patients to LIs (facilitator)	
Some appeal for a lessening of their own responsibility in terms of managing this condition	Egerton (2017) [31]

Domain 3: Patient factors

Health status

**Description**	**Reference**
Severity of disease and symptoms (barrier)	
Level of disease severity (i.e. whether people with very mild or very severe joint disease would benefit)	Egerton (2017) [31]
Patients delay care until they are highly symptomatic (missing opportunities to slow disease progression)	Miller (2020) [43]
Rehabilitation potential depended on length of disability (less potential with late management)	Poitras (2010) [46]
Paracetamol could mask pain/underlying physical problem (reducing opportunity to assess/manage problem)	Poitras (2010) [46]
Difficult to obtain effective analgesia with some patients	Poitras (2010) [46]
Potential to create unrealistic expectations and discouragement in patients that were too disabled	Poitras (2010) [46]
Questioning capacity to perform regular exercise because of severity of disability	Poitras (2010) [46]
Knee pain restricts activities in general (which makes weight loss difficult)	Poitras (2010) [46]
Pain (main barrier resulting in reduced dosage prescription of strengthening exercises)	Tang (2020) [49]
Patient’s ability to exercise (main barrier resulting in reduced dosage prescription of strengthening exercises)	Tang (2020) [49]
Pain (key barrier to prescription of exercise as recommended by CPGs)	Tang (2020) [49]
Pain (barrier to prescription of aerobic exercise)	Tang (2020) [49]
Osteoarthritis severity (mild/severe) (patient-related barrier)	Wallis (2020) [51]
Effectiveness related to severity of joint damage/pain level (less effective when more damage/pain)	Holden (2009) [60]
Reluctant to promote exercise in the presence of pain	Holden (2009) [60]
Pain (as patient-centered barrier to adherence)	Holden (2009) [60]
Exercises are not/might not be effective for patients if an X-ray shows severe knee osteoarthritis (*)	Holden (2009) [60]
Negative impact of comorbidities (barrier)	

For peer review only

Difficulty with managing multiple conditions/tendency to prioritize other conditions over OA	Christiansen (2020) [28]
Significant impact of other health problems on patients' ability to commit fully to exercise program	Lawford (2020) [38]
Patient body weight (overweight/obese) (impedes exercise/makes visits to services more difficult)	Miller (2020) [43]
Questioning capacity to perform regular exercise because of general health	Poitras (2010) [46]
Depression as important barrier to motivate patients to physical exercise	Rosemann (2006) [47]
Comorbidities (often more severe pain, hampering ability to exercise or be physically active)	Teo (2020) [50]
Existing comorbidities (patient-related barrier)	Wallis (2020) [51]
Other patient characteristics (barrier)	
Person had been sedentary throughout life	Poitras (2010) [46]
Questioning capacity to perform regular exercise because of age	Poitras (2010) [46]
Patients with knee OA tended to be older/less active/with slower metabolism (which makes weight loss difficult)	Poitras (2010) [46]
Older age (patient-related barrier)	Wallis (2020) [51]
Poor rate of previous (physiotherapy) success (*)	Reid (2014) [58]
Severity of disease and symptoms (facilitator)	
Most patients tolerated a lot more than was expected (amount of exercise)	Lawford (2020) [38]
Rehabilitation potential depended on length of disability (better outcomes with early management)	Poitras (2010) [46]
Effective analgesia necessary for patients to be able to accomplish activities	Poitras (2010) [46]
Exercise is effective for patients if an X-ray shows severe knee OA (*)	Cottrell (2016) [52]
Referring patients to physiotherapy if they had high levels of pain/disability and where radiographic evidence of OA was present (*)	Reid (2014) [58]
Effectiveness related to severity of joint damage/pain level (more effective when less damage/pain)	Holden (2009) [60]
Exercises are effective for patients if an X-ray shows moderate knee osteoarthritis (*)	Holden (2009) [60]
Exercises are effective for patients if an X-ray shows mild knee osteoarthritis (*)	Holden (2009) [60]
Other patient characteristics (facilitator)	
Clients' pre-existing activity level (e.g. active person)	MacKay (2018) [40]
Importance of evaluating a patient's overall functional ability (rather than only knee signs/symptoms)	Teo (2020) [50]
Referring patients to physiotherapy if they were of a younger age (*)	Reid (2014) [58]
Severity of disease and symptoms (unclear factor)	
Treatment decisions depended on people's symptoms/findings of physical assessment	MacKay (2020) [41]
Management strategies depended on how bad the knee is	Okwera (2019) [45]
Exercise does not/might not work just as well for everybody, regardless of the amount of pain they have (*)	Cottrell (2016) [52]
Exercise does not/might not work just as well for everybody, regardless of the amount of pain they have (*)	Holden (2009) [60]
Other patient characteristics (unclear factor)	
Clients' general health	MacKay (2018) [40]

Treatment expectations and preferences

**Description**	**Reference**

For peer review only

Negative attitude toward LIs (barrier)	
Patients' lack of motivation to exercise/patients want passive treatment approach or quick fix	Christiansen (2020) [28]
Patients often have own ideas on management (problematic if primarily passive treatments)	Egerton (2018) [32]
Reluctance from patients to talk about physical activity (physical therapist's role, not the coach's role)	Hinman (2016) [33]
Interdisciplinary consult with dietician could not always take place because patients refused to visit dietician	Knoop (2020) [35]
People that don't particularly like exercise	Lawford (2020) [38]
Disconnect between PTs' recommendations for treatment and clients' expectations or preferences	MacKay (2018) [40]
Prior experiences with physical therapy influenced client expectations of clinical encounter	MacKay (2018) [40]
People's preferences were at odds with physical therapists' beliefs about management	MacKay (2020) [41]
Doubts about patients' willingness to make behavioral changes	Mann (2011) [42]
Patients don't want to expend effort towards lifestyle change	Miller (2020) [43]
Public expectation of what physical therapy treatment should be (e.g. didn't come to have thinking challenged)	Nielsen (2014) [44]
Feeling that patients tended to prefer treatment administered to them	Okwera (2019) [45]
Negative comments about patient reports of a lack of "hands-on" physiotherapy	Okwera (2019) [45]
Benefits obtained in the long term, which often conflicted with patient expectations for short-term benefits	Poitras (2010) [46]
Lack of patient motivation in remaining active despite knee OA	Poitras (2010) [46]
Patient views and expectations rarely matched patient needs	Poitras (2010) [46]
Questioning capacity to perform regular exercise because of motivation	Poitras (2010) [46]
Success rate in motivating patients too low (distinctly resignated regarding their impact on patients' life style)	Rosemann (2006) [47]
Frustration about impact of information (e.g. self-help groups) (lot of patients find excuses not to participate)	Rosemann (2006) [47]
Self-motivation (intrinsic barrier for patient adherence)	Teo (2020) [50]
Patient expectations (not keen to participate in exercise/play active role in management, desire for quick fix)	Teo (2020) [50]
Lack of motivation to participate active lifestyle interventions (patient-related barrier)	Wallis (2020) [51]
Existing relationships with physiotherapists (as barrier to referral if patient already had treating physiotherapist)	Wallis (2020) [51]
Exercise does not match patient needs/expectations (*)	Cottrell (2016) [52]
The patients are not/might not be cooperative in applying the protocol in daily clinical practice (*)	De Rooij (2020) [59]
Patients with knee OA and comorbidity are not always motivated to perform exercises	De Rooij (2020) [59]
Lack of motivation or laziness (as patient-centered barrier to adherence)	Holden (2009) [60]
Negative treatment expectations (as patient-centered barrier to adherence)	Holden (2009) [60]
Positive attitude toward TJA (barrier)	
Patients' ideas about whether they wanted surgery influenced making referrals to the LMP	Law (2019) [36]
Unrealistic expectations of the outcome of joint replacement among patients	Mann (2011) [42]
Feeling pressure by patients to refer them to specialist	Rosemann (2006) [47]
Make use of patients' preference for TJA within LIs (facilitator)	
Suggestion to relist patients completing the programme further up the waiting list (for surgery)	Law (2019) [36]
Using bargaining techniques centering on implications of LMP for replacement surgery (put patient on the list)	Law (2019) [36]
Patients' preferences (unclear factor)	

For peer review only

Management strategies depended on what the person wants	Okwera (2019) [45]

Active participation

Description	Reference
Low patient adherence or engagement (barrier)	
Shifting patients' mind-sets to active participation/making lifestyle changes was challenging/time consuming	Egerton (2018) [32]
How well they felt the individual patient would engage with programme influenced making referrals to the LMP	Law (2019) [36]
Challenging work to get clients to initiate management and maintain it over the long term	MacKay (2018) [40]
Getting buy-in (engaging people in management) often portrayed as challenge	MacKay (2020) [41]
Lack of compliance with home exercise regimes and advice given to patients was common	Okwera (2019) [45]
Patients' adherence to management recommendations was limited (because of fatalism)	Poitras (2010) [46]
Patients' unsatisfactory adherence to exercise programs	Teo (2020) [50]
Main concern was participant adherence to physical activity routines after end of program	Duarte (2019) [53]
Negative perceptions of patients' levels of exercise adherence	Holden (2009) [60]
High patient adherence or engagement (facilitator)	
Positive impact of information, education, and structured monitoring on patients' adherence to exercise	Hinman (2016) [33]
Patients adherent/easy to work with when they engaged in exercise program/started seeing improvements	Lawford (2020) [38]
Cohort of patients, in general, was highly motivated (remained interested/motivated for entirety of 6 months)	Lawford (2021) [39]
Enthusiastic participation of the participants	Duarte (2019) [53]
Importance of high patient adherence or engagement for effectiveness of LIs (facilitator)	
Exercise progression was most effective when the participant requested progression	Davis (2018) [29]
Client participation in management was critical to see improvement in symptoms	MacKay (2018) [40]
Getting buy-in (engaging people in management) critical to improving outcomes	MacKay (2020) [41]
Necessity of patients' active participation in knee OA management (to achieve significant outcomes)	Poitras (2010) [46]
How well a patient complies with their exercise programme determines how effective it will be (*)	Cottrell (2016) [52]
Importance of exercise adherence/link between level of adherence and clinical outcomes (dose-response effect)	Holden (2009) [60]
How well a patient complies with their exercise program determines how effective it will be (*)	Holden (2009) [60]

Capabilities

Description	Reference
Low health literacy (barrier)	
Poor health literacy in chronic disease management negatively influenced discussing exercise/weight management	Egerton (2018) [32]
Diagnosis can foster fear-avoidance (e.g. reduced activity) due to belief activity/exercise will cause further damage	Egerton (2018) [32]
Pessimistic about patients' abilities to make lifestyle changes to address their knee OA (not capable)	Egerton (2018) [32]
Lack of information and patient-professional discussion at point of referral may hinder uptake/retention of LMP	Law (2019) [36]

For peer review only - http://bmjopen.bmj.com/site/about/guidelines.xhtml

Patients were sceptical about safety and benefits of strengthening exercise for OA	Lawford (2020) [38]
Fear (patients required a lot of encouragement and reassurance)	Lawford (2020) [38]
Patients were apprehensive about managing weight by themselves	Lawford (2021) [39]
Some clients had misconceptions about OA (nothing they could do/normalising it as part of ageing)	MacKay (2018) [40]
Some clients feared participation in exercise (concerns for further degeneration)	MacKay (2018) [40]
Accepting diagnosis of OA could be particularly challenging for people with early OA	MacKay (2018) [40]
Clients' language (e.g. haven't mastered English/French)	MacKay (2018) [40]
Clients' lifestyle (e.g. coping, attitude towards pain)	MacKay (2018) [40]
Insufficient information for OA patients (e.g. not providing leaflets)	Mann (2011) [42]
Difficulty convincing patients to consider non-surgical, non-medication treatments	Miller (2020) [43]
Lack of patient self-efficacy (regarding lifestyle changes)	Miller (2020) [43]
Lack of knowledge about OA (patient barrier)	Miller (2020) [43]
Most patients demonstrated fatalism/inadequate knowledge and beliefs related to knee OA management	Poitras (2010) [46]
Ambivalent about patients' ability to lose weight (not able to succeed in making lifestyle changes)	Selten (2017) [48]
The belief that patients are not capable of losing weight	Selten (2017) [48]
Fear of falling (intrinsic barrier for patient adherence)	Teo (2020) [50]
Fear of pain (intrinsic barrier for patient adherence)	Teo (2020) [50]
Language/different cultural backgrounds (patient-related barrier)	Wallis (2020) [51]
Patients do not always believe in/may lack knowledge about effectiveness of exercise therapy	De Rooij (2020) [59]
Fear of harm (as patient-centered barrier to adherence)	Holden (2009) [60]
Limited financial resources (barrier)	
Clients' socioeconomic status (e.g. great poverty, shelter system)	MacKay (2018) [40]
Costs related to weight loss can be prohibitive for patients with limited resources (financial burdens)	Miller (2020) [43]
Other responsibilities (barrier)	
Clients' family responsibilities (e.g. busy, lot going on)	MacKay (2018) [40]
Work/other commitments precluding exercise-therapy (patient-related barrier)	Wallis (2020) [51]
High health literacy or importance of education (facilitator)	
Importance of pain education and reassurance about safety and benefits of exercise	Lawford (2020) [38]
Education contributed to buy-in to treatment (pathology, consequences, treatments)	MacKay (2020) [41]
Need for early education about OA/self-management and treatment options and opportunity to discuss these	Mann (2011) [42]
Ambivalent about patients' ability to lose weight (able)	Selten (2017) [48]
Education focused on self-management strategies	Teo (2020) [50]
Inform patients with knee OA and comorbidity better about benefits of exercise therapy	De Rooij (2020) [59]
Social support (facilitator)	
Level of support patients had from family/people close to them seemed to make a big difference	Lawford (2021) [39]
Integrating patients' social system into treatment	Rosemann (2006) [47]
Health literacy (unclear factor)	

For peer review only - http://bmjopen.bmj.com/site/about/guidelines.xhtml

Management strategies depended on what the person can cope with	Okwera (2019) [45]
Other responsibilities (unclear factor)	
Clients' occupation	MacKay (2018) [40]

Domain 4: Professional interactions

Collaboration

**Description**	**Reference**
Non-optimal interdisciplinary collaboration or healthcare provision (barrier)	
Cautious not to encroach on other HCPs' territory	Allison (2019) [26]
Potential for confusion about the treatment plan	Egerton (2017) [31]
Potential for issues resulting from incongruence of patient advice and information	Egerton (2017) [31]
Second professional not necessary to fulfill health coach role (part of own professional role as physical therapists)	Hinman (2016) [33]
Overlapping roles of physical therapist and coach could be source of conflict if not working from same set of goals	Hinman (2016) [33]
Necessary teamwork less likely if coach/physical therapist did not recognize/support each other's goals	Hinman (2016) [33]
Physicians who did not make timely referrals to physical therapy	MacKay (2018) [40]
Physicians' attitudes could influence clients' perceptions and level of buy-in to physical therapy	MacKay (2018) [40]
Lack of provision for patients who were not candidates for surgery (too long without help)	Mann (2011) [42]
Patients lacked proactive follow-up to support self-management	Mann (2011) [42]
Lack of coordination between leisure, social and health services	Mann (2011) [42]
Insufficient (physiotherapy) intervention when patients were seen	Mann (2011) [42]
Belief that physiotherapists did not find it rewarding/interesting to treat OA patients	Mann (2011) [42]
Criticizing the decision to centralize musculoskeletal physiotherapy service (useful to have somebody in team)	Okwera (2019) [45]
Frustrations about lack of continuity regarding team of physiotherapists within clinic	Okwera (2019) [45]
Not working closely with physiotherapists/frustrations about working relationship	Okwera (2019) [45]
Dissatisfaction about loss of coherent working since centralizing musculoskeletal physiotherapy service	Okwera (2019) [45]
Disagreement on effective exercise parameters	Poitras (2010) [46]
Disagreement on optimal design of exercise programs to increase adherence	Poitras (2010) [46]
Mistrust in interventions dieticians use to help patients' with weight reduction attempts	Selten (2017) [48]
Negative views about physical therapists who provided non-evidence-based treatments	Selten (2017) [48]
Mistrust because they observed huge differences in quality of care delivered by physical therapists	Selten (2017) [48]
Occupational therapists, podiatrists and physical therapists do not work together optimally in OA care	Selten (2017) [48]
Role of rheumatologist in knee/hip OA care perceived as unclear/limited	Selten (2017) [48]
Agreement that orthopedic surgeon's primary task is to assess whether patient is eligible for surgery	Selten (2017) [48]
Orthopedic surgeons were perceived negatively by several healthcare providers	Selten (2017) [48]
Unclear what physio offers (*)	Cottrell (2016) [52]

My colleagues in physiotherapy are not/might not be cooperative in applying the protocol in daily clinical practice (*)	De Rooij (2020) [59]
The general practitioners or other physicians are not/might not be collaborative regarding the application of the protocol in daily clinical practice (*)	De Rooij (2020) [59]
Suboptimal collaboration with general practitioners and physicians	De Rooij (2020) [59]
Referring physicians do not always believe in/may lack knowledge about effectiveness of exercise therapy	De Rooij (2020) [59]
No access to other HCPs (barrier)	
Lack of access to other healthcare providers (e.g. physicians with expertise in OA)	MacKay (2018) [40]
Good interdisciplinary collaboration or healthcare provision, or suggestions for improvement (facilitator)	
Perceived status of physical therapists within health care team and wider community	Allison (2019) [26]
Importance of clearly understanding roles and functions of service, care support team, and themselves	Egerton (2017) [31]
Importance of having confidence in (the skills of) the staff of a new service to deliver on promises	Egerton (2017) [31]
Having a personal relationship with the people providing the service/a desire to work closely with service staff	Egerton (2017) [31]
Appreciation how their participation afforded physical therapists and coaches opportunities to collaborate	Hinman (2016) [33]
Importance of teamwork in delivering the integrated intervention	Hinman (2016) [33]
Reinforcement of health messages from another clinician could be valuable	Hinman (2016) [33]
Having a separate coach freed therapists up to focus on other treatment aspects	Hinman (2016) [33]
Multidisciplinary nature of LMP (ability to utilize expertise from other professionals)	Law (2019) [36]
Importance of good working relationships	MacKay (2018) [40]
Physicians who expressed support for physical therapy/exercise and referred clients to physical therapy early	MacKay (2018) [40]
Patients would be better served by long-term condition model of care (e.g. diabetes mellitus)	Mann (2011) [42]
Patients should initiate own follow-up when needed (as better use of time/health care resources)	Mann (2011) [42]
Allow patients, after initial referral, to use direct access system to service (no need for re-referral)	Mann (2011) [42]
Utilising clinic health educator who met with patients for weight loss discussions and followed up by phone	Miller (2020) [43]
Employing a multi-pronged approach to engage patients in weight loss	Miller (2020) [43]
Overall positive experience of physiotherapy service and therapists	Okwera (2019) [45]
GPs believed PT involvement was necessary to motivate the patient and manage the exercise program	Poitras (2010) [46]
Gate keeper role for GPs could reduce patients' pressure to refer to orthopaedics/decrease performed x-rays	Rosemann (2006) [47]
Involvement of practice nurses is imaginable in the area of life style counselling and advice giving	Rosemann (2006) [47]
Dieticians are helpful for patients trying to lose weight	Selten (2017) [48]
Need for physical therapists to provide evidence-based exercises instead of non-evidence-based modalities	Selten (2017) [48]
Non-pharmacological, non-surgical OA care can and should be provided in a primary care setting	Selten (2017) [48]
GPs have coordinating role (diagnose/monitor, refer when necessary, lifestyle education, long-term coach)	Selten (2017) [48]
Physical therapists can guide patients/provide lifestyle advice (more time compared with GPs)	Selten (2017) [48]
Perceiving rheumatologists' role as valuable (giving injections, providing lifestyle/medication advice, refer)	Selten (2017) [48]
Knowledge that program was delivered by well-trained and trusted physiotherapist	Wallis (2020) [51]
Exercise for CKP is more effectively provided by physiotherapists than GPs (*)	Cottrell (2016) [52]
Obese patients with symptomatic knee OA should be assessed by a specialist multidisciplinary service, which should include an orthopaedic surgeon (*)	Hill (2018) [54]

For peer review only

Support for creation of regional centres where orthopedic surgeons and bariatric surgeons, with their respective teams, could assess obese patients with symptomatic knee pain (*)	Hill (2018) [54]
Obese patients with symptomatic knee OA should be referred to a specialist weight management service before orthopaedic assessment (*)	Hill (2018) [55]
Obese patients with symptomatic knee OA should be assessed by a specialist multidisciplinary service (*)	Hill (2018) [55]
Agreements with colleagues about the content of the care trajectory (*)	Hofstede (2016) [56]
Positive attitudes of colleagues about non-surgical treatments (*)	Hofstede (2016) [56]
Agreements/ deliberations with primary care (GP, physical therapist, dietician) (*)	Hofstede (2016) [56]
Physiotherapists do not/might not lack expertise in OA management (*)	Reid (2014) [58]
Working with the protocol invites me to discuss more with experts in the field of the comorbidity (*)	De Rooij (2020) [59]
Inform referrers better about benefits of exercise therapy in patients with knee OA and comorbidity	De Rooij (2020) [59]
Optimize collaboration with orthopaedic surgeons and other health care providers	De Rooij (2020) [59]
Support from colleagues	Kloek (2020) [61]
Access to other HCPs (facilitator)	
Potential benefit of increased access to OA specialists	Egerton (2017) [31]
Access to a team on-site/a network of healthcare providers they trusted	MacKay (2018) [40]
Those with access to other clinicians recommended to consult another clinician for advice on diet as needed	MacKay (2020) [41]
Care could be improved if every GP practice contained an individual who took a particular interest in OA	Mann (2011) [42]
There should be OA specialist clinicians (all relevant allied health professions) providing services in community	Mann (2011) [42]
In-house physiotherapy (as suggestion for physiotherapy service improvement)	Okwera (2019) [45]

Communication and referral

Description	Reference
Lack of communication between HCPs (barrier)	
Frustrations about lack of contact/communication involved in the referral and discharge process	Okwera (2019) [45]
Dissatisfaction about loss of communication since centralizing musculoskeletal physiotherapy service	Okwera (2019) [45]
Specialist did not take time to explain what they had examined/x-rays he had taken	Rosemann (2006) [47]
Physicians are not always collaborating in discussing medical conditions of patients	De Rooij (2020) [59]
Challenges of communication and referral procedures (barrier)	
Necessary teamwork less likely if communication processes not clearly prescribed/structure not used	Hinman (2016) [33]
Different views were expressed about the preferred medium of communication	Hinman (2016) [33]
Interdisciplinary consult with dietician could not always take place because of problems with contacting dietician	Knoop (2020) [35]
Challenges when coordinating multimodal care (including difficulties with the referral system)	Miller (2020) [43]
Frustrations about restrictive referral pathways	Okwera (2019) [45]
Referral process was convoluted and at times irrelevant	Okwera (2019) [45]
Requesting medical information about patients from specialists takes a lot of time	De Rooij (2020) [59]
Improving communication between HCPs (facilitator)	

For peer review only

GPs wanted to be updated on the advice given and plan made so they know what has been said to their patient	Egerton (2017) [31]
Communication needed to be collaborative, patient-centered and consistent for integrated care to be effective	Hinman (2016) [33]
Need for improving communication (quality of referrals, information at discharge)	Okwera (2019) [45]
Better communication with specialists could increase efficacy of treatment	Rosemann (2006) [47]
Receiving communication back from program physiotherapist about patient outcomes	Wallis (2020) [51]
Clarity on what the patient has done at the physical therapist (*)	Hofstede (2016) [56]
Needs regarding communication and referral procedures (facilitator)	
Need to ensure referral procedures are streamlined in order to minimize impact on their busy schedules	Egerton (2017) [31]
Need for effective, useful and timely channels of communication between the GP and the care support team	Egerton (2017) [31]
Importance of effective mechanisms to communicate	MacKay (2018) [40]
Streamlining the physiotherapy referral process (as suggestion for physiotherapy service improvement)	Okwera (2019) [45]
Straightforward, easy and quick lines of communication among different disciplines in healthcare center	Selten (2017) [48]
Simple, streamlined referral process (suggestion for promotion and referrals)	Wallis (2020) [51]

Domain 5: Incentives and resources

Time

Description	Reference
Lack of time within patient consultations (barrier)	
Time pressure (unable to individualise weight management/develop exercise plans within appointment time)	Egerton (2018) [32]
Restricted in amount of time they could allot per patient	MacKay (2018) [40]
Lack of time to give patients sufficient opportunity to discuss their condition	Mann (2011) [42]
Lifestyle counseling is huge time commitment	Miller (2020) [43]
Appointment times too short to address all of patient's issues and provide lifestyle counseling	Miller (2020) [43]
Time required to teach PCST skills to patients	Nielsen (2014) [44]
Weight reduction advice takes too much time in a consultation	Selten (2017) [48]
Insufficient time in consultations (*)	Cottrell (2016) [52]
Time constraints prevent GPs from providing advice on individual exercises for CKP (*)	Cottrell (2016) [52]
Limited time to review individual patients reduced opportunities to facilitate behavior change	Holden (2009) [60]
Large caseloads and pressure of waiting lists reduced the number of treatment sessions provided	Holden (2009) [60]
Limited opportunity to provide follow-up sessions after discharge	Holden (2009) [60]
Lack of time due to other demands (or not further specified) (barrier)	
The addition of a care support team may increase paperwork	Egerton (2017) [31]
Lack of time to monitor attendance/provide support was compounded by increasing administrative demands	Law (2019) [36]
Wait lists as a burden	MacKay (2018) [40]
Lack of time	Rosemann (2006) [47]

For peer review only

Less satisfied about time needed to get used to e-Exercise during high work pressure/administrative burden	Kloek (2020) [61]
Perceiving web-based application as an additional burden	Kloek (2020) [61]
Busy work schedules and administrative burden hindered testing/using e-Exercise in their practice	Kloek (2020) [61]
Adequate duration of patient consultations (facilitator)	
Importance of longer consultations	Egerton (2018) [32]
Having adequate time to spend with clients	MacKay (2018) [40]
Adequate duration of specific interventions or protocols (facilitator)	
Idea of having some of the burden of managing this patient group (e.g. time) taken away appealing	Egerton (2017) [31]
Incorporating selected PCST components on as-needed basis most practical way within current environment	Nielsen (2014) [44]
Working according to the protocol is not/might not be too time-consuming (*)	De Rooij (2020) [59]
Perceiving web-based application as time-saving	Kloek (2020) [61]
I do not/might not have insufficient time available to get familiar with e-Exercise and to use the web-application (*)	Kloek (2020) [61]

Financial resources

Description	Reference
Limited financial resources within organization (barrier)	
Concern about capacity to recover costs of incorporating CBT into practice	Nielsen (2014) [44]
Treatments (e.g. physiotherapy) prescribed less frequently due to decreasing financial resources	Rosemann (2006) [47]
Financial reward for implementing LIs (facilitator)	
Financial incentivisation	Egerton (2017) [31]
Payment system has to be changed to upgrade conservative treatments and conversation with patient	Rosemann (2006) [47]
Interventions performed by practice nurses have to be reinsured sufficiently	Rosemann (2006) [47]
Working according to the protocol should be financially rewarded (*)	De Rooij (2020) [59]

Information resources

Description	Reference
Lack of information resources (barrier)	
Absence of clear guidelines for weight loss	Allison (2019) [26]
Lack of information about scheme hindered referral	Law (2019) [36]
Lack of resources for face-to-face patient education and patient reference	Miller (2020) [43]
Lack of information about self-help groups/offers on community level	Rosemann (2006) [47]
Missing information about offers e.g. in the community	Rosemann (2006) [47]
Challenges in accessing information resources (barrier)	
Challenges in accessing scientific papers	MacKay (2018) [40]
Difficulty finding high quality, patient-friendly OA educational materials	Miller (2020) [43]

Frustration that material found on Internet or provided by friends/family was frequently inaccurate	Miller (2020) [43]
Cannot access necessary resources (*)	Cottrell (2016) [52]
Availability of information resources (facilitator)	
Clear preference for concrete guidelines or tools for engaging in weight management	Allison (2019) [26]
Recommending informational materials for patients (to mitigate delays in OA care)	Miller (2020) [43]
Standardised flowsheet on OA management (as guide for providers/tool for patient discussions)	Miller (2020) [43]
Specific information about program (suggestion for promotion and referrals)	Wallis (2020) [51]
Clear referral criteria/guideline (*)	Hofstede (2016) [56]
Access to information resources (facilitator)	
Having access to customizable, printable patient resources	Egerton (2018) [32]
Access to current evidence	MacKay (2018) [40]
Professional networks/community of practice as mechanism to facilitate sharing of information	MacKay (2018) [40]
Integrating scientific evidence from studies into their approach to management	MacKay (2020) [41]
Including links on websites of partners (suggestion for promotion and referrals)	Wallis (2020) [51]

Facilities

**Description**	**Reference**
Negative attitude toward information technology (barrier)	
Sceptical about benefit of clinical practice information technology	Egerton (2018) [32]
Potential use of information technology (facilitator)	
Changes to clinical practice information technology (e.g. prompts into clinic software)	Egerton (2018) [32]
Having patient resources embedded within current practice software or routines	Egerton (2018) [32]
Electronic reminders for physicians on how to locate OA treatment information and resources	Miller (2020) [43]
Benefits of working in health centers (facilitator)	
Collaboration among multiple disciplines could be facilitated by working in a health center	Selten (2017) [48]

<u>Domain 6: Capacity for organizational change</u>

Professional paradigm

**Description**	**Reference**
Adequate professional paradigm or suggestions for expansion (facilitator)	
Nature of the physical therapy paradigm (in relation to weight management)	Allison (2019) [26]
Physical therapy scope of practice was adequate to manage clients with perceived early knee OA	MacKay (2018) [40]
Suggestion that it would be useful to expand scope of practice to include ordering diagnostic imaging	MacKay (2018) [40]
Value of increasing profession's explicit understanding/use of PCST skills (practice model may be required)	Nielsen (2014) [44]

For peer review only

Value of incorporating aspects of PCST mind-set into professional training (entry-level vs. postgraduate level)	Nielsen (2014) [44]

Monitoring

**Description**	**Reference**
Audit (facilitator)	
Peer review/audit of professional association (*)	Hofstede (2016) [56]

Support within the organization

**Description**	**Reference**
Management not supportive (barrier)	
The management of my practice is not/might not be collaborative regarding the application of the protocol in daily clinical practice (*)	De Rooij (2020) [59]

Domain 7: Social, political, and legal factors

Healthcare system

**Description**	**Reference**
Restrictions due to health insurance (barrier)	
Lack of funding prevented clients from accessing services/seeking help/getting full course of treatment	MacKay (2018) [40]
Treatments (e.g. physiotherapy) prescribed less frequently due to increasing restrictions by health insurances	Rosemann (2006) [47]
The number of treatments that the patient receives from their insurance company is a barrier in using the protocol (*)	De Rooij (2020) [59]
Number of treatment sessions patients receive from insurance companies restricted application of the strategy	De Rooij (2020) [59]
Benefits of good health insurance (facilitator)	
Patients who are well insured have improved access to services (e.g. physical therapy)	Miller (2020) [43]
Positivity toward private sector (patients will get seen a lot quicker)	Okwera (2019) [45]
Private healthcare supplementation (as suggestion for physiotherapy service improvement)	Okwera (2019) [45]
In complex patients insurance companies should reimburse more treatment sessions	De Rooij (2020) [59]
Government subsidies (facilitator)	
Government-subsidised allied health visits to facilitate utilisation of services that support exercise/weight loss	Egerton (2018) [32]

Domain 8: Patient and HCP interactions

Therapeutic alliance

**Description**	**Reference**

For peer review only

Potential negative influence of implementing LIs to relationship (barrier)	
Feelings of guilt when referring to LMP (dooming patients to a longer wait for surgery)	Law (2019) [36]
Importance of communication and relationship (facilitator)	
Strong therapeutic relationship with patients	Lawford (2020) [38]
Having positive attitude/being encouraging of small changes/being hopeful about OA management	MacKay (2020) [41]
More openly address psychological complaints of patients	Rosemann (2006) [47]
Good communication with patient may help in delaying surgery	Selten (2017) [48]
Importance of having trust	Selten (2017) [48]

Lifestyle as conversation topic

Description	Reference
Challenges of discussing weight (barrier)	
Perceived impact of own weight during weight discussions (not being overweight)	Allison (2019) [26]
Apparent discomfort with having conversations about weight	Allison (2019) [26]
Concern about how weight conversations might threaten patient rapport	Allison (2019) [26]
Weight loss is sensitive topic (afraid of upsetting their patients results in temptation to avoid discussion)	Egerton (2018) [32]
Weight was touchy/sensitive subject to discuss	MacKay (2020) [41]
Difficulties in communicating with patients about being overweight	Selten (2017) [48]
Viewing weight as sensitive subject/feeling uncomfortable discussing it	Tang (2020) [49]
Factors that could ease the way to discussing weight (facilitator)	
Perceived impact of own weight during weight discussions (being overweight)	Allison (2019) [26]
Feeling comfortable discussing role of physical activity in maintaining weight control	MacKay (2020) [41]
Developing rapport with people made it easier to discuss weight management	MacKay (2020) [41]
Reframe discussions around exercise and weight loss (e.g. not blaming/discouraging people)	Miller (2020) [43]
Relationship with patients, developed through numerous sessions, facilitated influence for lifestyle modifications	Poitras (2010) [46]
Having a relationship with patient built on mutual trust/respect would ease way to discussing weight reduction	Selten (2017) [48]

Domain 9: Disease factors

Image

Description	Reference
OA seen as low priority (barrier)	
Assigning low priority to OA as disease	Christiansen (2020) [28]
Concern about providing this service for a condition perceived as low priority	Egerton (2017) [31]
OA was not given enough attention, symptoms were often dismissed/minimized in health care	Mann (2011) [42]

For peer review only

Medical professionals saw OA as low priority with respect to managing their workload	Okwera (2019) [45]
Knee OA more often diagnosed as an unanticipated comorbidity (rarely primary reason for consultation)	Poitras (2010) [46]
Not enough emphasis put on primary prevention of knee OA	Poitras (2010) [46]
Knee OA management seen as unchallenging routine	Poitras (2010) [46]
Belief that nobody is willing to change lifestyle due to OA, disease has to be a lot worse	Rosemann (2006) [47]
OA seen as untreatable and local condition (wear-and-tear) (barrier)	
Describing OA as simply a problem of cartilage degeneration/joint space narrowing (on x-ray)/wear and tear	Egerton (2018) [32]
Belief that symptoms will progress, and that surgery is inevitable	Egerton (2018) [32]
Assumption that patients would have negative connotations associated with the label knee OA	Egerton (2018) [32]
No effective treatment options	Miller (2020) [43]
Incurable nature and negative prognosis of OA	Okwera (2019) [45]
Knee OA seen as uninteresting health problem on which they had limited impact and could not cure	Poitras (2010) [46]
Knee OA was perceived as a degenerative (wear and tear)	Teo (2020) [50]
Using negative language to describe OA (wear-and-tear/joint damage/bone-on-bone/degenerative condition)	Wallis (2020) [51]
Biomedical perspective on knee OA, attributing signs and symptoms to local knee pathology or wear and tear	Holden (2009) [60]
OA seen as chronic degenerative condition that would progressively worsen over time (only cure being surgery)	Holden (2009) [60]
Optimistic views toward OA (facilitator)	
Belief that knee OA is condition that can be successfully managed	Egerton (2018) [32]
Importance of conveying to patients that diagnosis is not all negative/delivering a relatively positive prognosis	Egerton (2018) [32]
Knee OA seen as technically challenging condition	Poitras (2010) [46]

Supplemental File 4. Full overview of all extracted factors per article

Explanation

- In the tables below, all extracted factors per included article are presented. The number in brackets ('[…]') displayed after each article corresponds to the reference number used in the main text of the manuscript.
- Column "Number": the capital letter and color used refer to barriers (B/red), facilitators (F/green) or unclear factors (U/orange).
- Column "Description": (*) at the end of the description indicates that the factor is derived from a close-ended question or attitude statement.
- Column "Subcategory (domain)": the relevant subcategory is displayed first, followed by the number of the domain to which this subcategory belongs. The domain numbers refer to the domains as described in the main text of the manuscript: (1) intervention factors; (2) individual HCP factors; (3) patient factors; (4) professional interactions; (5) incentives and resources; (6) capacity for organizational change; (7) social, political, and legal factors; (8) patient and HCP interactions; (9) disease factors.
- Abbreviations: ACSM: American College of Sports Medicine; BMI: body mass index; CBT: cognitive behavioral therapy; CKP: chronic knee pain; CPG: clinical practice guideline; GP: general practitioner; HCP: healthcare professional; LI: lifestyle intervention; LMP: Lifestyle Management Programme; NSAID: non-steroidal anti-inflammatory drug; NWBE: non-weight bearing quadriceps strengthening exercise; OA: osteoarthritis; PCST: pain coping skills training; PT: physiotherapist; RCT: randomized controlled trial; TKA: total knee arthroplasty; TJA: total joint arthroplasty; WBE: weight bearing functional exercise.

Allison (2019) [26]

Number	Description	Subcategory (domain)
B1	Cautious not to encroach on other HCPs' territory	Non-optimal interdisciplinary collaboration or healthcare provision (4)
B2	Lack of knowledge around appropriate interventions for weight loss	Lack of knowledge or skills around LIs or promoting behavioral change (2)
B3	Uncertainty about how to enact their understanding of relationship between weight and knee OA	Lack of knowledge or skills around LIs or promoting behavioral change (2)
B4	Absence of clear guidelines for weight loss	Lack of information resources (5)
B5	Perceived impact of own weight during weight discussions (not being overweight)	Challenges of discussing weight (8)
B6	Apparent discomfort with having conversations about weight	Challenges of discussing weight (8)
B7	Concern about how weight conversations might threaten patient rapport	Challenges of discussing weight (8)
F1	Nature of the physical therapy paradigm (in relation to weight management)	Adequate professional paradigm or suggestions for expansion (6)
F2	Perceived status of physical therapists within health care team and wider community	Good interdisciplinary collaboration or healthcare provision, or suggestions for improvement (4)
F3	Perceived professional responsibility to adhere to evidence-based guideline	Positive attitude toward guidelines or protocols (2)
F4	Clear preference for concrete guidelines or tools for engaging in weight management	Availability of information resources (5)

For peer review only - http://bmjopen.bmj.com/site/about/guidelines.xhtml

F5	Perceived impact of own weight during weight discussions (being overweight)	Factors that could ease the way to discussing weight (8)

Bossen (2016) [27]

Number	Description	Subcategory (domain)
B1	Lack of financial incentive if blended intervention substitutes conventional visits (reduced venues per patient)	Other challenges for HCPs regarding feasibility of telehealth (1)
B2	Most of the patients prefer traditional face-to-face treatments	Patient-related challenges regarding feasibility of telehealth (1)
B3	Most of the patients did not meet study inclusion criteria	LIs are unavailable or inaccessible (1)
B4	e-Exercise must be adapted for suitable integration into practice (e.g. no insight into modules patients receive)	Challenges for HCPs during delivery of LIs (1)
F1	24/7 availability of information and exercises	Patient-related benefits regarding feasibility of telehealth (1)
F2	Possibility to extend physical therapy treatment in patient's home environment	Benefits of telehealth in terms of effectiveness (1)
F3	Potential to enhance the adherence of home exercises	Benefits of telehealth in terms of effectiveness (1)
F4	Positive feedback regarding the content of e-Exercise	Positive experiences with or suggestions to improve the content or structure of LIs (1)

Christiansen (2020) [28]

Number	Description	Subcategory (domain)
B1	Assigning low priority to OA as disease	OA seen as low priority (9)
B2	Assigning low priority to exercise as treatment	Negative attitude toward LIs (2)
B3	Difficulty with managing multiple conditions/tendency to prioritize other conditions over OA	Negative impact of comorbidities (3)
B4	Not certain that exercise works	LIs have little or no effect on OA (1)
B5	Referring patients to other health care providers and for other treatments rather than recommending exercise	Perception of own role potentially impeding prescription or follow-up of LIs (2)
B6	Limited knowledge of exercise prescription (uncertainty of what exercise to recommend/how much)	Lack of knowledge or skills around LIs or promoting behavioral change (2)
B7	Referring patients to those with specialized knowledge rather than treating themselves (outside scope of practice)	Perception of own role potentially impeding prescription or follow-up of LIs (2)
B8	Not received sufficient training on exercise/lack of education	Lack of knowledge or skills around LIs or promoting behavioral change (2)
B9	Patients' lack of motivation to exercise/patients want passive treatment approach or quick fix	Negative attitude toward LIs (3)

Davis (2018) [29]

Number	Description	Subcategory (domain)

For peer review only

B1	Class required intense supervision, which was difficult to provide when most participants were new	Challenges for HCPs during delivery of LIs (1)
B2	Challenges of supervision when space did not allow clear line of sight	Challenges for HCPs during delivery of LIs (1)
F1	Enthusiastic about the program and described the results (e.g. it was empowering)	LIs have positive mental effects (1)
F2	Initial classes needed to be small with rolling recruitment very beneficial	Ease for HCPs during delivery of LIs (1)
F3	First education session was critical to reducing the participant's anxiety related to exercising	Positive experiences with or suggestions to improve the content or structure of LIs (1)
F4	Importance of empowering the patients rather than 'pushing' them, achieved by 'giving choices'	Positive experiences with or suggestions to improve the content or structure of LIs (1)
F5	Exercise progression was most effective when the participant requested progression	Importance of high patient adherence or engagement for effectiveness of LIs (3)

De Rooij (2014) [30]

Number	Description	Subcategory (domain)
F1	Protocols offered guidance in setting up treatment/making clinical decisions/adapting treatment to comorbidity	Available resources might improve knowledge and decision-making (2)
F2	List of restrictions for exercise therapy was conveniently arranged checklist for diagnostic and treatment phases	Ease for HCPs during delivery of LIs (1)
F3	List of restrictions was helpful in process of clinical decision making	Available resources might improve knowledge and decision-making (2)
F4	Suggestion to increase feasibility by reducing the protocols to three main protocols	Ease for HCPs during delivery of LIs (1)
F5	Less afraid to increase training intensity (preventing adverse events by tailoring programs to individual's capacity)	Research environment or protocols provide a safety net (1)

Egerton (2017) [31]

Number	Description	Subcategory (domain)
B1	Concern that uptake would be negatively impacted if patients were required to pay	Costs of LIs to patients (1)
B2	Concern for overcomplicated system when service is not compatible/complementary with existing initiatives	LIs are not feasible or sustainable (1)
B3	Not seeing need (already adequate skills/resources to support OA patient self-management and lifestyle change)	LIs are not feasible or sustainable (1)
B4	Concern about providing this service for a condition perceived as low priority	OA seen as low priority (9)
B5	Not seeing need (advice already given at their practice would be unhelpfully repeated)	LIs are not feasible or sustainable (1)
B6	Remote (telephone) delivery is not as good as face-to-face particularly in relation to exercise advice	Disadvantages of telehealth in terms of effectiveness (1)
B7	Advice to exercise and lose weight does not work	LIs have little or no effect on OA (1)

For peer review only

B8	Hesitancy to embrace an unfamiliar new service	Other challenges for HCPs regarding feasibility of telehealth (1)
B9	Concern regarding long-term service sustainability	LIs are not feasible or sustainable (1)
B10	Concerns about security of patient data and information confidentiality during the referral process	Telehealth is not safe for patients or patient/data privacy (1)
B11	Job satisfaction may be diminished when handing over care of their patients to third party with no involvement	Negative consequences for own role when referring patients to LIs (2)
B12	The addition of a care support team may add complexities to management	LIs are not feasible or sustainable (1)
B13	The addition of a care support team may increase paperwork	Lack of time due to other demands (or not further specified) (5)
B14	The addition of a care support team may lead them feeling disconnected with their patient's care	Negative consequences for own role when referring patients to LIs (2)
B15	Potential for confusion about the treatment plan	Non-optimal interdisciplinary collaboration or healthcare provision (4)
B16	Potential for issues resulting from incongruence of patient advice and information	Non-optimal interdisciplinary collaboration or healthcare provision (4)
B17	Concerned the service would not be able to provide individualized management for a very diverse population	Insufficient ability to provide personalized treatment within LIs (1)
B18	Hearing and cognitive difficulties as barriers for some patients to being able to interact with the service	Patient-related challenges regarding feasibility of telehealth (1)
B19	Level of disease severity (i.e. whether people with very mild or very severe joint disease would benefit)	Severity of disease and symptoms (3)
B20	Inability of a remote service to provide locally relevant information	Disadvantages of telehealth in terms of effectiveness (1)
B21	Skepticism about whether many patients would embrace such a model (i.e. because of remote-delivery aspect)	Patient-related challenges regarding feasibility of telehealth (1)
F1	More likely to engage with the care support team if it enabled more affordable/accessible allied health	LIs are available or accessible, or suggestions for improvement (1)
F2	Need to ensure referral procedures are streamlined in order to minimize impact on their busy schedules	Needs regarding communication and referral procedures (4)
F3	Need for effective, useful and timely channels of communication between the GP and the care support team	Needs regarding communication and referral procedures (4)
F4	GPs wanted to be updated on the advice given and plan made so they know what has been said to their patient	Improving communication between HCPs (4)
F5	Need for clarity about how the new service would integrate with existing schemes and payment structures	LIs are feasible or sustainable (1)
F6	Seeing need (advice/recommendations may need to be reinforced/provided over several health care episodes)	LIs are feasible or sustainable (1)
F7	Seeing need (extra time and encouragement for the patient would result in better outcomes)	LIs are feasible or sustainable (1)
F8	Potential benefit of increased access to OA specialists	Access to other HCPs (4)
F9	Importance of clearly understanding roles and functions of service, care support team, and themselves	Good interdisciplinary collaboration or healthcare provision, or suggestions for improvement (4)

For peer review only

F10	Importance of broad acceptance (patients/doctors/health service funders) if new service is to continue long term	LIs are feasible or sustainable (1)
F11	Importance of having confidence in (the skills of) the staff of a new service to deliver on promises	Good interdisciplinary collaboration or healthcare provision, or suggestions for improvement (4)
F12	Having a personal relationship with the people providing the service/a desire to work closely with service staff	Good interdisciplinary collaboration or healthcare provision, or suggestions for improvement (4)
F13	Idea of having some of the burden of managing this patient group (e.g. time) taken away appealing	Adequate duration of specific interventions or protocols (5)
F14	Some appeal for a lessening of their own responsibility in terms of managing this condition	Positive consequences for own role when referring patients to LIs (2)
F15	Service could increase access to support for rural patients	Patient-related benefits regarding feasibility of telehealth (1)
F16	Financial incentivisation	Financial reward for implementing LIs (5)

Egerton (2018) [32]

Number	Description	Subcategory (domain)
B1	Describing OA as simply a problem of cartilage degeneration/joint space narrowing (on x-ray)/wear and tear	OA seen as untreatable and local condition (wear-and-tear) (9)
B2	Belief that symptoms will progress, and that surgery is inevitable	OA seen as untreatable and local condition (wear-and-tear) (9)
B3	Knowledge of exercise and weight-loss treatments is sometimes inaccurate or inadequate	Lack of knowledge or skills around LIs or promoting behavioral change (2)
B4	Dubious about effect of exercise and weight-management advice on reducing symptoms	LIs have little or no effect on OA (1)
B5	Reduced confidence with providing suitable exercise and weight loss advice	Lack of knowledge or skills around LIs or promoting behavioral change (2)
B6	Lack of skills in promoting readiness and motivation for lifestyle treatments	Lack of knowledge or skills around LIs or promoting behavioral change (2)
B7	Time pressure (unable to individualise weight management/develop exercise plans within appointment time)	Lack of time within patient consultations (5)
B8	Concerns regarding financial cost to patients when considering referral to other services	Costs of LIs to patients (1)
B9	Lack of availability of support services (e.g. community-based rehabilitation programs) in remote locations	LIs are unavailable or inaccessible (1)
B10	Long waiting lists for support services (e.g. community-based rehabilitation programs)	LIs are unavailable or inaccessible (1)
B11	Sceptical about benefit of clinical practice information technology	Negative attitude toward information technology (5)
B12	The issue is not a lack of suitable patient resources but awareness of them	Lack of knowledge or skills around specific resources (2)
B13	Poor health literacy in chronic disease management negatively influenced discussing exercise/weight management	Low health literacy (3)
B14	Patients often have own ideas on management (problematic if primarily passive treatments)	Negative attitude toward LIs (3)

For peer review only

B15	Shifting patients' mind-sets to active participation/making lifestyle changes was challenging/time consuming	Low patient adherence or engagement (3)
B16	Paternalistic approach to care (low level of engagement in providing exercise and weight management advice)	Perception of own role potentially impeding prescription or follow-up of LIs (2)
B17	Assumption that patients would have negative connotations associated with the label knee OA	OA seen as untreatable and local condition (wear-and-tear) (9)
B18	Diagnosis can foster fear-avoidance (e.g. reduced activity) due to belief activity/exercise will cause further damage	Low health literacy (3)
B19	Pessimistic about patients' abilities to make lifestyle changes to address their knee OA (not capable)	Low health literacy (3)
B20	Weight loss is sensitive topic (afraid of upsetting their patients results in temptation to avoid discussion)	Challenges of discussing weight (8)
F1	Need for tailored GP education to improve confidence	Having or improving knowledge or skills around OA care in general (2)
F2	Importance of having highly effective communication skills	Having or improving knowledge or skills around LIs or promoting behavioral change (2)
F3	Lifestyle treatments benefited other chronic conditions	LIs have positive effects on general health (1)
F4	Importance of longer consultations	Adequate duration of patient consultations (5)
F5	Government-subsidised allied health visits to facilitate utilisation of services that support exercise/weight loss	Government subsidies (7)
F6	Changes to clinical practice information technology (e.g. prompts into clinic software)	Potential use of information technology (5)
F7	Having access to customizable, printable patient resources	Access to information resources (5)
F8	Having patient resources embedded within current practice software or routines	Potential use of information technology (5)
F9	Patient-centred approach (high level of engagement in providing exercise and weight management advice)	Perception of own role potentially stimulating prescription or follow-up of LIs (2)
F10	Belief that knee OA is condition that can be successfully managed	Optimistic views toward OA (9)
F11	Importance of conveying to patients that diagnosis is not all negative/delivering a relatively positive prognosis	Optimistic views toward OA (9)
F12	Acknowledging that weight loss (when someone is overweight) is important	Positive attitude toward LIs (2)

Hinman (2016) [33]

Number	Description	Subcategory (domain)
B1	Structure/timing of exercise program restricted capacity to modify exercises/provide adequate follow-up	Non-optimal content or structure of LIs (1)
B2	Lack of face-to-face contact difficult/hampered ability to establish normal rapport/build effective relationships	Negative aspects regarding communication and relationship using telehealth (1)
B3	Second professional not necessary to fulfill health coach role (part of own professional	Non-optimal interdisciplinary collaboration or healthcare provision (4)

	role as physical therapists)	
B4	Overlapping roles of physical therapist and coach could be source of conflict if not working from same set of goals	Non-optimal interdisciplinary collaboration or healthcare provision (4)
B5	Reluctance from patients to talk about physical activity (physical therapist's role, not the coach's role)	Negative attitude toward LIs (3)
B6	Necessary teamwork less likely if communication processes not clearly prescribed/structure not used	Challenges of communication and referral procedures (4)
B7	Necessary teamwork less likely if coach/physical therapist did not recognize/support each other's goals	Non-optimal interdisciplinary collaboration or healthcare provision (4)
B8	Different views were expressed about the preferred medium of communication	Challenges of communication and referral procedures (4)
F1	Appreciation how their participation afforded physical therapists and coaches opportunities to collaborate	Good interdisciplinary collaboration or healthcare provision, or suggestions for improvement (4)
F2	Positive impact on patients of personalized attention from coach and from advice/education they provided	LIs have positive effects (not further specified) (1)
F3	Value of monitoring/encouraging patients to develop own understanding of links between exercise/pain	Having or improving knowledge or skills around LIs or promoting behavioral change (2)
F4	Positive impact of information, education, and structured monitoring on patients' adherence to exercise	High patient adherence or engagement (3)
F5	Requirements of treatment protocol freed therapists to notice and reflect on impact of the interventions	Ease for HCPs during delivery of LIs (1)
F6	Positive comments about the exercise regimen	Positive experiences with or suggestions to improve the content or structure of LIs (1)
F7	Structured protocol allowed to experience different OA treatment regimen/observe and learn from impact	Ease for HCPs during delivery of LIs (1)
F8	Structure provided by protocol/structure of exercises (how patients included them into daily routine)	Positive experiences with or suggestions to improve the content or structure of LIs (1)
F9	Importance of teamwork in delivering the integrated intervention	Good interdisciplinary collaboration or healthcare provision, or suggestions for improvement (4)
F10	Reinforcement of health messages from another clinician could be valuable	Good interdisciplinary collaboration or healthcare provision, or suggestions for improvement (4)
F11	Having a separate coach freed therapists up to focus on other treatment aspects	Good interdisciplinary collaboration or healthcare provision, or suggestions for improvement (4)
F12	Communication needed to be collaborative, patient-centered and consistent for integrated care to be effective	Improving communication between HCPs (4)

Hinman (2017) [34]

Number	Description	Subcategory (domain)

For peer review only

B1	Occasional technical difficulties (e.g. poor internet connection) could disrupt the flow of the consultation	Negative aspects regarding communication and relationship using telehealth (1)
B2	Patient flexibility could come at a cost to the therapist sometimes (allowed patients to reschedule last minute)	Other challenges for HCPs regarding feasibility of telehealth (1)
B3	Forced to modify usual habits/rely more on information shared by patients (instead of own physical assessment)	Challenges for HCPs regarding lack of physical/visual contact (1)
B4	Some discomfort without hands-on assessment (no palpation of patient's knee/hands-on facilitation of exercises)	Challenges for HCPs regarding lack of physical/visual contact (1)
B5	Skype consultations more suitable as adjunctive to usual in-clinic care (initial assessment in person preferred)	Other challenges for HCPs regarding feasibility of telehealth (1)
F1	Ease of using Skype for consultations	Positive attitude or needs of HCPs regarding feasibility of telehealth (1)
F2	Quality of technology suitable for providing instructions/prescribing exercises/receiving instantaneous feedback	Positive attitude or needs of HCPs regarding feasibility of telehealth (1)
F3	Skype-delivered care convenient for patients (time efficiency/flexibility/access)	Patient-related benefits regarding feasibility of telehealth (1)
F4	Empowering effect of home environment on patient adherence with exercise program	Benefits of telehealth in terms of effectiveness (1)
F5	Home environment facilitated correct and safe exercise techniques	Telehealth is safe for patients or patient/data privacy (1)
F6	Using Skype distilled focus to most important and effective treatment elements to facilitate self-management	Benefits of telehealth in terms of effectiveness (1)
F7	Patients more relaxed in home environment/more receptive to the information the therapists provided	Benefits of telehealth in terms of effectiveness (1)
F8	Patients responded favorably to the exercises prescribed despite lack of hands-on assessment	Lack of physical/visual contact not a major issue for HCPs (1)
F9	Safety net provided by research environment (e.g. patients were previously screened for comorbidities/red flags)	Research environment or protocols provide a safety net (1)
F10	Hands-off approach was physically less demanding compared to usual care/contributed to sense of satisfaction	Lack of physical/visual contact not a major issue for HCPs (1)
F11	Functional improvements experienced by patients	LIs have positive effects on affected joint(s) (1)
F12	Functional improvements were observable using Skype	Lack of physical/visual contact not a major issue for HCPs (1)
F13	Greater confidence to exercise among patients	LIs have positive mental effects (1)

Knoop (2020) [35]

Number	Description	Subcategory (domain)
B1	Maximum number of four sessions was considered too low in many patients	Non-optimal content or structure of LIs (1)
B2	Behavioral approach in exercise therapy and advice to visit GP were considered unnecessary for most patients	Non-optimal content or structure of LIs (1)
B3	Interdisciplinary consult with dietician could not always take place because of problems with contacting dietician	Challenges of communication and referral procedures (4)

B4	Interdisciplinary consult with dietician could not always take place because patients refused to visit dietician	Negative attitude toward LIs (3)
F1	Model of stratified care easy to apply and having added value for daily practice	LIs are feasible or sustainable (1)
F2	Appreciation of applicability of treatment protocols	LIs are feasible or sustainable (1)

Law (2019) [36]

Number	Description	Subcategory (domain)
B1	Patients' ideas about whether they wanted surgery influenced making referrals to the LMP	Positive attitude toward TJA (3)
B2	How well they felt the individual patient would engage with programme influenced making referrals to the LMP	Low patient adherence or engagement (3)
B3	Lack of information about scheme hindered referral	Lack of information resources (5)
B4	Feelings of guilt when referring to LMP (dooming patients to a longer wait for surgery)	Potential negative influence of implementing LIs to relationship (8)
B5	Lack of information and patient-professional discussion at point of referral may hinder uptake/retention of LMP	Low health literacy (3)
B6	Lack of time to monitor attendance/provide support was compounded by increasing administrative demands	Lack of time due to other demands (or not further specified) (5)
F1	Multidisciplinary nature of LMP (whole-person, intensive and functional approach)	Positive experiences with or suggestions to improve the content or structure of LIs (1)
F2	Multidisciplinary nature of LMP (ability to utilize expertise from other professionals)	Good interdisciplinary collaboration or healthcare provision, or suggestions for improvement (4)
F3	Suggestion to relist patients completing the programme further up the waiting list (for surgery)	Make use of patients' preference for TJA within LIs (3)
F4	LMP would benefit from extension of inclusion criteria (patients with less severe OA and lower BMI)	LIs are available or accessible, or suggestions for improvement (1)
F5	Emphasising health benefits of programme	LIs have positive effects (not further specified) (1)
F6	Reminding patients of opportunity to self-manage	LIs have positive mental effects (1)
F7	Using bargaining techniques centering on implications of LMP for replacement surgery (put patient on the list)	Make use of patients' preference for TJA within LIs (3)
F8	Standardization was viewed as important for monitoring and evaluation purposes	Positive experiences with or suggestions to improve the content or structure of LIs (1)
F9	Flexibility was valuable when tackling local participation challenges	Ability and importance of providing personalized treatment within LIs (1)
F10	Helpful social impact of group-based programme	Positive experiences with or suggestions to improve the content or structure of LIs (1)
F11	Further and ongoing evaluation of the LMP would help to address current challenges	LIs are feasible or sustainable (1)

For peer review only

U1	Autonomy affects referral considerations	Autonomy (2)

Lawford (2019) [37]

Number	Description	Subcategory (domain)
B1	Telephone not viewed as primary mode of providing care (only for follow-up)	Other challenges for HCPs regarding feasibility of telehealth (1)
B2	Assessment of patients could be difficult when consulting via telephone (inability to observe)	Challenges for HCPs regarding lack of physical/visual contact (1)
B3	Relationships with patients might be adversely impacted/could be difficult to develop rapport	Negative aspects regarding communication and relationship using telehealth (1)
B4	Difficulties communicating might be experienced when consulting via telephone	Negative aspects regarding communication and relationship using telehealth (1)
B5	Lack of visual/physical contact would limit strategies available when teaching patients an exercise program	Challenges for HCPs regarding lack of physical/visual contact (1)
B6	Some difficulty scheduling telephone consultations during usual day of face-to-face consultations	Other challenges for HCPs regarding feasibility of telehealth (1)
F1	Telephone-delivered care would be convenient for patients	Patient-related benefits regarding feasibility of telehealth (1)
F2	Patients could be more comfortable talking about condition/engaging in exercise program from own home	Benefits of telehealth in terms of effectiveness (1)
F3	Telephone-delivered care could reduce patient costs associated with accessing physiotherapy services	Patient-related benefits regarding feasibility of telehealth (1)
F4	Telephone-delivered care could provide increased opportunities to educate patients about OA	Benefits of telehealth in terms of effectiveness (1)
F5	Telephone-delivered care could allow wider variety of patients to access physiotherapy	Patient-related benefits regarding feasibility of telehealth (1)
F6	More effective communication skills would be needed to consult via telephone	Positive attitude or needs of HCPs regarding feasibility of telehealth (1)
F7	It would be necessary to provide patients with pictures or videos of each exercise when consulting via telephone	Positive attitude or needs of HCPs regarding feasibility of telehealth (1)
F8	Experiences providing telephone-delivered care exceeded expectations, resulting in new enthusiasm	Positive attitude or needs of HCPs regarding feasibility of telehealth (1)
F9	Lack of physical and visual contact less of an issue than anticipated	Lack of physical/visual contact not a major issue for HCPs (1)
F10	Developed a strong rapport with patients over the telephone	Positive aspects regarding communication and relationship using telehealth (1)
F11	Patient adherence to telephone-delivered exercise program was high	Benefits of telehealth in terms of effectiveness (1)
F12	Consulting via telephone forced to focus on effective conversations with patients (more personal level)	Positive aspects regarding communication and relationship using telehealth (1)
F13	Noticeable shift in patients' expectations of physiotherapy care (more willing to self-manage their condition)	Patient-related benefits regarding feasibility of telehealth (1)
F14	Improvements in patient pain and function	LIs have positive effects on affected joint(s) (1)
F15	Increased confidence to self-manage	LIs have positive mental effects (1)

For peer review only

F16	Telephone-delivered care was convenient for patients	Patient-related benefits regarding feasibility of telehealth (1)
F17	Able to work around the lack of visual contact (erring on the side of caution)	Lack of physical/visual contact not a major issue for HCPs (1)
F18	Written materials provided to patients helped to prescribe exercises effectively	Positive attitude or needs of HCPs regarding feasibility of telehealth (1)
F19	There was a safety net in place with the trial (each patient had been screened)	Research environment or protocols provide a safety net (1)
F20	Training in communication and/or health coaching important to effectively deliver care over telephone	Positive attitude or needs of HCPs regarding feasibility of telehealth (1)

Lawford (2020) [38]

Number	Description	Subcategory (domain)
B1	Patients were sceptical about safety and benefits of strengthening exercise for OA	Low health literacy (3)
B2	Fear (patients required a lot of encouragement and reassurance)	Low health literacy (3)
B3	Being apprehensive about aggravating pain in patients	LIs are unsafe or have negative effects (1)
B4	People that don’t particularly like exercise	Negative attitude toward LIs (3)
B5	Mental effort required for WBE program was challenging for patients	Challenges for patients during participation in LIs (1)
B6	Tending to avoid pushing patients in usual clinical practice	LIs are unsafe or have negative effects (1)
B7	Physical challenge was the complexity of WBE program	Challenges for patients during participation in LIs (1)
B8	Challenges associated with cuff weights used to apply resistance in NWBE program	Challenges for HCPs during delivery of LIs (1)
B9	Straight leg raise challenging in NWBE program	Challenges for patients during participation in LIs (1)
B10	Significant impact of other health problems on patients’ ability to commit fully to exercise program	Negative impact of comorbidities (3)
F1	Experiences in study helped them push patients through more pain than they would have previously	Research environment or protocols provide a safety net (1)
F2	NWBE program was generally easier for patients to follow (mental effort)	Ease for patients during participation in LIs (1)
F3	NWBE program was easier to prescribe (mental effort)	Ease for HCPs during delivery of LIs (1)
F4	Most patients tolerated a lot more than was expected (amount of exercise)	Severity of disease and symptoms (3)
F5	Easier to prescribe and progress NWBE than WBE program (physical complexity)	Ease for HCPs during delivery of LIs (1)
F6	Patients adherent/easy to work with when they engaged in exercise program/started seeing improvements	High patient adherence or engagement (3)
F7	Strong therapeutic relationship with patients	Importance of communication and relationship (8)
F8	Importance of pain education and reassurance about safety and benefits of exercise	High health literacy or importance of education (3)
F9	Tailoring exercise programs to individual patient would overcome some challenges	Ability and importance of providing personalized treatment within LIs (1)

Lawford (2021) [39]

For peer review only

Number	Description	Subcategory (domain)
B1	Video consultations made it more difficult to have emotional conversations/read non-verbal cues	Negative aspects regarding communication and relationship using telehealth (1)
B2	Patients were apprehensive about managing weight by themselves	Low health literacy (3)
B3	Volume of resources could be overwhelming/confusing for some patients	Challenges for patients during participation in LIs (1)
F1	Simplicity and convenience of meal replacements	Ease for patients during participation in LIs (1)
F2	Video consultations were easy and convenient	Positive attitude or needs of HCPs regarding feasibility of telehealth (1)
F3	Pleasantly surprised by experience with video consultations (had some of the best conversations)	Positive aspects regarding communication and relationship using telehealth (1)
F4	Level of support patients had from family/people close to them seemed to make a big difference	Social support (3)
F5	Long-term follow-up consultations would be beneficial	Positive experiences with or suggestions to improve the content or structure of LIs (1)
F6	Cohort of patients, in general, was highly motivated (remained interested/motivated for entirety of 6 months)	High patient adherence or engagement (3)
F7	Rapid weight loss was primary driver of motivation	LIs have positive effects on general health (1)
F8	Extremely positive about educational resources provided	Positive experiences with or suggestions to improve the content or structure of LIs (1)
F9	More information about healthy eating beyond meal replacement phase could be included	Positive experiences with or suggestions to improve the content or structure of LIs (1)
F10	Exercise/physical activity program was an important part of intervention	Positive experiences with or suggestions to improve the content or structure of LIs (1)
F11	Large improvements in knee pain	LIs have positive effects on affected joint(s) (1)
F12	Positive lifestyle changes (patients) (e.g. thinking differently)	LIs have positive mental effects (1)

<u>MacKay (2018) [40]</u>

Number	Description	Subcategory (domain)
B1	Lack of infrastructure or local programmes (particularly in rural settings)	LIs are unavailable or inaccessible (1)
B2	Cost was a factor in whether clients could access facilities/programmes	Costs of LIs to patients (1)
B3	Lack of funding prevented clients from accessing services/seeking help/getting full course of treatment	Restrictions due to health insurance (7)
B4	Clients often had a waiting period before accessing care	LIs are unavailable or inaccessible (1)
B5	Wait lists as a burden	Lack of time due to other demands (or not further specified) (5)
B6	Lack of access to other healthcare providers (e.g. physicians with expertise in OA)	No access to other HCPs (4)
B7	Variability in confidence to provide weight management (not confident)	Lack of knowledge or skills around LIs or promoting behavioral change (2)

For peer review only

B8	Physicians who did not make timely referrals to physical therapy	Non-optimal interdisciplinary collaboration or healthcare provision (4)
B9	Physicians’ attitudes could influence clients’ perceptions and level of buy-in to physical therapy	Non-optimal interdisciplinary collaboration or healthcare provision (4)
B10	Restricted in amount of time they could allot per patient	Lack of time within patient consultations (5)
B11	Challenges in accessing scientific papers	Challenges in accessing information resources (5)
B12	Challenging work to get clients to initiate management and maintain it over the long term	Low patient adherence or engagement (3)
B13	Disconnect between PTs’ recommendations for treatment and clients’ expectations or preferences	Negative attitude toward LIs (3)
B14	Prior experiences with physical therapy influenced client expectations of clinical encounter	Negative attitude toward LIs (3)
B15	Some clients had misconceptions about OA (nothing they could do/normalising it as part of ageing)	Low health literacy (3)
B16	Some clients feared participation in exercise (concerns for further degeneration)	Low health literacy (3)
B17	Accepting diagnosis of OA could be particularly challenging for people with early OA	Low health literacy (3)
B18	Clients’ socioeconomic status (e.g. great poverty, shelter system)	Limited financial resources (3)
B19	Clients’ language (e.g. haven't mastered English/French)	Low health literacy (3)
B20	Clients’ family responsibilities (e.g. busy, lot going on)	Other responsibilities (3)
B21	Clients’ lifestyle (e.g. coping, attitude towards pain)	Low health literacy (3)
F1	Benefits of having infrastructure and programmes available in their communities	LIs are available or accessible, or suggestions for improvement (1)
F2	Access to a team on-site/a network of healthcare providers they trusted	Access to other HCPs (4)
F3	Importance of good working relationships	Good interdisciplinary collaboration or healthcare provision, or suggestions for improvement (4)
F4	Importance of effective mechanisms to communicate	Needs regarding communication and referral procedures (4)
F5	Confident in capabilities/skills to use strategies they believed to be effective within scope of practice	Having or improving knowledge or skills around LIs or promoting behavioral change (2)
F6	Identifying weight management as important	Positive attitude toward LIs (2)
F7	Variability in confidence to provide weight management (confident)	Having or improving knowledge or skills around LIs or promoting behavioral change (2)
F8	Treatment could improve clients’ symptoms (e.g. reduce pain, increase function)	LIs have positive effects on affected joint(s) (1)
F9	Treatment could potentially slow progression of symptoms	LIs have positive effects on affected joint(s) (1)
F10	Physicians who expressed support for physical therapy/exercise and referred clients to physical therapy early	Good interdisciplinary collaboration or healthcare provision, or suggestions for improvement (4)
F11	Physical therapy scope of practice was adequate to manage clients with perceived early knee OA	Adequate professional paradigm or suggestions for expansion (6)
F12	Suggestion that it would be useful to expand scope of practice to include ordering diagnostic imaging	Adequate professional paradigm or suggestions for expansion (6)

For peer review only

F13	Having adequate time to spend with clients	Adequate duration of patient consultations (5)
F14	Access to current evidence	Access to information resources (5)
F15	Professional networks/community of practice as mechanism to facilitate sharing of information	Access to information resources (5)
F16	Client participation in management was critical to see improvement in symptoms	Importance of high patient adherence or engagement for effectiveness of LIs (3)
F17	Viewing themselves as having an important role in supporting clients to participate in management	Perception of own role potentially stimulating prescription or follow-up of LIs (2)
F18	Clients' pre-existing activity level (e.g. active person)	Other patient characteristics (3)
U1	Clients' general health	Other patient characteristics (3)
U2	Clients' occupation	Other responsibilities (3)

MacKay (2020) [41]

Number	Description	Subcategory (domain)
B1	Lack of confidence/uncertainty related to role in weight management	Perception of own role potentially impeding prescription or follow-up of LIs (2)
B2	Weight was touchy/sensitive subject to discuss	Challenges of discussing weight (8)
B3	Not confident in knowledge about weight management	Lack of knowledge or skills around LIs or promoting behavioral change (2)
B4	Perception that discussions related to diet were not part of their scope of practice	Perception of own role potentially impeding prescription or follow-up of LIs (2)
B5	Getting buy-in (engaging people in management) often portrayed as challenge	Low patient adherence or engagement (3)
B6	People's preferences were at odds with physical therapists' beliefs about management	Negative attitude toward LIs (3)
F1	Clinical experience helped to read the person's situation (identify approach to motivate them)	Having or improving knowledge or skills around LIs or promoting behavioral change (2)
F2	Integrating scientific evidence from studies into their approach to management	Access to information resources (5)
F3	Postgraduate continuing professional development courses to expand toolkit of therapeutic interventions	Having or improving knowledge or skills around LIs or promoting behavioral change (2)
F4	Interventions in physical therapists' toolbox were not static (changed over time)	LIs are feasible or sustainable (1)
F5	Perception that exercise and physical activity were central to management	Positive attitude toward LIs (2)
F6	Acknowledging that weight management was a component of management	Positive attitude toward LIs (2)
F7	Confidence in addressing weight management	Having or improving knowledge or skills around LIs or promoting behavioral change (2)
F8	Routinely including education about weight management	Perception of own role potentially stimulating prescription or follow-up of LIs (2)
F9	Feeling comfortable discussing role of physical activity in maintaining weight control	Factors that could ease the way to discussing weight (8)
F10	Those with access to other clinicians recommended to consult another clinician for advice	Access to other HCPs (4)

For peer review only

	on diet as needed	
F11	Developing rapport with people made it easier to discuss weight management	Factors that could ease the way to discussing weight (8)
F12	Getting buy-in (engaging people in management) critical to improving outcomes	Importance of high patient adherence or engagement for effectiveness of LIs (3)
F13	Playing a role in promoting engagement in management	Perception of own role potentially stimulating prescription or follow-up of LIs (2)
F14	Education contributed to buy-in to treatment (pathology, consequences, treatments)	High health literacy or importance of education (3)
F15	Tailoring treatment to a person's goals/interests	Ability and importance of providing personalized treatment within LIs (1)
F16	Need to consider personal context by integrating people's home exercises into daily activities/other life demands	Ability and importance of providing personalized treatment within LIs (1)
F17	Improve people's symptoms early in treatment (to gain buy-in)	LIs have positive effects on affected joint(s) (1)
F18	Having positive attitude/being encouraging of small changes/being hopeful about OA management	Importance of communication and relationship (8)
U1	Driven by their professional experience of what does and doesn't work/trial and error	Clinical experience (2)
U2	Treatments were based more on what works clinically (opposed to scientific evidence)	Clinical experience (2)
U3	Treatment decisions depended on people's symptoms/findings of physical assessment	Severity of disease and symptoms (3)

Mann (2011) [42]

Number	Description	Subcategory (domain)
B1	Insufficient information for OA patients (e.g. not providing leaflets)	Low health literacy (3)
B2	Doubts about patients' willingness to make behavioral changes	Negative attitude toward LIs (3)
B3	Unrealistic expectations of the outcome of joint replacement among patients	Positive attitude toward TJA (3)
B4	OA was not given enough attention, symptoms were often dismissed/minimized in health care	OA seen as low priority (9)
B5	Lack of provision for patients who were not candidates for surgery (too long without help)	Non-optimal interdisciplinary collaboration or healthcare provision (4)
B6	Patients lacked proactive follow-up to support self-management	Non-optimal interdisciplinary collaboration or healthcare provision (4)
B7	Lack of time to give patients sufficient opportunity to discuss their condition	Lack of time within patient consultations (5)
B8	General lack of expertise/interest in OA (that could lead to inappropriate referral/suboptimal access to services)	Lack of knowledge or skills around OA care in general (2)
B9	Lack of facilities to promote continuing exercise in community	LIs are unavailable or inaccessible (1)
B10	Lack of coordination between leisure, social and health services	Non-optimal interdisciplinary collaboration or healthcare provision (4)
B11	Wait for physiotherapy was too long	LIs are unavailable or inaccessible (1)
B12	Insufficient (physiotherapy) intervention when patients were seen	Non-optimal interdisciplinary collaboration or healthcare provision (4)
B13	Belief that physiotherapists did not find it rewarding/interesting to treat OA patients	Non-optimal interdisciplinary collaboration or healthcare provision (4)

For peer review only

F1	Need for early education about OA/self-management and treatment options and opportunity to discuss these	High health literacy or importance of education (3)
F2	Patients would be better served by long-term condition model of care (e.g. diabetes mellitus)	Good interdisciplinary collaboration or healthcare provision, or suggestions for improvement (4)
F3	Patients should initiate own follow-up when needed (as better use of time/health care resources)	Good interdisciplinary collaboration or healthcare provision, or suggestions for improvement (4)
F4	Allow patients, after initial referral, to use direct access system to service (no need for re-referral)	Good interdisciplinary collaboration or healthcare provision, or suggestions for improvement (4)
F5	Care could be improved if every GP practice contained an individual who took a particular interest in OA	Access to other HCPs (4)
F6	There should be OA specialist clinicians (all relevant allied health professions) providing services in community	Access to other HCPs (4)

Miller (2020) [43]

Number	Description	Subcategory (domain)
B1	No effective treatment options	OA seen as untreatable and local condition (wear-and-tear) (9)
B2	Patients don't want to expend effort towards lifestyle change	Negative attitude toward LIs (3)
B3	Lifestyle counseling is huge time commitment	Lack of time within patient consultations (5)
B4	Difficulty convincing patients to consider non-surgical, non-medication treatments	Low health literacy (3)
B5	Lack of physician education on OA care	Lack of knowledge or skills around OA care in general (2)
B6	Patient body weight (overweight/obese) (impedes exercise/makes visits to services more difficult)	Negative impact of comorbidities (3)
B7	Costs related to weight loss can be prohibitive for patients with limited resources (financial burdens)	Limited financial resources (3)
B8	Lack of patient self-efficacy (regarding lifestyle changes)	Low health literacy (3)
B9	Patients delay care until they are highly symptomatic (missing opportunities to slow disease progression)	Severity of disease and symptoms (3)
B10	Lack of knowledge about OA (patient barrier)	Low health literacy (3)
B11	Costs to patients (lack of insurance coverage/high co-pays for specific services/time off work/travel expenses)	Costs of LIs to patients (1)
B12	Inaccessible treatment options within organization	LIs are unavailable or inaccessible (1)
B13	Lack of resources for face-to-face patient education and patient reference	Lack of information resources (5)
B14	Challenges when coordinating multimodal care (including difficulties with the referral system)	Challenges of communication and referral procedures (4)
B15	Appointment times too short to address all of patient's issues and provide lifestyle counseling	Lack of time within patient consultations (5)
B16	Difficulty finding high quality, patient-friendly OA educational materials	Challenges in accessing information resources (5)

For peer review only

B17	Frustration that material found on Internet or provided by friends/family was frequently inaccurate	Challenges in accessing information resources (5)
B18	Surgical methods have the best outcomes	LIs have little or no effect on OA (1)
B19	Changing own practice style remained as barrier after OA training	Perception of own role potentially impeding prescription or follow-up of LIs (2)
F1	Importance of provider knowledge regarding OA management	Having or improving knowledge or skills around OA care in general (2)
F2	Physical therapy helpful for patients most of the time	LIs have positive effects (not further specified) (1)
F3	Utilising clinic health educator who met with patients for weight loss discussions and followed up by phone	Good interdisciplinary collaboration or healthcare provision, or suggestions for improvement (4)
F4	Employing a multi-pronged approach to engage patients in weight loss	Good interdisciplinary collaboration or healthcare provision, or suggestions for improvement (4)
F5	Patients who are well insured have improved access to services (e.g. physical therapy)	Benefits of good health insurance (7)
F6	Physician education on OA management can affect both provider and patient attitudes	Having or improving knowledge or skills around OA care in general (2)
F7	Reframe discussions around exercise and weight loss (e.g. not blaming/discouraging people)	Factors that could ease the way to discussing weight (8)
F8	Recommending informational materials for patients (to mitigate delays in OA care)	Availability of information resources (5)
F9	Standardised flowsheet on OA management (as guide for providers/tool for patient discussions)	Availability of information resources (5)
F10	Electronic reminders for physicians on how to locate OA treatment information and resources	Potential use of information technology (5)

Nielsen (2014) [44]

Number	Description	Subcategory (domain)
B1	Concerns about capacity to learn/not having skills to fulfill study expectations/deal with challenging patients	Lack of knowledge or skills around LIs or promoting behavioral change (2)
B2	Requirements of RCT potentially created a barrier to responding to where the client was	Insufficient ability to provide personalized treatment within LIs (1)
B3	Difficulty for patients with PCST component (cognitive restructuring techniques)	Challenges for patients during participation in LIs (1)
B4	Not have sufficient skills to present PCST component (cognitive restructuring techniques) effectively	Lack of knowledge or skills around LIs or promoting behavioral change (2)
B5	Some process skills were dissimilar to pre-existing clinical communication skills and challenging to use	Lack of knowledge or skills around LIs or promoting behavioral change (2)
B6	Time required to teach PCST skills to patients	Lack of time within patient consultations (5)
B7	Concern about capacity to recover costs of incorporating CBT into practice	Limited financial resources within organization (5)
B8	Lack of knowledge about CBT (necessary to participate in training/RCT to fully appreciate value of CBT to practice)	Lack of knowledge or skills around LIs or promoting behavioral change (2)
B9	Public expectation of what physical therapy treatment should be (e.g. didn't come to have thinking challenged)	Negative attitude toward LIs (3)

For peer review only

F1	Training workshop as good introduction to content and process of delivering PCST program	Ease for HCPs during delivery of LIs (1)
F2	Weekly group interaction crucial to being able to deliver intervention effectively/problem-solve issues	Ease for HCPs during delivery of LIs (1)
F3	Input from supervising psychologist crucial to being able to deliver intervention effectively/problem-solve issues	Ease for HCPs during delivery of LIs (1)
F4	Would have liked more role-playing experience prior to beginning trial treatments	Ease for HCPs during delivery of LIs (1)
F5	Favorably comments on program content (positive way to help people be proactive about their pain)	Positive experiences with or suggestions to improve the content or structure of LIs (1)
F6	Opportunity to review PCST skills and learn more structured/deliberate ways of incorporating these into practice	Having or improving knowledge or skills around LIs or promoting behavioral change (2)
F7	Some modules worked better than others (depending on the individual patient and context)	Ability and importance of providing personalized treatment within LIs (1)
F8	Importance of PCST component (cognitive restructuring techniques)	Positive experiences with or suggestions to improve the content or structure of LIs (1)
F9	Structure of PCST sessions (overview/practice review/covering new skill/practice planning) worked well	Positive experiences with or suggestions to improve the content or structure of LIs (1)
F10	Regular group meetings were considered very important (if not essential) for delivery of PCST program	Ease for HCPs during delivery of LIs (1)
F11	Value of having a psychologist involved throughout the program, their professional input was helpful	Ease for HCPs during delivery of LIs (1)
F12	Expecting to utilize/continue integrating PCST in general clinical work as physical therapist (beyond the study)	Positive attitude toward LIs (2)
F13	The belief that a more flexible approach responsive to patient needs was required in their practice	Ability and importance of providing personalized treatment within LIs (1)
F14	Increasing confidence in using PCST skills over the course of the study	Having or improving knowledge or skills around LIs or promoting behavioral change (2)
F15	Improved interpersonal skills with general clinical patients as a result of participating in the study	Having or improving knowledge or skills around LIs or promoting behavioral change (2)
F16	Value of increasing profession's explicit understanding/use of PCST skills (practice model may be required)	Adequate professional paradigm or suggestions for expansion (6)
F17	Incorporating selected PCST components on as-needed basis most practical way within current environment	Adequate duration of specific interventions or protocols (5)
F18	Value of incorporating aspects of PCST mind-set into professional training (entry-level vs. postgraduate level)	Adequate professional paradigm or suggestions for expansion (6)

Okwera (2019) [45]

For peer review only

Number	Description	Subcategory (domain)
B1	Incurable nature and negative prognosis of OA	OA seen as untreatable and local condition (wear-and-tear) (9)
B2	Medical professionals saw OA as low priority with respect to managing their workload	OA seen as low priority (9)
B3	Frustrations about restrictive referral pathways	Challenges of communication and referral procedures (4)
B4	Frustrations about lack of autonomy with decision-making	Negative attitude toward guidelines or protocols (2)
B5	Only two GPs had clear understanding of clinical guidelines on OA	Lack of knowledge or skills around OA care in general (2)
B6	Negativity toward guidelines (clinical reasoning more important)	Negative attitude toward guidelines or protocols (2)
B7	Feeling that patients tended to prefer treatment administered to them	Negative attitude toward LIs (3)
B8	Lack of compliance with home exercise regimes and advice given to patients was common	Low patient adherence or engagement (3)
B9	Lack of confidence in clinical effectiveness of physiotherapy treatments	LIs have little or no effect on OA (1)
B10	Negative comments about patient reports of a lack of "hands-on" physiotherapy	Negative attitude toward LIs (3)
B11	Criticizing the decision to centralize musculoskeletal physiotherapy service (useful to have somebody in team)	Non-optimal interdisciplinary collaboration or healthcare provision (4)
B12	Frustrations about lack of continuity regarding team of physiotherapists within clinic	Non-optimal interdisciplinary collaboration or healthcare provision (4)
B13	Frustrations about lack of contact/communication involved in the referral and discharge process	Lack of communication between HCPs (4)
B14	Referral process was convoluted and at times irrelevant	Challenges of communication and referral procedures (4)
B15	Not working closely with physiotherapists/frustrations about working relationship	Non-optimal interdisciplinary collaboration or healthcare provision (4)
B16	Dissatisfaction about loss of communication since centralizing musculoskeletal physiotherapy service	Lack of communication between HCPs (4)
B17	Dissatisfaction about loss of coherent working since centralizing musculoskeletal physiotherapy service	Non-optimal interdisciplinary collaboration or healthcare provision (4)
F1	There is place for each (self-management programs/physiotherapy/orthopedic consultants) in OA management	Positive attitude toward LIs (2)
F2	Positivity toward private sector (patients will get seen a lot quicker)	Benefits of good health insurance (7)
F3	Reasonable understanding of role physiotherapy plays in management of lower-limb OA	Having or improving knowledge or skills around LIs or promoting behavioral change (2)
F4	Overall positive experience of physiotherapy service and therapists	Good interdisciplinary collaboration or healthcare provision, or suggestions for improvement (4)
F5	Need for improving communication (quality of referrals, information at discharge)	Improving communication between HCPs (4)
F6	In-house physiotherapy (as suggestion for physiotherapy service improvement)	Access to other HCPs (4)
F7	Streamlining the physiotherapy referral process (as suggestion for physiotherapy service improvement)	Needs regarding communication and referral procedures (4)
F8	Training sessions (as suggestion for physiotherapy service improvement)	Having or improving knowledge or skills around OA care in general (2)
F9	Triage service (as suggestion for physiotherapy service improvement)	LIs are available or accessible, or suggestions for improvement (1)
F10	Private healthcare supplementation (as suggestion for physiotherapy service improvement)	Benefits of good health insurance (7)

F11	A web-based physiotherapy service (as suggestion for physiotherapy service improvement)	LIs are available or accessible, or suggestions for improvement (1)
F12	Reduced waiting times (as suggestion for physiotherapy service improvement)	LIs are available or accessible, or suggestions for improvement (1)
U1	Management strategies depended on what the person wants	Patients' preferences (3)
U2	Management strategies depended on what the person can cope with	Health literacy (3)
U3	Management strategies depended on how bad the knee is	Severity of disease and symptoms (3)

Poitras (2010) [46]

Number	Description	Subcategory (domain)
B1	Knee OA seen as uninteresting health problem on which they had limited impact and could not cure	OA seen as untreatable and local condition (wear-and-tear) (9)
B2	Knee OA more often diagnosed as an unanticipated comorbidity (rarely primary reason for consultation)	OA seen as low priority (9)
B3	Not enough emphasis put on primary prevention of knee OA	OA seen as low priority (9)
B4	Most GPs believed their contribution was essentially limited to diagnosis of condition and medication	Perception of own role potentially impeding prescription or follow-up of LIs (2)
B5	Knee OA management seen as unchallenging routine	OA seen as low priority (9)
B6	Rehabilitation potential depended on length of disability (less potential with late management)	Severity of disease and symptoms (3)
B7	Benefits obtained in the long term, which often conflicted with patient expectations for short-term benefits	Negative attitude toward LIs (3)
B8	Paracetamol could mask pain/underlying physical problem (reducing opportunity to assess/manage problem)	Severity of disease and symptoms (3)
B9	Potential further damage to the knee due to activity	LIs are unsafe or have negative effects (1)
B10	Difficult to obtain effective analgesia with some patients	Severity of disease and symptoms (3)
B11	Lack of patient motivation in remaining active despite knee OA	Negative attitude toward LIs (3)
B12	Person had been sedentary throughout life	Other patient characteristics (3)
B13	Potential to create unrealistic expectations and discouragement in patients that were too disabled	Severity of disease and symptoms (3)
B14	Patient views and expectations rarely matched patient needs	Negative attitude toward LIs (3)
B15	Questioning capacity to perform regular exercise because of severity of disability	Severity of disease and symptoms (3)
B16	Questioning capacity to perform regular exercise because of age	Other patient characteristics (3)
B17	Questioning capacity to perform regular exercise because of general health	Negative impact of comorbidities (3)
B18	Questioning capacity to perform regular exercise because of motivation	Negative attitude toward LIs (3)
B19	Disagreement on effective exercise parameters	Non-optimal interdisciplinary collaboration or healthcare provision (4)
B20	Disagreement on optimal design of exercise programs to increase adherence	Non-optimal interdisciplinary collaboration or healthcare provision (4)

For peer review only

B21	Unclear on amount and type of activity necessary to obtain benefits without further damaging the knee	Lack of knowledge or skills around LIs or promoting behavioral change (2)
B22	Most patients demonstrated fatalism/inadequate knowledge and beliefs related to knee OA management	Low health literacy (3)
B23	Patients' adherence to management recommendations was limited (because of fatalism)	Low patient adherence or engagement (3)
B24	Limited impact of weight loss on established knee OA (more effective as a primary prevention strategy)	LIs have little or no effect on OA (1)
B25	Questioning direct relationship between weight and knee OA (numerous other factors associated)	LIs have little or no effect on OA (1)
B26	Knee pain restricts activities in general (which makes weight loss difficult)	Severity of disease and symptoms (3)
B27	Patients with knee OA tended to be older/less active/with slower metabolism (which makes weight loss difficult)	Other patient characteristics (3)
B28	Weight loss is difficult (multiplicity of factors need to be addressed, often involving change in lifestyle)	Potential effects of LIs are difficult to accomplish (1)
F1	Knee OA seen as technically challenging condition	Optimistic views toward OA (9)
F2	Necessity of physiotherapy to effectively rehabilitate knee OA patients (because of knowledge/availability)	Perception of own role potentially stimulating prescription or follow-up of LIs (2)
F3	Rehabilitation potential depended on length of disability (better outcomes with early management)	Severity of disease and symptoms (3)
F4	Many interventions should be used before resorting to medication (including physiotherapy)	Positive attitude toward LIs (2)
F5	Other interventions (including physiotherapy) should be used before paracetamol	Positive attitude toward LIs (2)
F6	NSAIDs alone are not sufficient to appropriately treat inflammation and have to be combined with physiotherapy	Positive attitude toward LIs (2)
F7	Importance of PT's role in educating patients with regards to NSAIDs/alternatives (including physiotherapy)	Perception of own role potentially stimulating prescription or follow-up of LIs (2)
F8	Benefits of activity on knee mobility	LIs have positive effects on affected joint(s) (1)
F9	Benefits of activity on general wellbeing	LIs have positive effects on general health (1)
F10	Effective analgesia necessary for patients to be able to accomplish activities	Severity of disease and symptoms (3)
F11	Patients should be encouraged to resume/maintain daily activities	Positive attitude toward LIs (2)
F12	Necessity of PT involvement in managing activity (because potentially detrimental if excessive)	Perception of own role potentially stimulating prescription or follow-up of LIs (2)
F13	GPs believed PT involvement was necessary to motivate the patient and manage the exercise program	Good interdisciplinary collaboration or healthcare provision, or suggestions for improvement (4)
F14	Exercise planning is usually PT's role (rather than GP's)	Perception of own role potentially stimulating prescription or follow-up of LIs (2)
F15	Exercise programs have to be individualized to each patient by the PT	Ability and importance of providing personalized treatment within LIs (1)

For peer review only

F16	Necessity of PT follow-up sessions to assess and encourage patient adherence	Perception of own role potentially stimulating prescription or follow-up of LIs (2)
F17	Activity necessary for the knee's health	LIs have positive effects on affected joint(s) (1)
F18	PT's role to individualize patients' activity according to needs and capacity	Perception of own role potentially stimulating prescription or follow-up of LIs (2)
F19	Necessity of patients' active participation in knee OA management (to achieve significant outcomes)	Importance of high patient adherence or engagement for effectiveness of LIs (3)
F20	Although agreeing with active patient participation, it is ultimately PT's role to appropriately manage patients	Perception of own role potentially stimulating prescription or follow-up of LIs (2)
F21	Weight loss effective at improving mobility in general	LIs have positive effects on general health (1)
F22	Weight loss improves pain and joint function	LIs have positive effects on affected joint(s) (1)
F23	Relationship with patients, developed through numerous sessions, facilitated influence for lifestyle modifications	Factors that could ease the way to discussing weight (8)
F24	Weight loss also benefits mobility in general	LIs have positive effects on general health (1)

Rosemann (2006) [47]

Number	Description	Subcategory (domain)
B1	Depression as important barrier to motivate patients to physical exercise	Negative impact of comorbidities (3)
B2	Feeling pressure by patients to refer them to specialist	Positive attitude toward TJA (3)
B3	Specialist did not take time to explain what they had examined/x-rays he had taken	Lack of communication between HCPs (4)
B4	Treatments (e.g. physiotherapy) prescribed less frequently due to decreasing financial resources	Limited financial resources within organization (5)
B5	Treatments (e.g. physiotherapy) prescribed less frequently due to increasing restrictions by health insurances	Restrictions due to health insurance (7)
B6	Not focusing on increasing patients' motivation for behavioural change, but just giving general recommendations	Perception of own role potentially impeding prescription or follow-up of LIs (2)
B7	Success rate in motivating patients too low (distinctly resignated regarding their impact on patients' life style)	Negative attitude toward LIs (3)
B8	Vicious circle (pain when exercising, people move less/eat more due to frustration/sometimes depression)	Potential effects of LIs are difficult to accomplish (1)
B9	Belief that nobody is willing to change lifestyle due to OA, disease has to be a lot worse	OA seen as low priority (9)
B10	Lack of information about self-help groups/offers on community level	Lack of information resources (5)
B11	Frustration about impact of information (e.g. self-help groups) (lot of patients find excuses not to participate)	Negative attitude toward LIs (3)
B12	Missing information about offers e.g. in the community	Lack of information resources (5)
B13	Lack of time	Lack of time due to other demands (or not further specified) (5)

For peer review only

B14	No knowledge about treatment	Lack of knowledge or skills around OA care in general (2)
F1	Gate keeper role for GPs could reduce patients' pressure to refer to orthopaedics/decrease performed x-rays	Good interdisciplinary collaboration or healthcare provision, or suggestions for improvement (4)
F2	Better communication with specialists could increase efficacy of treatment	Improving communication between HCPs (4)
F3	Payment system has to be changed to upgrade conservative treatments and conversation with patient	Financial reward for implementing LIs (5)
F4	Involvement of practice nurses is imaginable in the area of life style counselling and advice giving	Good interdisciplinary collaboration or healthcare provision, or suggestions for improvement (4)
F5	Interventions performed by practice nurses have to be reinsured sufficiently	Financial reward for implementing LIs (5)
F6	Desire to be more involved in life style counselling (upgrade of profession)	Perception of own role potentially stimulating prescription or follow-up of LIs (2)
F7	Knowledge about treatment	Having or improving knowledge or skills around OA care in general (2)
F8	Integrating patients' social system into treatment	Social support (3)
F9	More openly address psychological complaints of patients	Importance of communication and relationship (8)

Selten (2017) [48]

Number	Description	Subcategory (domain)
B1	Ambivalent about patients' ability to lose weight (not able to succeed in making lifestyle changes)	Low health literacy (3)
B2	Mistrust in interventions dieticians use to help patients' with weight reduction attempts	Non-optimal interdisciplinary collaboration or healthcare provision (4)
B3	Mentioning benefits of weight reduction, but not actively coaching or referring patients	Perception of own role potentially impeding prescription or follow-up of LIs (2)
B4	The belief that patients are not capable of losing weight	Low health literacy (3)
B5	Weight reduction advice takes too much time in a consultation	Lack of time within patient consultations (5)
B6	Not perceiving weight reduction advice as their responsibility	Perception of own role potentially impeding prescription or follow-up of LIs (2)
B7	Difficulties in communicating with patients about being overweight	Challenges of discussing weight (8)
B8	Uncertainties about dosage/frequency/type of physical activity	Lack of knowledge or skills around LIs or promoting behavioral change (2)
B9	Less certain about effectiveness of physical therapy (benefits variable or difficult to prove)	LIs have little or no effect on OA (1)
B10	Negative views about physical therapists who provided non-evidence-based treatments	Non-optimal interdisciplinary collaboration or healthcare provision (4)
B11	Mistrust because they observed huge differences in quality of care delivered by physical therapists	Non-optimal interdisciplinary collaboration or healthcare provision (4)
B12	Occupational therapists, podiatrists and physical therapists do not work together optimally in OA care	Non-optimal interdisciplinary collaboration or healthcare provision (4)
B13	Role of rheumatologist in knee/hip OA care perceived as unclear/limited	Non-optimal interdisciplinary collaboration or healthcare provision (4)

For peer review only

B14	Agreement that orthopedic surgeon's primary task is to assess whether patient is eligible for surgery	Non-optimal interdisciplinary collaboration or healthcare provision (4)
B15	Orthopedic surgeons were perceived negatively by several healthcare providers	Non-optimal interdisciplinary collaboration or healthcare provision (4)
F1	Benefits of weight reduction for relieving symptoms of knee/hip OA	LIs have positive effects on affected joint(s) (1)
F2	Ambivalent about patients' ability to lose weight (able)	High health literacy or importance of education (3)
F3	Dieticians are helpful for patients trying to lose weight	Good interdisciplinary collaboration or healthcare provision, or suggestions for improvement (4)
F4	Having a relationship with patient built on mutual trust/respect would ease way to discussing weight reduction	Factors that could ease the way to discussing weight (8)
F5	Value of lifestyle advice related to knee and hip OA	LIs have positive effects (not further specified) (1)
F6	Beneficial effects of physical therapy in reducing pain/stiffness and potential effects on cartilage	LIs have positive effects on affected joint(s) (1)
F7	Beneficial effects of physical therapy in reducing weight and for increasing mobility/posture/coordination	LIs have positive effects on general health (1)
F8	Physical therapy useful in increasing patients self-management in coping with/acceptance of symptoms	LIs have positive mental effects (1)
F9	Need for physical therapists to provide evidence-based exercises instead of non-evidence-based modalities	Good interdisciplinary collaboration or healthcare provision, or suggestions for improvement (4)
F10	Non-pharmacological, non-surgical treatment was considered useful to delay surgery	LIs have positive effects (not further specified) (1)
F11	Good communication with patient may help in delaying surgery	Importance of communication and relationship (8)
F12	Straightforward, easy and quick lines of communication among different disciplines in healthcare center	Needs regarding communication and referral procedures (4)
F13	Collaboration among multiple disciplines could be facilitated by working in a health center	Benefits of working in health centers (5)
F14	Non-pharmacological, non-surgical OA care can and should be provided in a primary care setting	Good interdisciplinary collaboration or healthcare provision, or suggestions for improvement (4)
F15	GPs have coordinating role (diagnose/monitor, refer when necessary, lifestyle education, long-term coach)	Good interdisciplinary collaboration or healthcare provision, or suggestions for improvement (4)
F16	Importance of having trust	Importance of communication and relationship (8)
F17	Physical therapists can guide patients/provide lifestyle advice (more time compared with GPs)	Good interdisciplinary collaboration or healthcare provision, or suggestions for improvement (4)
F18	Perceiving rheumatologists' role as valuable (giving injections, providing lifestyle/medication advice, refer)	Good interdisciplinary collaboration or healthcare provision, or suggestions for improvement (4)

Tang (2020) [49]

Number	Description	Subcategory (domain)
B1	Inability to discuss specific details of ACSM guideline	Lack of knowledge or skills around specific resources (2)

For peer review only

B2	Pain (main barrier resulting in reduced dosage prescription of strengthening exercises)	Severity of disease and symptoms (3)
B3	Patient's ability to exercise (main barrier resulting in reduced dosage prescription of strengthening exercises)	Severity of disease and symptoms (3)
B4	Unaware about practice guidelines in relation to aerobic exercise prescription/weight loss/pain management	Lack of knowledge or skills around LIs or promoting behavioral change (2)
B5	Knowledge about BMI/weight management was particularly poor (e.g. relying on visual estimations)	Lack of knowledge or skills around LIs or promoting behavioral change (2)
B6	Limited knowledge of how to address weight management	Lack of knowledge or skills around LIs or promoting behavioral change (2)
B7	Pain (key barrier to prescription of exercise as recommended by CPGs)	Severity of disease and symptoms (3)
B8	Less awareness about aerobic exercise prescription	Lack of knowledge or skills around LIs or promoting behavioral change (2)
B9	Pain (barrier to prescription of aerobic exercise)	Severity of disease and symptoms (3)
B10	Uncertainty over scope of practice/questioning whether weight and pain management fall outside scope	Perception of own role potentially impeding prescription or follow-up of LIs (2)
B11	Reduced confidence with recommending individual weight/pain management plans (discuss in general terms)	Lack of knowledge or skills around LIs or promoting behavioral change (2)
B12	Viewing weight as sensitive subject/feeling uncomfortable discussing it	Challenges of discussing weight (8)
F1	Knowledge/confidence in providing treatments related to strengthening and range of motion	Having or improving knowledge or skills around LIs or promoting behavioral change (2)
F2	Being aware about ACSM guidelines	Available resources might improve knowledge and decision-making (2)
F3	Confident in providing justifications for non-routinely adhering to guidelines (range of motion exercises)	Having or improving knowledge or skills around LIs or promoting behavioral change (2)
F4	Knowing the importance of weight management for knee OA	Positive attitude toward LIs (2)
F5	Being able to describe how they will manage pain during strengthening exercise	Having or improving knowledge or skills around LIs or promoting behavioral change (2)

Teo (2020) [50]

Number	Description	Subcategory (domain)
B1	Knee OA was perceived as a degenerative (wear and tear)	OA seen as untreatable and local condition (wear-and-tear) (9)
B2	Describing own role as prepping patients for knee surgery when they were referred for physiotherapy	Perception of own role potentially impeding prescription or follow-up of LIs (2)
B3	Comorbidities (often more severe pain, hampering ability to exercise or be physically active)	Negative impact of comorbidities (3)
B4	Patients' unsatisfactory adherence to exercise programs	Low patient adherence or engagement (3)
B5	Self-motivation (intrinsic barrier for patient adherence)	Negative attitude toward LIs (3)

For peer review only

B6	Fear of falling (intrinsic barrier for patient adherence)	Low health literacy (3)
B7	Fear of pain (intrinsic barrier for patient adherence)	Low health literacy (3)
B8	Costs (extrinsic barrier for patient adherence)	Costs of LIs to patients (1)
B9	Weather (extrinsic barrier for patient adherence)	Inconvenience to patients when accessing LIs (1)
B10	Patient expectations (not keen to participate in exercise/play active role in management, desire for quick fix)	Negative attitude toward LIs (3)
B11	Lack of confidence/knowledge/skills in implementing evidence into practice (e.g. weight management)	Lack of knowledge or skills around LIs or promoting behavioral change (2)
B12	Advice about how to lose weight was limited to brief general advice	Perception of own role potentially impeding prescription or follow-up of LIs (2)
B13	Considering weight loss to be outside own scope of practice (role of a dietician)	Perception of own role potentially impeding prescription or follow-up of LIs (2)
B14	Comfortable suggesting surgery to patients who responded poorly to conservative management	Perception of own role potentially impeding prescription or follow-up of LIs (2)
F1	Education focused on self-management strategies	High health literacy or importance of education (3)
F2	Importance of evaluating a patient's overall functional ability (rather than only knee signs/symptoms)	Other patient characteristics (3)
F3	Perceiving exercise prescription to be their main role	Perception of own role potentially stimulating prescription or follow-up of LIs (2)
F4	Importance of tailored exercise program	Ability and importance of providing personalized treatment within LIs (1)
F5	Advising patients against surgery for as long as possible (last option)	Perception of own role potentially stimulating prescription or follow-up of LIs (2)
F6	Implementing several strategies to boost adherence	Perception of own role potentially stimulating prescription or follow-up of LIs (2)
F7	Aware that being overweight/obese is risk factor for knee OA/losing weight is important	Positive attitude toward LIs (2)
F8	Not their role to advise the patient about knee surgery, opting not to discuss surgery at all	Perception of own role potentially stimulating prescription or follow-up of LIs (2)
F9	Advising patients against knee arthroscopy if specifically asked about this procedure	Perception of own role potentially stimulating prescription or follow-up of LIs (2)

Wallis (2020) [51]

Number	Description	Subcategory (domain)
B1	Cost (program access barrier)	Costs of LIs to patients (1)
B2	Transport, waiting time and parking related to attendance (program access barrier)	Inconvenience to patients when accessing LIs (1)
B3	Geography (program access barrier)	Inconvenience to patients when accessing LIs (1)
B4	Available session times (program access barrier)	Inconvenience to patients when accessing LIs (1)

B5	Using negative language to describe OA (wear-and-tear/joint damage/bone-on-bone/degenerative condition)	OA seen as untreatable and local condition (wear-and-tear) (9)
B6	Existing comorbidities (patient-related barrier)	Negative impact of comorbidities (3)
B7	Osteoarthritis severity (mild/severe) (patient-related barrier)	Severity of disease and symptoms (3)
B8	Lack of motivation to participate active lifestyle interventions (patient-related barrier)	Negative attitude toward LIs (3)
B9	Older age (patient-related barrier)	Other patient characteristics (3)
B10	Language/different cultural backgrounds (patient-related barrier)	Low health literacy (3)
B11	Work/other commitments precluding exercise-therapy (patient-related barrier)	Other responsibilities (3)
B12	Program factors (e.g. single discipline led intervention)	Non-optimal content or structure of LIs (1)
B13	Existing relationships with physiotherapists (as barrier to referral if patient already had treating physiotherapist)	Negative attitude toward LIs (3)
B14	Urging caution to patients about participating in higher impact exercise/activities	LIs are unsafe or have negative effects (1)
F1	A more holistic program as part of a multidisciplinary model of service was preferred	Positive experiences with or suggestions to improve the content or structure of LIs (1)
F2	Knowledge that program was delivered by well-trained and trusted physiotherapist	Good interdisciplinary collaboration or healthcare provision, or suggestions for improvement (4)
F3	Receiving communication back from program physiotherapist about patient outcomes	Improving communication between HCPs (4)
F4	Positive about program (alternative approach and opportunity to avoid a joint replacement)	LIs have positive effects (not further specified) (1)
F5	Exercise therapy may be effective by giving more muscular support for joints	LIs have positive effects on affected joint(s) (1)
F6	Exercise therapy may be effective by giving opportunity to improve confidence about activities/mobility	LIs have positive mental effects (1)
F7	Value of program's structure and peer (group) support	Positive experiences with or suggestions to improve the content or structure of LIs (1)
F8	Name of program ('Good Life with OsteoArthritis') implied optimism and positive outcome	Positive experiences with or suggestions to improve the content or structure of LIs (1)
F9	Received positive feedback from their patients about program	Positive experiences with or suggestions to improve the content or structure of LIs (1)
F10	Including links on websites of partners (suggestion for promotion and referrals)	Access to information resources (5)
F11	Simple, streamlined referral process (suggestion for promotion and referrals)	Needs regarding communication and referral procedures (4)
F12	Close, convenient locations (suggestion for promotion and referrals)	Convenience for patients when accessing LIs (1)
F13	Appropriate session times for working populations (suggestion for promotion and referrals)	Convenience for patients when accessing LIs (1)
F14	Specific information about program (suggestion for promotion and referrals)	Availability of information resources (5)
F15	Providing trial of sessions to assist patients to get started (suggestion for promotion and referrals)	Ease for patients during participation in LIs (1)
F16	Provision of free parking at health service (suggestion for promotion and referrals)	Convenience for patients when accessing LIs (1)

For peer review only

Cottrell (2016) [52]

Number	Description	Subcategory (domain)
B1	Insufficient time in consultations (*)	Lack of time within patient consultations (5)
B2	Limitations to accessing services (e.g. lack of facilities, costs) (*)	LIs are unavailable or inaccessible (1)
B3	Services do not meet expectations (*)	Non-optimal content or structure of LIs (1)
B4	Geographical problems (e.g. remote location, scared to walk in local area) (*)	Inconvenience to patients when accessing LIs (1)
B5	Cannot access necessary resources (*)	Challenges in accessing information resources (5)
B6	GP does not prioritise exercise (*)	Negative attitude toward LIs (2)
B7	Unclear what physio offers (*)	Non-optimal interdisciplinary collaboration or healthcare provision (4)
B8	Exercise does not match patient needs/expectations (*)	Negative attitude toward LIs (3)
B9	Achieving patient behavior change is difficult (*)	Potential effects of LIs are difficult to accomplish (1)
B10	GPs should (perhaps) not follow-up patients to monitor extent of continuation of exercises (*)	Perception of own role potentially impeding prescription or follow-up of LIs (2)
B11	It is the patient's own responsibility to continue doing their exercise programme (*)	Perception of own role potentially impeding prescription or follow-up of LIs (2)
B12	Increasing the overall activity levels does not/might not stop the knee problem getting worse (*)	LIs have little or no effect on OA (1)
B13	Time constraints prevent GPs from providing advice on individual exercises for CKP (*)	Lack of time within patient consultations (5)
F1	Physiotherapy (referral) needs to be prioritised	Positive attitude toward LIs (2)
F2	It is part of my job to reassure patients about the safety of exercise for CKP (*)	Perception of own role potentially stimulating prescription or follow-up of LIs (2)
F3	Exercise for CKP is most beneficial when it is tailored to meet individual patient needs (*)	Ability and importance of providing personalized treatment within LIs (1)
F4	A standard set of exercises is not/might not be sufficient for every patient with chronic knee problems (*)	Ability and importance of providing personalized treatment within LIs (1)
F5	GPs should educate patients with CKP about how to change their lifestyle for the better (*)	Perception of own role potentially stimulating prescription or follow-up of LIs (2)
F6	It is important that people with CKP increase their overall activity levels (*)	Positive attitude toward LIs (2)
F7	How well a patient complies with their exercise programme determines how effective it will be (*)	Importance of high patient adherence or engagement for effectiveness of LIs (3)
F8	GPs should prescribe quadriceps strengthening exercises to every patient with CKP (*)	Perception of own role potentially stimulating prescription or follow-up of LIs (2)
F9	GPs should prescribe general exercise (e.g. walking or swimming) for every patient with CKP (*)	Perception of own role potentially stimulating prescription or follow-up of LIs (2)
F10	Knee problems are improved by quadriceps strengthening exercises (*)	LIs have positive effects on affected joint(s) (1)
F11	Knee problems are improved by general exercise (e.g. walking or swimming) (*)	LIs have positive effects on affected joint(s) (1)

For peer review only

F12	Quadriceps strengthening exercises for the knee are safe for everybody to do (*)	LIs are safe (1)
F13	General exercise (e.g. walking or swimming) is safe for everybody to do (*)	LIs are safe (1)
F14	Exercise is effective for patients if an X-ray shows severe knee OA (*)	Severity of disease and symptoms (3)
F15	Increasing the strength of the muscles around the knee stops the knee problem getting worse (*)	LIs have positive effects on affected joint(s) (1)
F16	Exercise for CKP should (perhaps) preferably be used before drug treatment has been tried (*)	Positive attitude toward LIs (2)
F17	Exercise for CKP is more effectively provided by physiotherapists than GPs (*)	Good interdisciplinary collaboration or healthcare provision, or suggestions for improvement (4)
U1	Exercise does not/might not work just as well for everybody, regardless of the amount of pain they have (*)	Severity of disease and symptoms (3)

Duarte (2019) [53]

Number	Description	Subcategory (domain)
B1	Main concern was participant adherence to physical activity routines after end of program	Low patient adherence or engagement (3)
F1	Improvement in the physical condition of participants	LIs have positive effects (not further specified) (1)
F2	Enthusiastic participation of the participants	High patient adherence or engagement (3)

Hill (2018) [54]

Number	Description	Subcategory (domain)
B1	Not (or perhaps not) interested in being the orthopedic surgeon in an ortho-bariatric centre (*)	Perception of own role potentially impeding prescription or follow-up of LIs (2)
F1	There was a BMI threshold above which they would not perform a TKA at all (*)	Perception of own role potentially stimulating prescription or follow-up of LIs (2)
F2	There was a BMI threshold above which they would not perform a TKA until the patient had attended a weight management program (*)	Perception of own role potentially stimulating prescription or follow-up of LIs (2)
F3	Weight loss should be the first-line treatment in the management of obese patients with symptomatic knee OA (*)	Positive attitude toward LIs (2)
F4	Obese patients with symptomatic knee OA should be referred to a specialist weight management service before orthopaedic assessment (*)	Positive attitude toward LIs (2)
F5	Obese patients with symptomatic knee OA should be assessed by a specialist multidisciplinary service, which should include an orthopaedic surgeon (*)	Good interdisciplinary collaboration or healthcare provision, or suggestions for improvement (4)
F6	Support for creation of regional centres where orthopedic surgeons and bariatric surgeons, with their respective teams, could assess obese patients with symptomatic knee pain (*)	Good interdisciplinary collaboration or healthcare provision, or suggestions for improvement (4)

For peer review only

Hill (2018) [55]

Number	Description	Subcategory (domain)
B1	I don't have/might not have the required knowledge and training around obesity care (*)	Lack of knowledge or skills around LIs or promoting behavioral change (2)
B2	Weight management services are not/might not be adequately commissioned in my area (*)	LIs are unavailable or inaccessible (1)
F1	Weight loss should be the first-line treatment in the management of symptomatic knee OA in obesity (*)	Positive attitude toward LIs (2)
F2	Community interventions are effective at achieving sufficient and sustained weight loss (*)	LIs have positive effects on general health (1)
F3	Obese patients with symptomatic knee OA should be referred to a specialist weight management service before orthopaedic assessment (*)	Good interdisciplinary collaboration or healthcare provision, or suggestions for improvement (4)
F4	Obese patients with symptomatic knee OA should be assessed by a specialist multidisciplinary service (*)	Good interdisciplinary collaboration or healthcare provision, or suggestions for improvement (4)
F5	Support for creation of regional centres where orthopaedic surgeons and bariatric surgeons, with their respective teams, could assess obese patients with symptomatic knee pain (*)	Positive attitude toward LIs (2)
F6	Intention to refer patients to an ortho-bariatric centre if it existed (*)	Perception of own role potentially stimulating prescription or follow-up of LIs (2)

Hofstede (2016) [56]

Number	Description	Subcategory (domain)
F1	Clear referral criteria/guideline (*)	Availability of information resources (5)
F2	Important to follow guidelines (*)	Positive attitude toward guidelines or protocols (2)
F3	Important to try non-surgical treatments first (*)	Positive attitude toward LIs (2)
F4	Only few drawbacks for the use of non-surgical treatments (*)	LIs are safe (1)
F5	Patients benefit from weight loss (*)	LIs have positive effects on general health (1)
F6	Non-surgical treatments motivate patients to do things themselves (*)	LIs have positive mental effects (1)
F7	Good results of physical therapy (*)	LIs have positive effects (not further specified) (1)
F8	Important to delay a surgery as long as possible (*)	Positive attitude toward LIs (2)
F9	Agreements with colleagues about the content of the care trajectory (*)	Good interdisciplinary collaboration or healthcare provision, or suggestions for improvement (4)
F10	Peer review/audit of professional association (*)	Audit (6)
F11	Positive attitudes of colleagues about non-surgical treatments (*)	Good interdisciplinary collaboration or healthcare provision, or suggestions for improvement (4)
F12	Clarity on what the patient has done at the physical therapist (*)	Improving communication between HCPs (4)
F13	Agreements/ deliberations with primary care (GP, physical therapist, dietician) (*)	Good interdisciplinary collaboration or healthcare provision, or suggestions for improvement (4)

For peer review only

F14	Availability of non-surgical treatments (*)	LIs are available or accessible, or suggestions for improvement (1)

Lawford (2018) [57]

Number	Description	Subcategory (domain)
B1	Using the telephone to consult with an OA patient and prescribe an exercise program would not/might not be easy for me (*)	Other challenges for HCPs regarding feasibility of telehealth (1)
B2	I would not/might not be as satisfied talking to an OA patient over the telephone as I would be talking to the patient in person in my consulting room (*)	Other challenges for HCPs regarding feasibility of telehealth (1)
B3	An exercise program prescribed by a PT over the telephone would not/might not improve a patient's OA (*)	Disadvantages of telehealth in terms of effectiveness (1)
B4	I would not/might not be able to adequately monitor a patient's OA over the telephone (*)	Challenges for HCPs regarding lack of physical/visual contact (1)
B5	I do not/might not like that there would be no physical contact with an OA patient when consulting over the telephone (*)	Challenges for HCPs regarding lack of physical/visual contact (1)
B6	I do not/might not like that there would be no physical contact with an OA patient when consulting over the internet video (*)	Challenges for HCPs regarding lack of physical/visual contact (1)
B7	I would not/might not be interested in being involved in a service offering PT-prescribed exercise over the telephone for my people with OA (*)	Other challenges for HCPs regarding feasibility of telehealth (1)
B8	Using the telephone would not/might not be an acceptable way for me to deliver an exercise program to patients with OA (*)	Other challenges for HCPs regarding feasibility of telehealth (1)
B9	Using the telephone would not/might not be a useful (practical) way for me to deliver an exercise program to patients with OA (*)	Other challenges for HCPs regarding feasibility of telehealth (1)
B10	Using the telephone would not/might not be an effective way for me to deliver an exercise program to patients with OA (*)	Other challenges for HCPs regarding feasibility of telehealth (1)
B11	Using the telephone would not/might not be a safe way for patients to receive a PT-prescribed exercise program for their OA (*)	Telehealth is not safe for patients or patient/data privacy (1)
F1	Exercise is beneficial for OA (*)	LIs have positive effects on affected joint(s) (1)
F2	I would get a good understanding of a patient's OA over the telephone (*)	Lack of physical/visual contact not a major issue for HCPs (1)
F3	I would get a good understanding of a patient's OA over the internet video (*)	Lack of physical/visual contact not a major issue for HCPs (1)
F4	A patient's privacy would not be violated if I prescribed them an exercise program over the telephone (*)	Telehealth is safe for patients or patient/data privacy (1)
F5	A patient's privacy would not be violated if I prescribed them an exercise program over the internet video (*)	Telehealth is safe for patients or patient/data privacy (1)
F6	Using the internet video to consult with an OA patient and prescribe an exercise program would be easy for me (*)	Positive attitude or needs of HCPs regarding feasibility of telehealth (1)

F7	I would be as satisfied talking to an OA patient over the internet video as I would be talking to the patient in person in my consulting room (*)	Positive attitude or needs of HCPs regarding feasibility of telehealth (1)
F8	An exercise program prescribed by a PT over the internet video would improve a patient's OA (*)	Benefits of telehealth in terms of effectiveness (1)
F9	An exercise program prescribed by a PT over the telephone would save a patient money (*)	Patient-related benefits regarding feasibility of telehealth (1)
F10	An exercise program prescribed by a PT over the internet video would save a patient money (*)	Patient-related benefits regarding feasibility of telehealth (1)
F11	I would be able to adequately monitor a patient's OA over the internet video (*)	Lack of physical/visual contact not a major issue for HCPs (1)
F12	Receiving an exercise program from a PT over the telephone would be a convenient form of health care for an OA patient (*)	Patient-related benefits regarding feasibility of telehealth (1)
F13	Receiving an exercise program from a PT over the internet video would be a convenient form of health care for an OA patient (*)	Patient-related benefits regarding feasibility of telehealth (1)
F14	Receiving an exercise program from a PT over the telephone would save the patient time (*)	Patient-related benefits regarding feasibility of telehealth (1)
F15	Receiving an exercise program from a PT over the internet video would save the patient time (*)	Patient-related benefits regarding feasibility of telehealth (1)
F16	I would be interested in being involved in a service offering PT-prescribed exercise over the internet video for my people with OA (*)	Positive attitude or needs of HCPs regarding feasibility of telehealth (1)
F17	Using the internet video would be an acceptable way for me to deliver an exercise program to patients with OA (*)	Positive attitude or needs of HCPs regarding feasibility of telehealth (1)
F18	Using the internet video would be a useful (practical) way for me to deliver an exercise program to patients with OA (*)	Positive attitude or needs of HCPs regarding feasibility of telehealth (1)
F19	Using the internet video would be an effective way for me to deliver an exercise program to patients with OA (*)	Positive attitude or needs of HCPs regarding feasibility of telehealth (1)
F20	Using the telephone would be an affordable way for patients to receive a PT-prescribed exercise program for their OA (*)	Patient-related benefits regarding feasibility of telehealth (1)
F21	Using the internet video would be an affordable way for patients to receive a PT-prescribed exercise program for their OA (*)	Patient-related benefits regarding feasibility of telehealth (1)
F22	Using the internet video would be a safe way for patients to receive a PT-prescribed exercise program for their OA (*)	Telehealth is safe for patients or patient/data privacy (1)

Reid (2014) [58]

Number	Description	Subcategory (domain)
B1	Lack of availability of physiotherapy	LIs are unavailable or inaccessible (1)
B2	Poor rate of previous (physiotherapy) success (*)	Other patient characteristics (3)
B3	There is a paucity of evidence in regards to the effectiveness of physiotherapy treatment for	LIs have little or no effect on OA (1)

For peer review only

	OA hip and/or knee (*)	
B4	Past experience has shown physiotherapy to be ineffective (*)	LIs have little or no effect on OA (1)
F1	Referring patients to physiotherapy if they had high levels of pain/disability and where radiographic evidence of OA was present (*)	Severity of disease and symptoms (3)
F2	Referring patients to physiotherapy if they were of a younger age (*)	Other patient characteristics (3)
F3	Good access to physiotherapy in area (*)	LIs are available or accessible, or suggestions for improvement (1)
F4	Physiotherapists do not/might not lack expertise in OA management (*)	Good interdisciplinary collaboration or healthcare provision, or suggestions for improvement (4)
F5	Conservative treatment is (perhaps) an important part of OA management (*)	Positive attitude toward LIs (2)

De Rooij (2020) [59]

Number	Description	Subcategory (domain)
B1	In my daily clinical practice I can (perhaps) not integrate working according to the protocol well (*)	LIs are not feasible or sustainable (1)
B2	The lay out of the protocol does not/might not facilitate its usage in daily practice (*)	Challenges for HCPs during delivery of LIs (1)
B3	I do not/might not treat enough patients with knee OA and comorbidity to apply the protocol (*)	LIs are not feasible or sustainable (1)
B4	The protocol does not/might not fit well with my working methods of daily clinical practice (*)	LIs are not feasible or sustainable (1)
B5	I do not/might not have sufficient knowledge about knee OA exercise therapy and comorbidity to apply the protocol in daily clinical practice (*)	Lack of knowledge or skills around LIs or promoting behavioral change (2)
B6	I do not/might not have sufficient skills to apply the protocol in daily clinical practice (*)	Lack of knowledge or skills around specific resources (2)
B7	I do not/might not read the protocol sufficiently to remember any of its contents (*)	Lack of knowledge or skills around specific resources (2)
B8	The number of treatments that the patient receives from their insurance company is a barrier in using the protocol (*)	Restrictions due to health insurance (7)
B9	The patients are not/might not be cooperative in applying the protocol in daily clinical practice (*)	Negative attitude toward LIs (3)
B10	My colleagues in physiotherapy are not/might not be cooperative in applying the protocol in daily clinical practice (*)	Non-optimal interdisciplinary collaboration or healthcare provision (4)
B11	The management of my practice is not/might not be collaborative regarding the application of the protocol in daily clinical practice (*)	Management not supportive (6)
B12	The general practitioners or other physicians are not/might not be collaborative regarding the application of the protocol in daily clinical practice (*)	Non-optimal interdisciplinary collaboration or healthcare provision (4)
B13	Suboptimal collaboration with general practitioners and physicians	Non-optimal interdisciplinary collaboration or healthcare provision (4)
B14	Referring physicians do not always believe in/may lack knowledge about effectiveness of exercise therapy	Non-optimal interdisciplinary collaboration or healthcare provision (4)
B15	Patients do not always believe in/may lack knowledge about effectiveness of exercise therapy	Low health literacy (3)
B16	Total amount of knee OA patients with comorbidity was lower than expected	LIs are not feasible or sustainable (1)

For peer review only

B17	Number of treatment sessions patients receive from insurance companies restricted application of the strategy	Restrictions due to health insurance (7)
B18	Patients with knee OA and comorbidity are not always motivated to perform exercises	Negative attitude toward LIs (3)
B19	Requesting medical information about patients from specialists takes a lot of time	Challenges of communication and referral procedures (4)
B20	Physicians are not always collaborating in discussing medical conditions of patients	Lack of communication between HCPs (4)
F1	The protocol is feasible in daily clinical practice (*)	LIs are feasible or sustainable (1)
F2	The protocol supports me in clinical reasoning (*)	Available resources might improve knowledge and decision-making (2)
F3	The protocol gives the opportunity to make your own decisions regarding history taking, physical examination, and treatment (*)	Ability and importance of providing personalized treatment within LIs (1)
F4	Some contents of the protocol are not/might not be incorrect (*)	Positive experiences with or suggestions to improve the content or structure of LIs (1)
F5	In my daily clinical practice, I work with sufficient equipment (including blood pressure meter, saturation meter) to properly apply the protocol (*)	LIs are feasible or sustainable (1)
F6	The protocol is supporting the improvement of my knowledge regarding knee OA exercise therapy and comorbidity (*)	Available resources might improve knowledge and decision-making (2)
F7	The recommendations over adapting the diagnostic phase (history taking and physical examination) in the protocol are clear and understandable (*)	Ease for HCPs during delivery of LIs (1)
F8	The recommendations over adapting the OA exercise therapy in the protocol are clear and understandable (*)	Ease for HCPs during delivery of LIs (1)
F9	The protocol is supportive in which comorbidity-related symptoms I need to monitor before, during and after treatment (*)	Available resources might improve knowledge and decision-making (2)
F10	Working with the protocol invites me to discuss more with experts in the field of the comorbidity (*)	Good interdisciplinary collaboration or healthcare provision, or suggestions for improvement (4)
F11	In general, I do not/might not feel resistance towards working according to protocols (*)	Positive attitude toward guidelines or protocols (2)
F12	I have changed my working method (due to the protocol) (*)	LIs are feasible or sustainable (1)
F13	Working according to the protocol is not/might not be too time-consuming (*)	Adequate duration of specific interventions or protocols (5)
F14	Working according to the protocol should be financially rewarded (*)	Financial reward for implementing LIs (5)
F15	The protocol is applicable to OA patients with comorbidity that I see in my clinical practice (*)	Positive experiences with or suggestions to improve the content or structure of LIs (1)
F16	Intake procedure is feasible and implementable	LIs are feasible or sustainable (1)
F17	Important to extend the intake phase to at least to 45 min	Positive experiences with or suggestions to improve the content or structure of LIs (1)
F18	The more you apply the strategy in daily practice, the easier it is to integrate it in your daily working method	LIs are feasible or sustainable (1)
F19	More insight into exercise tolerance/more background knowledge to make clinical decision by using strategy	Available resources might improve knowledge and decision-making (2)
F20	Inform referrers better about benefits of exercise therapy in patients with knee OA and	Good interdisciplinary collaboration or healthcare provision, or

For peer review only

	comorbidity	suggestions for improvement (4)
F21	Inform patients with knee OA and comorbidity better about benefits of exercise therapy	High health literacy or importance of education (3)
F22	Optimize collaboration with orthopaedic surgeons and other health care providers	Good interdisciplinary collaboration or healthcare provision, or suggestions for improvement (4)
F23	In complex patients insurance companies should reimburse more treatment sessions	Benefits of good health insurance (7)
F24	Useful to plan follow up/refreshment training to repeat/discuss content of course/protocol and application	Ease for HCPs during delivery of LIs (1)
F25	Shortening the protocol would increase user-friendliness	Ease for HCPs during delivery of LIs (1)

Holden (2009) [60]

Number	Description	Subcategory (domain)
B1	Biomedical perspective on knee OA, attributing signs and symptoms to local knee pathology or wear and tear	OA seen as untreatable and local condition (wear-and-tear) (9)
B2	OA seen as chronic degenerative condition that would progressively worsen over time (only cure being surgery)	OA seen as untreatable and local condition (wear-and-tear) (9)
B3	Effectiveness related to severity of joint damage/pain level (less effective when more damage/pain)	Severity of disease and symptoms (3)
B4	Fear of increasing symptoms (as barrier to prescribing exercise)	LIs are unsafe or have negative effects (1)
B5	Causing disease progression, particularly through weight-bearing activities (as barrier to prescribing exercise)	LIs are unsafe or have negative effects (1)
B6	Exacerbating patient's comorbidities (as barrier to prescribing exercise)	LIs are unsafe or have negative effects (1)
B7	Reluctant to promote exercise in the presence of pain	Severity of disease and symptoms (3)
B8	Negative perceptions of patients' levels of exercise adherence	Low patient adherence or engagement (3)
B9	Lack of motivation or laziness (as patient-centered barrier to adherence)	Negative attitude toward LIs (3)
B10	Human nature (as patient-centered barrier to adherence)	Potential effects of LIs are difficult to accomplish (1)
B11	Pain (as patient-centered barrier to adherence)	Severity of disease and symptoms (3)
B12	Fear of harm (as patient-centered barrier to adherence)	Low health literacy (3)
B13	Negative treatment expectations (as patient-centered barrier to adherence)	Negative attitude toward LIs (3)
B14	Therapist's role seen as assessment/exercise prescription/education (relatively short-term responsibilities)	Perception of own role potentially impeding prescription or follow-up of LIs (2)
B15	Patient's role to follow prescribed exercise program over long term/get on board with treatment	Perception of own role potentially impeding prescription or follow-up of LIs (2)
B16	Limited time to review individual patients reduced opportunities to facilitate behavior change	Lack of time within patient consultations (5)
B17	Large caseloads and pressure of waiting lists reduced the number of treatment sessions provided	Lack of time within patient consultations (5)
B18	Limited opportunity to provide follow-up sessions after discharge	Lack of time within patient consultations (5)
B19	Poor links to community facilities such as local leisure centres	LIs are unavailable or inaccessible (1)

For peer review only

B20	Gaps in knowledge/skills (including how to facilitate behavior change, particularly with less motivated patients)	Lack of knowledge or skills around LIs or promoting behavioral change (2)
B21	Exercises are not/might not be effective for patients if an X-ray shows severe knee osteoarthritis (*)	Severity of disease and symptoms (3)
B22	Increasing overall activity levels does not/might not stop the knee problem getting worse (*)	LIs have little or no effect on OA (1)
B23	Increasing the strength of the muscles around the knee does not/might not stop the knee problem getting worse (*)	LIs have little or no effect on OA (1)
B24	General exercise is not/might not be safe for everybody to do (*)	LIs are unsafe or have negative effects (1)
B25	Local strengthening exercises for the knee are not/might not be safe for everybody to do (*)	LIs are unsafe or have negative effects (1)
B26	Knee problems are not/might not be improved by general exercise (*)	LIs have little or no effect on OA (1)
B27	Physical therapists should (perhaps) not prescribe general exercise for every patient with knee OA (*)	Perception of own role potentially impeding prescription or follow-up of LIs (2)
B28	It is the patient's own responsibility to continue doing their exercise program (*)	Perception of own role potentially impeding prescription or follow-up of LIs (2)
B29	It is not/might not be the physiotherapist's responsibility to make sure that the patient will continue doing their exercise program (*)	Perception of own role potentially impeding prescription or follow-up of LIs (2)
B30	It is not/might not be important that people with knee OA increase their overall activity levels (*)	Negative attitude toward LIs (2)
F1	Effectiveness related to severity of joint damage/pain level (more effective when less damage/pain)	Severity of disease and symptoms (3)
F2	Importance of exercise adherence/link between level of adherence and clinical outcomes (dose-response effect)	Importance of high patient adherence or engagement for effectiveness of LIs (3)
F3	Recognizing potential influence on exercise adherence, sharing responsibility of exercise adherence with patient	Perception of own role potentially stimulating prescription or follow-up of LIs (2)
F4	Exercises are effective for patients if an X-ray shows moderate knee osteoarthritis (*)	Severity of disease and symptoms (3)
F5	Exercises are effective for patients if an X-ray shows mild knee osteoarthritis (*)	Severity of disease and symptoms (3)
F6	Knee problems are improved by local strengthening exercises (*)	LIs have positive effects on affected joint(s) (1)
F7	Physical therapists should prescribe local strengthening exercise for every patient with knee OA (*)	Perception of own role potentially stimulating prescription or follow-up of LIs (2)
F8	How well a patient complies with their exercise program determines how effective it will be (*)	Importance of high patient adherence or engagement for effectiveness of LIs (3)
F9	Physiotherapists should educate chronic patients with knee OA about how to change their lifestyle for the better (*)	Perception of own role potentially stimulating prescription or follow-up of LIs (2)
F10	A standard set of exercises is not/might not be sufficient for every patient with knee OA (*)	Ability and importance of providing personalized treatment within LIs (1)
F11	Exercise for knee OA is most beneficial when it is tailored to meet individual patient needs (*)	Ability and importance of providing personalized treatment within LIs (1)
U1	Exercise does not/might not work just as well for everybody, regardless of the amount of pain they have (*)	Severity of disease and symptoms (3)

For peer review only

Kloek (2020) [61]

Number	Description	Subcategory (domain)
B1	Less satisfied about the applicability of e-Exercise for only one diagnosis	Insufficient ability to provide personalized treatment within LIs (1)
B2	Less satisfied about time needed to get used to e-Exercise during high work pressure/administrative burden	Lack of time due to other demands (or not further specified) (5)
B3	Lack of technology affinity (reason for patients' non-willingness to participate in e-Exercise)	Patient-related challenges regarding feasibility of telehealth (1)
B4	Patients preferred regular face-to-face contact	Patient-related challenges regarding feasibility of telehealth (1)
B5	Perceiving web-based application as an additional burden	Lack of time due to other demands (or not further specified) (5)
B6	Technical skills (lack of)	Other challenges for HCPs regarding feasibility of telehealth (1)
B7	Clarity of instruction manual and course (lack of)	Challenges for HCPs during delivery of LIs (1)
B8	Adaptive capacity to change treatment routines (lack of)	Other challenges for HCPs regarding feasibility of telehealth (1)
B9	Busy work schedules and administrative burden hindered testing/using e-Exercise in their practice	Lack of time due to other demands (or not further specified) (5)
B10	Reduced face-to-face contact interfered with professional autonomy	Challenges for HCPs regarding lack of physical/visual contact (1)
B11	Absence of national e-Health guideline or standard	Other challenges for HCPs regarding feasibility of telehealth (1)
B12	Loss of income due to substitution of face-to-face session	Other challenges for HCPs regarding feasibility of telehealth (1)
B13	E-Exercise does not/might not contain all essential elements for the treatment of hip/knee OA (*)	Non-optimal content or structure of LIs (1)
B14	I do not/might not have enough influence on the content of patients' individual e-Exercise program (*)	Insufficient ability to provide personalized treatment within LIs (1)
B15	The content of e-Exercise is not/might not be aligned with my opinion about treating patients with OA (*)	Non-optimal content or structure of LIs (1)
B16	The intervention provided through e-Exercise is not/might not be appropriate for the average patient with OA (*)	Non-optimal content or structure of LIs (1)
B17	I do not/might not experience that e-Exercise supports patients in doing their exercises at home (*)	Disadvantages of telehealth in terms of effectiveness (1)
B18	Patients who were treated with e-Exercise were (perhaps) not generally positive about the intervention (*)	Non-optimal content or structure of LIs (1)
F1	More flexibility in web-based application (intervention duration, number of sets/repetitions, type of exercises)	Ability and importance of providing personalized treatment within LIs (1)
F2	Completeness of web-based application (exercises/assignments/information)	Positive experiences with or suggestions to improve the content or structure of LIs (1)
F3	Perception that e-Exercise is an appropriate treatment option for subgroup of OA patients	Positive experiences with or suggestions to improve the content or structure of LIs (1)
F4	Added value in terms of exercise adherence (important factor to use web-based	Benefits of telehealth in terms of effectiveness (1)

For peer review only

	application)	
F5	Perceiving web-based application as time-saving	Adequate duration of specific interventions or protocols (5)
F6	More flexibility in intervention (more possibilities to personalize to individual needs)	Ability and importance of providing personalized treatment within LIs (1)
F7	Support from colleagues	Good interdisciplinary collaboration or healthcare provision, or suggestions for improvement (4)
F8	Advantage of reducing number of treatments	Positive attitude or needs of HCPs regarding feasibility of telehealth (1)
F9	Offering an innovative intervention attracted new patients	Positive attitude or needs of HCPs regarding feasibility of telehealth (1)
F10	The instruction course and manual assisted me so that I knew how to work with e-Exercise (*)	Ease for HCPs during delivery of LIs (1)
F11	That it results in less income is not/might not be a major disadvantage of e-Exercise (*)	Positive attitude or needs of HCPs regarding feasibility of telehealth (1)
F12	Our physiotherapy practice has the intention to use e-Health innovations (*)	Positive attitude or needs of HCPs regarding feasibility of telehealth (1)
F13	I do not/might not have insufficient time available to get familiar with e-Exercise and to use the web-application (*)	Adequate duration of specific interventions or protocols (5)
F14	I believe that patient data gathered at the e-Exercise web-application is stored safely (*)	Telehealth is safe for patients or patient/data privacy (1)

For peer review only

Preferred Reporting Items for Systematic reviews and Meta-Analyses extension for Scoping Reviews (PRISMA-ScR) Checklist

SECTION	ITEM	PRISMA-ScR CHECKLIST ITEM	REPORTED ON PAGE #
**TITLE**			
Title	1	Identify the report as a scoping review.	Page 1
**ABSTRACT**			
Structured summary	2	Provide a structured summary that includes (as applicable): background, objectives, eligibility criteria, sources of evidence, charting methods, results, and conclusions that relate to the review questions and objectives.	Page 2
**INTRODUCTION**			
Rationale	3	Describe the rationale for the review in the context of what is already known. Explain why the review questions/objectives lend themselves to a scoping review approach.	Page 4-5
Objectives	4	Provide an explicit statement of the questions and objectives being addressed with reference to their key elements (e.g., population or participants, concepts, and context) or other relevant key elements used to conceptualize the review questions and/or objectives.	Page 5
**METHODS**			
Protocol and registration	5	Indicate whether a review protocol exists; state if and where it can be accessed (e.g., a Web address); and if available, provide registration information, including the registration number.	Page 5
Eligibility criteria	6	Specify characteristics of the sources of evidence used as eligibility criteria (e.g., years considered, language, and publication status), and provide a rationale.	Page 6
Information sources*	7	Describe all information sources in the search (e.g., databases with dates of coverage and contact with authors to identify additional sources), as well as the date the most recent search was executed.	Page 5
Search	8	Present the full electronic search strategy for at least 1 database, including any limits used, such that it could be repeated.	Supplemental File 1
Selection of sources of evidence†	9	State the process for selecting sources of evidence (i.e., screening and eligibility) included in the scoping review.	Page 6-7
Data charting process‡	10	Describe the methods of charting data from the included sources of evidence (e.g., calibrated forms or forms that have been tested by the team before their use, and whether data charting was done independently or in duplicate) and any processes for obtaining and confirming data from investigators.	Page 7-8
Data items	11	List and define all variables for which data were sought and any assumptions and simplifications made.	Page 7-8
Critical appraisal of individual sources of evidence§	12	If done, provide a rationale for conducting a critical appraisal of included sources of evidence; describe the methods used and how this information was used in any data synthesis (if appropriate).	Page 7
Synthesis of results	13	Describe the methods of handling and summarizing the data that were charted.	Page 8-9

St. Michael's
Inspired Care.
Inspiring Science.

For peer review only - http://bmjopen.bmj.com/site/about/guidelines.xhtml

SECTION	ITEM	PRISMA-ScR CHECKLIST ITEM	REPORTED ON PAGE #
**RESULTS**			
Selection of sources of evidence	14	Give numbers of sources of evidence screened, assessed for eligibility, and included in the review, with reasons for exclusions at each stage, ideally using a flow diagram.	Figure 1 Page 9
Characteristics of sources of evidence	15	For each source of evidence, present characteristics for which data were charted and provide the citations.	Table 1 Page 10
Critical appraisal within sources of evidence	16	If done, present data on critical appraisal of included sources of evidence (see item 12).	Supplemental File 2 Page 10-11
Results of individual sources of evidence	17	For each included source of evidence, present the relevant data that were charted that relate to the review questions and objectives.	Supplemental File 4
Synthesis of results	18	Summarize and/or present the charting results as they relate to the review questions and objectives.	Table 2 Table 3 Supplemental File 3 Page 11-14
**DISCUSSION**			
Summary of evidence	19	Summarize the main results (including an overview of concepts, themes, and types of evidence available), link to the review questions and objectives, and consider the relevance to key groups.	Page 15-17
Limitations	20	Discuss the limitations of the scoping review process.	Page 18
Conclusions	21	Provide a general interpretation of the results with respect to the review questions and objectives, as well as potential implications and/or next steps.	Page 18-19
**FUNDING**			
Funding	22	Describe sources of funding for the included sources of evidence, as well as sources of funding for the scoping review. Describe the role of the funders of the scoping review.	Page 20

JBI = Joanna Briggs Institute; PRISMA-ScR = Preferred Reporting Items for Systematic reviews and Meta-Analyses extension for Scoping Reviews.
* Where *sources of evidence* (see second footnote) are compiled from, such as bibliographic databases, social media platforms, and Web sites.
† A more inclusive/heterogeneous term used to account for the different types of evidence or data sources (e.g., quantitative and/or qualitative research, expert opinion, and policy documents) that may be eligible in a scoping review as opposed to only studies. This is not to be confused with *information sources* (see first footnote).
‡ The frameworks by Arksey and O'Malley (6) and Levac and colleagues (7) and the JBI guidance (4, 5) refer to the process of data extraction in a scoping review as data charting.
§ The process of systematically examining research evidence to assess its validity, results, and relevance before using it to inform a decision. This term is used for items 12 and 19 instead of "risk of bias" (which is more applicable to systematic reviews of interventions) to include and acknowledge the various sources of evidence that may be used in a scoping review (e.g., quantitative and/or qualitative research, expert opinion, and policy document).

From: Tricco AC, Lillie E, Zarin W, O'Brien KK, Colquhoun H, Levac D, et al. PRISMA Extension for Scoping Reviews (PRISMAScR): Checklist and Explanation. Ann Intern Med. 2018;169:467–473. doi: 10.7326/M18-0850.

For peer review only - http://bmjopen.bmj.com/site/about/guidelines.xhtml

BMJ Open

Barriers and facilitators perceived by healthcare professionals for implementing lifestyle interventions in patients with osteoarthritis: a scoping review

Journal:	*BMJ Open*
Manuscript ID	bmjopen-2021-056831.R1
Article Type:	Original research
Date Submitted by the Author:	10-Dec-2021
Complete List of Authors:	Bouma, Sjoukje; University of Groningen, University Medical Center Groningen, Department of Orthopedics<br>van Beek, Juliette; University of Groningen, University Medical Center Groningen, Department of Orthopedics; University of Groningen, University Medical Center Groningen, Center for Human Movement Sciences<br>Diercks, Ron; University of Groningen, University Medical Center Groningen, Department of Orthopedics<br>van der Woude, Lucas; University of Groningen, University Medical Center Groningen, Center for Human Movement Sciences; University of Groningen, University Medical Center Groningen, Department of Rehabilitation Medicine<br>Stevens, Martin; University of Groningen, University Medical Center Groningen, Department of Orthopedics<br>van den Akker-Scheek, Inge; University of Groningen, University Medical Center Groningen, Department of Orthopedics
**Primary Subject Heading**:	Rehabilitation medicine
Secondary Subject Heading:	Sports and exercise medicine
Keywords:	ORTHOPAEDIC & TRAUMA SURGERY, Hip < ORTHOPAEDIC & TRAUMA SURGERY, Knee < ORTHOPAEDIC & TRAUMA SURGERY, Musculoskeletal disorders < ORTHOPAEDIC & TRAUMA SURGERY, REHABILITATION MEDICINE, SPORTS MEDICINE

SCHOLARONE™
Manuscripts

I, the Submitting Author has the right to grant and does grant on behalf of all authors of the Work (as defined in the below author licence), an exclusive licence and/or a non-exclusive licence for contributions from authors who are: i) UK Crown employees; ii) where BMJ has agreed a CC-BY licence shall apply, and/or iii) in accordance with the terms applicable for US Federal Government officers or employees acting as part of their official duties; on a worldwide, perpetual, irrevocable, royalty-free basis to BMJ Publishing Group Ltd ("BMJ") its licensees and where the relevant Journal is co-owned by BMJ to the co-owners of the Journal, to publish the Work in this journal and any other BMJ products and to exploit all rights, as set out in our licence.

The Submitting Author accepts and understands that any supply made under these terms is made by BMJ to the Submitting Author unless you are acting as an employee on behalf of your employer or a postgraduate student of an affiliated institution which is paying any applicable article publishing charge ("APC") for Open Access articles. Where the Submitting Author wishes to make the Work available on an Open Access basis (and intends to pay the relevant APC), the terms of reuse of such Open Access shall be governed by a Creative Commons licence – details of these licences and which Creative Commons licence will apply to this Work are set out in our licence referred to above.

Other than as permitted in any relevant BMJ Author's Self Archiving Policies, I confirm this Work has not been accepted for publication elsewhere, is not being considered for publication elsewhere and does not duplicate material already published. I confirm all authors consent to publication of this Work and authorise the granting of this licence.

For peer review only

Barriers and facilitators perceived by healthcare professionals for implementing lifestyle interventions in patients with osteoarthritis: a scoping review

S.E. (Sjoukje) Bouma[1], J.F.E. (Juliette) van Beek[1,2], R.L. (Ron) Diercks[1], L.H.V. (Lucas) van der Woude[2,3,4], M. (Martin) Stevens[1*], I. (Inge) van den Akker-Scheek[1*]

[1] University of Groningen, University Medical Center Groningen, Department of Orthopedics, Groningen, The Netherlands

[2] University of Groningen, University Medical Center Groningen, Center for Human Movement Sciences, Groningen, The Netherlands

[3] University of Groningen, University Medical Center Groningen, Department of Rehabilitation Medicine, Groningen, The Netherlands

[4] Peter Harrison Centre for Disability Sport, School of Sport, Exercise and Health Sciences, Loughborough University, United Kingdom

[*] These authors contributed equally to this work.

Corresponding author:

S.E. (Sjoukje) Bouma

University of Groningen, University Medical Center Groningen

Department of Orthopedics (BB51)

PO Box 30.001

9700 RB Groningen, The Netherlands

E-mail: s.e.bouma@umcg.nl

For peer review only

Abstract

Objective: To provide an overview of barriers and facilitators that healthcare professionals (HCPs) perceive regarding the implementation of lifestyle interventions (LIs) in patients with hip and/or knee osteoarthritis (OA).

Design: Scoping review.

Data sources: The databases PubMed, Embase, CINAHL, PsycINFO, and the Cochrane Library were searched from inception up to January 2021.

Eligibility criteria: Primary research articles with a quantitative, qualitative or mixed-methods design were eligible for inclusion if they reported: (1) perceptions of primary and/or secondary HCPs (*population*); (2) on implementing LIs with physical activity and/or weight management as key components (*concept*); (3) on conservative management of hip and/or knee OA (*context*). Articles not published in English, German, or Dutch were excluded.

Data extraction and synthesis: Barriers and facilitators were extracted by two researchers independently. Subsequently, the extracted factors were linked to a framework based on the Tailored Implementation for Chronic Diseases checklist.

Results: Thirty-six articles were included. In total, 809 factors were extracted and subdivided into nine domains. The extracted barriers were mostly related to non-optimal interdisciplinary collaboration, patients' negative attitude toward LIs, patients' low health literacy, and HCPs' lack of knowledge and skills around LIs or promoting behavioral change. The extracted facilitators were mostly related to good interdisciplinary collaboration, a positive perception of HCPs' own role in implementing LIs, the content or structure of LIs, and HCPs' positive attitude toward LIs.

Conclusions: Multiple individual and environmental factors influence the implementation of LIs by HCPs in patients with hip and/or knee OA. The resulting overview of barriers and facilitators can guide future research on the implementation of LIs within OA care. To

For peer review only

investigate whether factor frequency is related to the relevance of each domain, further research should assess the relative importance of the identified factors involving all relevant disciplines of primary and secondary HCPs.

PROSPERO registration number: CRD42019129348.

Abstract word count: 300 (max. 300 words)

Manuscript word count: 4152 (recommended: ≤ 4000 words)

Strengths and limitations of this study

- To our knowledge, this is the first scoping review to classify barriers and facilitators for implementing lifestyle interventions by healthcare professionals as conservative treatment for hip and/or knee osteoarthritis in which qualitative and quantitative data were combined.
- The study population consisted of all primary and secondary healthcare professionals involved in hip and/or knee osteoarthritis care.
- Given the broad definition of "implementing lifestyle interventions", the identified barriers and facilitators provide insight into the full spectrum of influencing factors rather than being applicable to every single way of implementing lifestyle interventions.
- Grey literature was not included in the search and selection process.

For peer review only

For peer review only - http://bmjopen.bmj.com/site/about/guidelines.xhtml

Introduction

Regular physical activity and weight management are recommended by national and international clinical guidelines for the conservative management of hip and/or knee osteoarthritis (OA)[1-5]. Previous studies have demonstrated that lifestyle interventions (LIs) focusing on exercise, alone or combined with dietary weight loss, are able to reduce hip and/or knee OA-related disability and to postpone or even prevent total joint arthroplasty[6-10]. However, these positive results are not always transferred from research settings to daily practice, which means that LIs are underutilized[11]. This suboptimal implementation of LIs as treatment for hip and/or knee OA can result from factors related to the patient, the healthcare professional (HCP) or the societal context[12]. Research on adhering to LIs has so far focused mainly on identifying barriers and facilitators at the patient level. However, these studies have also shown that HCPs can have a facilitating role in the lifestyle behavior of their patients, for example by providing advice, education, encouragement, and instructions[13,14].

Some research has already been conducted investigating the perspective of HCPs and the implementation of LIs in their daily practice. This knowledge is needed in order to enhance the implementation of LIs. As far as the authors know, no (systematic) literature review has previously been performed that identified and/or classified barriers and facilitators for implementing LIs in the conservative treatment of hip and/or knee OA from the perspective of all HCPs involved. One systematic review focused on the views toward OA management based on recommendations in clinical practice guidelines of HCPs working in primary care[15]. However, HCPs working in secondary care are also involved in the treatment of patients with OA, which draws attention to the importance of collaboration and communication between primary and secondary care practitioners[16]. Therefore, a scoping review was conducted

For peer review only - http://bmjopen.bmj.com/site/about/guidelines.xhtml

aiming to provide a comprehensive overview of barriers and facilitators perceived by primary and secondary HCPs regarding the implementation of LIs in patients with hip and/or knee OA. The Tailored Implementation for Chronic Diseases (TICD) checklist was used to guide data synthesis[17]. Within the context of this review, implementation was defined as the use of LIs as conservative treatment for hip and/or knee OA by individual HCPs.

Method

Study design

A scoping review has been defined as follows by Colquhoun et al.: "a form of knowledge synthesis that addresses an exploratory research question aimed at mapping key concepts, types of evidence and gaps in research related to a defined area or field by systematically searching, selecting, and synthesizing existing knowledge"[18]. Therefore, a scoping review was considered a suitable methodology to summarize existing literature on barriers and facilitators for implementing LIs in hip and/or knee OA and to identify potential gaps in the current literature on participation of primary and secondary HCPs. We conducted this scoping review according to the framework developed by Arksey and O'Malley[19]. Five stages were followed successively: (1) identifying the research question; (2) identifying relevant studies; (3) study selection; (4) charting the data; and (5) collating, summarizing and reporting the results[19]. The PRISMA Extension for Scoping Reviews (PRISMA-ScR) checklist was used as reporting guideline[20]. The review protocol was registered in the PROSPERO database (registration number: CRD42019129348).

Data sources and searches

For peer review only - http://bmjopen.bmj.com/site/about/guidelines.xhtml

A search strategy was developed consisting of four components: search terms related to (1) primary and secondary HCPs; (2) hip and/or knee OA; (3) LIs; and (4) barriers and facilitators. This search strategy was applied in five bibliographic electronic databases (i.e. PubMed, Embase, CINAHL, PsycINFO, and the Cochrane Library) to identify relevant articles from inception up to 19 January 2021. A detailed search strategy for each of the databases can be found in Supplemental File 1. Reference lists of included articles were manually searched for additional relevant articles. Primary research articles with a quantitative, qualitative or mixed-methods design were eligible for inclusion; study protocols, reviews, abstracts, and commentaries were excluded. Articles written in English, German, or Dutch were eligible for inclusion. No restrictions were applied regarding publication period.

Study selection

Eligibility criteria were described according to the Population-Concept-Context framework[21]. First, the study population was defined as all primary and secondary HCPs who are involved in the conservative treatment of patients with hip and/or knee OA. This definition includes, respectively, HCPs providing general medical care and HCPs providing more specialized care (with or without a referral). Articles focusing solely on the perspective of patients with hip and/or knee OA were excluded. Second, the concepts central to this review were barriers and facilitators for implementing LIs. Barriers and facilitators were defined as any belief, experience, factor, opinion, reason, or view reported by an HCP that potentially influences (either impedes or facilitates) implementation of LIs in patients with hip and/or knee OA. These barriers and facilitators were extracted from both quantitative (e.g. survey) and qualitative (e.g. interview) data. Implementing LIs was broadly defined, ranging from mentioning or discussing a healthy lifestyle to recommending or running specific lifestyle programs, as long as it was clearly described that physical activity and/or weight management

For peer review only - http://bmjopen.bmj.com/site/about/guidelines.xhtml

were key components. This definition includes physiotherapeutic exercise interventions (aerobic, functional, or strengthening programs), dietary interventions, and self-management programs. Physiotherapeutic modalities such as acupuncture, manual therapy, and massage, and self-management programs whose content was not specified were not considered LIs and were therefore excluded. Physical activity was also broadly defined, ranging from physical activity during activities of daily living to participation in supervised or non-supervised exercise therapy or sports. Articles not primarily focusing on implementing LIs (e.g. development and evaluation of clinical guidelines, general management of hip and/or knee OA, general patient-practitioner relationship or shared decision-making) also fell outside the scope of this review. Lastly, the context of this review was the conservative treatment of hip and/or knee OA in both primary and secondary healthcare settings. Articles focusing on preoperative or postoperative treatment of hip and/or knee OA were excluded. Two researchers (SB together with AJ or JvB) independently assessed the eligibility of the identified articles based on the above criteria in three consecutive rounds: based on (1) title; (2) abstract; and (3) full-text of the article. Any disagreements among the researchers were resolved in consensus meetings.

Data extraction and quality assessment

A data extraction form was created and pilot-tested in order to systematically record study characteristics (first author, year of publication, country of origin, aims/purpose, study design, data collection method, data analysis method, theoretical basis, study population, setting, recruitment method, type of LI, patient population) and outcomes (barriers, facilitators, and/or unclear factors (i.e. an influencing factor, but not clearly defined as barrier or facilitator)). Study quality was assessed with the Mixed Methods Appraisal Tool (MMAT). The MMAT is a critical appraisal tool that can be used in reviews of mixed studies to assess the

For peer review only - http://bmjopen.bmj.com/site/about/guidelines.xhtml

methodological quality of different study design categories: mixed-methods, qualitative, and quantitative studies (randomized controlled trials, non-randomized studies, and descriptive studies)[22,23]. Since calculating a total score is discouraged[23], it was chosen to present the ratings of the individual criteria.

Data extraction was performed in two stages. The first stage consisted of filling in the data extraction form and the MMAT for each article, done by two researchers (SB/JvB) independently. Regarding barriers and facilitators, both researchers extracted the relevant units of text and/or descriptive statistics from the Results sections. Any discrepancies between the researchers in this first stage were resolved in consensus meetings. During the second stage, the extraction of barriers and facilitators was discussed among the research team (SB/MS/IvdAS) and the process was further refined for both quantitative and qualitative data. Regarding quantitative data, factors were only extracted if ≥50% of participants indicated that the factor influenced the implementation of LIs[24,25]. For close-ended questions or attitude statements with multiple answer options, participants were classified as being "in agreement" or "not in agreement". If this classification had not yet been made by the authors of the original article, it was made based on the possible answer options, with "(strongly) agree", "to a reasonable/large extent" and "yes" indicating agreement, and "neither disagree or agree", "don't know", "neutral", "a little bit/not at all", "(strongly) disagree", and "no" indicating not in agreement. Next, the factor was classified as barrier or facilitator depending on the formulation of the question and which of the two groups ("in agreement" versus "not in agreement") comprised ≥50% of the participants. In case of open-ended questions, all mentioned factors were extracted. Regarding qualitative data, if the authors of the original study did not explicitly identify a factor as barrier or facilitator, the description in the text or the participants' quotes were used to classify the factor as barrier (i.e.

For peer review only - http://bmjopen.bmj.com/site/about/guidelines.xhtml

impeding/negative/problem/lack), facilitator (i.e. facilitating/positive/solution/need), or unclear (i.e. insufficient information). In addition, all unclear factors were re-discussed with a third researcher (IvdAS) to assess whether these factors could nevertheless be classified as barrier or facilitator. At the end of the second stage, final data extraction based on the above criteria was performed by one researcher (SB), who also checked the consistency of the entire data extraction process.

Data synthesis and analysis

A narrative synthesis of the data was undertaken, based on the TICD checklist developed by Flottorp et al.[17]. This checklist aims to assist in identifying key determinants of professional practice, defined as factors that might prevent or enable healthcare improvements, and is intended for use in research on implementation and quality improvement in healthcare. It consists of seven domains: (1) guideline factors; (2) individual health professional factors; (3) patient factors; (4) professional interactions; (5) incentives and resources; (6) capacity for organizational change; and (7) social, political, and legal factors. The authors of the current study have previously used the TICD checklist in the analysis of focus group data on the same topic, revealing two additional domains: (8) patient and HCP interactions; and (9) disease factors[26]. One researcher (SB) assigned all extracted factors to one of these nine domains and then inductively developed different categories and subcategories of factors per domain. The resulting classification of factors and corresponding conclusions were subsequently discussed among the research team (SB/MS/IvdAS).

Patient and public involvement

Patients or the public were not involved in this study as the study aim did not concern patients but HCPs.

For peer review only - http://bmjopen.bmj.com/site/about/guidelines.xhtml

Results

Study selection

A flowchart of the study selection process is presented in Figure 1. A total of 8338 articles were retrieved. After removal of duplicates and exclusion of articles based on title or abstract, 93 potentially relevant articles remained for full-text screening. Ultimately, 36 articles were included in the qualitative synthesis[27-62].

[**Figure 1.** Flowchart of the study selection process]

Study characteristics

General characteristics of the included studies are presented in Table 1. The majority of studies were conducted in Australia (36%), the Netherlands (19%), the United Kingdom (19%) and Canada (11%). Qualitative data were extracted in 26 studies (72%), quantitative data in 7 studies (19%), and both qualitative and quantitative data in the remaining 3 studies (8%). Individual interviews were most commonly used as qualitative data collection method, while the quantitative studies were all based on cross-sectional surveys. Most studies included physiotherapists or general practitioners (or physicians) as study population. Other participants were dieticians, exercise professionals, a nurse practitioner, an occupational therapist, orthopedic surgeons, practice nurses, program instructors, rheumatologists, telephone coaches, and triaging clinicians.

[Table 1 near here]

For peer review only - http://bmjopen.bmj.com/site/about/guidelines.xhtml

Quality assessment

Findings of the quality assessment of the included studies based on the MMAT are shown in Supplemental File 2. Regarding the qualitative data assessments, only one study had the maximum of five positive ratings. Seven studies had a negative rating for the item on substantiating the interpretation of results, as no or a limited number of participant quotes were presented. In addition, many unknown ratings were given due to a lack of information about the applied qualitative approach and/or data analysis methods and their rationale. Regarding the quantitative data assessments, most studies had a negative or unknown rating for the risk of non-response bias due to low response rates or a lack of information about the response rate and/or reasons for non-response. In addition, the item on representativeness of the sample was often given an unknown rating because insufficient information about the sample and/or non-responders was presented. Finally, all three mixed-methods studies had a negative rating since the qualitative and quantitative components did not adhere to their specific quality criteria. For the other four mixed-methods criteria, only one of these three studies obtained positive ratings.

Synthesis of results

A total of 809 factors were extracted from the 36 included articles. Table 2 presents the distribution of factors from the individual studies across the aforementioned nine domains, which were largely based on the TICD checklist. The highest number of factors was assigned to intervention factors (n=315), followed by individual HCP factors (n=144), and patient factors (n=137). The lowest number of factors was assigned to capacity for organizational change (n=7), followed by social, political, and legal factors (n=9), and patient and HCP interactions (n=19). In Table 3 the content of the nine domains is further explained by presenting an overview of the created categories and subcategories of factors that potentially

For peer review only

For peer review only - http://bmjopen.bmj.com/site/about/guidelines.xhtml

influence the implementation of LIs by HCPs within each domain. A full overview of all extracted factors can be found in Supplemental File 3 (presented per domain) and Supplemental File 4 (presented per article).

[Table 2 near here]

[Table 3 near here]

Categories

The distribution of barriers and facilitators across the various categories is presented in Figure 2. The highest number of barriers was assigned to the following five categories: telehealth (n=40), collaboration (n=32), expertise (n=32), accessibility (n=32) and treatment expectations and preferences (n=31). The highest number of facilitators was assigned to the following five categories: telehealth (n=60), collaboration (n=46), design (n=45), effectiveness (n=41) and role (n=28).

[**Figure 2.** Overview of the number of barriers and facilitators per category]

Figure caption – The domain numbers indicated in brackets refer to the domains as presented in Table 3: (1) intervention factors; (2) individual HCP factors; (3) patient factors; (4) professional interactions; (5) incentives and resources; (6) capacity for organizational change; (7) social, political, and legal factors; (8) patient and HCP interactions; (9) disease factors. Unclear factors were not included in this figure due to the low number (n=11).

Subcategories

For peer review only - http://bmjopen.bmj.com/site/about/guidelines.xhtml

Table 4 and Table 5 present the rankings of the ten largest subcategories of barriers and facilitators respectively. The first place in both rankings was assigned to a subcategory related to interdisciplinary collaboration or healthcare provision.

[Table 4 near here]

[Table 5 near here]

Discussion

The aim of this review was to provide an overview of barriers and facilitators that primary and secondary HCPs perceive for implementing LIs in patients with hip and/or knee OA. By linking the identified factors to a framework that was largely based on the TICD checklist[17], a comprehensive overview of influencing factors was created that could serve as a basis for improving the implementation of LIs within primary and secondary OA care. The variety of domains shows that multiple levels (i.e. both the level of the individual HCP and several environmental levels) should be considered in order to achieve this. Within this framework, the extracted barriers were most frequently related to non-optimal interdisciplinary collaboration, a negative attitude of patients toward LIs, low health literacy of patients, and a lack of knowledge and skills of HCPs around LIs or promoting behavioral change. The extracted facilitators were most frequently related to good interdisciplinary collaboration, a positive perception of HCPs' own role in implementing LIs, the content or structure of LIs, and a positive attitude of HCPs toward LIs.

A relatively large number of studies were included, a majority of which was published in recent years. From these 36 studies, a total of 809 influencing factors were extracted.

For peer review only - http://bmjopen.bmj.com/site/about/guidelines.xhtml

Although all nine domains were covered, the total number of factors identified within each domain differed greatly, ranging from 7 *(capacity for organizational change)* to 315 *(intervention factors)*. In addition, a large variation was found in the number of barriers and facilitators between the various categories and subcategories. However, we do not know yet whether the established factor frequency is directly related to the importance of the domain, category or subcategory in question. So the fact that we found the highest number of factors within certain domains, categories or subcategories does not necessarily mean that these are the most important or relevant in the context of implementation. It could also be an indication that studies to date have mainly focused on these aspects, and that the others are still underexposed in the available literature. Therefore, we recommend to take all domains into account in future research in order to avoid missing factors that might be highly relevant for the implementation of LIs. The quality assessment of the included studies showed many unknown ratings due to a lack of information about, for example, the applied methods and their rationale. This finding does not have to mean that the studies are of low quality, but it does emphasize the importance of accurate and complete reporting of research using design-specific reporting guidelines.

Our results reflect those of a previous systematic review conducted by Egerton et al.[15], in which the authors synthesized qualitative evidence only on primary care clinicians' views on providing recommended management of OA up to August 2016. In addition to exercise and weight loss, recommended management included education, self-management support, and medication. The authors identified four barriers as main themes: (1) "OA is not that serious"; (2) "clinicians are, or perceive they are, underprepared"; (3) "personal beliefs at odds with providing recommended practice"; and (4) "dissonant patient expectations". A few system-related factors (e.g. time, payment system) were mentioned, but these were not found to be

For peer review only - http://bmjopen.bmj.com/site/about/guidelines.xhtml

themes across multiple studies. The added value of the current review in comparison to the review by Egerton et al. is that factors related to interdisciplinary collaboration and the organizational and societal context were in fact identified. Although these domains were relatively small in terms of number of factors, the current review shows that these factors can also influence the implementation of LIs and thus offers an even broader perspective on the implementation status of LIs within OA care. Besides an expansion of the review's scope (i.e. the inclusion of quantitative data and the perspectives of secondary HCPs), this broader perspective of our review most likely arises from the date of the search. The vast majority (72%) of the included articles were in fact published in the past five years (after Egerton et al. had conducted their review), which shows that there is growing attention for the role of lifestyle as treatment for hip and/or knee OA. Very recently another scoping review has been published, conducted by Nissen et al.[63], which focused on clinicians' beliefs and attitudes about physical activity and exercise therapy as treatment for hip and/or knee OA. The authors thematically analyzed qualitative data from four types of HCPs (physiotherapists, general practitioners, orthopedic surgeons and rheumatologists). Their main finding is that many clinicians perceive OA to be a low priority "wear and tear" disease. In addition, they identified a relative lack of knowledge about and interest in physical activity and exercise management among many clinicians. These findings are also reflected in our results (especially in the domains *disease factors* and *individual HCP factors*). In addition, even more barriers and facilitators have been identified in the current review. Compared to this review by Nissen et al, our review again has a broader scope (i.e. the inclusion of weight management, quantitative data and the perspectives of more types of HCPs) and can therefore be seen as relevant addition to the existing literature on this topic.

For peer review only

For peer review only - http://bmjopen.bmj.com/site/about/guidelines.xhtml

In addition to summarizing the existing literature on barriers and facilitators for implementing LIs, this review aimed to identify potential gaps in literature on the participation of HCPs. Although we aimed to include perceptions of various primary and secondary HCPs, the results show that studies to date have mainly focused on the views of physiotherapists and general practitioners. These primary HCPs may well be the first point of contact for patients within the care pathway, yet we recommend that other relevant disciplines − like dieticians, lifestyle counselors, practice nurses, and orthopedic clinicians − be more involved in follow-up research, allowing for a more complete understanding of the patient journey in OA care. Special attention should then be drawn to potential differences in perceived barriers and facilitators between types of HCPs, so that implementation strategies can be tailored as much as possible to the various types of HCPs and their clinical practice.

The resulting overview of barriers and facilitators can be used to improve the implementation of LIs in daily practice. This overview presents factors that are relevant for individual HCPs, as well as for policymakers, who can facilitate the organizational and societal context in which primary and secondary HCPs work. When developing implementation strategies, possible interactions between the various domains should also be considered. For instance, more time *(domain 5)* can be used in various ways by HCPs: for their own education *(domain 2)*, provision of information to patients *(domain 3),* or interdisciplinary consultation *(domain 4)*. Another example is that societal changes in health insurance or payment structures *(domain 7)* can lead to increased accessibility of LIs *(domain 1)*, and that limited financial resources might be less of an obstacle for patients *(domain 3)*. Hence changes related to the established factors can have positive effects on multiple levels.

For peer review only - http://bmjopen.bmj.com/site/about/guidelines.xhtml

Within the domain of intervention factors, a separate category was created for factors specific to delivering LIs via telehealth. Attention for this modality of healthcare provision has been growing for some time[64]. In addition, during the course of the current review the COVID-19 pandemic emerged, which meant that many HCPs actually had to use telehealth in their daily practice[65]. Although telehealth was not a specific focus of this review, it could be interesting to further investigate the experiences with telehealth and its value for long-term counseling of patients with hip and/or knee OA on behavioral change[66].

To the best of our knowledge, this is the first review to focus specifically on the implementation of LIs as conservative treatment for hip and/or knee OA while taking into account the perceptions of all primary and secondary HCPs involved. Both qualitative and quantitative data were included, providing broad insight into the topic. All included studies were conducted in North America, Europe and Oceania. Given that the majority of these studies were conducted quite recently, our results are expected to be representative of the current situation in these continents.

There are also a few limitations to acknowledge. First, "implementing LIs" was defined very broadly and can be seen as an umbrella term, ranging from mentioning a healthy lifestyle to running specific lifestyle programs. Due to the heterogeneity of the included studies in terms of study design and evaluated LIs, no distinction was made between the different ways of implementing LIs during data analysis. Consequently, the identified barriers and facilitators may not fit with every single way of implementing LIs, but may rather provide insight into the full spectrum of influencing factors. Although data synthesis has not been performed separately for physical activity and weight management either, the created overview gives us the overall impression that barriers and facilitators related to these two lifestyle components

For peer review only - http://bmjopen.bmj.com/site/about/guidelines.xhtml

are quite similar. One barrier that seems to be unique to weight management is the perception of it being a difficult or sensitive subject to discuss. Regarding physical activity, the perception that it is unsafe or has negative effects seems to be a unique barrier. Second, although data extraction and quality assessment were performed by two researchers independently, data analysis was performed primarily by one researcher. By discussing the resulting classification of factors and any doubts during the process with members of the research team, we aimed to increase the reliability of our findings. Third, the chosen cut-off percentage for extracting quantitative data was based on other scoping reviews combining the results of quantitative and qualitative studies[24,25]. Therefore, there is a chance that factors that would have been extracted when using a lower cut-off percentage are missing. However, it is also possible that these factors were already extracted from the other included studies and therefore still included in our results. Lastly, as we did not search grey literature there is a slight chance that relevant studies may have been missed.

The comprehensive overview of barriers and facilitators for implementing LIs in patients with hip and/or knee OA by HCPs resulting from this review can serve as a basis for further research and the development of implementation strategies that focus on both the individual and the environmental context of HCPs. However, what the relative importance of the identified factors is and whether differences exist between the various types of primary and secondary HCPs with respect to these factors are not known yet. Further research is required to provide more insight into this relative importance and therewith the most relevant targets for change in daily practice.

Conclusion

For peer review only

For peer review only - http://bmjopen.bmj.com/site/about/guidelines.xhtml

This review has shown that multiple factors influence whether or not HCPs implement LIs when treating patients with hip and/or knee OA. Data analysis has resulted in a comprehensive overview of influencing factors, where barriers and facilitators have been subdivided into nine domains, both at an individual and at several environmental levels. The review contributes to existing knowledge about the implementation of LIs by identifying multiple factors related to the intervention, interdisciplinary collaboration, and the organizational and societal context. The broad inventory created in this review can be a first step toward an improved implementation of LIs by HCPs in OA care. Future research in this area should focus on determining the relative importance of the identified factors involving all relevant disciplines of primary and secondary HCPs.

Acknowledgments

The authors would like to thank Truus van Ittersum for her advice on search strategies and Annemarie Jenks for her contribution to the study selection process.

Contributors

Conception and design: SB, RD, LvdW, MS, IvdAS. Collection and assembly of data: SB, JvB. Analysis and interpretation of the data: SB, MS, IvdAS. Drafting of the article: SB. Critical revision of the article for important intellectual content: SB, JvB, RD, LvdW, MS, IvdAS. Final approval of the article: SB, JvB, RD, LvdW, MS, IvdAS. Obtaining of funding: SB, RD, LvdW, MS, IvdAS.

Funding

This study is supported by a scholarship from the University of Groningen/University Medical Center Groningen (grant/award number: not applicable). The funders played no role

For peer review only - http://bmjopen.bmj.com/site/about/guidelines.xhtml

or the decision to submit the manuscript for publication.

in the study design, collection, analysis and interpretation of data, writing of the manuscript, or the decision to submit the manuscript for publication.

Conflict of interest

The authors report no potential conflicts of interest.

Data availability statement

Data sharing is not applicable to this article as no new data were created or analyzed in this study.

Ethics approval

Ethics approval was not required for this study as it was a scoping review in which already published articles were included.

References

1. Koninklijk Nederlands Genootschap voor Fysiotherapie (KNGF). KNGF-richtlijn Artrose heup-knie. Conservatieve, pre- en postoperatieve behandeling [Internet]. 2018 [cited 2020 Aug 25]. Available from: https://www.kngf.nl/kennisplatform/richtlijnen/artrose-heup-knie.
2. Nederlands Huisartsen Genootschap (NHG). NHG-Standaard Niet-traumatische knieklachten [Internet]. 2016 [cited 2020 Aug 25]. Available from: https://www.nhg.org/standaarden/samenvatting/niet-traumatische-knieklachten.
3. Nederlandse Orthopaedische Vereniging (NOV). Conservatieve behandeling van artrose in heup of knie [Internet]. 2018 [cited 2020 Aug 25]. Available from:

For peer review only - http://bmjopen.bmj.com/site/about/guidelines.xhtml

https://richtlijnendatabase.nl/richtlijn/artrose_in_heup_of_knie/startpagina_-_heup-_of_knieartrose.html.

4. Bannuru RR, Osani MC, Vaysbrot EE, Arden NK, Bennell K, Bierma-Zeinstra SMA, et al. OARSI guidelines for the non-surgical management of knee, hip, and polyarticular osteoarthritis. Osteoarthr Cartilage 2019; 27: 1578-89.

5. Fernandes L, Hagen KB, Bijlsma JWJ, Andreassen O, Christensen P, Conaghan PG, et al. EULAR recommendations for the non-pharmacological core management of hip and knee osteoarthritis. Ann Rheum Dis 2013; 72: 1125-35.

6. Fransen M, McConnell S, Hernandez-Molina G, Reichenbach S. Exercise for osteoarthritis of the hip. Cochrane Database Syst Rev. 2014(4):CD007912. DOI:10.1002/14651858.CD007912.pub2.

7. Fransen M, McConnell S, Harmer AR, Van der Esch M, Simic M, Bennell KL. Exercise for osteoarthritis of the knee: a Cochrane systematic review. Br J Sports Med 2015; 49: 1554-7.

8. Messier SP, Loeser RF, Miller GD, Morgan TM, Rejeski WJ, Sevick MA, et al. Exercise and dietary weight loss in overweight and obese older adults with knee osteoarthritis: the Arthritis, Diet, and Activity Promotion Trial. Arthritis Rheum 2004; 50: 1501-10.

9. Paans N, van den Akker-Scheek I, Dilling RG, Bos M, van der Meer K, Bulstra SK, et al. Effect of exercise and weight loss in people who have hip osteoarthritis and are overweight or obese: a prospective cohort study. Phys Ther 2013; 93: 137-46.

10. Svege I, Nordsletten L, Fernandes L, Risberg MA. Exercise therapy may postpone total hip replacement surgery in patients with hip osteoarthritis: a long-term follow-up of a randomised trial. Ann Rheum Dis 2015; 74: 164-9.

For peer review only - http://bmjopen.bmj.com/site/about/guidelines.xhtml

11. Snijders GF, den Broeder AA, van Riel PL, Straten VH, de Man FH, van den Hoogen FH, et al. Evidence-based tailored conservative treatment of knee and hip osteoarthritis: between knowing and doing. Scand J Rheumatol 2011; 40: 225-31.

12. Bennell KL, Dobson F, Hinman RS. Exercise in osteoarthritis: moving from prescription to adherence. Best Pract Res Clin Rheumatol 2014; 28: 93-117.

13. Dobson F, Bennell KL, French SD, Nicolson PJ, Klaasman RN, Holden MA, et al. Barriers and facilitators to exercise participation in people with hip and/or knee osteoarthritis: synthesis of the literature using behavior change theory. Am J Phys Med Rehabil 2016; 95: 372-89.

14. Kanavaki AM, Rushton A, Efstathiou N, Alrushud A, Klocke R, Abhishek A, et al. Barriers and facilitators of physical activity in knee and hip osteoarthritis: a systematic review of qualitative evidence. BMJ Open. 2017;7(12):e017042. DOI:10.1136/bmjopen-2017-017042

15. Egerton T, Diamond LE, Buchbinder R, Bennell KL, Slade SC. A systematic review and evidence synthesis of qualitative studies to identify primary care clinicians' barriers and enablers to the management of osteoarthritis. Osteoarthr Cartilage 2017; 25: 625-38.

16. Landelijke Huisartsen Vereniging (LHV)/Nederlands Huisartsen Genootschap (NHG). Toekomstvisie Huisartsenzorg 2022 [Internet]. 2012 [cited 2020 Dec 21]. Available from: https://www.nhg.org/toekomstvisie.

17. Flottorp SA, Oxman AD, Krause J, Musila NR, Wensing M, Godycki-Cwirko M, et al. A checklist for identifying determinants of practice: a systematic review and synthesis of frameworks and taxonomies of factors that prevent or enable improvements in healthcare professional practice. Implement Sci. 2013;8:35. DOI:10.1186/1748-5908-8-35

For peer review only

For peer review only - http://bmjopen.bmj.com/site/about/guidelines.xhtml

18. Colquhoun HL, Levac D, O'Brien KK, Straus S, Tricco AC, Perrier L, et al. Scoping reviews: time for clarity in definition, methods, and reporting. J Clin Epidemiol 2014; 67: 1291-4.

19. Arksey H, O'Malley L. Scoping studies: towards a methodological framework. Int J Soc Res Method 2005; 8: 19-32.

20. Tricco AC, Lillie E, Zarin W, O'Brien KK, Colquhoun H, Levac D, et al. PRISMA Extension for Scoping Reviews (PRISMA-ScR): Checklist and Explanation. Ann Intern Med 2018; 169: 467-73.

21. Peters MDJ, Godfrey CM, McInerney P, Baldini Soares C, Khalil H, Parker D. The Joanna Briggs Institute Reviewers' Manual 2015: Methodology for JBI scoping reviews. Adelaide: The Joanna Briggs Institute 2015.

22. Pluye P, Gagnon M-P, Griffiths F, Johnson-Lafleur J. A scoring system for appraising mixed methods research, and concomitantly appraising qualitative, quantitative and mixed methods primary studies in Mixed Studies Reviews. Int J Nurs Stud 2009; 46: 529-46.

23. Hong QN, Pluye P, Fàbregues S, Bartlett G, Boardman F, Cargo M, et al. Mixed Methods Appraisal Tool (MMAT), version 2018. Registration of Copyright (#1148552), Canadian Intellectual Property Office, Industry Canada 2018.

24. Garne-Dalgaard A, Mann S, Bredahl TVG, Stochkendahl MJ. Implementation strategies, and barriers and facilitators for implementation of physical activity at work: a scoping review. Chiropr Man Therap. 2019;27. DOI:10.1186/s12998-019-0268-5

25. Weatherson KA, Gainforth HL, Jung ME. A theoretical analysis of the barriers and facilitators to the implementation of school-based physical activity policies in Canada: a mixed methods scoping review. Implementation Sci. 2017;12. DOI:10.1186/s13012-017-0570-3

For peer review only - http://bmjopen.bmj.com/site/about/guidelines.xhtml

26. Bouma SE, van Beek JFE, Alma MA, Diercks RL, van der Woude LHV, van den Akker-Scheek I, et al. What affects the implementation of lifestyle interventions in patients with osteoarthritis? A multidisciplinary focus group study among healthcare professionals. Disabil Rehabil (forthcoming). DOI:10.1080/09638288.2021.2011438

27. Allison K, Setchell J, Egerton T, Delany C, Bennell KL. In Theory, Yes; in Practice, Uncertain: A Qualitative Study Exploring Physical Therapists' Attitudes Toward Their Roles in Weight Management for People With Knee Osteoarthritis. Phys Ther 2019; 99: 601-11.

28. Bossen D, Kloek C, Snippe HW, Dekker J, de Bakker D, Veenhof C. A Blended Intervention for Patients With Knee and Hip Osteoarthritis in the Physical Therapy Practice: Development and a Pilot Study. JMIR Res Protoc 2016; 5: e32.

29. Christiansen MB, White DK, Christian J, Waugh E, Gakhal N, King L, et al. "It ... doesn't always make it [to] the top of the list" Primary care physicians' experiences with prescribing exercise for knee osteoarthritis. Canadian Family Physician 2020; 66: e14-20.

30. Davis AM, Kennedy D, Wong R, Robarts S, Skou ST, McGlasson R, et al. Cross-cultural adaptation and implementation of Good Life with osteoarthritis in Denmark (GLA:D™): group education and exercise for hip and knee osteoarthritis is feasible in Canada. Osteoarthritis Cartilage 2018; 26: 211-9.

31. de Rooij M, van der Leeden M, Avezaat E, Häkkinen A, Klaver R, Maas T, et al. Development of comorbidity-adapted exercise protocols for patients with knee osteoarthritis. Clin Interv Aging 2014; 9: 829-42.

32. Egerton T, Nelligan R, Setchell J, Atkins L, Bennell KL. General practitioners' perspectives on a proposed new model of service delivery for primary care management of knee osteoarthritis: a qualitative study. BMC Fam Pract 2017; 18: 85.

For peer review only - http://bmjopen.bmj.com/site/about/guidelines.xhtml

33. Egerton T, Nelligan RK, Setchell J, Atkins L, Bennell KL. General practitioners' views on managing knee osteoarthritis: a thematic analysis of factors influencing clinical practice guideline implementation in primary care. BMC Rheumatol 2018; 2: 30.

34. Hinman RS, Delany CM, Campbell PK, Gale J, Bennell KL. Physical Therapists, Telephone Coaches, and Patients With Knee Osteoarthritis: Qualitative Study About Working Together to Promote Exercise Adherence. Phys Ther 2016; 96: 479-93.

35. Hinman RS, Nelligan RK, Bennell KL, Delany C. "Sounds a Bit Crazy, But It Was Almost More Personal:" A Qualitative Study of Patient and Clinician Experiences of Physical Therapist-Prescribed Exercise For Knee Osteoarthritis Via Skype. Arthritis Care Res (Hoboken) 2017; 69: 1834-44.

36. Knoop J, van der Leeden M, van der Esch M, de Rooij M, Peter WF, Bennell KL, et al. Is a model of stratified exercise therapy by physical therapists in primary care feasible in patients with knee osteoarthritis? : a mixed methods study. Physiotherapy 2020; 106: 101-10.

37. Law RJ, Nafees S, Hiscock J, Wynne C, Williams NH. A lifestyle management programme focused on exercise, diet and physiotherapy support for patients with hip or knee osteoarthritis and a body mass index over 35: A qualitative study. Musculoskeletal Care 2019; 17: 145-51.

38. Lawford BJ, Delany C, Bennell KL, Hinman RS. "I Was Really Pleasantly Surprised": Firsthand Experience and Shifts in Physical Therapist Perceptions of Telephone-Delivered Exercise Therapy for Knee Osteoarthritis-A Qualitative Study. Arthritis Care Res (Hoboken) 2019; 71: 545-57.

39. Lawford BJ, Bennell KL, Allison K, Schwartz S, Hinman RS. Challenges with strengthening exercises for people with knee osteoarthritis and comorbid obesity: a

For peer review only

qualitative study with patients and physiotherapists. Arthritis Care Res (Hoboken). 2020. DOI:10.1002/acr.24439

40. Lawford BJ, Bennell KL, Jones SE, Keating C, Brown C, Hinman RS. "It's the single best thing I've done in the last 10 years": a qualitative study exploring patient and dietitian experiences with, and perceptions of, a multi-component dietary weight loss program for knee osteoarthritis. Osteoarthritis Cartilage 2021; 29: 507-17.

41. MacKay C, Hawker GA, Jaglal SB. Qualitative study exploring the factors influencing physical therapy management of early knee osteoarthritis in Canada. BMJ Open 2018; 8: e023457.

42. MacKay C, Hawker GA, Jaglal SB. How Do Physical Therapists Approach Management of People With Early Knee Osteoarthritis? A Qualitative Study. Phys Ther 2020; 100: 295-306.

43. Mann C, Gooberman-Hill R. Health care provision for osteoarthritis: concordance between what patients would like and what health professionals think they should have. Arthritis Care Res (Hoboken) 2011; 63: 963-72.

44. Miller KA, Osman F, Baier Manwell L. Patient and physician perceptions of knee and hip osteoarthritis care: A qualitative study. Int J Clin Pract 2020; 74: e13627.

45. Nielsen M, Keefe FJ, Bennell K, Jull GA. Physical therapist-delivered cognitive-behavioral therapy: a qualitative study of physical therapists' perceptions and experiences. Phys Ther 2014; 94: 197-209.

46. Okwera A, May S. Views of general practitioners toward physiotherapy management of osteoarthritis-a qualitative study. Physiother Theory Pract 2019; 35: 940-6.

47. Poitras S, Rossignol M, Avouac J, Avouac B, Cedraschi C, Nordin M, et al. Management recommendations for knee osteoarthritis: how usable are they? Joint Bone Spine 2010; 77: 458-65.

For peer review only - http://bmjopen.bmj.com/site/about/guidelines.xhtml

48. Rosemann T, Wensing M, Joest K, Backenstrass M, Mahler C, Szecsenyi J. Problems and needs for improving primary care of osteoarthritis patients: the views of patients, general practitioners and practice nurses. BMC Musculoskel Dis 2006; 7: 48.

49. Selten EMH, Vriezekolk JE, Nijhof MW, Schers HJ, van der Meulen-Dilling RG, van der Laan WH, et al. Barriers Impeding the Use of Non-pharmacological, Non-surgical Care in Hip and Knee Osteoarthritis: The Views of General Practitioners, Physical Therapists, and Medical Specialists. J Clin Rheumatol 2017; 23: 405-10.

50. Tang CY, Pile R, Croft A, Watson NJ. Exploring Physical Therapist Adherence to Clinical Guidelines When Treating Patients With Knee Osteoarthritis in Australia: A Mixed Methods Study. Phys Ther 2020; 100: 1084-93.

51. Teo PL, Bennell KL, Lawford BJ, Egerton T, Dziedzic KS, Hinman RS. Physiotherapists may improve management of knee osteoarthritis through greater psychosocial focus, being proactive with advice, and offering longer-term reviews: a qualitative study. J Physiother 2020; 66: 256-65.

52. Wallis JA, Ackerman IN, Brusco NK, Kemp JL, Sherwood J, Young K, et al. Barriers and enablers to uptake of a contemporary guideline-based management program for hip and knee osteoarthritis: A qualitative study. Osteoarthritis and Cartilage Open 2020; 2.

53. Cottrell E, Roddy E, Rathod T, Porcheret M, Foster NE. What influences general practitioners' use of exercise for patients with chronic knee pain? Results from a national survey. BMC Fam Pract 2016; 17: 172.

54. Duarte N, Hughes SL, Paúl C. Cultural adaptation and specifics of the Fit & Strong! program in Portugal. Transl Behav Med 2019; 9: 67-75.

For peer review only - http://bmjopen.bmj.com/site/about/guidelines.xhtml

55. Hill DS, Freudmann M, Sergeant JC, Board T. Management of symptomatic knee osteoarthritis in obesity: a survey of orthopaedic surgeons' opinions and practice. Eur J Orthop Surg Traumatol 2018; 28: 967-74.

56. Hill D, Boyd A, Board T. Management of symptomatic knee osteoarthritis in obesity: a survey of general practitioners' opinions and practice. Eur J Orthop Surg Traumatol 2018; 28: 1183-9.

57. Hofstede SN, Marang-van de Mheen PJ, Vliet Vlieland TP, van den Ende CH, Nelissen RG, van Bodegom-Vos L. Barriers and Facilitators Associated with Non-Surgical Treatment Use for Osteoarthritis Patients in Orthopaedic Practice. PLoS One 2016; 11: e0147406.

58. Lawford BJ, Bennell KL, Kasza J, Hinman RS. Physical Therapists' Perceptions of Telephone- and Internet Video-Mediated Service Models for Exercise Management of People With Osteoarthritis. Arthritis Care Res (Hoboken) 2018; 70: 398-408.

59. Reid DA, Potts G, Burnett M, Konings B. Physiotherapy management of knee and hip osteoarthritis: a survey of patient and medicai practitioners' expectations, experiences and perceptions of effectiveness of treatment. New Zeal J Physiother 2014; 42: 118-25.

60. de Rooij M, van der Leeden M, van der Esch M, Lems WF, Meesters JJL, Peter WF, et al. Evaluation of an educational course for primary care physiotherapists on comorbidity-adapted exercise therapy in knee osteoarthritis: an observational study. Musculoskeletal Care 2020; 18: 122-33.

61. Holden MA, Nicholls EE, Young J, Hay EM, Foster NE. UK-based physical therapists' attitudes and beliefs regarding exercise and knee osteoarthritis: findings from a mixed-methods study. Arthritis Rheum 2009; 61: 1511-21.

For peer review only - http://bmjopen.bmj.com/site/about/guidelines.xhtml

62. Kloek CJJ, Bossen D, de Vries HJ, de Bakker DH, Veenhof C, Dekker J. Physiotherapists' experiences with a blended osteoarthritis intervention: a mixed methods study. Physiother Theory Pract 2020; 36: 572-9.

63. Nissen N, Holm PM, Bricca A, Dideriksen M, Tang LH, Skou ST. Clinicians' beliefs and attitudes to physical activity and exercise therapy as treatment for knee and/or hip osteoarthritis: a scoping review. Osteoarthritis Cartilage (online ahead of print). DOI:10.1016/j.joca.2021.11.008

64. Slater H, Dear BF, Merolli MA, Li LC, Briggs AM. Use of eHealth technologies to enable the implementation of musculoskeletal Models of Care: Evidence and practice. Best Pract Res Clin Rheumatol 2016; 30: 483-502.

65. Loeb AE, Rao SS, Ficke JR, Morris CD, Riley LH, Levin AS. Departmental Experience and Lessons Learned With Accelerated Introduction of Telemedicine During the COVID-19 Crisis. J Am Acad Orthop Surg 2020; 28: e469-76.

66. Piga M, Cangemi I, Mathieu A, Cauli A. Telemedicine for patients with rheumatic diseases: Systematic review and proposal for research agenda. Semin Arthritis Rheum 2017; 47: 121-8.

For peer review only - http://bmjopen.bmj.com/site/about/guidelines.xhtml

702 **Table 1.** Overview of included studies[a]

Reference	Country and health setting	Study focus	Type of data extracted	Data collection method	Data analysis method	Participants
Allison[27] (2019)	Australia (private primary care and public hospital care or community health)	Attitudes and perceptions toward role in weight management (knee OA)	Qualitative	Individual interviews	Inductive thematic analysis	PT (n=13, 61% female, age range 27-61 years)
Bossen[28] (2016)	The Netherlands (private practice)	Development and feasibility of the blended exercise therapy intervention "e-Exercise" (hip and/or knee OA)	Qualitative	1) Focus group<br>2) Individual interviews	1) Summarizing<br>2) Thematic trend analysis	1) PT (n=7)<br>2) PT (n=5)
Christiansen[29] (2020)	Canada (academic and community family health practice)	Experiences with and barriers to prescribing exercise (knee OA)	Qualitative	Individual interviews	Constant comparison approach	Physician (n=11)
Davis[30] (2018)	Canada (single assessment center)	Implementation of the "Good Life with osteoArthritis in Denmark (GLA:D^{TM}) Canada" program (hip and/or knee OA)	Qualitative	Individual interviews	Thematic content analysis	PT (n=3)
De Rooij[31] (2014)	The Netherlands (rehabilitation center)	Development of comorbidity-adapted exercise protocols (knee OA)	Qualitative	Individual interviews	Analyzing notes	PT (n=3)
Egerton[32] (2017)[b]	Australia (primary care)	Perspectives on potential barriers and facilitators to engagement with a proposed model of service delivery for primary care management (knee OA)	Qualitative	Individual interviews	Interpretive thematic analysis	GP (n=11, 64% female, mean age 50.8 years (range: 34-67))
Egerton[33] (2018)[b]	Australia (primary care)	Barriers and facilitators influencing clinical practice guideline implementation in	Qualitative	Individual interviews	Interpretive thematic analysis	GP (n=11, 64% female)

For peer review only - http://bmjopen.bmj.com/site/about/guidelines.xhtml

		primary care (knee OA)				
Hinman[34] (2016)	Australia (private practice)	Experiences of being involved in delivering an integrated program of PT-supervised exercise and telephone coaching (knee OA)	Qualitative	Individual interviews	Thematic analysis informed by grounded theory	PT (n=10, 50% female, mean age 43 years (SD: 13))<br><br>Telephone coach (n=4; 100% female, mean age 42 years (SD: 11))
Hinman[35] (2017)	Australia (not specified)	Experiences using Skype as a service delivery model for PT-prescribed exercise management (knee OA)	Qualitative	Individual interviews	Thematic and constant comparative analytical approach	PT (n=8, 50% female, mean age 39 years (SD: 9))
Knoop[36] (2020)	The Netherlands (primary care)	Feasibility of a newly developed model of stratified exercise therapy in primary care (knee OA)	Qualitative	1) Individual interviews<br><br>2) Focus group	Analyzed descriptively	1) PT (n=9)<br><br>2) PT (n=14)
Law[37] (2019)	United Kingdom (leisure center)	Experiences and views of referring and delivering professionals regarding the "Lifestyle Management Program" (hip and/or knee OA)	Qualitative	1) Focus groups<br><br>2) Individual interviews	Framework analysis method	1) Dietician (n=2)<br><br>Exercise professional (n=3)<br><br>PT (n=4)<br><br>Triaging clinician (n=1)<br><br>2) GP (n=3)<br><br>Total group: 46% female

For peer review only - http://bmjopen.bmj.com/site/about/guidelines.xhtml

Lawford[38] (2019)	Australia (private and public practice)	Pre- and post-intervention perceptions of telephone-delivered exercise therapy (knee OA)	Qualitative	Individual interviews	Thematic analysis approach	PT (n=8, 50% female)
Lawford[39] (2020)	Australia (private and public practice)	Experiences and perceptions with prescribing a strengthening exercise program for people with comorbid obesity (knee OA)	Qualitative	Individual interviews	Inductive thematic approach	PT (n=7, 14% female)
Lawford[40] (2021)	Australia (private and public practice)	Experiences with a multi-component dietary weight loss program (knee OA)	Qualitative	Individual interviews	Thematic approach informed by grounded theory	Dietician (n=5, 100% female)
MacKay[41] (2018)[c]	Canada (community-based and outpatient setting)	Factors influencing physical therapy management (knee OA)	Qualitative	Individual interviews	Inductive thematic analysis	PT (n=33, 76% female)
MacKay[42] (2020)[c]	Canada (community-based and outpatient setting)	Perceptions related to physical therapy management (knee OA)	Qualitative	Individual interviews	Inductive thematic analysis	PT (n=33, 76% female)
Mann[43] (2011)	United Kingdom (primary and secondary care)	Perceptions of current service provision and possible service improvements (hip and/or knee OA)	Qualitative	Individual interviews	Framework method	GP (n=2)<br>Nurse practitioner (n=1)<br>Occupational therapist (n=1)<br>OS (n=2)<br>Practice nurse (n=3)<br>PT (n=2)

For peer review only - http://bmjopen.bmj.com/site/about/guidelines.xhtml

						RH (n=1)
Miller[44] (2020)	United States of America (large academic medical center)	Barriers and facilitators to guideline-based treatment (hip and/or knee OA)	Qualitative	Individual interviews	Conventional content analysis	Physician (n=6, 50% female)
Nielsen[45] (2014)	Australia (not specified)	Perspectives on and experiences with an intervention of exercise combined with cognitive behavioral therapy (Pain Coping Skills Training) and the implementation process (knee OA)	Qualitative	Individual interviews	Framework analysis	PT (n=8, 88% female, age range 35-58 years)
Okwera[46] (2019)	United Kingdom (general practice within NHS)	Beliefs on physiotherapy management in primary care (hip and/or knee OA)	Qualitative	Individual interviews	Framework analysis	GP (n=8, 50% female, age range 31-60 years)
Poitras[47] (2010)	France (general practice; work setting PTs not specified)	Barriers to use of conservative management recommendations (knee OA)	Qualitative	Focus groups	Thematic content analysis	GP (n=7, 29% female, median age 53 years (range: 48-77))<br><br>PT (n=10, 40% female, median age 46.5 years (range: 24-69))
Rosemann[48] (2006)	Germany (general practice)	Problems and needs for improving primary care (hip and/or knee OA)	Qualitative	Individual interviews	Description of coding process, but no specific method reported	GP (n=20, 20% female, mean age 43.5 years (range: 33-57))<br><br>Practice nurse (n=20, 100% female, mean age 41.3 years (range: 29-56))
Selten[49] (2017)	The Netherlands	Views on non-pharmacological, non-	Qualitative	Individual	Thematic analysis	GP (n=5)

For peer review only - http://bmjopen.bmj.com/site/about/guidelines.xhtml

	(general practice; work setting PTs, OSs and RHs not specified)	surgical management (hip and/or knee OA)		interviews		OS (n=7)<br>PT (n=7)<br>RH (n=5)<br>Total group: 50% female, age range 24-64 years
Tang[50] (2020)	Australia (large metropolitan public health service)	Application of clinical practice guidelines (knee OA)	Qualitative	Individual interviews	Thematic analysis	PT (n=18)
Teo[51] (2020)	Australia (private practice and tertiary or non-tertiary hospitals)	Experiences with delivering care (knee OA)	Qualitative	Individual interviews	Inductive thematic approach	PT (n=22, 50% female, mean age 34 years (SD: 8, range: 24-54))
Wallis[52] (2020)	Australia (general practice; OSs and RHs working in private and public hospitals)	Perceptions about management including barriers and enablers for referral to the "Good Life with osteoArthritis in Denmark (GLA:D®) Australia" program (hip and/or knee OA)	Qualitative	Individual interviews	Inductive thematic analysis	GP (n=5)<br>OS (n=6)<br>RH (n=4)<br>Total group: mean age 52 years (SD: 12)
Cottrell[53] (2016)	United Kingdom (general practice)	Attitudes and beliefs regarding exercise (knee OA)	Quantitative	Survey (RR: 17%)	Descriptive statistics (frequency)	GP (n=835, 51% female)
Duarte[54] (2019)	Portugal (not specified)	Development and acceptability of the Portuguese version of the "Fit & Strong!" program (hip and/or knee OA)	Quantitative	Survey (RR: 100%)	Not reported	Program instructor (n=2)
Hill[55] (2018)	United Kingdom	Opinions and practices regarding the	Quantitative	Survey (RR:	Descriptive statistics	OS (n=205)

For peer review only - http://bmjopen.bmj.com/site/about/guidelines.xhtml

	(specialist practice in knee surgery)	management of symptomatic OA in obesity (knee OA)		52%)	(frequency)	
Hill[56] (2018)	United Kingdom (general practice)	Opinions and practices regarding the management of symptomatic OA in obesity (knee OA)	Quantitative	Survey (RR: 75%)	Descriptive statistics (frequency)	GP (n=130)
Hofstede[57] (2016)	The Netherlands (52% of OSs worked at a general hospital)	Barriers and facilitators associated with prescription of different non-surgical treatments (hip and/or knee OA)	Quantitative	Survey (RR: 36%)	Descriptive statistics (frequency)	OS (n=172, 9% female, mean age 48.4 years (SD: 8.6))
Lawford[58] (2018)	Australia (private and public practice)	Perceptions of remotely-delivered service models for exercise management (hip and/or knee OA)	Quantitative	Survey (RR: unknown)	Descriptive statistics (frequency and level of agreement)	PT (n=217, 72% female)
Reid[59] (2014)	New Zealand (general practice; work setting OSs not specified)	Self-reported behavior, experiences, expectations and perceptions regarding physiotherapy referral and management (hip and/or knee OA)	Quantitative	Survey (RR: 46% (GP) and 26% (OS))	Descriptive statistics (frequency)	GP (n=24)<br><br>OS (n=20)<br><br>Total group: 34% female, mean age 52.2 years (SD: 8.5)
De Rooij[60] (2020)	The Netherlands (primary care)	Facilitators and barriers for usage of a strategy for exercise prescription in patients with comorbidity (knee OA)	Mixed-methods	1) Survey (RR: 100%)<br><br>2) Individual interviews	1) Descriptive statistics (frequency)<br><br>2) Summarizing notes	1) PT (n=34, 68% female, mean age 43.7 years (SD: 11.1))<br><br>2) PT (n=10)
Holden[61] (2009)	United Kingdom (NHS and non-NHS)	Attitudes and beliefs regarding exercise (knee OA)	Mixed-methods	1) Survey (RR: 58%)<br><br>2) Individual interviews	1) Descriptive statistics (level of agreement)<br><br>2) Thematic analysis	1) PT (n=538, 87% female)<br><br>2) PT (n=24, 67% female)

For peer review only - http://bmjopen.bmj.com/site/about/guidelines.xhtml

Kloek[62] (2020)	The Netherlands (primary care practice)	Experiences with and determinants related to the usage of the blended physiotherapy intervention "e-Exercise" (hip and/or knee OA)	Mixed-methods	1) Survey (RR: 40%)<br><br>2) Individual interviews	1) Descriptive statistics (frequency)<br>2) Grounded theory methodology	1) PT (n=49)<br><br>2) PT (n=9, 33% female, median age 52 years (range: 24-59))

[a] GP: general practitioner; NHS: National Health Service; OA: osteoarthritis; OS: orthopedic surgeon; PT: physiotherapist; RH: rheumatologist; RR: response rate; SD: standard deviation.
[b,c] Data for both studies were collected during the same interview.

For peer review only

For peer review only - http://bmjopen.bmj.com/site/about/guidelines.xhtml

Table 2. Distribution of the extracted factors per included article across the domains, which were largely based on the Tailored Implementation of Chronic Diseases checklist[a]

Reference	Domain 1: Intervention factors	Domain 2: Individual HCP factors	Domain 3: Patient factors	Domain 4: Professional interactions	Domain 5: Incentives and resources	Domain 6: Capacity for organizational change	Domain 7: Social, political, and legal factors	Domain 8: Patient and HCP interactions	Domain 9: Disease factors	Total number of factors in article
Allison[27] (2019)		3		2	2	1		4		12
Bossen[28] (2016)	8									8
Christiansen[29] (2020)	1	5	2						1	9
Davis[30] (2018)	6		1							7
De Rooij[31] (2014)	3	2								5
Egerton[32] (2017)	20	3	1	9	3				1	37
Egerton[33] (2018)	5	9	5		6		1	1	5	32
Hinman[34] (2016)	7	1	2	10						20
Hinman[35]	18									18

For peer review only

(2017)										
Knoop[36] (2020)	4		1	1						6
Law[37] (2019)	8	1	5	1	2			1		18
Lawford[38] (2019)	26									26
Lawford[39] (2020)	11		7					1		19
Lawford[40] (2021)	12		3							15
MacKay[41] (2018)	6	5	14	7	6	2	1			41
MacKay[42] (2020)	4	12	5	1	1			4		27
Mann[43] (2011)	2	1	4	10	1				1	19
Miller[44] (2020)	4	4	7	3	8		1	1	1	29
Nielsen[45] (2014)	13	8	1		3	2				27
Okwera[46] (2019)	4	6	6	12			2		2	32
Poitras[47]	11	13	19	3				1	5	52

For peer review only

(2010)										
Rosemann[48] (2006)	1	4	5	4	6		1	1	1	23
Selten[49] (2017)	7	3	3	14	2			4		33
Tang[50] (2020)		12	4					1		17
Teo[51] (2020)	3	11	8						1	23
Wallis[52] (2020)	17		7	3	2				1	30
Cottrell[53] (2016)	12	10	4	2	3					31
Duarte[54] (2019)	1		2							3
Hill[55] (2018)		5		2						7
Hill[56] (2018)	2	4		2						8
Hofstede[57] (2016)	5	3		4	1	1				14
Lawford[58] (2018)	33									33
Reid[59] (2014)	4	1	3	1						9
De Rooij[60] (2020)	18	8	4	9	2	1	3			45

For peer review only

Holden[61] (2009)	13	10	14		3				2	42
Kloek[62] (2020)	26			1	5					32
**Total number of factors in domain**	**315**	**144**	**137**	**101**	**56**	**7**	**9**	**19**	**21**	**809**

[a] HCP: healthcare professional.

For peer review only

Table 3. Overview of barriers, facilitators, and unclear factors that influence the implementation of LIs as perceived by HCPs for all domains, which were largely based on the Tailored Implementation of Chronic Diseases checklist[a]

**Category**	**Subcategory – Barriers**	**Subcategory – Facilitators**	**Subcategory – Unclear factors**
Domain 1: Intervention factors (factors related to LIs)			
Effectiveness	- LIs have little or no effect on OA[29,32,33,44,46,47,49,53,59,61]<br><br>- Potential effects of LIs are difficult to accomplish[47,48,53,61]	- LIs have positive effects on affected joint(s)[35,38,40-42,47,49,52,53,58,61]<br><br>- LIs have positive effects on general health[33,40,47,49,56,57]<br><br>- LIs have positive mental effects[30,35,37,38,40,49,52,57]<br><br>- LIs have positive effects (not further specified)[34,37,44,49,52,54,57]	
Safety	- LIs are unsafe or have negative effects[39,47,52,61]	- LIs are safe[53,57]<br><br>- Research environment or protocols provide a safety net[31,35,38,39]	
Design	- Non-optimal content or structure of LIs[34,36,52,53,62]<br><br>- Challenges for patients during participation in LIs[39,40,45]<br><br>- Challenges for HCPs during delivery of LIs[28,30,39,60,62]	- Positive experiences with or suggestions to improve the content or structure of LIs[28,30,34,37,40,45,52,60,62]<br><br>- Ease for patients during participation in LIs[39,40,52]<br><br>- Ease for HCPs during delivery of LIs[30,31,34,39,45,60,62]	
Personalized treatment	- Insufficient ability to provide personalized treatment within LIs[32,45,62]	- Ability and importance of providing personalized treatment within LIs[37,39,42,45,47,51,53,60-62]	
Accessibility	- LIs are unavailable or	- LIs are available or accessible, or suggestions for	

For peer review only - http://bmjopen.bmj.com/site/about/guidelines.xhtml

	inaccessible[28,33,41,43,44,53,56,59,61]<br><br>- Costs of LIs to patients[32,33,41,44,51,52]<br><br>- LIs are not feasible or sustainable[32,60]<br><br>- Inconvenience to patients when accessing LIs[51-53]	improvement[32,37,41,46,57,59]<br><br>- LIs are feasible or sustainable[32,36,37,42,60]<br><br>- Convenience for patients when accessing LIs[52]	
Telehealth	- Disadvantages of telehealth in terms of effectiveness[32,58,62]<br><br>- Telehealth is not safe for patients or patient/data privacy[32,58]<br><br>- Challenges for HCPs regarding lack of physical/visual contact[35,38,58,62]<br><br>- Other challenges for HCPs regarding feasibility of telehealth[28,32,35,38,58,62]<br><br>- Patient-related challenges regarding feasibility of telehealth[28,32,62]<br><br>- Negative aspects regarding communication and relationship using telehealth[34,35,38,40]	- Benefits of telehealth in terms of effectiveness[28,35,38,58,62]<br><br>- Telehealth is safe for patients or patient/data privacy[35,58,62]<br><br>- Lack of physical/visual contact not a major issue for HCPs[35,38,58]<br><br>- Positive attitude or needs of HCPs regarding feasibility of telehealth[35,38,40,58,62]<br><br>- Patient-related benefits regarding feasibility of telehealth[28,32,35,38,58]<br><br>- Positive aspects regarding communication and relationship using telehealth[38,40]	
Domain 2: Individual HCP factors (factors related to individual primary and secondary HCPs)			
Expertise	- Lack of knowledge or skills around LIs or promoting behavioral change[27,29,33,41,42,45,47,49-51,56,60,61]<br><br>- Lack of knowledge or skills around OA care in general[43,44,46,48]<br><br>- Lack of knowledge or skills around specific	- Having or improving knowledge or skills around LIs or promoting behavioral change[33,34,41,42,45,46,50]<br><br>- Having or improving knowledge or skills around OA care in general[33,44,46,48]<br><br>- Available resources might improve knowledge and decision-making[31,50,60]	- Clinical experience[42]

For peer review only - http://bmjopen.bmj.com/site/about/guidelines.xhtml

	resources[33,50,60]		
Attitude	- Negative attitude toward LIs[29,53,61]<br><br>- Negative attitude toward guidelines or protocols[46]	- Positive attitude toward LIs[33,41,42,45-47,50,51,53,55-57,59]<br><br>- Positive attitude toward guidelines or protocols[27,57,60]	- Autonomy[37]
Role	- Perception of own role potentially impeding prescription or follow-up of LIs[29,33,42,44,47-51,53,55,61]<br><br>- Negative consequences for own role when referring patients to LIs[32]	- Perception of own role potentially stimulating prescription or follow-up of LIs[33,41,42,47,48,51,53,55,56,61]<br><br>- Positive consequences for own role when referring patients to LIs[32]	
Domain 3: Patient factors (factors related to patients with hip and/or knee OA)			
Health status	- Severity of disease and symptoms[32,44,47,50,52,61]<br><br>- Negative impact of comorbidities[29,39,44,47,48,51,52]<br><br>- Other patient characteristics[47,52,59]	- Severity of disease and symptoms[39,47,53,59,61]<br><br>- Other patient characteristics[41,51,59]	- Severity of disease and symptoms[42,46,53,61]<br><br>- Other patient characteristics[41]
Treatment expectations and preferences	- Negative attitude toward LIs[29,33,34,36,39,41-48,51-53,60,61]<br><br>- Positive attitude toward TJA[37,43,48]	- Make use of patients' preference for TJA within LIs[37]	- Patients' preferences[46]
Active participation	- Low patient adherence or engagement[33,37,41,42,46,47,51,54,61]	- High patient adherence or engagement[34,39,40,54]<br><br>- Importance of high patient adherence or engagement for effectiveness of LIs[30,41,42,47,53,61]	
Capabilities	- Low health literacy[33,37,39-41,43,44,47,49,51,52,60,61]<br><br>- Limited financial resources[41,44]<br><br>- Other responsibilities[41,52]	- High health literacy or importance of education[39,42,43,49,51,60]<br><br>- Social support[40,48]	- Health literacy[46]<br><br>- Other responsibilities[41]
Domain 4: Professional interactions (factors related to interactions between primary and secondary HCPs)			

For peer review only

Collaboration	- Non-optimal interdisciplinary collaboration or healthcare provision[27,32,34,41,43,46,47,49,53,60]<br><br>- No access to other HCPs[41]	- Good interdisciplinary collaboration or healthcare provision, or suggestions for improvement[27,32,34,37,41,43,44,46-49,52,53,55-57,59,60,62]<br><br>- Access to other HCPs[32,41-43,46]
Communication and referral	- Lack of communication between HCPs[46,48,60]<br><br>- Challenges of communication and referral procedures[34,36,44,46,60]	- Improving communication between HCPs[32,34,46,48,52,57]<br><br>- Needs regarding communication and referral procedures[32,41,46,49,52]
Domain 5: Incentives and resources (factors related to the availability of incentives and resources for primary and secondary HCPs)		
Time	- Lack of time within patient consultations[33,41,43-45,49,53,61]<br><br>- Lack of time due to other demands (or not further specified)[32,37,41,48,62]	- Adequate duration of patient consultations[33,41]<br><br>- Adequate duration of specific interventions or protocols[32,45,60,62]
Financial resources	- Limited financial resources within organization[45,48]	- Financial reward for implementing LIs[32,48,60]
Information resources	- Lack of information resources[27,37,44,48]<br><br>- Challenges in accessing information resources[41,44,53]	- Availability of information resources[27,44,52,57]<br><br>- Access to information resources[33,41,42,52]
Facilities	- Negative attitude toward information technology[33]	- Potential use of information technology[33,44]<br><br>- Benefits of working in health centers[49]
Domain 6: Capacity for organizational change (factors related to the organization where primary and secondary HCPs work)		
Professional paradigm		- Adequate professional paradigm or suggestions for expansion[27,41,45]
Monitoring		- Audit[57]

For peer review only - http://bmjopen.bmj.com/site/about/guidelines.xhtml

Support within the organization	- Management not supportive[60]	
Domain 7: Social, political, and legal factors (factors related to the social, political, and legal context)		
Healthcare system	- Restrictions due to health insurance[41,48,60]	- Benefits of good health insurance[44,46,60]<br>- Government subsidies[33]
Domain 8: Patient and HCP interactions (factors related to interactions between patients with hip and/or knee OA and primary and secondary HCPs)		
Therapeutic alliance	- Potential negative influence of implementing LIs to relationship[37]	- Importance of communication and relationship[39,42,48,49]
Lifestyle as conversation topic	- Challenges of discussing weight[27,33,42,49,50]	- Factors that could ease the way to discussing weight[27,42,44,47,49]
Domain 9: Disease factors (factors related to OA)		
Image	- OA seen as low priority[29,32,43,46-48]<br>- OA seen as untreatable and local condition (wear-and-tear)[33,44,46,47,51,52,61]	- Optimistic views toward OA[33,47]

[a] HCP: healthcare professional; LI: lifestyle intervention; OA: osteoarthritis; TJA: total joint arthroplasty.

For peer review only

712 **Table 4.** Ranking of the ten largest subcategories of barriers[a]

Rank	Subcategory of barriers *(domain)*	Factors (n)
1	Non-optimal interdisciplinary collaboration or healthcare provision *(4 – professional interactions)*	31
2	Negative attitude toward LIs *(3 – patient factors)*	28
3	Low health literacy *(3 – patient factors)*	24
	Lack of knowledge or skills around LIs or promoting behavioral change *(2 – individual HCP factors)*	24
5	Perception of own role potentially impeding prescription or follow-up of LIs *(2 – individual HCP factors)*	23
6	Severity of disease and symptoms *(3 – patient factors)*	17
7	Other challenges for HCPs regarding feasibility of telehealth *(1 – intervention factors)*	16
8	LIs have little or no effect on OA *(1 – intervention factors)*	14
9	Lack of time within patient consultations *(5 – incentives and resources)*	12
	LIs are unavailable or inaccessible *(1 – intervention factors)*	12

713 [a] HCP: healthcare professional; LI: lifestyle intervention; OA: osteoarthritis.

For peer review only - http://bmjopen.bmj.com/site/about/guidelines.xhtml

714 **Table 5.** Ranking of the ten largest subcategories of facilitators[a]

Rank	Subcategory of facilitators ***(domain)***	Factors (n)
1	Good interdisciplinary collaboration or healthcare provision, or suggestions for improvement *(4 – professional interactions)*	40
2	Perception of own role potentially stimulating prescription or follow-up of LIs *(2 – individual HCP factors)*	27
3	Positive experiences with or suggestions to improve the content or structure of LIs *(1 – intervention factors)*	24
4	Positive attitude toward LIs *(2 – individual HCP factors)*	22
5	Positive attitude or needs of HCPs regarding feasibility of telehealth *(1 – intervention factors)*	18
	Ease for HCPs during delivery of LIs *(1 – intervention factors)*	18
7	LIs have positive effects on affected joint(s) *(1 – intervention factors)*	17
8	Patient-related benefits regarding feasibility of telehealth *(1 – intervention factors)*	16
9	Ability and importance of providing personalized treatment within LIs *(1 – intervention factors)*	15
10	Having or improving knowledge or skills around LIs or promoting behavioral change *(2 – individual HCP factors)*	14

715 [a] HCP: healthcare professional; LI: lifestyle intervention.

For peer review only

For peer review only - http://bmjopen.bmj.com/site/about/guidelines.xhtml

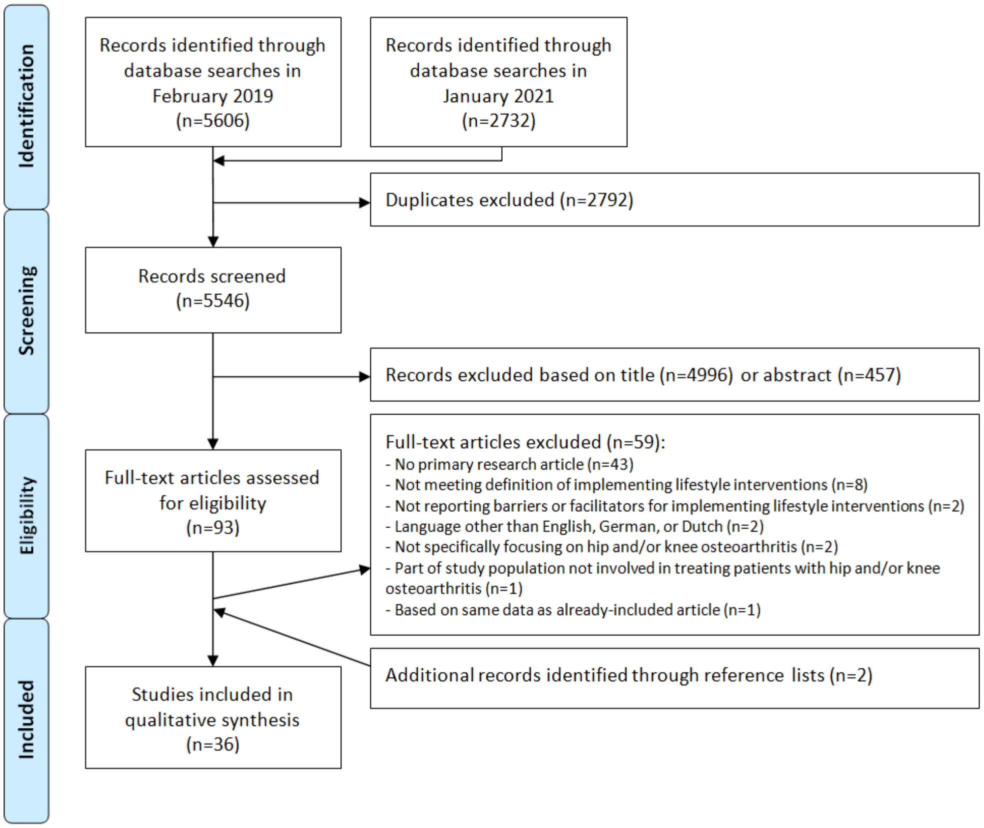

Figure 1. Flowchart of the study selection process

260x217mm (96 x 96 DPI)

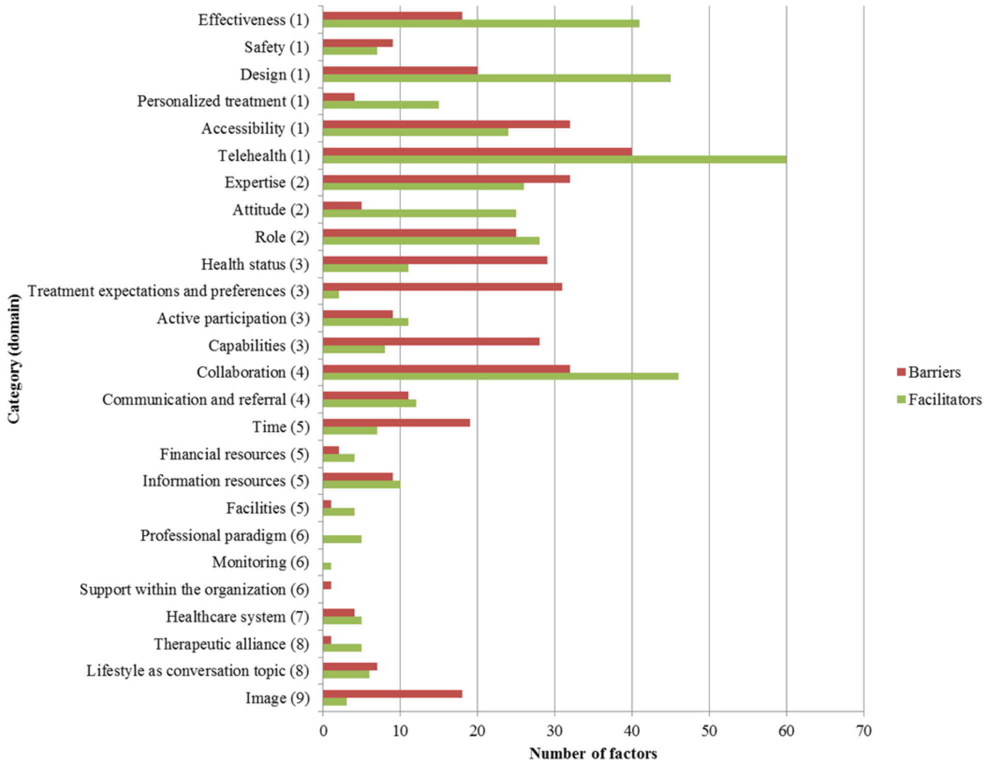

Figure 2. Overview of the number of barriers and facilitators per category
The domain numbers indicated in brackets refer to the domains as presented in Table 3: (1) intervention factors; (2) individual HCP factors; (3) patient factors; (4) professional interactions; (5) incentives and resources; (6) capacity for organizational change; (7) social, political, and legal factors; (8) patient and HCP interactions; (9) disease factors. Unclear factors were not included in this figure due to the low number (n=11).

282x219mm (96 x 96 DPI)

Supplemental File 1. Search strategies for the different databases

The search strategies presented below were applied in five bibliographic electronic databases on two different dates: initially on 19 February 2019 and again on 19 January 2021 to update the results. For each database it is shown below which search strategy was used in 2019 (#1) and in 2021 (#2) and how many records were retrieved using these search strategies.

PubMed

#1<br>(1798 hits)	(((((("Health Personnel"[Mesh] OR "Delivery of Health Care"[Mesh] OR "Primary Health Care"[Mesh]) OR (Health personnel*[tiab] OR Health professional*[tiab] OR Medical staff*[tiab] OR Clinician*[tiab] OR Medical specialist*[tiab] OR Health care*[tiab] OR Healthcare*[tiab] OR Primary care*[tiab] OR Secondary care*[tiab] OR Allied health personnel*[tiab] OR Allied health professional*[tiab] OR Paramedic*[tiab] OR Nurse*[tiab] OR Nursing staff*[tiab] OR Nurse practit*[tiab] OR Physician assistant*[tiab] OR Physicians assistant*[tiab] OR Nutritionist*[tiab] OR Dietician*[tiab] OR Dietitian*[tiab] OR Physical therapist*[tiab] OR Physiotherapist*[tiab] OR Lifestyle coach*[tiab] OR Lifestyle counselor*[tiab] OR Physician*[tiab] OR Orthopedic surgeon*[tiab] OR Orthopedist*[tiab] OR General practitioner*[tiab] OR General practice*[tiab] OR Psychologist*[tiab])))<br>**AND**<br>((("Osteoarthritis"[Mesh]) OR (Osteoarthrit*[tiab] OR Osteoarthro*[tiab] OR Degenerative arthrit*[tiab]))) AND ((("Hip Joint"[Mesh] OR "Knee Joint"[Mesh] OR "Lower Extremity"[Mesh]) OR (Hip*[tiab] OR Cox*[tiab] OR Acetabulofemoral joint*[tiab] OR Knee*[tiab] OR Superior tibulofibular joint*[tiab] OR Patellofemoral*[tiab] OR Lower extremit*[tiab] OR Lower limb*[tiab]))))<br>**AND**<br>((((("Life Style"[Mesh] OR "Behavior"[Mesh]) OR (Life style*[tiab] OR Lifestyle*[tiab] OR Behavior*[tiab] OR Behaviour*[tiab] OR Habit*[tiab] OR Risk reduction*[tiab] OR Early therap*[tiab] OR Secondary prevention*[tiab] OR Tertiary prevention*[tiab] OR Disease prevention*[tiab]))) OR ((("Exercise"[Mesh] OR "Exercise Therapy"[Mesh] OR "Sports"[Mesh]) OR (Physical activit*[tiab] OR Physical training*[tiab] OR Physical fitness*[tiab] OR Physical condition*[tiab] OR Physical therap*[tiab] OR Physiotherap*[tiab] OR Exercis*[tiab] OR Run*[tiab] OR Jog*[tiab] OR Walk*[tiab] OR Bicycl*[tiab] OR Swim*[tiab] OR Strength*[tiab] OR Resistance*[tiab] OR Sport*[tiab] OR Athletic*[tiab] OR Train*[tiab] OR Sedentary[tiab]))) OR ((("Diet, Food, and Nutrition"[Mesh] OR "Nutrition Therapy"[Mesh] OR "Body Weight"[Mesh]) OR (Healthy diet*[tiab] OR Healthy eating*[tiab] OR Nutrition*[tiab] OR Diet*[tiab] OR Food*[tiab] OR Weight loss*[tiab] OR Weight loss program*[tiab] OR Weight reduction*[tiab] OR Weight reduction program*[tiab] OR Body weight*[tiab] OR Overweight*[tiab] OR Obesity*[tiab] OR Overnutrition*[tiab] OR Hypernutrition*[tiab])))))<br>**AND**<br>((("Attitude"[Mesh] OR "Motivation"[Mesh]) OR (Barrier*[tiab] OR Facilitator*[tiab] OR Enabler*[tiab] OR Driver*[tiab] OR Motivat*[tiab] OR Opinion*[tiab] OR View*[tiab] OR Attitude*[tiab] OR Expectation*[tiab] OR Incentive*[tiab] OR Disincentive*[tiab] OR Belief*[tiab] OR Influencing factor*[tiab] OR Experience*[tiab] OR Perspective*[tiab] OR Perception*[tiab] OR Hinder*[tiab] OR Impediment*[tiab] OR obstacle*[tiab]))))))
#2<br>(467 hits)	#1 **AND** (("2019/01/01"[Date - Create] : "3000"[Date - Create]) OR ("2019/01/01"[Date - Entry] : "3000"[Date - Entry]) OR ("2019/01/01"[Date - MeSH] : "3000"[Date - MeSH]))

Embase

#1	(‘health care personnel’/exp OR ‘health care delivery’/exp OR (‘Health personnel*’ OR ‘Health professional*’ OR ‘Medical staff*’ OR ‘Clinician*’ OR

For peer review only - http://bmjopen.bmj.com/site/about/guidelines.xhtml

(3036 hits)	'Medical specialist*' OR 'Health care*' OR 'Healthcare*' OR 'Primary care*' OR 'Secondary care*' OR 'Allied health personnel*' OR 'Allied health professional*' OR 'Paramedic*' OR 'Nurse*' OR 'Nursing staff*' OR 'Nurse practit*' OR 'Physician assistant*' OR 'Physicians assistant*' OR 'Nutritionist*' OR 'Dietician*' OR 'Dietitian*' OR 'Physical therapist*' OR 'Physiotherapist*' OR 'Lifestyle coach*' OR 'Lifestyle counselor*' OR 'Physician*' OR 'Orthopedic surgeon*' OR 'Orthopedist*' OR 'General practitioner*' OR 'General practice*' OR 'Psychologist*'):ab,ti)<br>**AND**<br>('osteoarthritis'/exp OR ('Osteoarthrit*' OR 'Osteoarthro*' OR 'Degenerative arthrit*'):ab,ti) AND ('lower limb'/exp OR ('Hip*' OR 'Cox*' OR 'Acetabulofemoral joint*' OR 'Knee*' OR 'Superior tibulofibular joint*' OR 'Patellofemoral*' OR 'Lower extremit*' OR 'Lower limb*'):ab,ti)<br>**AND**<br>(('lifestyle'/exp OR 'lifestyle modification'/exp OR 'behavior'/exp OR ('Life style*' OR 'Lifestyle*' OR 'Behavior*' OR 'Behaviour*' OR 'Habit*' OR 'Risk reduction*' OR 'Early therap*' OR 'Secondary prevention*' OR 'Tertiary prevention*' OR 'Disease prevention*'):ab,ti) OR ('exercise'/exp OR 'kinesiotherapy'/exp OR 'sport'/exp OR ('Physical activit*' OR 'Physical training*' OR 'Physical fitness*' OR 'Physical condition*' OR 'Physical therap*' OR 'Physiotherap*' OR 'Exercis*' OR 'Run*' OR 'Jog*' OR 'Walk*' OR 'Bicycl*' OR 'Swim*' OR 'Strength*' OR 'Resistance*' OR 'Sport*' OR 'Athletic*' OR 'Train*' OR 'Sedentary'):ab,ti) OR ('nutrition'/exp OR 'diet therapy'/exp OR 'body weight'/exp OR 'body weight management'/exp OR ('Healthy diet*' OR 'Healthy eating*' OR 'Nutrition*' OR 'Diet*' OR 'Food*' OR 'Weight loss*' OR 'Weight loss program*' OR 'Weight reduction*' OR 'Weight reduction program*' OR 'Body weight*' OR 'Overweight*' OR 'Obesity*' OR 'Overnutrition*' OR 'Hypernutrition*'):ab,ti))<br>**AND**<br>('attitude'/exp OR 'motivation'/exp OR ('Barrier*' OR 'Facilitator*' OR 'Enabler*' OR 'Driver*' OR 'Motivat*' OR 'Opinion*' OR 'View*' OR 'Attitude*' OR 'Expectation*' OR 'Incentive*' OR 'Disincentive*' OR 'Belief*' OR 'Influencing factor*' OR 'Experience*' OR 'Perspective*' OR 'Perception*' OR 'Hinder*' OR 'Impediment*' OR 'obstacle*'):ab,ti)
#2<br>(1021 hits)	#1 **AND** [1-1-2019]/sd NOT [20-1-2021]/sd

<u>CINAHL</u>

#1<br>(424 hits)	(MH "Health Personnel+" OR MH "Health Care Delivery+" OR TI(Health personnel* OR Health professional* OR Medical staff* OR Clinician* OR Medical specialist* OR Health care* OR Healthcare* OR Primary care* OR Secondary care* OR Allied health personnel* OR Allied health professional* OR Paramedic* OR Nurse* OR Nursing staff* OR Nurse practit* OR Physician assistant* OR Physicians assistant* OR Nutritionist* OR Dietician* OR Dietitian* OR Physical therapist* OR Physiotherapist* OR Lifestyle coach* OR Lifestyle counselor* OR Physician* OR Orthopedic surgeon* OR Orthopedist* OR General practitioner* OR General practice* OR Psychologist*) OR AB(Health personnel* OR Health professional* OR Medical staff* OR Clinician* OR Medical specialist* OR Health care* OR Healthcare* OR Primary care* OR Secondary care* OR Allied health personnel* OR Allied health professional* OR Paramedic* OR Nurse* OR Nursing staff* OR Nurse practit* OR Physician assistant* OR Physicians assistant* OR Nutritionist* OR Dietician* OR Dietitian* OR Physical therapist* OR Physiotherapist* OR Lifestyle coach* OR Lifestyle counselor* OR Physician* OR Orthopedic surgeon* OR Orthopedist* OR General practitioner* OR General practice* OR Psychologist*))<br>**AND**<br>(MH "Osteoarthritis+" OR TI(Osteoarthrit* OR Osteoarthro* OR Degenerative arthrit*) OR AB(Osteoarthrit* OR Osteoarthro* OR Degenerative arthrit*)) AND (MH "Lower Extremity+" OR MH "Hip Joint+" OR MH "Knee Joint+" OR TI(Hip* OR Cox* OR Acetabulofemoral joint* OR Knee* OR Superior tibulofibular joint* OR Patellofemoral* OR Lower extremit* OR Lower limb*) OR AB(Hip* OR Cox* OR Acetabulofemoral joint* OR Knee* OR Superior tibulofibular joint* OR Patellofemoral* OR Lower extremit* OR Lower limb*))

For peer review only - http://bmjopen.bmj.com/site/about/guidelines.xhtml

	**AND**<br>((MH “Life Style+” OR MH “Behavior+” OR TI(Life style* OR Lifestyle* OR Behavior* OR Behaviour* OR Habit* OR Risk reduction* OR Early therap* OR Secondary prevention* OR Tertiary prevention* OR Disease prevention*) OR AB(Life style* OR Lifestyle* OR Behavior* OR Behaviour* OR Habit* OR Risk reduction* OR Early therap* OR Secondary prevention* OR Tertiary prevention* OR Disease prevention*)) OR (MH “Exercise+” OR MH “Therapeutic Exercise+” OR MH “Sports+” OR TI(Physical activit* OR Physical training* OR Physical fitness* OR Physical condition* OR Physical therap* OR Physiotherap* OR Exercis* OR Run* OR Jog* OR Walk* OR Bicycl* OR Swim* OR Strength* OR Resistance* OR Sport* OR Athletic* OR Train* OR Sedentary) OR AB(Physical activit* OR Physical training* OR Physical fitness* OR Physical condition* OR Physical therap* OR Physiotherap* OR Exercis* OR Run* OR Jog* OR Walk* OR Bicycl* OR Swim* OR Strength* OR Resistance* OR Sport* OR Athletic* OR Train* OR Sedentary)) OR (MH “Nutrition+” OR MH “Food+” OR MH “Diet Therapy+” OR MH “Body Weight+” OR TI(Healthy diet* OR Healthy eating* OR Nutrition* OR Diet* OR Food* OR Weight loss* OR Weight loss program* OR Weight reduction* OR Weight reduction program* OR Body weight* OR Overweight* OR Obesity* OR Overnutrition* OR Hypernutrition*) OR AB(Healthy diet* OR Healthy eating* OR Nutrition* OR Diet* OR Food* OR Weight loss* OR Weight loss program* OR Weight reduction* OR Weight reduction program* OR Body weight* OR Overweight* OR Obesity* OR Overnutrition* OR Hypernutrition*)))<br>**AND**<br>(MH “Attitude+” OR MH “Motivation+” OR TI(Barrier* OR Facilitator* OR Enabler* OR Driver* OR Motivat* OR Opinion* OR View* OR Attitude* OR Expectation* OR Incentive* OR Disincentive* OR Belief* OR Influencing factor* OR Experience* OR Perspective* OR Perception* OR Hinder* OR Impediment* OR Obstacle*) OR AB(Barrier* OR Facilitator* OR Enabler* OR Driver* OR Motivat* OR Opinion* OR View* OR Attitude* OR Expectation* OR Incentive* OR Disincentive* OR Belief* OR Influencing factor* OR Experience* OR Perspective* OR Perception* OR Hinder* OR Impediment* OR Obstacle*))
#2<br>(598 hits)	#1

PsycINFO

#1<br>(49 hits)	(DE “Health Personnel” OR DE “Health Care Delivery” OR DE “Primary Health Care” OR DE "Physical Therapists" OR DE "Nurses" OR DE “Physicians” OR DE "Family Physicians" OR DE "General Practitioners" OR DE "Surgeons" OR DE "Clinical Psychologists" OR DE "Allied Health Personnel" OR DE "Caregivers" OR DE "Medical Personnel" OR DE "Mental Health Personnel" OR DE "Clinicians" OR DE "Therapists" OR DE "Psychologists" OR TI(Health personnel* OR Health professional* OR Medical staff* OR Clinician* OR Medical specialist* OR Health care* OR Healthcare* OR Primary care* OR Secondary care* OR Allied health personnel* OR Allied health professional* OR Paramedic* OR Nurse* OR Nursing staff* OR Nurse practit* OR Physician assistant* OR Physicians assistant* OR Nutritionist* OR Dietician* OR Dietitian* OR Physical therapist* OR Physiotherapist* OR Lifestyle coach* OR Lifestyle counselor* OR Physician* OR Orthopedic surgeon* OR Orthopedist* OR General practitioner* OR General practice* OR Psychologist*) OR AB(Health personnel* OR Health professional* OR Medical staff* OR Clinician* OR Medical specialist* OR Health care* OR Healthcare* OR Primary care* OR Secondary care* OR Allied health personnel* OR Allied health professional* OR Paramedic* OR Nurse* OR Nursing staff* OR Nurse practit* OR Physician assistant* OR Physicians assistant* OR Nutritionist* OR Dietician* OR Dietitian* OR Physical therapist* OR Physiotherapist* OR Lifestyle coach* OR Lifestyle counselor* OR Physician* OR Orthopedic surgeon* OR Orthopedist* OR General practitioner* OR General practice* OR Psychologist*))<br>**AND**<br>(TI(Osteoarthrit* OR Osteoarthro* OR Degenerative arthrit*) OR AB(Osteoarthrit* OR Osteoarthro* OR Degenerative arthrit*)) AND (DE "Hips" OR DE “Knee” OR TI(Hip* OR Cox* OR Acetabulofemoral joint* OR Knee* OR Superior tibulofibular joint* OR Patellofemoral* OR Lower extremit* OR Lower limb*) OR AB(Hip* OR Cox* OR Acetabulofemoral joint* OR Knee* OR Superior tibulofibular joint* OR Patellofemoral* OR Lower extremit* OR Lower

	limb*))<br>**AND**<br>((DE "Lifestyle" OR DE "Behavior" OR DE "Active Living" OR DE "Lifestyle Changes" OR DE "Activity Level" OR DE "Habits" OR DE "Behavior Change" OR DE "Readiness to Change" OR DE "Stages of Change" OR DE "Illness Behavior" OR DE "Health Behavior" OR DE "Health Attitudes" OR TI(Life style* OR Lifestyle* OR Behavior* OR Behaviour* OR Habit* OR Risk reduction* OR Early therap* OR Secondary prevention* OR Tertiary prevention* OR Disease prevention*) OR AB(Life style* OR Lifestyle* OR Behavior* OR Behaviour* OR Habit* OR Risk reduction* OR Early therap* OR Secondary prevention* OR Tertiary prevention* OR Disease prevention*)) OR (DE "Exercise" OR DE "Movement Therapy" OR DE "Sports" OR DE "Aerobic Exercise" OR DE "Physical Activity" OR DE "Physical Fitness" OR DE "Rehabilitation" OR DE "Physical Therapy" OR DE "Sedentary Behavior" OR TI(Physical activit* OR Physical training* OR Physical fitness* OR Physical condition* OR Physical therap* OR Physiotherap* OR Exercis* OR Run* OR Jog* OR Walk* OR Bicycl* OR Swim* OR Strength* OR Resistance* OR Sport* OR Athletic* OR Train* OR Sedentary) OR AB(Physical activit* OR Physical training* OR Physical fitness* OR Physical condition* OR Physical therap* OR Physiotherap* OR Exercis* OR Run* OR Jog* OR Walk* OR Bicycl* OR Swim* OR Strength* OR Resistance* OR Sport* OR Athletic* OR Train* OR Sedentary)) OR (DE "Nutrition" OR DE "Food" OR DE "Diets" OR DE "Body Weight" OR DE "Weight Control" OR DE "Calories" OR DE "Body Mass Index" OR DE "Overweight" OR DE "Weight Loss" OR DE "Obesity" OR TI(Healthy diet* OR Healthy eating* OR Nutrition* OR Diet* OR Food* OR Weight loss* OR Weight loss program* OR Weight reduction* OR Weight reduction program* OR Body weight* OR Overweight* OR Obesity* OR Overnutrition* OR Hypernutrition*) OR AB(Healthy diet* OR Healthy eating* OR Nutrition* OR Diet* OR Food* OR Weight loss* OR Weight loss program* OR Weight reduction* OR Weight reduction program* OR Body weight* OR Overweight* OR Obesity* OR Overnutrition* OR Hypernutrition*)))<br>**AND**<br>(DE "Attitudes" OR DE "Motivation" OR DE "Health Personnel Attitudes" OR DE "Therapist Attitudes" OR DE "Psychologist Attitudes" OR DE "Extrinsic Motivation" OR DE "Intrinsic Motivation" OR TI(Barrier* OR Facilitator* OR Enabler* OR Driver* OR Motivat* OR Opinion* OR View* OR Attitude* OR Expectation* OR Incentive* OR Disincentive* OR Belief* OR Influencing factor* OR Experience* OR Perspective* OR Perception* OR Hinder* OR Impediment* OR Obstacle*) OR AB(Barrier* OR Facilitator* OR Enabler* OR Driver* OR Motivat* OR Opinion* OR View* OR Attitude* OR Expectation* OR Incentive* OR Disincentive* OR Belief* OR Influencing factor* OR Experience* OR Perspective* OR Perception* OR Hinder* OR Impediment* OR Obstacle*))
#2<br>(14 hits)	#1 **AND** RD 20190101-

<u>The Cochrane Library</u>

#1<br>(299 hits)	((Health personnel* OR Health professional* OR Medical staff* OR Clinician* OR Medical specialist* OR Health care* OR Healthcare* OR Primary care* OR Secondary care* OR Allied health personnel* OR Allied health professional* OR Paramedic* OR Nurse* OR Nursing staff* OR Nurse practit* OR Physician assistant* OR Physicians assistant* OR Nutritionist* OR Dietician* OR Dietitian* OR Physical therapist* OR Physiotherapist* OR Lifestyle coach* OR Lifestyle counselor* OR Physician* OR Orthopedic surgeon* OR Orthopedist* OR General practitioner* OR General practice* OR Psychologist*)<br>**AND**<br>(Osteoarthrit* OR Osteoarthro* OR Degenerative arthrit*) AND (Hip* OR Cox* OR Acetabulofemoral joint* OR Knee* OR Superior tibulofibular joint* OR Patellofemoral* OR Lower extremit* OR Lower limb*)<br>**AND**<br>((Life style* OR Lifestyle* OR Behavior* OR Behaviour* OR Habit* OR Risk reduction* OR Early therap* OR Secondary prevention* OR Tertiary

For peer review only

	prevention* OR Disease prevention*) OR (Physical activit* OR Physical training* OR Physical fitness* OR Physical condition* OR Physical therap* OR Physiotherap* OR Exercis* OR Run* OR Jog* OR Walk* OR Bicycl* OR Swim* OR Strength* OR Resistance* OR Sport* OR Athletic* OR Train* OR Sedentary) OR (Healthy diet* OR Healthy eating* OR Nutrition* OR Diet* OR Food* OR Weight loss* OR Weight loss program* OR Weight reduction* OR Weight reduction program* OR Body weight* OR Overweight* OR Obesity* OR Overnutrition* OR Hypernutrition*))<br>**AND**<br>(Barrier* OR Facilitator* OR Enabler* OR Driver* OR Motivat* OR Opinion* OR View* OR Attitude* OR Expectation* OR Incentive* OR Disincentive* OR Belief* OR Influencing factor* OR Experience* OR Perspective* OR Perception* OR Hinder* OR Impediment* OR Obstacle*)):ti,ab,kw
#2<br>(632 hits)	#1

For peer review only

Supplemental File 2. Quality assessment of the included studies based on the Mixed Methods Appraisal Tool[a]

**Reference[b]**	**Screening questions[c]**		**1. Qualitative[d]**					**4. Quantitative descriptive[e]**					**5. Mixed-methods[f]**				
	**S1**	**S2**	**1.1**	**1.2**	**1.3**	**1.4**	**1.5**	**4.1**	**4.2**	**4.3**	**4.4**	**4.5**	**5.1**	**5.2**	**5.3**	**5.4**	**5.5**
Allison (2019) [27]	+	+	+	+	?	+	+										
Bossen (2016) [28][g]	+	+	?	+	?	-	?										
Christiansen (2020) [29]	+	+	?	+	?	+	+										
Davis (2018) [30]	+	+	?	+	?	-	?										
De Rooij (2014) [31]	+	+	?	+	?	-	?										
Egerton (2017) [32]	+	+	?	+	?	+	+										
Egerton (2018) [33]	+	+	?	+	?	+	+										
Hinman (2016) [34]	+	+	+	+	+	+	+										
Hinman (2017) [35]	+	+	?	+	?	+	+										
Knoop (2020) [36]	+	+	?	+	?	-	?										
Law (2019) [37]	+	+	?	+	?	+	+										
Lawford (2019) [38]	+	+	?	+	?	+	+										
Lawford (2020) [39]	+	+	?	+	?	+	+										
Lawford (2021) [40]	+	+	?	+	?	+	+										
MacKay (2018) [41]	+	+	?	+	?	+	+										
MacKay (2020) [42]	+	+	?	+	?	+	+										
Mann (2011) [43]	+	+	?	+	?	+	+										
Miller (2020) [44]	+	+	?	+	?	-	+										
Nielsen (2014) [45]	+	+	?	+	?	+	+										
Okwera (2019) [46]	+	+	?	+	?	+	+										
Poitras (2010) [47]	+	+	?	+	?	-	+										
Rosemann (2006) [48]	+	+	?	+	?	-	?										
Selten (2017) [49]	+	+	?	+	?	+	+										
Tang (2020) [50]	+	+	?	+	?	+	+										
Teo (2020) [51]	+	+	?	+	?	+	+										
Wallis (2020) [52]	+	+	?	+	?	+	+										
Cottrell (2016) [53]	+	+						+	+	+	-	+					
Duarte (2019) [54]	+	+						+	?	+	+	?					
Hill (2018) [55]	+	+						+	?	+	-	+					
Hill (2018) [56]	+	+						-	?	+	?	+					
Hofstede (2016) [57]	+	+						+	?	+	-	+					
Lawford (2018) [58]	+	+						+	?	+	?	+					

For peer review only

Reid (2014) [59]	+	+						+	?	+	-	+					
De Rooij (2020) [60]	+	+	?	+	?	-	?	+	?	+	+	+	?	+	?	+	-
Holden (2009) [61]	+	+	?	+	?	+	+	+	?	+	-	+	+	+	+	+	-
Kloek (2020) [62]	+	+	?	+	?	+	+	+	?	+	-	+	+	+	?	-	-

[a] + = yes; - = no, ? = can't tell
[b] The numbers in brackets ('[…]') correspond to the reference numbers used in the main text of the manuscript.
[c] S1 = Are there clear research questions?; S2 = Do the collected data allow to address the research questions?
[d] 1.1 = Is the qualitative approach appropriate to answer the research question?; 1.2 = Are the qualitative data collection methods adequate to address the research question?; 1.3 = Are the findings adequately derived from the data?; 1.4 = Is the interpretation of results sufficiently substantiated by data?; 1.5 = Is there coherence between qualitative data sources, collection, analysis and interpretation?
[e] 4.1 = Is the sampling strategy relevant to address the research question?; 4.2 = Is the sample representative of the target population?; 4.3 = Are the measurements appropriate?; 4.4 = Is the risk of nonresponse bias low?; 4.5 = Is the statistical analysis appropriate to answer the research question?
[f] 5.1 = Is there an adequate rationale for using a mixed methods design to address the research question?; 5.2 = Are the different components of the study effectively integrated to answer the research question?; 5.3 = Are the outputs of the integration of qualitative and quantitative components adequately interpreted?; 5.4 = Are divergences and inconsistencies between quantitative and qualitative results adequately addressed?; 5.5 = Do the different components of the study adhere to the quality criteria of each tradition of the methods involved?
[g] The MMAT was only applied to the section regarding the pilot study on the feasibility of the blended intervention.

For peer review only

Supplemental File 3. Full overview of all extracted factors per domain

<u>Explanation</u>
- In the tables below, all extracted factors per subcategory of each domain are presented. The colors of the subcategories correspond to barriers (red), facilitators (green) or unclear factors (orange).
- Column "Description": (*) at the end of the description indicates that the factor is derived from a close-ended question or attitude statement.
- Column "Reference": the numbers in brackets ('[…]') correspond to the reference numbers used in the main text of the manuscript.
- Abbreviations: ACSM: American College of Sports Medicine; BMI: body mass index; CBT: cognitive behavioral therapy; CKP: chronic knee pain; CPG: clinical practice guideline; GP: general practitioner; HCP: healthcare professional; LI: lifestyle intervention; LMP: Lifestyle Management Programme; NSAID: non-steroidal anti-inflammatory drug; NWBE: non-weight bearing quadriceps strengthening exercise; OA: osteoarthritis; PCST: pain coping skills training; PT: physiotherapist; RCT: randomized controlled trial; TKA: total knee arthroplasty; TJA: total joint arthroplasty; WBE: weight bearing functional exercise.

<u>Domain 1: Intervention factors</u>

Effectiveness

Description	Reference
LIs have little or no effect on OA (barrier)	
Not certain that exercise works	Christiansen (2020) [29]
Advice to exercise and lose weight does not work	Egerton (2017) [32]
Dubious about effect of exercise and weight-management advice on reducing symptoms	Egerton (2018) [33]
Surgical methods have the best outcomes	Miller (2020) [44]
Lack of confidence in clinical effectiveness of physiotherapy treatments	Okwera (2019) [46]
Limited impact of weight loss on established knee OA (more effective as a primary prevention strategy)	Poitras (2010) [47]
Questioning direct relationship between weight and knee OA (numerous other factors associated)	Poitras (2010) [47]
Less certain about effectiveness of physical therapy (benefits variable or difficult to prove)	Selten (2017) [49]
Increasing the overall activity levels does not/might not stop the knee problem getting worse (*)	Cottrell (2016) [53]
There is a paucity of evidence in regards to the effectiveness of physiotherapy treatment for OA hip and/or knee (*)	Reid (2014) [59]
Past experience has shown physiotherapy to be ineffective (*)	Reid (2014) [59]
Increasing overall activity levels does not/might not stop the knee problem getting worse (*)	Holden (2009) [61]
Increasing the strength of the muscles around the knee does not/might not stop the knee problem getting worse (*)	Holden (2009) [61]
Knee problems are not/might not be improved by general exercise (*)	Holden (2009) [61]
Potential effects of LIs are difficult to accomplish (barrier)	
Weight loss is difficult (multiplicity of factors need to be addressed, often involving change in lifestyle)	Poitras (2010) [47]

Vicious circle (pain when exercising, people move less/eat more due to frustration/sometimes depression)	Rosemann (2006) [48]
Achieving patient behavior change is difficult (*)	Cottrell (2016) [53]
Human nature (as patient-centered barrier to adherence)	Holden (2009) [61]
LIs have positive effects on affected joint(s) (facilitator)	
Functional improvements experienced by patients	Hinman (2017) [35]
Improvements in patient pain and function	Lawford (2019) [38]
Large improvements in knee pain	Lawford (2021) [40]
Treatment could improve clients' symptoms (e.g. reduce pain, increase function)	MacKay (2018) [41]
Treatment could potentially slow progression of symptoms	MacKay (2018) [41]
Improve people's symptoms early in treatment (to gain buy-in)	MacKay (2020) [42]
Benefits of activity on knee mobility	Poitras (2010) [47]
Activity necessary for the knee's health	Poitras (2010) [47]
Weight loss improves pain and joint function	Poitras (2010) [47]
Benefits of weight reduction for relieving symptoms of knee/hip OA	Selten (2017) [49]
Beneficial effects of physical therapy in reducing pain/stiffness and potential effects on cartilage	Selten (2017) [49]
Exercise therapy may be effective by giving more muscular support for joints	Wallis (2020) [52]
Knee problems are improved by quadriceps strengthening exercises (*)	Cottrell (2016) [53]
Knee problems are improved by general exercise (e.g. walking or swimming) (*)	Cottrell (2016) [53]
Increasing the strength of the muscles around the knee stops the knee problem getting worse (*)	Cottrell (2016) [53]
Exercise is beneficial for OA (*)	Lawford (2018) [58]
Knee problems are improved by local strengthening exercises (*)	Holden (2009) [61]
LIs have positive effects on general health (facilitator)	
Lifestyle treatments benefited other chronic conditions	Egerton (2018) [33]
Rapid weight loss was primary driver of motivation	Lawford (2021) [40]
Benefits of activity on general wellbeing	Poitras (2010) [47]
Weight loss effective at improving mobility in general	Poitras (2010) [47]
Weight loss also benefits mobility in general	Poitras (2010) [47]
Beneficial effects of physical therapy in reducing weight and for increasing mobility/posture/coordination	Selten (2017) [49]
Community interventions are effective at achieving sufficient and sustained weight loss (*)	Hill (2018) [56]
Patients benefit from weight loss (*)	Hofstede (2016) [57]
LIs have positive mental effects (facilitator)	
Enthusiastic about the program and described the results (e.g. it was empowering)	Davis (2018) [30]
Greater confidence to exercise among patients	Hinman (2017) [35]
Reminding patients of opportunity to self-manage	Law (2019) [37]
Increased confidence to self-manage	Lawford (2019) [38]
Positive lifestyle changes (patients) (e.g. thinking differently)	Lawford (2021) [40]
Physical therapy useful in increasing patients self-management in coping with/acceptance of symptoms	Selten (2017) [49]

For peer review only

Exercise therapy may be effective by giving opportunity to improve confidence about activities/mobility	Wallis (2020) [52]
Non-surgical treatments motivate patients to do things themselves (*)	Hofstede (2016) [57]
LIs have positive effects (not further specified) (facilitator)	
Positive impact on patients of personalized attention from coach and from advice/education they provided	Hinman (2016) [34]
Emphasising health benefits of programme	Law (2019) [37]
Physical therapy helpful for patients most of the time	Miller (2020) [44]
Value of lifestyle advice related to knee and hip OA	Selten (2017) [49]
Non-pharmacological, non-surgical treatment was considered useful to delay surgery	Selten (2017) [49]
Positive about program (alternative approach and opportunity to avoid a joint replacement)	Wallis (2020) [52]
Improvement in the physical condition of participants	Duarte (2019) [54]
Good results of physical therapy (*)	Hofstede (2016) [57]

Safety

Description	Reference
LIs are unsafe or have negative effects (barrier)	
Being apprehensive about aggravating pain in patients	Lawford (2020) [39]
Tending to avoid pushing patients in usual clinical practice	Lawford (2020) [39]
Potential further damage to the knee due to activity	Poitras (2010) [47]
Urging caution to patients about participating in higher impact exercise/activities	Wallis (2020) [52]
Fear of increasing symptoms (as barrier to prescribing exercise)	Holden (2009) [61]
Causing disease progression, particularly through weight-bearing activities (as barrier to prescribing exercise)	Holden (2009) [61]
Exacerbating patient's comorbidities (as barrier to prescribing exercise)	Holden (2009) [61]
General exercise is not/might not be safe for everybody to do (*)	Holden (2009) [61]
Local strengthening exercises for the knee are not/might not be safe for everybody to do (*)	Holden (2009) [61]
LIs are safe (facilitator)	
Quadriceps strengthening exercises for the knee are safe for everybody to do (*)	Cottrell (2016) [53]
General exercise (e.g. walking or swimming) is safe for everybody to do (*)	Cottrell (2016) [53]
Only few drawbacks for the use of non-surgical treatments (*)	Hofstede (2016) [57]
Research environment or protocols provide a safety net (facilitator)	
Less afraid to increase training intensity (preventing adverse events by tailoring programs to individual's capacity)	De Rooij (2014) [31]
Safety net provided by research environment (e.g. patients were previously screened for comorbidities/red flags)	Hinman (2017) [35]
There was a safety net in place with the trial (each patient had been screened)	Lawford (2019) [38]
Experiences in study helped them push patients through more pain than they would have previously	Lawford (2020) [39]

Design

Description	Reference
Non-optimal content or structure of LIs (barrier)	
Structure/timing of exercise program restricted capacity to modify exercises/provide adequate follow-up	Hinman (2016) [34]
Maximum number of four sessions was considered too low in many patients	Knoop (2020) [36]
Behavioral approach in exercise therapy and advice to visit GP were considered unnecessary for most patients	Knoop (2020) [36]
Program factors (e.g. single discipline led intervention)	Wallis (2020) [52]
Services do not meet expectations (*)	Cottrell (2016) [53]
E-Exercise does not/might not contain all essential elements for the treatment of hip/knee OA (*)	Kloek (2020) [62]
The content of e-Exercise is not/might not be aligned with my opinion about treating patients with OA (*)	Kloek (2020) [62]
The intervention provided through e-Exercise is not/might not be appropriate for the average patient with OA (*)	Kloek (2020) [62]
Patients who were treated with e-Exercise were (perhaps) not generally positive about the intervention (*)	Kloek (2020) [62]
Challenges for patients during participation in LIs (barrier)	
Mental effort required for WBE program was challenging for patients	Lawford (2020) [39]
Physical challenge was the complexity of WBE program	Lawford (2020) [39]
Straight leg raise challenging in NWBE program	Lawford (2020) [39]
Volume of resources could be overwhelming/confusing for some patients	Lawford (2021) [40]
Difficulty for patients with PCST component (cognitive restructuring techniques)	Nielsen (2014) [45]
Challenges for HCPs during delivery of LIs (barrier)	
e-Exercise must be adapted for suitable integration into practice (e.g. no insight into modules patients receive)	Bossen (2016) [28]
Class required intense supervision, which was difficult to provide when most participants were new	Davis (2018) [30]
Challenges of supervision when space did not allow clear line of sight	Davis (2018) [30]
Challenges associated with cuff weights used to apply resistance in NWBE program	Lawford (2020) [39]
The lay out of the protocol does not/might not facilitate its usage in daily practice (*)	De Rooij (2020) [60]
Clarity of instruction manual and course (lack of)	Kloek (2020) [62]
Positive experiences with or suggestions for improving the content or structure of LIs (facilitator)	
Positive feedback regarding the content of e-Exercise	Bossen (2016) [28]
First education session was critical to reducing the participant's anxiety related to exercising	Davis (2018) [30]
Importance of empowering the patients rather than 'pushing' them, achieved by 'giving choices'	Davis (2018) [30]
Positive comments about the exercise regimen	Hinman (2016) [34]
Structure provided by protocol/structure of exercises (how patients included them into daily routine)	Hinman (2016) [34]
Multidisciplinary nature of LMP (whole-person, intensive and functional approach)	Law (2019) [37]
Standardization was viewed as important for monitoring and evaluation purposes	Law (2019) [37]
Helpful social impact of group-based programme	Law (2019) [37]
Long-term follow-up consultations would be beneficial	Lawford (2021) [40]
Extremely positive about educational resources provided	Lawford (2021) [40]
More information about healthy eating beyond meal replacement phase could be included	Lawford (2021) [40]
Exercise/physical activity program was an important part of intervention	Lawford (2021) [40]

For peer review only

Favorably comments on program content (positive way to help people be proactive about their pain)	Nielsen (2014) [45]
Importance of PCST component (cognitive restructuring techniques)	Nielsen (2014) [45]
Structure of PCST sessions (overview/practice review/covering new skill/practice planning) worked well	Nielsen (2014) [45]
A more holistic program as part of a multidisciplinary model of service was preferred	Wallis (2020) [52]
Value of program's structure and peer (group) support	Wallis (2020) [52]
Name of program (‘Good Life with OsteoArthritis’) implied optimism and positive outcome	Wallis (2020) [52]
Received positive feedback from their patients about program	Wallis (2020) [52]
Some contents of the protocol are not/might not be incorrect (*)	De Rooij (2020) [60]
The protocol is applicable to OA patients with comorbidity that I see in my clinical practice (*)	De Rooij (2020) [60]
Important to extend the intake phase to at least to 45 min	De Rooij (2020) [60]
Completeness of web-based application (exercises/assignments/information)	Kloek (2020) [62]
Perception that e-Exercise is an appropriate treatment option for subgroup of OA patients	Kloek (2020) [62]
Ease for patients during participation in LIs (facilitator)	
NWBE program was generally easier for patients to follow (mental effort)	Lawford (2020) [39]
Simplicity and convenience of meal replacements	Lawford (2021) [40]
Providing trial of sessions to assist patients to get started (suggestion for promotion and referrals)	Wallis (2020) [52]
Ease for HCPs during delivery of LIs (facilitator)	
Initial classes needed to be small with rolling recruitment very beneficial	Davis (2018) [30]
List of restrictions for exercise therapy was conveniently arranged checklist for diagnostic and treatment phases	De Rooij (2014) [31]
Suggestion to increase feasibility by reducing the protocols to three main protocols	De Rooij (2014) [31]
Requirements of treatment protocol freed therapists to notice and reflect on impact of the interventions	Hinman (2016) [34]
Structured protocol allowed to experience different OA treatment regimen/observe and learn from impact	Hinman (2016) [34]
NWBE program was easier to prescribe (mental effort)	Lawford (2020) [39]
Easier to prescribe and progress NWBE than WBE program (physical complexity)	Lawford (2020) [39]
Training workshop as good introduction to content and process of delivering PCST program	Nielsen (2014) [45]
Weekly group interaction crucial to being able to deliver intervention effectively/problem-solve issues	Nielsen (2014) [45]
Input from supervising psychologist crucial to being able to deliver intervention effectively/problem-solve issues	Nielsen (2014) [45]
Would have liked more role-playing experience prior to beginning trial treatments	Nielsen (2014) [45]
Regular group meetings were considered very important (if not essential) for delivery of PCST program	Nielsen (2014) [45]
Value of having a psychologist involved throughout the program, their professional input was helpful	Nielsen (2014) [45]
The recommendations over adapting the diagnostic phase (history taking and physical examination) in the protocol are clear and understandable (*)	De Rooij (2020) [60]
The recommendations over adapting the OA exercise therapy in the protocol are clear and understandable (*)	De Rooij (2020) [60]
Useful to plan follow up/refreshment training to repeat/discuss content of course/protocol and application	De Rooij (2020) [60]
Shortening the protocol would increase user-friendliness	De Rooij (2020) [60]
The instruction course and manual assisted me so that I knew how to work with e-Exercise (*)	Kloek (2020) [62]

Personalized treatment

For peer review only

Description	Reference
Insufficient ability to provide personalized treatment within LIs (barrier)	
Concerned the service would not be able to provide individualized management for a very diverse population	Egerton (2017) [32]
Requirements of RCT potentially created a barrier to responding to where the client was	Nielsen (2014) [45]
Less satisfied about the applicability of e-Exercise for only one diagnosis	Kloek (2020) [62]
I do not/might not have enough influence on the content of patients' individual e-Exercise program (*)	Kloek (2020) [62]
Ability and importance of providing personalized treatment within LIs (facilitator)	
Flexibility was valuable when tackling local participation challenges	Law (2019) [37]
Tailoring exercise programs to individual patient would overcome some challenges	Lawford (2020) [39]
Tailoring treatment to a person's goals/interests	MacKay (2020) [42]
Need to consider personal context by integrating people's home exercises into daily activities/other life demands	MacKay (2020) [42]
Some modules worked better than others (depending on the individual patient and context)	Nielsen (2014) [45]
The belief that a more flexible approach responsive to patient needs was required in their practice	Nielsen (2014) [45]
Exercise programs have to be individualized to each patient by the PT	Poitras (2010) [47]
Importance of tailored exercise program	Teo (2020) [51]
Exercise for CKP is most beneficial when it is tailored to meet individual patient needs (*)	Cottrell (2016) [53]
A standard set of exercises is not/might not be sufficient for every patient with chronic knee problems (*)	Cottrell (2016) [53]
The protocol gives the opportunity to make your own decisions regarding history taking, physical examination, and treatment (*)	De Rooij (2020) [60]
A standard set of exercises is not/might not be sufficient for every patient with knee OA (*)	Holden (2009) [61]
Exercise for knee OA is most beneficial when it is tailored to meet individual patient needs (*)	Holden (2009) [61]
More flexibility in web-based application (intervention duration, number of sets/repetitions, type of exercises)	Kloek (2020) [62]
More flexibility in intervention (more possibilities to personalize to individual needs)	Kloek (2020) [62]

Accessibility

Description	Reference
LIs are unavailable or inaccessible (barrier)	
Most of the patients did not meet study inclusion criteria	Bossen (2016) [28]
Lack of availability of support services (e.g. community-based rehabilitation programs) in remote locations	Egerton (2018) [33]
Long waiting lists for support services (e.g. community-based rehabilitation programs)	Egerton (2018) [33]
Lack of infrastructure or local programmes (particularly in rural settings)	MacKay (2018) [41]
Clients often had a waiting period before accessing care	MacKay (2018) [41]
Lack of facilities to promote continuing exercise in community	Mann (2011) [43]
Wait for physiotherapy was too long	Mann (2011) [43]
Inaccessible treatment options within organization	Miller (2020) [44]
Limitations to accessing services (e.g. lack of facilities, costs) (*)	Cottrell (2016) [53]

For peer review only

Weight management services are not/might not be adequately commissioned in my area (*)	Hill (2018) [56]
Lack of availability of physiotherapy	Reid (2014) [59]
Poor links to community facilities such as local leisure centres	Holden (2009) [61]
Costs of LIs to patients (barrier)	
Concern that uptake would be negatively impacted if patients were required to pay	Egerton (2017) [32]
Concerns regarding financial cost to patients when considering referral to other services	Egerton (2018) [33]
Cost was a factor in whether clients could access facilities/programmes	MacKay (2018) [41]
Costs to patients (lack of insurance coverage/high co-pays for specific services/time off work/travel expenses)	Miller (2020) [44]
Costs (extrinsic barrier for patient adherence)	Teo (2020) [51]
Cost (program access barrier)	Wallis (2020) [52]
LIs are not feasible or sustainable (barrier)	
Concern for overcomplicated system when service is not compatible/complementary with existing initiatives	Egerton (2017) [32]
Not seeing need (already adequate skills/resources to support OA patient self-management and lifestyle change)	Egerton (2017) [32]
Not seeing need (advice already given at their practice would be unhelpfully repeated)	Egerton (2017) [32]
Concern regarding long-term service sustainability	Egerton (2017) [32]
The addition of a care support team may add complexities to management	Egerton (2017) [32]
In my daily clinical practice I can (perhaps) not integrate working according to the protocol well (*)	De Rooij (2020) [60]
I do not/might not treat enough patients with knee OA and comorbidity to apply the protocol (*)	De Rooij (2020) [60]
The protocol does not/might not fit well with my working methods of daily clinical practice (*)	De Rooij (2020) [60]
Total amount of knee OA patients with comorbidity was lower than expected	De Rooij (2020) [60]
Inconvenience to patients when accessing LIs (barrier)	
Weather (extrinsic barrier for patient adherence)	Teo (2020) [51]
Transport, waiting time and parking related to attendance (program access barrier)	Wallis (2020) [52]
Geography (program access barrier)	Wallis (2020) [52]
Available session times (program access barrier)	Wallis (2020) [52]
Geographical problems (e.g. remote location, scared to walk in local area) (*)	Cottrell (2016) [53]
LIs are available or accessible, or suggestions for improvement (facilitator)	
More likely to engage with the care support team if it enabled more affordable/accessible allied health	Egerton (2017) [32]
LMP would benefit from extension of inclusion criteria (patients with less severe OA and lower BMI)	Law (2019) [37]
Benefits of having infrastructure and programmes available in their communities	MacKay (2018) [41]
Triage service (as suggestion for physiotherapy service improvement)	Okwera (2019) [46]
A web-based physiotherapy service (as suggestion for physiotherapy service improvement)	Okwera (2019) [46]
Reduced waiting times (as suggestion for physiotherapy service improvement)	Okwera (2019) [46]
Availability of non-surgical treatments (*)	Hofstede (2016) [57]
Good access to physiotherapy in area (*)	Reid (2014) [59]
LIs are feasible or sustainable (facilitator)	
Need for clarity about how the new service would integrate with existing schemes and payment structures	Egerton (2017) [32]

For peer review only

Seeing need (advice/recommendations may need to be reinforced/provided over several health care episodes)	Egerton (2017) [32]
Seeing need (extra time and encouragement for the patient would result in better outcomes)	Egerton (2017) [32]
Importance of broad acceptance (patients/doctors/health service funders) if new service is to continue long term	Egerton (2017) [32]
Model of stratified care easy to apply and having added value for daily practice	Knoop (2020) [36]
Appreciation of applicability of treatment protocols	Knoop (2020) [36]
Further and ongoing evaluation of the LMP would help to address current challenges	Law (2019) [37]
Interventions in physical therapists' toolbox were not static (changed over time)	MacKay (2020) [42]
The protocol is feasible in daily clinical practice (*)	De Rooij (2020) [60]
In my daily clinical practice, I work with sufficient equipment (including blood pressure meter, saturation meter) to properly apply the protocol (*)	De Rooij (2020) [60]
I have changed my working method (due to the protocol) (*)	De Rooij (2020) [60]
Intake procedure is feasible and implementable	De Rooij (2020) [60]
The more you apply the strategy in daily practice, the easier it is to integrate it in your daily working method	De Rooij (2020) [60]
Convenience for patients when accessing LIs (facilitator)	
Close, convenient locations (suggestion for promotion and referrals)	Wallis (2020) [52]
Appropriate session times for working populations (suggestion for promotion and referrals)	Wallis (2020) [52]
Provision of free parking at health service (suggestion for promotion and referrals)	Wallis (2020) [52]

Telehealth

**Description**	**Reference**
Disadvantages of telehealth in terms of effectiveness (barrier)	
Remote (telephone) delivery is not as good as face-to-face particularly in relation to exercise advice	Egerton (2017) [32]
Inability of a remote service to provide locally relevant information	Egerton (2017) [32]
An exercise program prescribed by a PT over the telephone would not/might not improve a patient's OA (*)	Lawford (2018) [58]
I do not/might not experience that e-Exercise supports patients in doing their exercises at home (*)	Kloek (2020) [62]
Telehealth is not safe for patients or patient/data privacy (barrier)	
Concerns about security of patient data and information confidentiality during the referral process	Egerton (2017) [32]
Using the telephone would not/might not be a safe way for patients to receive a PT-prescribed exercise program for their OA (*)	Lawford (2018) [58]
Challenges for HCPs regarding lack of physical/visual contact (barrier)	
Forced to modify usual habits/rely more on information shared by patients (instead of own physical assessment)	Hinman (2017) [35]
Some discomfort without hands-on assessment (no palpation of patient's knee/hands-on facilitation of exercises)	Hinman (2017) [35]
Assessment of patients could be difficult when consulting via telephone (inability to observe)	Lawford (2019) [38]
Lack of visual/physical contact would limit strategies available when teaching patients an exercise program	Lawford (2019) [38]
I would not/might not be able to adequately monitor a patient's OA over the telephone (*)	Lawford (2018) [58]
I do not/might not like that there would be no physical contact with an OA patient when consulting over the telephone (*)	Lawford (2018) [58]
I do not/might not like that there would be no physical contact with an OA patient when consulting over the internet video (*)	Lawford (2018) [58]
Reduced face-to-face contact interfered with professional autonomy	Kloek (2020) [62]

Other challenges for HCPs regarding feasibility of telehealth (barrier)	
Lack of financial incentive if blended intervention substitutes conventional visits (reduced venues per patient)	Bossen (2016) [28]
Hesitancy to embrace an unfamiliar new service	Egerton (2017) [32]
Patient flexibility could come at a cost to the therapist sometimes (allowed patients to reschedule last minute)	Hinman (2017) [35]
Skype consultations more suitable as adjunctive to usual in-clinic care (initial assessment in person preferred)	Hinman (2017) [35]
Telephone not viewed as primary mode of providing care (only for follow-up)	Lawford (2019) [38]
Some difficulty scheduling telephone consultations during usual day of face-to-face consultations	Lawford (2019) [38]
Using the telephone to consult with an OA patient and prescribe an exercise program would not/might not be easy for me (*)	Lawford (2018) [58]
I would not/might not be as satisfied talking to an OA patient over the telephone as I would be talking to the patient in person in my consulting room (*)	Lawford (2018) [58]
I would not/might not be interested in being involved in a service offering PT-prescribed exercise over the telephone for my people with OA (*)	Lawford (2018) [58]
Using the telephone would not/might not be an acceptable way for me to deliver an exercise program to patients with OA (*)	Lawford (2018) [58]
Using the telephone would not/might not be a useful (practical) way for me to deliver an exercise program to patients with OA (*)	Lawford (2018) [58]
Using the telephone would not/might not be an effective way for me to deliver an exercise program to patients with OA (*)	Lawford (2018) [58]
Technical skills (lack of)	Kloek (2020) [62]
Adaptive capacity to change treatment routines (lack of)	Kloek (2020) [62]
Absence of national e-Health guideline or standard	Kloek (2020) [62]
Loss of income due to substitution of face-to-face session	Kloek (2020) [62]
Patient-related challenges regarding feasibility of telehealth (barrier)	
Most of the patients prefer traditional face-to-face treatments	Bossen (2016) [28]
Hearing and cognitive difficulties as barriers for some patients to being able to interact with the service	Egerton (2017) [32]
Skepticism about whether many patients would embrace such a model (i.e. because of remote-delivery aspect)	Egerton (2017) [32]
Lack of technology affinity (reason for patients' non-willingness to participate in e-Exercise)	Kloek (2020) [62]
Patients preferred regular face-to-face contact	Kloek (2020) [62]
Negative aspects regarding communication and relationship using telehealth (barrier)	
Lack of face-to-face contact difficult/hampered ability to establish normal rapport/build effective relationships	Hinman (2016) [34]
Occasional technical difficulties (e.g. poor internet connection) could disrupt the flow of the consultation	Hinman (2017) [35]
Relationships with patients might be adversely impacted/could be difficult to develop rapport	Lawford (2019) [38]
Difficulties communicating might be experienced when consulting via telephone	Lawford (2019) [38]
Video consultations made it more difficult to have emotional conversations/read non-verbal cues	Lawford (2021) [40]
Benefits of telehealth in terms of effectiveness (facilitator)	
Possibility to extend physical therapy treatment in patient's home environment	Bossen (2016) [28]
Potential to enhance the adherence of home exercises	Bossen (2016) [28]
Empowering effect of home environment on patient adherence with exercise program	Hinman (2017) [35]
Using Skype distilled focus to most important and effective treatment elements to facilitate self-management	Hinman (2017) [35]
Patients more relaxed in home environment/more receptive to the information the therapists provided	Hinman (2017) [35]
Patients could be more comfortable talking about condition/engaging in exercise program from own home	Lawford (2019) [38]

For peer review only

Telephone-delivered care could provide increased opportunities to educate patients about OA	Lawford (2019) [38]
Patient adherence to telephone-delivered exercise program was high	Lawford (2019) [38]
An exercise program prescribed by a PT over the internet video would improve a patient's OA (*)	Lawford (2018) [58]
Added value in terms of exercise adherence (important factor to use web-based application)	Kloek (2020) [62]
Telehealth is safe for patients or patient/data privacy (facilitator)	
Home environment facilitated correct and safe exercise techniques	Hinman (2017) [35]
A patient's privacy would not be violated if I prescribed them an exercise program over the telephone (*)	Lawford (2018) [58]
A patient's privacy would not be violated if I prescribed them an exercise program over the internet video (*)	Lawford (2018) [58]
Using the internet video would be a safe way for patients to receive a PT-prescribed exercise program for their OA (*)	Lawford (2018) [58]
I believe that patient data gathered at the e-Exercise web-application is stored safely (*)	Kloek (2020) [62]
Lack of physical/visual contact not a major issue for HCPs (facilitator)	
Patients responded favorably to the exercises prescribed despite lack of hands-on assessment	Hinman (2017) [35]
Hands-off approach was physically less demanding compared to usual care/contributed to sense of satisfaction	Hinman (2017) [35]
Functional improvements were observable using Skype	Hinman (2017) [35]
Lack of physical and visual contact less of an issue than anticipated	Lawford (2019) [38]
Able to work around the lack of visual contact (erring on the side of caution)	Lawford (2019) [38]
I would get a good understanding of a patient's OA over the telephone (*)	Lawford (2018) [58]
I would get a good understanding of a patient's OA over the internet video (*)	Lawford (2018) [58]
I would be able to adequately monitor a patient's OA over the internet video (*)	Lawford (2018) [58]
Positive attitude or needs of HCPs regarding feasibility of telehealth (facilitator)	
Ease of using Skype for consultations	Hinman (2017) [35]
Quality of technology suitable for providing instructions/prescribing exercises/receiving instantaneous feedback	Hinman (2017) [35]
More effective communication skills would be needed to consult via telephone	Lawford (2019) [38]
It would be necessary to provide patients with pictures or videos of each exercise when consulting via telephone	Lawford (2019) [38]
Experiences providing telephone-delivered care exceeded expectations, resulting in new enthusiasm	Lawford (2019) [38]
Written materials provided to patients helped to prescribe exercises effectively	Lawford (2019) [38]
Training in communication and/or health coaching important to effectively deliver care over telephone	Lawford (2019) [38]
Video consultations were easy and convenient	Lawford (2021) [40]
Using the internet video to consult with an OA patient and prescribe an exercise program would be easy for me (*)	Lawford (2018) [58]
I would be as satisfied talking to an OA patient over the internet video as I would be talking to the patient in person in my consulting room (*)	Lawford (2018) [58]
I would be interested in being involved in a service offering PT-prescribed exercise over the internet video for my people with OA (*)	Lawford (2018) [58]
Using the internet video would be an acceptable way for me to deliver an exercise program to patients with OA (*)	Lawford (2018) [58]
Using the internet video would be a useful (practical) way for me to deliver an exercise program to patients with OA (*)	Lawford (2018) [58]
Using the internet video would be an effective way for me to deliver an exercise program to patients with OA (*)	Lawford (2018) [58]
Advantage of reducing number of treatments	Kloek (2020) [62]
Offering an innovative intervention attracted new patients	Kloek (2020) [62]
That it results in less income is not/might not be a major disadvantage of e-Exercise (*)	Kloek (2020) [62]

Our physiotherapy practice has the intention to use e-Health innovations (*)	Kloek (2020) [62]
Patient-related benefits regarding feasibility of telehealth (facilitator)	
24/7 availability of information and exercises	Bossen (2016) [28]
Service could increase access to support for rural patients	Egerton (2017) [32]
Skype-delivered care convenient for patients (time efficiency/flexibility/access)	Hinman (2017) [35]
Telephone-delivered care would be convenient for patients	Lawford (2019) [38]
Telephone-delivered care could reduce patient costs associated with accessing physiotherapy services	Lawford (2019) [38]
Telephone-delivered care could allow wider variety of patients to access physiotherapy	Lawford (2019) [38]
Noticeable shift in patients' expectations of physiotherapy care (more willing to self-manage their condition)	Lawford (2019) [38]
Telephone-delivered care was convenient for patients	Lawford (2019) [38]
An exercise program prescribed by a PT over the telephone would save a patient money (*)	Lawford (2018) [58]
An exercise program prescribed by a PT over the internet video would save a patient money (*)	Lawford (2018) [58]
Receiving an exercise program from a PT over the telephone would be a convenient form of health care for an OA patient (*)	Lawford (2018) [58]
Receiving an exercise program from a PT over the internet video would be a convenient form of health care for an OA patient (*)	Lawford (2018) [58]
Receiving an exercise program from a PT over the telephone would save the patient time (*)	Lawford (2018) [58]
Receiving an exercise program from a PT over the internet video would save the patient time (*)	Lawford (2018) [58]
Using the telephone would be an affordable way for patients to receive a PT-prescribed exercise program for their OA (*)	Lawford (2018) [58]
Using the internet video would be an affordable way for patients to receive a PT-prescribed exercise program for their OA (*)	Lawford (2018) [58]
Positive aspects regarding communication and relationship using telehealth (facilitator)	
Developed a strong rapport with patients over the telephone	Lawford (2019) [38]
Consulting via telephone forced to focus on effective conversations with patients (more personal level)	Lawford (2019) [38]
Pleasantly surprised by experience with video consultations (had some of the best conversations)	Lawford (2021) [40]

Domain 2: Individual HCP factors

Expertise

Description	Reference
Lack of knowledge or skills around LIs or promoting behavioral change (barrier)	
Lack of knowledge around appropriate interventions for weight loss	Allison (2019) [27]
Uncertainty about how to enact their understanding of relationship between weight and knee OA	Allison (2019) [27]
Limited knowledge of exercise prescription (uncertainty of what exercise to recommend/how much)	Christiansen (2020) [29]
Not received sufficient training on exercise/lack of education	Christiansen (2020) [29]
Knowledge of exercise and weight-loss treatments is sometimes inaccurate or inadequate	Egerton (2018) [33]
Reduced confidence with providing suitable exercise and weight loss advice	Egerton (2018) [33]
Lack of skills in promoting readiness and motivation for lifestyle treatments	Egerton (2018) [33]
Variability in confidence to provide weight management (not confident)	MacKay (2018) [41]

For peer review only

Not confident in knowledge about weight management	MacKay (2020) [42]
Concerns about capacity to learn/not having skills to fulfill study expectations/deal with challenging patients	Nielsen (2014) [45]
Not have sufficient skills to present PCST component (cognitive restructuring techniques) effectively	Nielsen (2014) [45]
Some process skills were dissimilar to pre-existing clinical communication skills and challenging to use	Nielsen (2014) [45]
Lack of knowledge about CBT (necessary to participate in training/RCT to fully appreciate value of CBT to practice)	Nielsen (2014) [45]
Unclear on amount and type of activity necessary to obtain benefits without further damaging the knee	Poitras (2010) [47]
Uncertainties about dosage/frequency/type of physical activity	Selten (2017) [49]
Unaware about practice guidelines in relation to aerobic exercise prescription/weight loss/pain management	Tang (2020) [50]
Knowledge about BMI/weight management was particularly poor (e.g. relying on visual estimations)	Tang (2020) [50]
Limited knowledge of how to address weight management	Tang (2020) [50]
Less awareness about aerobic exercise prescription	Tang (2020) [50]
Reduced confidence with recommending individual weight/pain management plans (discuss in general terms)	Tang (2020) [50]
Lack of confidence/knowledge/skills in implementing evidence into practice (e.g. weight management)	Teo (2020) [51]
I don't have/might not have the required knowledge and training around obesity care (*)	Hill (2018) [56]
I do not/might not have sufficient knowledge about knee OA exercise therapy and comorbidity to apply the protocol in daily clinical practice (*)	De Rooij (2020) [60]
Gaps in knowledge/skills (including how to facilitate behavior change, particularly with less motivated patients)	Holden (2009) [61]
Lack of knowledge or skills around OA care in general (barrier)	
General lack of expertise/interest in OA (that could lead to inappropriate referral/suboptimal access to services)	Mann (2011) [43]
Lack of physician education on OA care	Miller (2020) [44]
Only two GPs had clear understanding of clinical guidelines on OA	Okwera (2019) [46]
No knowledge about treatment	Rosemann (2006) [48]
Lack of knowledge or skills around specific resources (barrier)	
The issue is not a lack of suitable patient resources but awareness of them	Egerton (2018) [33]
Inability to discuss specific details of ACSM guideline	Tang (2020) [50]
I do not/might not have sufficient skills to apply the protocol in daily clinical practice (*)	De Rooij (2020) [60]
I do not/might not read the protocol sufficiently to remember any of its contents (*)	De Rooij (2020) [60]
Having or improving knowledge or skills around LIs or promoting behavioral change (facilitator)	
Importance of having highly effective communication skills	Egerton (2018) [33]
Value of monitoring/encouraging patients to develop own understanding of links between exercise/pain	Hinman (2016) [34]
Confident in capabilities/skills to use strategies they believed to be effective within scope of practice	MacKay (2018) [41]
Variability in confidence to provide weight management (confident)	MacKay (2018) [41]
Clinical experience helped to read the person’s situation (identify approach to motivate them)	MacKay (2020) [42]
Postgraduate continuing professional development courses to expand toolkit of therapeutic interventions	MacKay (2020) [42]
Confidence in addressing weight management	MacKay (2020) [42]
Opportunity to review PCST skills and learn more structured/deliberate ways of incorporating these into practice	Nielsen (2014) [45]
Increasing confidence in using PCST skills over the course of the study	Nielsen (2014) [45]
Improved interpersonal skills with general clinical patients as a result of participating in the study	Nielsen (2014) [45]

Reasonable understanding of role physiotherapy plays in management of lower-limb OA	Okwera (2019) [46]
Knowledge/confidence in providing treatments related to strengthening and range of motion	Tang (2020) [50]
Confident in providing justifications for non-routinely adhering to guidelines (range of motion exercises)	Tang (2020) [50]
Being able to describe how they will manage pain during strengthening exercise	Tang (2020) [50]
Having or improving knowledge or skills around OA care in general (facilitator)	
Need for tailored GP education to improve confidence	Egerton (2018) [33]
Importance of provider knowledge regarding OA management	Miller (2020) [44]
Physician education on OA management can affect both provider and patient attitudes	Miller (2020) [44]
Training sessions (as suggestion for physiotherapy service improvement)	Okwera (2019) [46]
Knowledge about treatment	Rosemann (2006) [48]
Available resources might improve knowledge and decision-making (facilitator)	
Protocols offered guidance in setting up treatment/making clinical decisions/adapting treatment to comorbidity	De Rooij (2014) [31]
List of restrictions was helpful in process of clinical decision making	De Rooij (2014) [31]
Being aware about ACSM guidelines	Tang (2020) [50]
The protocol supports me in clinical reasoning (*)	De Rooij (2020) [60]
The protocol is supporting the improvement of my knowledge regarding knee OA exercise therapy and comorbidity (*)	De Rooij (2020) [60]
The protocol is supportive in which comorbidity-related symptoms I need to monitor before, during and after treatment (*)	De Rooij (2020) [60]
More insight into exercise tolerance/more background knowledge to make clinical decision by using strategy	De Rooij (2020) [60]
Clinical experience (unclear factor)	
Driven by their professional experience of what does and doesn't work/trial and error	MacKay (2020) [42]
Treatments were based more on what works clinically (opposed to scientific evidence)	MacKay (2020) [42]

Attitude

Description	Reference
Negative attitude toward LIs (barrier)	
Assigning low priority to exercise as treatment	Christiansen (2020) [29]
GP does not prioritise exercise (*)	Cottrell (2016) [53]
It is not/might not be important that people with knee OA increase their overall activity levels (*)	Holden (2009) [61]
Negative attitude toward guidelines or protocols (barrier)	
Frustrations about lack of autonomy with decision-making	Okwera (2019) [46]
Negativity toward guidelines (clinical reasoning more important)	Okwera (2019) [46]
Positive attitude toward LIs (facilitator)	
Acknowledging that weight loss (when someone is overweight) is important	Egerton (2018) [33]
Identifying weight management as important	MacKay (2018) [41]
Perception that exercise and physical activity were central to management	MacKay (2020) [42]
Acknowledging that weight management was a component of management	MacKay (2020) [42]

Expecting to utilize/continue integrating PCST in general clinical work as physical therapist (beyond the study)	Nielsen (2014) [45]
There is place for each (self-management programs/physiotherapy/orthopedic consultants) in OA management	Okwera (2019) [46]
Many interventions should be used before resorting to medication (including physiotherapy)	Poitras (2010) [47]
Other interventions (including physiotherapy) should be used before paracetamol	Poitras (2010) [47]
NSAIDs alone are not sufficient to appropriately treat inflammation and have to be combined with physiotherapy	Poitras (2010) [47]
Patients should be encouraged to resume/maintain daily activities	Poitras (2010) [47]
Knowing the importance of weight management for knee OA	Tang (2020) [50]
Aware that being overweight/obese is risk factor for knee OA/losing weight is important	Teo (2020) [51]
Physiotherapy (referral) needs to be prioritised	Cottrell (2016) [53]
It is important that people with CKP increase their overall activity levels (*)	Cottrell (2016) [53]
Exercise for CKP should (perhaps) preferably be used before drug treatment has been tried (*)	Cottrell (2016) [53]
Weight loss should be the first-line treatment in the management of obese patients with symptomatic knee OA (*)	Hill (2018) [55]
Obese patients with symptomatic knee OA should be referred to a specialist weight management service before orthopaedic assessment (*)	Hill (2018) [55]
Weight loss should be the first-line treatment in the management of symptomatic knee OA in obesity (*)	Hill (2018) [56]
Support for creation of regional centres where orthopaedic surgeons and bariatric surgeons, with their respective teams, could assess obese patients with symptomatic knee pain (*)	Hill (2018) [56]
Important to try non-surgical treatments first (*)	Hofstede (2016) [57]
Important to delay a surgery as long as possible (*)	Hofstede (2016) [57]
Conservative treatment is (perhaps) an important part of OA management (*)	Reid (2014) [59]
Positive attitude toward guidelines or protocols (facilitator)	
Perceived professional responsibility to adhere to evidence-based guideline	Allison (2019) [27]
Important to follow guidelines (*)	Hofstede (2016) [57]
In general, I do not/might not feel resistance towards working according to protocols (*)	De Rooij (2020) [60]
Autonomy (unclear factor)	
Autonomy affects referral considerations	Law (2019) [37]

Role

Description	Reference
Perception of own role potentially impeding prescription or follow-up of LIs (barrier)	
Referring patients to other health care providers and for other treatments rather than recommending exercise	Christiansen (2020) [29]
Referring patients to those with specialized knowledge rather than treating themselves (outside scope of practice)	Christiansen (2020) [29]
Paternalistic approach to care (low level of engagement in providing exercise and weight management advice)	Egerton (2018) [33]
Lack of confidence/uncertainty related to role in weight management	MacKay (2020) [42]
Perception that discussions related to diet were not part of their scope of practice	MacKay (2020) [42]
Changing own practice style remained as barrier after OA training	Miller (2020) [44]
Most GPs believed their contribution was essentially limited to diagnosis of condition and medication	Poitras (2010) [47]

For peer review only

Not focusing on increasing patients' motivation for behavioural change, but just giving general recommendations	Rosemann (2006) [48]
Mentioning benefits of weight reduction, but not actively coaching or referring patients	Selten (2017) [49]
Not perceiving weight reduction advice as their responsibility	Selten (2017) [49]
Uncertainty over scope of practice/questioning whether weight and pain management fall outside scope	Tang (2020) [50]
Describing own role as prepping patients for knee surgery when they were referred for physiotherapy	Teo (2020) [51]
Advice about how to lose weight was limited to brief general advice	Teo (2020) [51]
Considering weight loss to be outside own scope of practice (role of a dietician)	Teo (2020) [51]
Comfortable suggesting surgery to patients who responded poorly to conservative management	Teo (2020) [51]
GPs should (perhaps) not follow-up patients to monitor extent of continuation of exercises (*)	Cottrell (2016) [53]
It is the patient's own responsibility to continue doing their exercise programme (*)	Cottrell (2016) [53]
Not (or perhaps not) interested in being the orthopedic surgeon in an ortho-bariatric centre (*)	Hill (2018) [55]
Therapist's role seen as assessment/exercise prescription/education (relatively short-term responsibilities)	Holden (2009) [61]
Patient's role to follow prescribed exercise program over long term/get on board with treatment	Holden (2009) [61]
Physical therapists should (perhaps) not prescribe general exercise for every patient with knee OA (*)	Holden (2009) [61]
It is the patient's own responsibility to continue doing their exercise program (*)	Holden (2009) [61]
It is not/might not be the physiotherapist's responsibility to make sure that the patient will continue doing their exercise program (*)	Holden (2009) [61]
Negative consequences for own role when referring patients to LIs (barrier)	
Job satisfaction may be diminished when handing over care of their patients to third party with no involvement	Egerton (2017) [32]
The addition of a care support team may lead them feeling disconnected with their patient's care	Egerton (2017) [32]
Perception of own role potentially stimulating prescription or follow-up of LIs (facilitator)	
Patient-centred approach (high level of engagement in providing exercise and weight management advice)	Egerton (2018) [33]
Viewing themselves as having an important role in supporting clients to participate in management	MacKay (2018) [41]
Routinely including education about weight management	MacKay (2020) [42]
Playing a role in promoting engagement in management	MacKay (2020) [42]
Necessity of physiotherapy to effectively rehabilitate knee OA patients (because of knowledge/availability)	Poitras (2010) [47]
Importance of PT's role in educating patients with regards to NSAIDs/alternatives (including physiotherapy)	Poitras (2010) [47]
Necessity of PT involvement in managing activity (because potentially detrimental if excessive)	Poitras (2010) [47]
Exercise planning is usually PT's role (rather than GP's)	Poitras (2010) [47]
Necessity of PT follow-up sessions to assess and encourage patient adherence	Poitras (2010) [47]
PT's role to individualize patients' activity according to needs and capacity	Poitras (2010) [47]
Although agreeing with active patient participation, it is ultimately PT's role to appropriately manage patients	Poitras (2010) [47]
Desire to be more involved in life style counselling (upgrade of profession)	Rosemann (2006) [48]
Perceiving exercise prescription to be their main role	Teo (2020) [51]
Advising patients against surgery for as long as possible (last option)	Teo (2020) [51]
Implementing several strategies to boost adherence	Teo (2020) [51]
Not their role to advise the patient about knee surgery, opting not to discuss surgery at all	Teo (2020) [51]
Advising patients against knee arthroscopy if specifically asked about this procedure	Teo (2020) [51]

For peer review only

It is part of my job to reassure patients about the safety of exercise for CKP (*)	Cottrell (2016) [53]
GPs should educate patients with CKP about how to change their lifestyle for the better (*)	Cottrell (2016) [53]
GPs should prescribe quadriceps strengthening exercises to every patient with CKP (*)	Cottrell (2016) [53]
GPs should prescribe general exercise (e.g. walking or swimming) for every patient with CKP (*)	Cottrell (2016) [53]
There was a BMI threshold above which they would not perform a TKA at all (*)	Hill (2018) [55]
There was a BMI threshold above which they would not perform a TKA until the patient had attended a weight management program (*)	Hill (2018) [55]
Intention to refer patients to an ortho-bariatric centre if it existed (*)	Hill (2018) [56]
Recognizing potential influence on exercise adherence, sharing responsibility of exercise adherence with patient	Holden (2009) [61]
Physical therapists should prescribe local strengthening exercise for every patient with knee OA (*)	Holden (2009) [61]
Physiotherapists should educate chronic patients with knee OA about how to change their lifestyle for the better (*)	Holden (2009) [61]
Positive consequences for own role when referring patients to LIs (facilitator)	
Some appeal for a lessening of their own responsibility in terms of managing this condition	Egerton (2017) [32]

Domain 3: Patient factors

Health status

Description	Reference
Severity of disease and symptoms (barrier)	
Level of disease severity (i.e. whether people with very mild or very severe joint disease would benefit)	Egerton (2017) [32]
Patients delay care until they are highly symptomatic (missing opportunities to slow disease progression)	Miller (2020) [44]
Rehabilitation potential depended on length of disability (less potential with late management)	Poitras (2010) [47]
Paracetamol could mask pain/underlying physical problem (reducing opportunity to assess/manage problem)	Poitras (2010) [47]
Difficult to obtain effective analgesia with some patients	Poitras (2010) [47]
Potential to create unrealistic expectations and discouragement in patients that were too disabled	Poitras (2010) [47]
Questioning capacity to perform regular exercise because of severity of disability	Poitras (2010) [47]
Knee pain restricts activities in general (which makes weight loss difficult)	Poitras (2010) [47]
Pain (main barrier resulting in reduced dosage prescription of strengthening exercises)	Tang (2020) [50]
Patient's ability to exercise (main barrier resulting in reduced dosage prescription of strengthening exercises)	Tang (2020) [50]
Pain (key barrier to prescription of exercise as recommended by CPGs)	Tang (2020) [50]
Pain (barrier to prescription of aerobic exercise)	Tang (2020) [50]
Osteoarthritis severity (mild/severe) (patient-related barrier)	Wallis (2020) [52]
Effectiveness related to severity of joint damage/pain level (less effective when more damage/pain)	Holden (2009) [61]
Reluctant to promote exercise in the presence of pain	Holden (2009) [61]
Pain (as patient-centered barrier to adherence)	Holden (2009) [61]
Exercises are not/might not be effective for patients if an X-ray shows severe knee osteoarthritis (*)	Holden (2009) [61]
Negative impact of comorbidities (barrier)	

For peer review only

Difficulty with managing multiple conditions/tendency to prioritize other conditions over OA	Christiansen (2020) [29]
Significant impact of other health problems on patients' ability to commit fully to exercise program	Lawford (2020) [39]
Patient body weight (overweight/obese) (impedes exercise/makes visits to services more difficult)	Miller (2020) [44]
Questioning capacity to perform regular exercise because of general health	Poitras (2010) [47]
Depression as important barrier to motivate patients to physical exercise	Rosemann (2006) [48]
Comorbidities (often more severe pain, hampering ability to exercise or be physically active)	Teo (2020) [51]
Existing comorbidities (patient-related barrier)	Wallis (2020) [52]
Other patient characteristics (barrier)	
Person had been sedentary throughout life	Poitras (2010) [47]
Questioning capacity to perform regular exercise because of age	Poitras (2010) [47]
Patients with knee OA tended to be older/less active/with slower metabolism (which makes weight loss difficult)	Poitras (2010) [47]
Older age (patient-related barrier)	Wallis (2020) [52]
Poor rate of previous (physiotherapy) success (*)	Reid (2014) [59]
Severity of disease and symptoms (facilitator)	
Most patients tolerated a lot more than was expected (amount of exercise)	Lawford (2020) [39]
Rehabilitation potential depended on length of disability (better outcomes with early management)	Poitras (2010) [47]
Effective analgesia necessary for patients to be able to accomplish activities	Poitras (2010) [47]
Exercise is effective for patients if an X-ray shows severe knee OA (*)	Cottrell (2016) [53]
Referring patients to physiotherapy if they had high levels of pain/disability and where radiographic evidence of OA was present (*)	Reid (2014) [59]
Effectiveness related to severity of joint damage/pain level (more effective when less damage/pain)	Holden (2009) [61]
Exercises are effective for patients if an X-ray shows moderate knee osteoarthritis (*)	Holden (2009) [61]
Exercises are effective for patients if an X-ray shows mild knee osteoarthritis (*)	Holden (2009) [61]
Other patient characteristics (facilitator)	
Clients' pre-existing activity level (e.g. active person)	MacKay (2018) [41]
Importance of evaluating a patient's overall functional ability (rather than only knee signs/symptoms)	Teo (2020) [51]
Referring patients to physiotherapy if they were of a younger age (*)	Reid (2014) [59]
Severity of disease and symptoms (unclear factor)	
Treatment decisions depended on people's symptoms/findings of physical assessment	MacKay (2020) [42]
Management strategies depended on how bad the knee is	Okwera (2019) [46]
Exercise does not/might not work just as well for everybody, regardless of the amount of pain they have (*)	Cottrell (2016) [53]
Exercise does not/might not work just as well for everybody, regardless of the amount of pain they have (*)	Holden (2009) [61]
Other patient characteristics (unclear factor)	
Clients' general health	MacKay (2018) [41]

Treatment expectations and preferences

**Description**	**Reference**

For peer review only

Negative attitude toward LIs (barrier)	
Patients' lack of motivation to exercise/patients want passive treatment approach or quick fix	Christiansen (2020) [29]
Patients often have own ideas on management (problematic if primarily passive treatments)	Egerton (2018) [33]
Reluctance from patients to talk about physical activity (physical therapist's role, not the coach's role)	Hinman (2016) [34]
Interdisciplinary consult with dietician could not always take place because patients refused to visit dietician	Knoop (2020) [36]
People that don't particularly like exercise	Lawford (2020) [39]
Disconnect between PTs' recommendations for treatment and clients' expectations or preferences	MacKay (2018) [41]
Prior experiences with physical therapy influenced client expectations of clinical encounter	MacKay (2018) [41]
People's preferences were at odds with physical therapists' beliefs about management	MacKay (2020) [42]
Doubts about patients' willingness to make behavioral changes	Mann (2011) [43]
Patients don't want to expend effort towards lifestyle change	Miller (2020) [44]
Public expectation of what physical therapy treatment should be (e.g. didn't come to have thinking challenged)	Nielsen (2014) [45]
Feeling that patients tended to prefer treatment administered to them	Okwera (2019) [46]
Negative comments about patient reports of a lack of "hands-on" physiotherapy	Okwera (2019) [46]
Benefits obtained in the long term, which often conflicted with patient expectations for short-term benefits	Poitras (2010) [47]
Lack of patient motivation in remaining active despite knee OA	Poitras (2010) [47]
Patient views and expectations rarely matched patient needs	Poitras (2010) [47]
Questioning capacity to perform regular exercise because of motivation	Poitras (2010) [47]
Success rate in motivating patients too low (distinctly resignated regarding their impact on patients' life style)	Rosemann (2006) [48]
Frustration about impact of information (e.g. self-help groups) (lot of patients find excuses not to participate)	Rosemann (2006) [48]
Self-motivation (intrinsic barrier for patient adherence)	Teo (2020) [51]
Patient expectations (not keen to participate in exercise/play active role in management, desire for quick fix)	Teo (2020) [51]
Lack of motivation to participate active lifestyle interventions (patient-related barrier)	Wallis (2020) [52]
Existing relationships with physiotherapists (as barrier to referral if patient already had treating physiotherapist)	Wallis (2020) [52]
Exercise does not match patient needs/expectations (*)	Cottrell (2016) [53]
The patients are not/might not be cooperative in applying the protocol in daily clinical practice (*)	De Rooij (2020) [60]
Patients with knee OA and comorbidity are not always motivated to perform exercises	De Rooij (2020) [60]
Lack of motivation or laziness (as patient-centered barrier to adherence)	Holden (2009) [61]
Negative treatment expectations (as patient-centered barrier to adherence)	Holden (2009) [61]
Positive attitude toward TJA (barrier)	
Patients' ideas about whether they wanted surgery influenced making referrals to the LMP	Law (2019) [37]
Unrealistic expectations of the outcome of joint replacement among patients	Mann (2011) [43]
Feeling pressure by patients to refer them to specialist	Rosemann (2006) [48]
Make use of patients' preference for TJA within LIs (facilitator)	
Suggestion to relist patients completing the programme further up the waiting list (for surgery)	Law (2019) [37]
Using bargaining techniques centering on implications of LMP for replacement surgery (put patient on the list)	Law (2019) [37]
Patients' preferences (unclear factor)	

For peer review only

Management strategies depended on what the person wants	Okwera (2019) [46]

Active participation

Description	Reference
Low patient adherence or engagement (barrier)	
Shifting patients' mind-sets to active participation/making lifestyle changes was challenging/time consuming	Egerton (2018) [33]
How well they felt the individual patient would engage with programme influenced making referrals to the LMP	Law (2019) [37]
Challenging work to get clients to initiate management and maintain it over the long term	MacKay (2018) [41]
Getting buy-in (engaging people in management) often portrayed as challenge	MacKay (2020) [42]
Lack of compliance with home exercise regimes and advice given to patients was common	Okwera (2019) [46]
Patients' adherence to management recommendations was limited (because of fatalism)	Poitras (2010) [47]
Patients' unsatisfactory adherence to exercise programs	Teo (2020) [51]
Main concern was participant adherence to physical activity routines after end of program	Duarte (2019) [54]
Negative perceptions of patients' levels of exercise adherence	Holden (2009) [61]
High patient adherence or engagement (facilitator)	
Positive impact of information, education, and structured monitoring on patients' adherence to exercise	Hinman (2016) [34]
Patients adherent/easy to work with when they engaged in exercise program/started seeing improvements	Lawford (2020) [39]
Cohort of patients, in general, was highly motivated (remained interested/motivated for entirety of 6 months)	Lawford (2021) [40]
Enthusiastic participation of the participants	Duarte (2019) [54]
Importance of high patient adherence or engagement for effectiveness of LIs (facilitator)	
Exercise progression was most effective when the participant requested progression	Davis (2018) [30]
Client participation in management was critical to see improvement in symptoms	MacKay (2018) [41]
Getting buy-in (engaging people in management) critical to improving outcomes	MacKay (2020) [42]
Necessity of patients' active participation in knee OA management (to achieve significant outcomes)	Poitras (2010) [47]
How well a patient complies with their exercise programme determines how effective it will be (*)	Cottrell (2016) [53]
Importance of exercise adherence/link between level of adherence and clinical outcomes (dose-response effect)	Holden (2009) [61]
How well a patient complies with their exercise program determines how effective it will be (*)	Holden (2009) [61]

Capabilities

Description	Reference
Low health literacy (barrier)	
Poor health literacy in chronic disease management negatively influenced discussing exercise/weight management	Egerton (2018) [33]
Diagnosis can foster fear-avoidance (e.g. reduced activity) due to belief activity/exercise will cause further damage	Egerton (2018) [33]
Pessimistic about patients' abilities to make lifestyle changes to address their knee OA (not capable)	Egerton (2018) [33]
Lack of information and patient-professional discussion at point of referral may hinder uptake/retention of LMP	Law (2019) [37]

For peer review only

Patients were sceptical about safety and benefits of strengthening exercise for OA	Lawford (2020) [39]
Fear (patients required a lot of encouragement and reassurance)	Lawford (2020) [39]
Patients were apprehensive about managing weight by themselves	Lawford (2021) [40]
Some clients had misconceptions about OA (nothing they could do/normalising it as part of ageing)	MacKay (2018) [41]
Some clients feared participation in exercise (concerns for further degeneration)	MacKay (2018) [41]
Accepting diagnosis of OA could be particularly challenging for people with early OA	MacKay (2018) [41]
Clients' language (e.g. haven't mastered English/French)	MacKay (2018) [41]
Clients' lifestyle (e.g. coping, attitude towards pain)	MacKay (2018) [41]
Insufficient information for OA patients (e.g. not providing leaflets)	Mann (2011) [43]
Difficulty convincing patients to consider non-surgical, non-medication treatments	Miller (2020) [44]
Lack of patient self-efficacy (regarding lifestyle changes)	Miller (2020) [44]
Lack of knowledge about OA (patient barrier)	Miller (2020) [44]
Most patients demonstrated fatalism/inadequate knowledge and beliefs related to knee OA management	Poitras (2010) [47]
Ambivalent about patients' ability to lose weight (not able to succeed in making lifestyle changes)	Selten (2017) [49]
The belief that patients are not capable of losing weight	Selten (2017) [49]
Fear of falling (intrinsic barrier for patient adherence)	Teo (2020) [51]
Fear of pain (intrinsic barrier for patient adherence)	Teo (2020) [51]
Language/different cultural backgrounds (patient-related barrier)	Wallis (2020) [52]
Patients do not always believe in/may lack knowledge about effectiveness of exercise therapy	De Rooij (2020) [60]
Fear of harm (as patient-centered barrier to adherence)	Holden (2009) [61]
Limited financial resources (barrier)	
Clients' socioeconomic status (e.g. great poverty, shelter system)	MacKay (2018) [41]
Costs related to weight loss can be prohibitive for patients with limited resources (financial burdens)	Miller (2020) [44]
Other responsibilities (barrier)	
Clients' family responsibilities (e.g. busy, lot going on)	MacKay (2018) [41]
Work/other commitments precluding exercise-therapy (patient-related barrier)	Wallis (2020) [52]
High health literacy or importance of education (facilitator)	
Importance of pain education and reassurance about safety and benefits of exercise	Lawford (2020) [39]
Education contributed to buy-in to treatment (pathology, consequences, treatments)	MacKay (2020) [42]
Need for early education about OA/self-management and treatment options and opportunity to discuss these	Mann (2011) [43]
Ambivalent about patients' ability to lose weight (able)	Selten (2017) [49]
Education focused on self-management strategies	Teo (2020) [51]
Inform patients with knee OA and comorbidity better about benefits of exercise therapy	De Rooij (2020) [60]
Social support (facilitator)	
Level of support patients had from family/people close to them seemed to make a big difference	Lawford (2021) [40]
Integrating patients' social system into treatment	Rosemann (2006) [48]
Health literacy (unclear factor)	

Management strategies depended on what the person can cope with	Okwera (2019) [46]
Other responsibilities (unclear factor)	
Clients' occupation	MacKay (2018) [41]

Domain 4: Professional interactions

Collaboration

**Description**	**Reference**
Non-optimal interdisciplinary collaboration or healthcare provision (barrier)	
Cautious not to encroach on other HCPs' territory	Allison (2019) [27]
Potential for confusion about the treatment plan	Egerton (2017) [32]
Potential for issues resulting from incongruence of patient advice and information	Egerton (2017) [32]
Second professional not necessary to fulfill health coach role (part of own professional role as physical therapists)	Hinman (2016) [34]
Overlapping roles of physical therapist and coach could be source of conflict if not working from same set of goals	Hinman (2016) [34]
Necessary teamwork less likely if coach/physical therapist did not recognize/support each other's goals	Hinman (2016) [34]
Physicians who did not make timely referrals to physical therapy	MacKay (2018) [41]
Physicians' attitudes could influence clients' perceptions and level of buy-in to physical therapy	MacKay (2018) [41]
Lack of provision for patients who were not candidates for surgery (too long without help)	Mann (2011) [43]
Patients lacked proactive follow-up to support self-management	Mann (2011) [43]
Lack of coordination between leisure, social and health services	Mann (2011) [43]
Insufficient (physiotherapy) intervention when patients were seen	Mann (2011) [43]
Belief that physiotherapists did not find it rewarding/interesting to treat OA patients	Mann (2011) [43]
Criticizing the decision to centralize musculoskeletal physiotherapy service (useful to have somebody in team)	Okwera (2019) [46]
Frustrations about lack of continuity regarding team of physiotherapists within clinic	Okwera (2019) [46]
Not working closely with physiotherapists/frustrations about working relationship	Okwera (2019) [46]
Dissatisfaction about loss of coherent working since centralizing musculoskeletal physiotherapy service	Okwera (2019) [46]
Disagreement on effective exercise parameters	Poitras (2010) [47]
Disagreement on optimal design of exercise programs to increase adherence	Poitras (2010) [47]
Mistrust in interventions dieticians use to help patients' with weight reduction attempts	Selten (2017) [49]
Negative views about physical therapists who provided non-evidence-based treatments	Selten (2017) [49]
Mistrust because they observed huge differences in quality of care delivered by physical therapists	Selten (2017) [49]
Occupational therapists, podiatrists and physical therapists do not work together optimally in OA care	Selten (2017) [49]
Role of rheumatologist in knee/hip OA care perceived as unclear/limited	Selten (2017) [49]
Agreement that orthopedic surgeon's primary task is to assess whether patient is eligible for surgery	Selten (2017) [49]
Orthopedic surgeons were perceived negatively by several healthcare providers	Selten (2017) [49]
Unclear what physio offers (*)	Cottrell (2016) [53]

For peer review only

My colleagues in physiotherapy are not/might not be cooperative in applying the protocol in daily clinical practice (*)	De Rooij (2020) [60]
The general practitioners or other physicians are not/might not be collaborative regarding the application of the protocol in daily clinical practice (*)	De Rooij (2020) [60]
Suboptimal collaboration with general practitioners and physicians	De Rooij (2020) [60]
Referring physicians do not always believe in/may lack knowledge about effectiveness of exercise therapy	De Rooij (2020) [60]
No access to other HCPs (barrier)	
Lack of access to other healthcare providers (e.g. physicians with expertise in OA)	MacKay (2018) [41]
Good interdisciplinary collaboration or healthcare provision, or suggestions for improvement (facilitator)	
Perceived status of physical therapists within health care team and wider community	Allison (2019) [27]
Importance of clearly understanding roles and functions of service, care support team, and themselves	Egerton (2017) [32]
Importance of having confidence in (the skills of) the staff of a new service to deliver on promises	Egerton (2017) [32]
Having a personal relationship with the people providing the service/a desire to work closely with service staff	Egerton (2017) [32]
Appreciation how their participation afforded physical therapists and coaches opportunities to collaborate	Hinman (2016) [34]
Importance of teamwork in delivering the integrated intervention	Hinman (2016) [34]
Reinforcement of health messages from another clinician could be valuable	Hinman (2016) [34]
Having a separate coach freed therapists up to focus on other treatment aspects	Hinman (2016) [34]
Multidisciplinary nature of LMP (ability to utilize expertise from other professionals)	Law (2019) [37]
Importance of good working relationships	MacKay (2018) [41]
Physicians who expressed support for physical therapy/exercise and referred clients to physical therapy early	MacKay (2018) [41]
Patients would be better served by long-term condition model of care (e.g. diabetes mellitus)	Mann (2011) [43]
Patients should initiate own follow-up when needed (as better use of time/health care resources)	Mann (2011) [43]
Allow patients, after initial referral, to use direct access system to service (no need for re-referral)	Mann (2011) [43]
Utilising clinic health educator who met with patients for weight loss discussions and followed up by phone	Miller (2020) [44]
Employing a multi-pronged approach to engage patients in weight loss	Miller (2020) [44]
Overall positive experience of physiotherapy service and therapists	Okwera (2019) [46]
GPs believed PT involvement was necessary to motivate the patient and manage the exercise program	Poitras (2010) [47]
Gate keeper role for GPs could reduce patients' pressure to refer to orthopaedics/decrease performed x-rays	Rosemann (2006) [48]
Involvement of practice nurses is imaginable in the area of life style counselling and advice giving	Rosemann (2006) [48]
Dieticians are helpful for patients trying to lose weight	Selten (2017) [49]
Need for physical therapists to provide evidence-based exercises instead of non-evidence-based modalities	Selten (2017) [49]
Non-pharmacological, non-surgical OA care can and should be provided in a primary care setting	Selten (2017) [49]
GPs have coordinating role (diagnose/monitor, refer when necessary, lifestyle education, long-term coach)	Selten (2017) [49]
Physical therapists can guide patients/provide lifestyle advice (more time compared with GPs)	Selten (2017) [49]
Perceiving rheumatologists' role as valuable (giving injections, providing lifestyle/medication advice, refer)	Selten (2017) [49]
Knowledge that program was delivered by well-trained and trusted physiotherapist	Wallis (2020) [52]
Exercise for CKP is more effectively provided by physiotherapists than GPs (*)	Cottrell (2016) [53]
Obese patients with symptomatic knee OA should be assessed by a specialist multidisciplinary service, which should include an orthopaedic surgeon (*)	Hill (2018) [55]

For peer review only

Support for creation of regional centres where orthopedic surgeons and bariatric surgeons, with their respective teams, could assess obese patients with symptomatic knee pain (*)	Hill (2018) [55]
Obese patients with symptomatic knee OA should be referred to a specialist weight management service before orthopaedic assessment (*)	Hill (2018) [56]
Obese patients with symptomatic knee OA should be assessed by a specialist multidisciplinary service (*)	Hill (2018) [56]
Agreements with colleagues about the content of the care trajectory (*)	Hofstede (2016) [57]
Positive attitudes of colleagues about non-surgical treatments (*)	Hofstede (2016) [57]
Agreements/ deliberations with primary care (GP, physical therapist, dietician) (*)	Hofstede (2016) [57]
Physiotherapists do not/might not lack expertise in OA management (*)	Reid (2014) [59]
Working with the protocol invites me to discuss more with experts in the field of the comorbidity (*)	De Rooij (2020) [60]
Inform referrers better about benefits of exercise therapy in patients with knee OA and comorbidity	De Rooij (2020) [60]
Optimize collaboration with orthopaedic surgeons and other health care providers	De Rooij (2020) [60]
Support from colleagues	Kloek (2020) [62]
Access to other HCPs (facilitator)	
Potential benefit of increased access to OA specialists	Egerton (2017) [32]
Access to a team on-site/a network of healthcare providers they trusted	MacKay (2018) [41]
Those with access to other clinicians recommended to consult another clinician for advice on diet as needed	MacKay (2020) [42]
Care could be improved if every GP practice contained an individual who took a particular interest in OA	Mann (2011) [43]
There should be OA specialist clinicians (all relevant allied health professions) providing services in community	Mann (2011) [43]
In-house physiotherapy (as suggestion for physiotherapy service improvement)	Okwera (2019) [46]

Communication and referral

Description	Reference
Lack of communication between HCPs (barrier)	
Frustrations about lack of contact/communication involved in the referral and discharge process	Okwera (2019) [46]
Dissatisfaction about loss of communication since centralizing musculoskeletal physiotherapy service	Okwera (2019) [46]
Specialist did not take time to explain what they had examined/x-rays he had taken	Rosemann (2006) [48]
Physicians are not always collaborating in discussing medical conditions of patients	De Rooij (2020) [60]
Challenges of communication and referral procedures (barrier)	
Necessary teamwork less likely if communication processes not clearly prescribed/structure not used	Hinman (2016) [34]
Different views were expressed about the preferred medium of communication	Hinman (2016) [34]
Interdisciplinary consult with dietician could not always take place because of problems with contacting dietician	Knoop (2020) [36]
Challenges when coordinating multimodal care (including difficulties with the referral system)	Miller (2020) [44]
Frustrations about restrictive referral pathways	Okwera (2019) [46]
Referral process was convoluted and at times irrelevant	Okwera (2019) [46]
Requesting medical information about patients from specialists takes a lot of time	De Rooij (2020) [60]
Improving communication between HCPs (facilitator)	

For peer review only

GPs wanted to be updated on the advice given and plan made so they know what has been said to their patient	Egerton (2017) [32]
Communication needed to be collaborative, patient-centered and consistent for integrated care to be effective	Hinman (2016) [34]
Need for improving communication (quality of referrals, information at discharge)	Okwera (2019) [46]
Better communication with specialists could increase efficacy of treatment	Rosemann (2006) [48]
Receiving communication back from program physiotherapist about patient outcomes	Wallis (2020) [52]
Clarity on what the patient has done at the physical therapist (*)	Hofstede (2016) [57]
Needs regarding communication and referral procedures (facilitator)	
Need to ensure referral procedures are streamlined in order to minimize impact on their busy schedules	Egerton (2017) [32]
Need for effective, useful and timely channels of communication between the GP and the care support team	Egerton (2017) [32]
Importance of effective mechanisms to communicate	MacKay (2018) [41]
Streamlining the physiotherapy referral process (as suggestion for physiotherapy service improvement)	Okwera (2019) [46]
Straightforward, easy and quick lines of communication among different disciplines in healthcare center	Selten (2017) [49]
Simple, streamlined referral process (suggestion for promotion and referrals)	Wallis (2020) [52]

Domain 5: Incentives and resources

Time

**Description**	**Reference**
Lack of time within patient consultations (barrier)	
Time pressure (unable to individualise weight management/develop exercise plans within appointment time)	Egerton (2018) [33]
Restricted in amount of time they could allot per patient	MacKay (2018) [41]
Lack of time to give patients sufficient opportunity to discuss their condition	Mann (2011) [43]
Lifestyle counseling is huge time commitment	Miller (2020) [44]
Appointment times too short to address all of patient's issues and provide lifestyle counseling	Miller (2020) [44]
Time required to teach PCST skills to patients	Nielsen (2014) [45]
Weight reduction advice takes too much time in a consultation	Selten (2017) [49]
Insufficient time in consultations (*)	Cottrell (2016) [53]
Time constraints prevent GPs from providing advice on individual exercises for CKP (*)	Cottrell (2016) [53]
Limited time to review individual patients reduced opportunities to facilitate behavior change	Holden (2009) [61]
Large caseloads and pressure of waiting lists reduced the number of treatment sessions provided	Holden (2009) [61]
Limited opportunity to provide follow-up sessions after discharge	Holden (2009) [61]
Lack of time due to other demands (or not further specified) (barrier)	
The addition of a care support team may increase paperwork	Egerton (2017) [32]
Lack of time to monitor attendance/provide support was compounded by increasing administrative demands	Law (2019) [37]
Wait lists as a burden	MacKay (2018) [41]
Lack of time	Rosemann (2006) [48]

For peer review only

Less satisfied about time needed to get used to e-Exercise during high work pressure/administrative burden	Kloek (2020) [62]
Perceiving web-based application as an additional burden	Kloek (2020) [62]
Busy work schedules and administrative burden hindered testing/using e-Exercise in their practice	Kloek (2020) [62]
Adequate duration of patient consultations (facilitator)	
Importance of longer consultations	Egerton (2018) [33]
Having adequate time to spend with clients	MacKay (2018) [41]
Adequate duration of specific interventions or protocols (facilitator)	
Idea of having some of the burden of managing this patient group (e.g. time) taken away appealing	Egerton (2017) [32]
Incorporating selected PCST components on as-needed basis most practical way within current environment	Nielsen (2014) [45]
Working according to the protocol is not/might not be too time-consuming (*)	De Rooij (2020) [60]
Perceiving web-based application as time-saving	Kloek (2020) [62]
I do not/might not have insufficient time available to get familiar with e-Exercise and to use the web-application (*)	Kloek (2020) [62]

Financial resources

Description	Reference
Limited financial resources within organization (barrier)	
Concern about capacity to recover costs of incorporating CBT into practice	Nielsen (2014) [45]
Treatments (e.g. physiotherapy) prescribed less frequently due to decreasing financial resources	Rosemann (2006) [48]
Financial reward for implementing LIs (facilitator)	
Financial incentivisation	Egerton (2017) [32]
Payment system has to be changed to upgrade conservative treatments and conversation with patient	Rosemann (2006) [48]
Interventions performed by practice nurses have to be reinsured sufficiently	Rosemann (2006) [48]
Working according to the protocol should be financially rewarded (*)	De Rooij (2020) [60]

Information resources

Description	Reference
Lack of information resources (barrier)	
Absence of clear guidelines for weight loss	Allison (2019) [27]
Lack of information about scheme hindered referral	Law (2019) [37]
Lack of resources for face-to-face patient education and patient reference	Miller (2020) [44]
Lack of information about self-help groups/offers on community level	Rosemann (2006) [48]
Missing information about offers e.g. in the community	Rosemann (2006) [48]
Challenges in accessing information resources (barrier)	
Challenges in accessing scientific papers	MacKay (2018) [41]
Difficulty finding high quality, patient-friendly OA educational materials	Miller (2020) [44]

For peer review only - http://bmjopen.bmj.com/site/about/guidelines.xhtml

Frustration that material found on Internet or provided by friends/family was frequently inaccurate	Miller (2020) [44]
Cannot access necessary resources (*)	Cottrell (2016) [53]
Availability of information resources (facilitator)	
Clear preference for concrete guidelines or tools for engaging in weight management	Allison (2019) [27]
Recommending informational materials for patients (to mitigate delays in OA care)	Miller (2020) [44]
Standardised flowsheet on OA management (as guide for providers/tool for patient discussions)	Miller (2020) [44]
Specific information about program (suggestion for promotion and referrals)	Wallis (2020) [52]
Clear referral criteria/guideline (*)	Hofstede (2016) [57]
Access to information resources (facilitator)	
Having access to customizable, printable patient resources	Egerton (2018) [33]
Access to current evidence	MacKay (2018) [41]
Professional networks/community of practice as mechanism to facilitate sharing of information	MacKay (2018) [41]
Integrating scientific evidence from studies into their approach to management	MacKay (2020) [42]
Including links on websites of partners (suggestion for promotion and referrals)	Wallis (2020) [52]

Facilities

**Description**	**Reference**
Negative attitude toward information technology (barrier)	
Sceptical about benefit of clinical practice information technology	Egerton (2018) [33]
Potential use of information technology (facilitator)	
Changes to clinical practice information technology (e.g. prompts into clinic software)	Egerton (2018) [33]
Having patient resources embedded within current practice software or routines	Egerton (2018) [33]
Electronic reminders for physicians on how to locate OA treatment information and resources	Miller (2020) [44]
Benefits of working in health centers (facilitator)	
Collaboration among multiple disciplines could be facilitated by working in a health center	Selten (2017) [49]

Domain 6: Capacity for organizational change

Professional paradigm

**Description**	**Reference**
Adequate professional paradigm or suggestions for expansion (facilitator)	
Nature of the physical therapy paradigm (in relation to weight management)	Allison (2019) [27]
Physical therapy scope of practice was adequate to manage clients with perceived early knee OA	MacKay (2018) [41]
Suggestion that it would be useful to expand scope of practice to include ordering diagnostic imaging	MacKay (2018) [41]
Value of increasing profession's explicit understanding/use of PCST skills (practice model may be required)	Nielsen (2014) [45]

Value of incorporating aspects of PCST mind-set into professional training (entry-level vs. postgraduate level)	Nielsen (2014) [45]

Monitoring

**Description**	**Reference**
Audit (facilitator)	
Peer review/audit of professional association (*)	Hofstede (2016) [57]

Support within the organization

**Description**	**Reference**
Management not supportive (barrier)	
The management of my practice is not/might not be collaborative regarding the application of the protocol in daily clinical practice (*)	De Rooij (2020) [60]

Domain 7: Social, political, and legal factors

Healthcare system

**Description**	**Reference**
Restrictions due to health insurance (barrier)	
Lack of funding prevented clients from accessing services/seeking help/getting full course of treatment	MacKay (2018) [41]
Treatments (e.g. physiotherapy) prescribed less frequently due to increasing restrictions by health insurances	Rosemann (2006) [48]
The number of treatments that the patient receives from their insurance company is a barrier in using the protocol (*)	De Rooij (2020) [60]
Number of treatment sessions patients receive from insurance companies restricted application of the strategy	De Rooij (2020) [60]
Benefits of good health insurance (facilitator)	
Patients who are well insured have improved access to services (e.g. physical therapy)	Miller (2020) [44]
Positivity toward private sector (patients will get seen a lot quicker)	Okwera (2019) [46]
Private healthcare supplementation (as suggestion for physiotherapy service improvement)	Okwera (2019) [46]
In complex patients insurance companies should reimburse more treatment sessions	De Rooij (2020) [60]
Government subsidies (facilitator)	
Government-subsidised allied health visits to facilitate utilisation of services that support exercise/weight loss	Egerton (2018) [33]

Domain 8: Patient and HCP interactions

Therapeutic alliance

**Description**	**Reference**

For peer review only - http://bmjopen.bmj.com/site/about/guidelines.xhtml

Potential negative influence of implementing LIs to relationship (barrier)	
Feelings of guilt when referring to LMP (dooming patients to a longer wait for surgery)	Law (2019) [37]
Importance of communication and relationship (facilitator)	
Strong therapeutic relationship with patients	Lawford (2020) [39]
Having positive attitude/being encouraging of small changes/being hopeful about OA management	MacKay (2020) [42]
More openly address psychological complaints of patients	Rosemann (2006) [48]
Good communication with patient may help in delaying surgery	Selten (2017) [49]
Importance of having trust	Selten (2017) [49]

Lifestyle as conversation topic

**Description**	**Reference**
Challenges of discussing weight (barrier)	
Perceived impact of own weight during weight discussions (not being overweight)	Allison (2019) [27]
Apparent discomfort with having conversations about weight	Allison (2019) [27]
Concern about how weight conversations might threaten patient rapport	Allison (2019) [27]
Weight loss is sensitive topic (afraid of upsetting their patients results in temptation to avoid discussion)	Egerton (2018) [33]
Weight was touchy/sensitive subject to discuss	MacKay (2020) [42]
Difficulties in communicating with patients about being overweight	Selten (2017) [49]
Viewing weight as sensitive subject/feeling uncomfortable discussing it	Tang (2020) [50]
Factors that could ease the way to discussing weight (facilitator)	
Perceived impact of own weight during weight discussions (being overweight)	Allison (2019) [27]
Feeling comfortable discussing role of physical activity in maintaining weight control	MacKay (2020) [42]
Developing rapport with people made it easier to discuss weight management	MacKay (2020) [42]
Reframe discussions around exercise and weight loss (e.g. not blaming/discouraging people)	Miller (2020) [44]
Relationship with patients, developed through numerous sessions, facilitated influence for lifestyle modifications	Poitras (2010) [47]
Having a relationship with patient built on mutual trust/respect would ease way to discussing weight reduction	Selten (2017) [49]

Domain 9: Disease factors

Image

**Description**	**Reference**
OA seen as low priority (barrier)	
Assigning low priority to OA as disease	Christiansen (2020) [29]
Concern about providing this service for a condition perceived as low priority	Egerton (2017) [32]
OA was not given enough attention, symptoms were often dismissed/minimized in health care	Mann (2011) [43]

Medical professionals saw OA as low priority with respect to managing their workload	Okwera (2019) [46]
Knee OA more often diagnosed as an unanticipated comorbidity (rarely primary reason for consultation)	Poitras (2010) [47]
Not enough emphasis put on primary prevention of knee OA	Poitras (2010) [47]
Knee OA management seen as unchallenging routine	Poitras (2010) [47]
Belief that nobody is willing to change lifestyle due to OA, disease has to be a lot worse	Rosemann (2006) [48]
OA seen as untreatable and local condition (wear-and-tear) (barrier)	
Describing OA as simply a problem of cartilage degeneration/joint space narrowing (on x-ray)/wear and tear	Egerton (2018) [33]
Belief that symptoms will progress, and that surgery is inevitable	Egerton (2018) [33]
Assumption that patients would have negative connotations associated with the label knee OA	Egerton (2018) [33]
No effective treatment options	Miller (2020) [44]
Incurable nature and negative prognosis of OA	Okwera (2019) [46]
Knee OA seen as uninteresting health problem on which they had limited impact and could not cure	Poitras (2010) [47]
Knee OA was perceived as a degenerative (wear and tear)	Teo (2020) [51]
Using negative language to describe OA (wear-and-tear/joint damage/bone-on-bone/degenerative condition)	Wallis (2020) [52]
Biomedical perspective on knee OA, attributing signs and symptoms to local knee pathology or wear and tear	Holden (2009) [61]
OA seen as chronic degenerative condition that would progressively worsen over time (only cure being surgery)	Holden (2009) [61]
Optimistic views toward OA (facilitator)	
Belief that knee OA is condition that can be successfully managed	Egerton (2018) [33]
Importance of conveying to patients that diagnosis is not all negative/delivering a relatively positive prognosis	Egerton (2018) [33]
Knee OA seen as technically challenging condition	Poitras (2010) [47]

For peer review only

Supplemental File 4. Full overview of all extracted factors per article

Explanation

- In the tables below, all extracted factors per included article are presented. The number in brackets ('[…]') displayed after each article corresponds to the reference number used in the main text of the manuscript.
- Column "Number": the capital letter and color used refer to barriers (B/red), facilitators (F/green) or unclear factors (U/orange).
- Column "Description": (*) at the end of the description indicates that the factor is derived from a close-ended question or attitude statement.
- Column "Subcategory (domain)": the relevant subcategory is displayed first, followed by the number of the domain to which this subcategory belongs. The domain numbers refer to the domains as described in the main text of the manuscript: (1) intervention factors; (2) individual HCP factors; (3) patient factors; (4) professional interactions; (5) incentives and resources; (6) capacity for organizational change; (7) social, political, and legal factors; (8) patient and HCP interactions; (9) disease factors.
- Abbreviations: ACSM: American College of Sports Medicine; BMI: body mass index; CBT: cognitive behavioral therapy; CKP: chronic knee pain; CPG: clinical practice guideline; GP: general practitioner; HCP: healthcare professional; LI: lifestyle intervention; LMP: Lifestyle Management Programme; NSAID: non-steroidal anti-inflammatory drug; NWBE: non-weight bearing quadriceps strengthening exercise; OA: osteoarthritis; PCST: pain coping skills training; PT: physiotherapist; RCT: randomized controlled trial; TKA: total knee arthroplasty; TJA: total joint arthroplasty; WBE: weight bearing functional exercise.

Allison (2019) [27]

Number	Description	Subcategory (domain)
B1	Cautious not to encroach on other HCPs' territory	Non-optimal interdisciplinary collaboration or healthcare provision (4)
B2	Lack of knowledge around appropriate interventions for weight loss	Lack of knowledge or skills around LIs or promoting behavioral change (2)
B3	Uncertainty about how to enact their understanding of relationship between weight and knee OA	Lack of knowledge or skills around LIs or promoting behavioral change (2)
B4	Absence of clear guidelines for weight loss	Lack of information resources (5)
B5	Perceived impact of own weight during weight discussions (not being overweight)	Challenges of discussing weight (8)
B6	Apparent discomfort with having conversations about weight	Challenges of discussing weight (8)
B7	Concern about how weight conversations might threaten patient rapport	Challenges of discussing weight (8)
F1	Nature of the physical therapy paradigm (in relation to weight management)	Adequate professional paradigm or suggestions for expansion (6)
F2	Perceived status of physical therapists within health care team and wider community	Good interdisciplinary collaboration or healthcare provision, or suggestions for improvement (4)
F3	Perceived professional responsibility to adhere to evidence-based guideline	Positive attitude toward guidelines or protocols (2)
F4	Clear preference for concrete guidelines or tools for engaging in weight management	Availability of information resources (5)

F5	Perceived impact of own weight during weight discussions (being overweight)	Factors that could ease the way to discussing weight (8)

Bossen (2016) [28]

Number	Description	Subcategory (domain)
B1	Lack of financial incentive if blended intervention substitutes conventional visits (reduced venues per patient)	Other challenges for HCPs regarding feasibility of telehealth (1)
B2	Most of the patients prefer traditional face-to-face treatments	Patient-related challenges regarding feasibility of telehealth (1)
B3	Most of the patients did not meet study inclusion criteria	LIs are unavailable or inaccessible (1)
B4	e-Exercise must be adapted for suitable integration into practice (e.g. no insight into modules patients receive)	Challenges for HCPs during delivery of LIs (1)
F1	24/7 availability of information and exercises	Patient-related benefits regarding feasibility of telehealth (1)
F2	Possibility to extend physical therapy treatment in patient's home environment	Benefits of telehealth in terms of effectiveness (1)
F3	Potential to enhance the adherence of home exercises	Benefits of telehealth in terms of effectiveness (1)
F4	Positive feedback regarding the content of e-Exercise	Positive experiences with or suggestions to improve the content or structure of LIs (1)

Christiansen (2020) [29]

Number	Description	Subcategory (domain)
B1	Assigning low priority to OA as disease	OA seen as low priority (9)
B2	Assigning low priority to exercise as treatment	Negative attitude toward LIs (2)
B3	Difficulty with managing multiple conditions/tendency to prioritize other conditions over OA	Negative impact of comorbidities (3)
B4	Not certain that exercise works	LIs have little or no effect on OA (1)
B5	Referring patients to other health care providers and for other treatments rather than recommending exercise	Perception of own role potentially impeding prescription or follow-up of LIs (2)
B6	Limited knowledge of exercise prescription (uncertainty of what exercise to recommend/how much)	Lack of knowledge or skills around LIs or promoting behavioral change (2)
B7	Referring patients to those with specialized knowledge rather than treating themselves (outside scope of practice)	Perception of own role potentially impeding prescription or follow-up of LIs (2)
B8	Not received sufficient training on exercise/lack of education	Lack of knowledge or skills around LIs or promoting behavioral change (2)
B9	Patients' lack of motivation to exercise/patients want passive treatment approach or quick fix	Negative attitude toward LIs (3)

Davis (2018) [30]

Number	Description	Subcategory (domain)

For peer review only

B1	Class required intense supervision, which was difficult to provide when most participants were new	Challenges for HCPs during delivery of LIs (1)
B2	Challenges of supervision when space did not allow clear line of sight	Challenges for HCPs during delivery of LIs (1)
F1	Enthusiastic about the program and described the results (e.g. it was empowering)	LIs have positive mental effects (1)
F2	Initial classes needed to be small with rolling recruitment very beneficial	Ease for HCPs during delivery of LIs (1)
F3	First education session was critical to reducing the participant's anxiety related to exercising	Positive experiences with or suggestions to improve the content or structure of LIs (1)
F4	Importance of empowering the patients rather than 'pushing' them, achieved by 'giving choices'	Positive experiences with or suggestions to improve the content or structure of LIs (1)
F5	Exercise progression was most effective when the participant requested progression	Importance of high patient adherence or engagement for effectiveness of LIs (3)

De Rooij (2014) [31]

Number	Description	Subcategory (domain)
F1	Protocols offered guidance in setting up treatment/making clinical decisions/adapting treatment to comorbidity	Available resources might improve knowledge and decision-making (2)
F2	List of restrictions for exercise therapy was conveniently arranged checklist for diagnostic and treatment phases	Ease for HCPs during delivery of LIs (1)
F3	List of restrictions was helpful in process of clinical decision making	Available resources might improve knowledge and decision-making (2)
F4	Suggestion to increase feasibility by reducing the protocols to three main protocols	Ease for HCPs during delivery of LIs (1)
F5	Less afraid to increase training intensity (preventing adverse events by tailoring programs to individual's capacity)	Research environment or protocols provide a safety net (1)

Egerton (2017) [32]

Number	Description	Subcategory (domain)
B1	Concern that uptake would be negatively impacted if patients were required to pay	Costs of LIs to patients (1)
B2	Concern for overcomplicated system when service is not compatible/complementary with existing initiatives	LIs are not feasible or sustainable (1)
B3	Not seeing need (already adequate skills/resources to support OA patient self-management and lifestyle change)	LIs are not feasible or sustainable (1)
B4	Concern about providing this service for a condition perceived as low priority	OA seen as low priority (9)
B5	Not seeing need (advice already given at their practice would be unhelpfully repeated)	LIs are not feasible or sustainable (1)
B6	Remote (telephone) delivery is not as good as face-to-face particularly in relation to exercise advice	Disadvantages of telehealth in terms of effectiveness (1)
B7	Advice to exercise and lose weight does not work	LIs have little or no effect on OA (1)

For peer review only

B8	Hesitancy to embrace an unfamiliar new service	Other challenges for HCPs regarding feasibility of telehealth (1)
B9	Concern regarding long-term service sustainability	LIs are not feasible or sustainable (1)
B10	Concerns about security of patient data and information confidentiality during the referral process	Telehealth is not safe for patients or patient/data privacy (1)
B11	Job satisfaction may be diminished when handing over care of their patients to third party with no involvement	Negative consequences for own role when referring patients to LIs (2)
B12	The addition of a care support team may add complexities to management	LIs are not feasible or sustainable (1)
B13	The addition of a care support team may increase paperwork	Lack of time due to other demands (or not further specified) (5)
B14	The addition of a care support team may lead them feeling disconnected with their patient's care	Negative consequences for own role when referring patients to LIs (2)
B15	Potential for confusion about the treatment plan	Non-optimal interdisciplinary collaboration or healthcare provision (4)
B16	Potential for issues resulting from incongruence of patient advice and information	Non-optimal interdisciplinary collaboration or healthcare provision (4)
B17	Concerned the service would not be able to provide individualized management for a very diverse population	Insufficient ability to provide personalized treatment within LIs (1)
B18	Hearing and cognitive difficulties as barriers for some patients to being able to interact with the service	Patient-related challenges regarding feasibility of telehealth (1)
B19	Level of disease severity (i.e. whether people with very mild or very severe joint disease would benefit)	Severity of disease and symptoms (3)
B20	Inability of a remote service to provide locally relevant information	Disadvantages of telehealth in terms of effectiveness (1)
B21	Skepticism about whether many patients would embrace such a model (i.e. because of remote-delivery aspect)	Patient-related challenges regarding feasibility of telehealth (1)
F1	More likely to engage with the care support team if it enabled more affordable/accessible allied health	LIs are available or accessible, or suggestions for improvement (1)
F2	Need to ensure referral procedures are streamlined in order to minimize impact on their busy schedules	Needs regarding communication and referral procedures (4)
F3	Need for effective, useful and timely channels of communication between the GP and the care support team	Needs regarding communication and referral procedures (4)
F4	GPs wanted to be updated on the advice given and plan made so they know what has been said to their patient	Improving communication between HCPs (4)
F5	Need for clarity about how the new service would integrate with existing schemes and payment structures	LIs are feasible or sustainable (1)
F6	Seeing need (advice/recommendations may need to be reinforced/provided over several health care episodes)	LIs are feasible or sustainable (1)
F7	Seeing need (extra time and encouragement for the patient would result in better outcomes)	LIs are feasible or sustainable (1)
F8	Potential benefit of increased access to OA specialists	Access to other HCPs (4)
F9	Importance of clearly understanding roles and functions of service, care support team, and themselves	Good interdisciplinary collaboration or healthcare provision, or suggestions for improvement (4)

For peer review only

F10	Importance of broad acceptance (patients/doctors/health service funders) if new service is to continue long term	LIs are feasible or sustainable (1)
F11	Importance of having confidence in (the skills of) the staff of a new service to deliver on promises	Good interdisciplinary collaboration or healthcare provision, or suggestions for improvement (4)
F12	Having a personal relationship with the people providing the service/a desire to work closely with service staff	Good interdisciplinary collaboration or healthcare provision, or suggestions for improvement (4)
F13	Idea of having some of the burden of managing this patient group (e.g. time) taken away appealing	Adequate duration of specific interventions or protocols (5)
F14	Some appeal for a lessening of their own responsibility in terms of managing this condition	Positive consequences for own role when referring patients to LIs (2)
F15	Service could increase access to support for rural patients	Patient-related benefits regarding feasibility of telehealth (1)
F16	Financial incentivisation	Financial reward for implementing LIs (5)

Egerton (2018) [33]

Number	Description	Subcategory (domain)
B1	Describing OA as simply a problem of cartilage degeneration/joint space narrowing (on x-ray)/wear and tear	OA seen as untreatable and local condition (wear-and-tear) (9)
B2	Belief that symptoms will progress, and that surgery is inevitable	OA seen as untreatable and local condition (wear-and-tear) (9)
B3	Knowledge of exercise and weight-loss treatments is sometimes inaccurate or inadequate	Lack of knowledge or skills around LIs or promoting behavioral change (2)
B4	Dubious about effect of exercise and weight-management advice on reducing symptoms	LIs have little or no effect on OA (1)
B5	Reduced confidence with providing suitable exercise and weight loss advice	Lack of knowledge or skills around LIs or promoting behavioral change (2)
B6	Lack of skills in promoting readiness and motivation for lifestyle treatments	Lack of knowledge or skills around LIs or promoting behavioral change (2)
B7	Time pressure (unable to individualise weight management/develop exercise plans within appointment time)	Lack of time within patient consultations (5)
B8	Concerns regarding financial cost to patients when considering referral to other services	Costs of LIs to patients (1)
B9	Lack of availability of support services (e.g. community-based rehabilitation programs) in remote locations	LIs are unavailable or inaccessible (1)
B10	Long waiting lists for support services (e.g. community-based rehabilitation programs)	LIs are unavailable or inaccessible (1)
B11	Sceptical about benefit of clinical practice information technology	Negative attitude toward information technology (5)
B12	The issue is not a lack of suitable patient resources but awareness of them	Lack of knowledge or skills around specific resources (2)
B13	Poor health literacy in chronic disease management negatively influenced discussing exercise/weight management	Low health literacy (3)
B14	Patients often have own ideas on management (problematic if primarily passive treatments)	Negative attitude toward LIs (3)

For peer review only - http://bmjopen.bmj.com/site/about/guidelines.xhtml

B15	Shifting patients' mind-sets to active participation/making lifestyle changes was challenging/time consuming	Low patient adherence or engagement (3)
B16	Paternalistic approach to care (low level of engagement in providing exercise and weight management advice)	Perception of own role potentially impeding prescription or follow-up of LIs (2)
B17	Assumption that patients would have negative connotations associated with the label knee OA	OA seen as untreatable and local condition (wear-and-tear) (9)
B18	Diagnosis can foster fear-avoidance (e.g. reduced activity) due to belief activity/exercise will cause further damage	Low health literacy (3)
B19	Pessimistic about patients' abilities to make lifestyle changes to address their knee OA (not capable)	Low health literacy (3)
B20	Weight loss is sensitive topic (afraid of upsetting their patients results in temptation to avoid discussion)	Challenges of discussing weight (8)
F1	Need for tailored GP education to improve confidence	Having or improving knowledge or skills around OA care in general (2)
F2	Importance of having highly effective communication skills	Having or improving knowledge or skills around LIs or promoting behavioral change (2)
F3	Lifestyle treatments benefited other chronic conditions	LIs have positive effects on general health (1)
F4	Importance of longer consultations	Adequate duration of patient consultations (5)
F5	Government-subsidised allied health visits to facilitate utilisation of services that support exercise/weight loss	Government subsidies (7)
F6	Changes to clinical practice information technology (e.g. prompts into clinic software)	Potential use of information technology (5)
F7	Having access to customizable, printable patient resources	Access to information resources (5)
F8	Having patient resources embedded within current practice software or routines	Potential use of information technology (5)
F9	Patient-centred approach (high level of engagement in providing exercise and weight management advice)	Perception of own role potentially stimulating prescription or follow-up of LIs (2)
F10	Belief that knee OA is condition that can be successfully managed	Optimistic views toward OA (9)
F11	Importance of conveying to patients that diagnosis is not all negative/delivering a relatively positive prognosis	Optimistic views toward OA (9)
F12	Acknowledging that weight loss (when someone is overweight) is important	Positive attitude toward LIs (2)

<u>Hinman (2016) [34]</u>

Number	Description	Subcategory (domain)
B1	Structure/timing of exercise program restricted capacity to modify exercises/provide adequate follow-up	Non-optimal content or structure of LIs (1)
B2	Lack of face-to-face contact difficult/hampered ability to establish normal rapport/build effective relationships	Negative aspects regarding communication and relationship using telehealth (1)
B3	Second professional not necessary to fulfill health coach role (part of own professional	Non-optimal interdisciplinary collaboration or healthcare provision (4)

For peer review only - http://bmjopen.bmj.com/site/about/guidelines.xhtml

	role as physical therapists)	
B4	Overlapping roles of physical therapist and coach could be source of conflict if not working from same set of goals	Non-optimal interdisciplinary collaboration or healthcare provision (4)
B5	Reluctance from patients to talk about physical activity (physical therapist's role, not the coach's role)	Negative attitude toward LIs (3)
B6	Necessary teamwork less likely if communication processes not clearly prescribed/structure not used	Challenges of communication and referral procedures (4)
B7	Necessary teamwork less likely if coach/physical therapist did not recognize/support each other's goals	Non-optimal interdisciplinary collaboration or healthcare provision (4)
B8	Different views were expressed about the preferred medium of communication	Challenges of communication and referral procedures (4)
F1	Appreciation how their participation afforded physical therapists and coaches opportunities to collaborate	Good interdisciplinary collaboration or healthcare provision, or suggestions for improvement (4)
F2	Positive impact on patients of personalized attention from coach and from advice/education they provided	LIs have positive effects (not further specified) (1)
F3	Value of monitoring/encouraging patients to develop own understanding of links between exercise/pain	Having or improving knowledge or skills around LIs or promoting behavioral change (2)
F4	Positive impact of information, education, and structured monitoring on patients' adherence to exercise	High patient adherence or engagement (3)
F5	Requirements of treatment protocol freed therapists to notice and reflect on impact of the interventions	Ease for HCPs during delivery of LIs (1)
F6	Positive comments about the exercise regimen	Positive experiences with or suggestions to improve the content or structure of LIs (1)
F7	Structured protocol allowed to experience different OA treatment regimen/observe and learn from impact	Ease for HCPs during delivery of LIs (1)
F8	Structure provided by protocol/structure of exercises (how patients included them into daily routine)	Positive experiences with or suggestions to improve the content or structure of LIs (1)
F9	Importance of teamwork in delivering the integrated intervention	Good interdisciplinary collaboration or healthcare provision, or suggestions for improvement (4)
F10	Reinforcement of health messages from another clinician could be valuable	Good interdisciplinary collaboration or healthcare provision, or suggestions for improvement (4)
F11	Having a separate coach freed therapists up to focus on other treatment aspects	Good interdisciplinary collaboration or healthcare provision, or suggestions for improvement (4)
F12	Communication needed to be collaborative, patient-centered and consistent for integrated care to be effective	Improving communication between HCPs (4)

Hinman (2017) [35]

Number	Description	Subcategory (domain)

For peer review only

B1	Occasional technical difficulties (e.g. poor internet connection) could disrupt the flow of the consultation	Negative aspects regarding communication and relationship using telehealth (1)
B2	Patient flexibility could come at a cost to the therapist sometimes (allowed patients to reschedule last minute)	Other challenges for HCPs regarding feasibility of telehealth (1)
B3	Forced to modify usual habits/rely more on information shared by patients (instead of own physical assessment)	Challenges for HCPs regarding lack of physical/visual contact (1)
B4	Some discomfort without hands-on assessment (no palpation of patient's knee/hands-on facilitation of exercises)	Challenges for HCPs regarding lack of physical/visual contact (1)
B5	Skype consultations more suitable as adjunctive to usual in-clinic care (initial assessment in person preferred)	Other challenges for HCPs regarding feasibility of telehealth (1)
F1	Ease of using Skype for consultations	Positive attitude or needs of HCPs regarding feasibility of telehealth (1)
F2	Quality of technology suitable for providing instructions/prescribing exercises/receiving instantaneous feedback	Positive attitude or needs of HCPs regarding feasibility of telehealth (1)
F3	Skype-delivered care convenient for patients (time efficiency/flexibility/access)	Patient-related benefits regarding feasibility of telehealth (1)
F4	Empowering effect of home environment on patient adherence with exercise program	Benefits of telehealth in terms of effectiveness (1)
F5	Home environment facilitated correct and safe exercise techniques	Telehealth is safe for patients or patient/data privacy (1)
F6	Using Skype distilled focus to most important and effective treatment elements to facilitate self-management	Benefits of telehealth in terms of effectiveness (1)
F7	Patients more relaxed in home environment/more receptive to the information the therapists provided	Benefits of telehealth in terms of effectiveness (1)
F8	Patients responded favorably to the exercises prescribed despite lack of hands-on assessment	Lack of physical/visual contact not a major issue for HCPs (1)
F9	Safety net provided by research environment (e.g. patients were previously screened for comorbidities/red flags)	Research environment or protocols provide a safety net (1)
F10	Hands-off approach was physically less demanding compared to usual care/contributed to sense of satisfaction	Lack of physical/visual contact not a major issue for HCPs (1)
F11	Functional improvements experienced by patients	LIs have positive effects on affected joint(s) (1)
F12	Functional improvements were observable using Skype	Lack of physical/visual contact not a major issue for HCPs (1)
F13	Greater confidence to exercise among patients	LIs have positive mental effects (1)

Knoop (2020) [36]

Number	Description	Subcategory (domain)
B1	Maximum number of four sessions was considered too low in many patients	Non-optimal content or structure of LIs (1)
B2	Behavioral approach in exercise therapy and advice to visit GP were considered unnecessary for most patients	Non-optimal content or structure of LIs (1)
B3	Interdisciplinary consult with dietician could not always take place because of problems with contacting dietician	Challenges of communication and referral procedures (4)

For peer review only

B4	Interdisciplinary consult with dietician could not always take place because patients refused to visit dietician	Negative attitude toward LIs (3)
F1	Model of stratified care easy to apply and having added value for daily practice	LIs are feasible or sustainable (1)
F2	Appreciation of applicability of treatment protocols	LIs are feasible or sustainable (1)

Law (2019) [37]

Number	Description	Subcategory (domain)
B1	Patients' ideas about whether they wanted surgery influenced making referrals to the LMP	Positive attitude toward TJA (3)
B2	How well they felt the individual patient would engage with programme influenced making referrals to the LMP	Low patient adherence or engagement (3)
B3	Lack of information about scheme hindered referral	Lack of information resources (5)
B4	Feelings of guilt when referring to LMP (dooming patients to a longer wait for surgery)	Potential negative influence of implementing LIs to relationship (8)
B5	Lack of information and patient-professional discussion at point of referral may hinder uptake/retention of LMP	Low health literacy (3)
B6	Lack of time to monitor attendance/provide support was compounded by increasing administrative demands	Lack of time due to other demands (or not further specified) (5)
F1	Multidisciplinary nature of LMP (whole-person, intensive and functional approach)	Positive experiences with or suggestions to improve the content or structure of LIs (1)
F2	Multidisciplinary nature of LMP (ability to utilize expertise from other professionals)	Good interdisciplinary collaboration or healthcare provision, or suggestions for improvement (4)
F3	Suggestion to relist patients completing the programme further up the waiting list (for surgery)	Make use of patients' preference for TJA within LIs (3)
F4	LMP would benefit from extension of inclusion criteria (patients with less severe OA and lower BMI)	LIs are available or accessible, or suggestions for improvement (1)
F5	Emphasising health benefits of programme	LIs have positive effects (not further specified) (1)
F6	Reminding patients of opportunity to self-manage	LIs have positive mental effects (1)
F7	Using bargaining techniques centering on implications of LMP for replacement surgery (put patient on the list)	Make use of patients' preference for TJA within LIs (3)
F8	Standardization was viewed as important for monitoring and evaluation purposes	Positive experiences with or suggestions to improve the content or structure of LIs (1)
F9	Flexibility was valuable when tackling local participation challenges	Ability and importance of providing personalized treatment within LIs (1)
F10	Helpful social impact of group-based programme	Positive experiences with or suggestions to improve the content or structure of LIs (1)
F11	Further and ongoing evaluation of the LMP would help to address current challenges	LIs are feasible or sustainable (1)

For peer review only

U1	Autonomy affects referral considerations	Autonomy (2)

Lawford (2019) [38]

Number	Description	Subcategory (domain)
B1	Telephone not viewed as primary mode of providing care (only for follow-up)	Other challenges for HCPs regarding feasibility of telehealth (1)
B2	Assessment of patients could be difficult when consulting via telephone (inability to observe)	Challenges for HCPs regarding lack of physical/visual contact (1)
B3	Relationships with patients might be adversely impacted/could be difficult to develop rapport	Negative aspects regarding communication and relationship using telehealth (1)
B4	Difficulties communicating might be experienced when consulting via telephone	Negative aspects regarding communication and relationship using telehealth (1)
B5	Lack of visual/physical contact would limit strategies available when teaching patients an exercise program	Challenges for HCPs regarding lack of physical/visual contact (1)
B6	Some difficulty scheduling telephone consultations during usual day of face-to-face consultations	Other challenges for HCPs regarding feasibility of telehealth (1)
F1	Telephone-delivered care would be convenient for patients	Patient-related benefits regarding feasibility of telehealth (1)
F2	Patients could be more comfortable talking about condition/engaging in exercise program from own home	Benefits of telehealth in terms of effectiveness (1)
F3	Telephone-delivered care could reduce patient costs associated with accessing physiotherapy services	Patient-related benefits regarding feasibility of telehealth (1)
F4	Telephone-delivered care could provide increased opportunities to educate patients about OA	Benefits of telehealth in terms of effectiveness (1)
F5	Telephone-delivered care could allow wider variety of patients to access physiotherapy	Patient-related benefits regarding feasibility of telehealth (1)
F6	More effective communication skills would be needed to consult via telephone	Positive attitude or needs of HCPs regarding feasibility of telehealth (1)
F7	It would be necessary to provide patients with pictures or videos of each exercise when consulting via telephone	Positive attitude or needs of HCPs regarding feasibility of telehealth (1)
F8	Experiences providing telephone-delivered care exceeded expectations, resulting in new enthusiasm	Positive attitude or needs of HCPs regarding feasibility of telehealth (1)
F9	Lack of physical and visual contact less of an issue than anticipated	Lack of physical/visual contact not a major issue for HCPs (1)
F10	Developed a strong rapport with patients over the telephone	Positive aspects regarding communication and relationship using telehealth (1)
F11	Patient adherence to telephone-delivered exercise program was high	Benefits of telehealth in terms of effectiveness (1)
F12	Consulting via telephone forced to focus on effective conversations with patients (more personal level)	Positive aspects regarding communication and relationship using telehealth (1)
F13	Noticeable shift in patients' expectations of physiotherapy care (more willing to self-manage their condition)	Patient-related benefits regarding feasibility of telehealth (1)
F14	Improvements in patient pain and function	LIs have positive effects on affected joint(s) (1)
F15	Increased confidence to self-manage	LIs have positive mental effects (1)

For peer review only

F16	Telephone-delivered care was convenient for patients	Patient-related benefits regarding feasibility of telehealth (1)
F17	Able to work around the lack of visual contact (erring on the side of caution)	Lack of physical/visual contact not a major issue for HCPs (1)
F18	Written materials provided to patients helped to prescribe exercises effectively	Positive attitude or needs of HCPs regarding feasibility of telehealth (1)
F19	There was a safety net in place with the trial (each patient had been screened)	Research environment or protocols provide a safety net (1)
F20	Training in communication and/or health coaching important to effectively deliver care over telephone	Positive attitude or needs of HCPs regarding feasibility of telehealth (1)

Lawford (2020) [39]

Number	Description	Subcategory (domain)
B1	Patients were sceptical about safety and benefits of strengthening exercise for OA	Low health literacy (3)
B2	Fear (patients required a lot of encouragement and reassurance)	Low health literacy (3)
B3	Being apprehensive about aggravating pain in patients	LIs are unsafe or have negative effects (1)
B4	People that don't particularly like exercise	Negative attitude toward LIs (3)
B5	Mental effort required for WBE program was challenging for patients	Challenges for patients during participation in LIs (1)
B6	Tending to avoid pushing patients in usual clinical practice	LIs are unsafe or have negative effects (1)
B7	Physical challenge was the complexity of WBE program	Challenges for patients during participation in LIs (1)
B8	Challenges associated with cuff weights used to apply resistance in NWBE program	Challenges for HCPs during delivery of LIs (1)
B9	Straight leg raise challenging in NWBE program	Challenges for patients during participation in LIs (1)
B10	Significant impact of other health problems on patients' ability to commit fully to exercise program	Negative impact of comorbidities (3)
F1	Experiences in study helped them push patients through more pain than they would have previously	Research environment or protocols provide a safety net (1)
F2	NWBE program was generally easier for patients to follow (mental effort)	Ease for patients during participation in LIs (1)
F3	NWBE program was easier to prescribe (mental effort)	Ease for HCPs during delivery of LIs (1)
F4	Most patients tolerated a lot more than was expected (amount of exercise)	Severity of disease and symptoms (3)
F5	Easier to prescribe and progress NWBE than WBE program (physical complexity)	Ease for HCPs during delivery of LIs (1)
F6	Patients adherent/easy to work with when they engaged in exercise program/started seeing improvements	High patient adherence or engagement (3)
F7	Strong therapeutic relationship with patients	Importance of communication and relationship (8)
F8	Importance of pain education and reassurance about safety and benefits of exercise	High health literacy or importance of education (3)
F9	Tailoring exercise programs to individual patient would overcome some challenges	Ability and importance of providing personalized treatment within LIs (1)

Lawford (2021) [40]

For peer review only - http://bmjopen.bmj.com/site/about/guidelines.xhtml

Number	Description	Subcategory (domain)
B1	Video consultations made it more difficult to have emotional conversations/read non-verbal cues	Negative aspects regarding communication and relationship using telehealth (1)
B2	Patients were apprehensive about managing weight by themselves	Low health literacy (3)
B3	Volume of resources could be overwhelming/confusing for some patients	Challenges for patients during participation in LIs (1)
F1	Simplicity and convenience of meal replacements	Ease for patients during participation in LIs (1)
F2	Video consultations were easy and convenient	Positive attitude or needs of HCPs regarding feasibility of telehealth (1)
F3	Pleasantly surprised by experience with video consultations (had some of the best conversations)	Positive aspects regarding communication and relationship using telehealth (1)
F4	Level of support patients had from family/people close to them seemed to make a big difference	Social support (3)
F5	Long-term follow-up consultations would be beneficial	Positive experiences with or suggestions to improve the content or structure of LIs (1)
F6	Cohort of patients, in general, was highly motivated (remained interested/motivated for entirety of 6 months)	High patient adherence or engagement (3)
F7	Rapid weight loss was primary driver of motivation	LIs have positive effects on general health (1)
F8	Extremely positive about educational resources provided	Positive experiences with or suggestions to improve the content or structure of LIs (1)
F9	More information about healthy eating beyond meal replacement phase could be included	Positive experiences with or suggestions to improve the content or structure of LIs (1)
F10	Exercise/physical activity program was an important part of intervention	Positive experiences with or suggestions to improve the content or structure of LIs (1)
F11	Large improvements in knee pain	LIs have positive effects on affected joint(s) (1)
F12	Positive lifestyle changes (patients) (e.g. thinking differently)	LIs have positive mental effects (1)

MacKay (2018) [41]

Number	Description	Subcategory (domain)
B1	Lack of infrastructure or local programmes (particularly in rural settings)	LIs are unavailable or inaccessible (1)
B2	Cost was a factor in whether clients could access facilities/programmes	Costs of LIs to patients (1)
B3	Lack of funding prevented clients from accessing services/seeking help/getting full course of treatment	Restrictions due to health insurance (7)
B4	Clients often had a waiting period before accessing care	LIs are unavailable or inaccessible (1)
B5	Wait lists as a burden	Lack of time due to other demands (or not further specified) (5)
B6	Lack of access to other healthcare providers (e.g. physicians with expertise in OA)	No access to other HCPs (4)
B7	Variability in confidence to provide weight management (not confident)	Lack of knowledge or skills around LIs or promoting behavioral change (2)

For peer review only

B8	Physicians who did not make timely referrals to physical therapy	Non-optimal interdisciplinary collaboration or healthcare provision (4)
B9	Physicians' attitudes could influence clients' perceptions and level of buy-in to physical therapy	Non-optimal interdisciplinary collaboration or healthcare provision (4)
B10	Restricted in amount of time they could allot per patient	Lack of time within patient consultations (5)
B11	Challenges in accessing scientific papers	Challenges in accessing information resources (5)
B12	Challenging work to get clients to initiate management and maintain it over the long term	Low patient adherence or engagement (3)
B13	Disconnect between PTs' recommendations for treatment and clients' expectations or preferences	Negative attitude toward LIs (3)
B14	Prior experiences with physical therapy influenced client expectations of clinical encounter	Negative attitude toward LIs (3)
B15	Some clients had misconceptions about OA (nothing they could do/normalising it as part of ageing)	Low health literacy (3)
B16	Some clients feared participation in exercise (concerns for further degeneration)	Low health literacy (3)
B17	Accepting diagnosis of OA could be particularly challenging for people with early OA	Low health literacy (3)
B18	Clients' socioeconomic status (e.g. great poverty, shelter system)	Limited financial resources (3)
B19	Clients' language (e.g. haven't mastered English/French)	Low health literacy (3)
B20	Clients' family responsibilities (e.g. busy, lot going on)	Other responsibilities (3)
B21	Clients' lifestyle (e.g. coping, attitude towards pain)	Low health literacy (3)
F1	Benefits of having infrastructure and programmes available in their communities	LIs are available or accessible, or suggestions for improvement (1)
F2	Access to a team on-site/a network of healthcare providers they trusted	Access to other HCPs (4)
F3	Importance of good working relationships	Good interdisciplinary collaboration or healthcare provision, or suggestions for improvement (4)
F4	Importance of effective mechanisms to communicate	Needs regarding communication and referral procedures (4)
F5	Confident in capabilities/skills to use strategies they believed to be effective within scope of practice	Having or improving knowledge or skills around LIs or promoting behavioral change (2)
F6	Identifying weight management as important	Positive attitude toward LIs (2)
F7	Variability in confidence to provide weight management (confident)	Having or improving knowledge or skills around LIs or promoting behavioral change (2)
F8	Treatment could improve clients' symptoms (e.g. reduce pain, increase function)	LIs have positive effects on affected joint(s) (1)
F9	Treatment could potentially slow progression of symptoms	LIs have positive effects on affected joint(s) (1)
F10	Physicians who expressed support for physical therapy/exercise and referred clients to physical therapy early	Good interdisciplinary collaboration or healthcare provision, or suggestions for improvement (4)
F11	Physical therapy scope of practice was adequate to manage clients with perceived early knee OA	Adequate professional paradigm or suggestions for expansion (6)
F12	Suggestion that it would be useful to expand scope of practice to include ordering diagnostic imaging	Adequate professional paradigm or suggestions for expansion (6)

For peer review only

F13	Having adequate time to spend with clients	Adequate duration of patient consultations (5)
F14	Access to current evidence	Access to information resources (5)
F15	Professional networks/community of practice as mechanism to facilitate sharing of information	Access to information resources (5)
F16	Client participation in management was critical to see improvement in symptoms	Importance of high patient adherence or engagement for effectiveness of LIs (3)
F17	Viewing themselves as having an important role in supporting clients to participate in management	Perception of own role potentially stimulating prescription or follow-up of LIs (2)
F18	Clients' pre-existing activity level (e.g. active person)	Other patient characteristics (3)
U1	Clients' general health	Other patient characteristics (3)
U2	Clients' occupation	Other responsibilities (3)

MacKay (2020) [42]

Number	Description	Subcategory (domain)
B1	Lack of confidence/uncertainty related to role in weight management	Perception of own role potentially impeding prescription or follow-up of LIs (2)
B2	Weight was touchy/sensitive subject to discuss	Challenges of discussing weight (8)
B3	Not confident in knowledge about weight management	Lack of knowledge or skills around LIs or promoting behavioral change (2)
B4	Perception that discussions related to diet were not part of their scope of practice	Perception of own role potentially impeding prescription or follow-up of LIs (2)
B5	Getting buy-in (engaging people in management) often portrayed as challenge	Low patient adherence or engagement (3)
B6	People's preferences were at odds with physical therapists' beliefs about management	Negative attitude toward LIs (3)
F1	Clinical experience helped to read the person's situation (identify approach to motivate them)	Having or improving knowledge or skills around LIs or promoting behavioral change (2)
F2	Integrating scientific evidence from studies into their approach to management	Access to information resources (5)
F3	Postgraduate continuing professional development courses to expand toolkit of therapeutic interventions	Having or improving knowledge or skills around LIs or promoting behavioral change (2)
F4	Interventions in physical therapists' toolbox were not static (changed over time)	LIs are feasible or sustainable (1)
F5	Perception that exercise and physical activity were central to management	Positive attitude toward LIs (2)
F6	Acknowledging that weight management was a component of management	Positive attitude toward LIs (2)
F7	Confidence in addressing weight management	Having or improving knowledge or skills around LIs or promoting behavioral change (2)
F8	Routinely including education about weight management	Perception of own role potentially stimulating prescription or follow-up of LIs (2)
F9	Feeling comfortable discussing role of physical activity in maintaining weight control	Factors that could ease the way to discussing weight (8)
F10	Those with access to other clinicians recommended to consult another clinician for advice	Access to other HCPs (4)

For peer review only

	on diet as needed	
F11	Developing rapport with people made it easier to discuss weight management	Factors that could ease the way to discussing weight (8)
F12	Getting buy-in (engaging people in management) critical to improving outcomes	Importance of high patient adherence or engagement for effectiveness of LIs (3)
F13	Playing a role in promoting engagement in management	Perception of own role potentially stimulating prescription or follow-up of LIs (2)
F14	Education contributed to buy-in to treatment (pathology, consequences, treatments)	High health literacy or importance of education (3)
F15	Tailoring treatment to a person's goals/interests	Ability and importance of providing personalized treatment within LIs (1)
F16	Need to consider personal context by integrating people's home exercises into daily activities/other life demands	Ability and importance of providing personalized treatment within LIs (1)
F17	Improve people's symptoms early in treatment (to gain buy-in)	LIs have positive effects on affected joint(s) (1)
F18	Having positive attitude/being encouraging of small changes/being hopeful about OA management	Importance of communication and relationship (8)
U1	Driven by their professional experience of what does and doesn't work/trial and error	Clinical experience (2)
U2	Treatments were based more on what works clinically (opposed to scientific evidence)	Clinical experience (2)
U3	Treatment decisions depended on people's symptoms/findings of physical assessment	Severity of disease and symptoms (3)

Mann (2011) [43]

Number	Description	Subcategory (domain)
B1	Insufficient information for OA patients (e.g. not providing leaflets)	Low health literacy (3)
B2	Doubts about patients' willingness to make behavioral changes	Negative attitude toward LIs (3)
B3	Unrealistic expectations of the outcome of joint replacement among patients	Positive attitude toward TJA (3)
B4	OA was not given enough attention, symptoms were often dismissed/minimized in health care	OA seen as low priority (9)
B5	Lack of provision for patients who were not candidates for surgery (too long without help)	Non-optimal interdisciplinary collaboration or healthcare provision (4)
B6	Patients lacked proactive follow-up to support self-management	Non-optimal interdisciplinary collaboration or healthcare provision (4)
B7	Lack of time to give patients sufficient opportunity to discuss their condition	Lack of time within patient consultations (5)
B8	General lack of expertise/interest in OA (that could lead to inappropriate referral/suboptimal access to services)	Lack of knowledge or skills around OA care in general (2)
B9	Lack of facilities to promote continuing exercise in community	LIs are unavailable or inaccessible (1)
B10	Lack of coordination between leisure, social and health services	Non-optimal interdisciplinary collaboration or healthcare provision (4)
B11	Wait for physiotherapy was too long	LIs are unavailable or inaccessible (1)
B12	Insufficient (physiotherapy) intervention when patients were seen	Non-optimal interdisciplinary collaboration or healthcare provision (4)
B13	Belief that physiotherapists did not find it rewarding/interesting to treat OA patients	Non-optimal interdisciplinary collaboration or healthcare provision (4)

For peer review only

F1	Need for early education about OA/self-management and treatment options and opportunity to discuss these	High health literacy or importance of education (3)
F2	Patients would be better served by long-term condition model of care (e.g. diabetes mellitus)	Good interdisciplinary collaboration or healthcare provision, or suggestions for improvement (4)
F3	Patients should initiate own follow-up when needed (as better use of time/health care resources)	Good interdisciplinary collaboration or healthcare provision, or suggestions for improvement (4)
F4	Allow patients, after initial referral, to use direct access system to service (no need for re-referral)	Good interdisciplinary collaboration or healthcare provision, or suggestions for improvement (4)
F5	Care could be improved if every GP practice contained an individual who took a particular interest in OA	Access to other HCPs (4)
F6	There should be OA specialist clinicians (all relevant allied health professions) providing services in community	Access to other HCPs (4)

Miller (2020) [44]

Number	Description	Subcategory (domain)
B1	No effective treatment options	OA seen as untreatable and local condition (wear-and-tear) (9)
B2	Patients don't want to expend effort towards lifestyle change	Negative attitude toward LIs (3)
B3	Lifestyle counseling is huge time commitment	Lack of time within patient consultations (5)
B4	Difficulty convincing patients to consider non-surgical, non-medication treatments	Low health literacy (3)
B5	Lack of physician education on OA care	Lack of knowledge or skills around OA care in general (2)
B6	Patient body weight (overweight/obese) (impedes exercise/makes visits to services more difficult)	Negative impact of comorbidities (3)
B7	Costs related to weight loss can be prohibitive for patients with limited resources (financial burdens)	Limited financial resources (3)
B8	Lack of patient self-efficacy (regarding lifestyle changes)	Low health literacy (3)
B9	Patients delay care until they are highly symptomatic (missing opportunities to slow disease progression)	Severity of disease and symptoms (3)
B10	Lack of knowledge about OA (patient barrier)	Low health literacy (3)
B11	Costs to patients (lack of insurance coverage/high co-pays for specific services/time off work/travel expenses)	Costs of LIs to patients (1)
B12	Inaccessible treatment options within organization	LIs are unavailable or inaccessible (1)
B13	Lack of resources for face-to-face patient education and patient reference	Lack of information resources (5)
B14	Challenges when coordinating multimodal care (including difficulties with the referral system)	Challenges of communication and referral procedures (4)
B15	Appointment times too short to address all of patient's issues and provide lifestyle counseling	Lack of time within patient consultations (5)
B16	Difficulty finding high quality, patient-friendly OA educational materials	Challenges in accessing information resources (5)

For peer review only

B17	Frustration that material found on Internet or provided by friends/family was frequently inaccurate	Challenges in accessing information resources (5)
B18	Surgical methods have the best outcomes	LIs have little or no effect on OA (1)
B19	Changing own practice style remained as barrier after OA training	Perception of own role potentially impeding prescription or follow-up of LIs (2)
F1	Importance of provider knowledge regarding OA management	Having or improving knowledge or skills around OA care in general (2)
F2	Physical therapy helpful for patients most of the time	LIs have positive effects (not further specified) (1)
F3	Utilising clinic health educator who met with patients for weight loss discussions and followed up by phone	Good interdisciplinary collaboration or healthcare provision, or suggestions for improvement (4)
F4	Employing a multi-pronged approach to engage patients in weight loss	Good interdisciplinary collaboration or healthcare provision, or suggestions for improvement (4)
F5	Patients who are well insured have improved access to services (e.g. physical therapy)	Benefits of good health insurance (7)
F6	Physician education on OA management can affect both provider and patient attitudes	Having or improving knowledge or skills around OA care in general (2)
F7	Reframe discussions around exercise and weight loss (e.g. not blaming/discouraging people)	Factors that could ease the way to discussing weight (8)
F8	Recommending informational materials for patients (to mitigate delays in OA care)	Availability of information resources (5)
F9	Standardised flowsheet on OA management (as guide for providers/tool for patient discussions)	Availability of information resources (5)
F10	Electronic reminders for physicians on how to locate OA treatment information and resources	Potential use of information technology (5)

Nielsen (2014) [45]

Number	Description	Subcategory (domain)
B1	Concerns about capacity to learn/not having skills to fulfill study expectations/deal with challenging patients	Lack of knowledge or skills around LIs or promoting behavioral change (2)
B2	Requirements of RCT potentially created a barrier to responding to where the client was	Insufficient ability to provide personalized treatment within LIs (1)
B3	Difficulty for patients with PCST component (cognitive restructuring techniques)	Challenges for patients during participation in LIs (1)
B4	Not have sufficient skills to present PCST component (cognitive restructuring techniques) effectively	Lack of knowledge or skills around LIs or promoting behavioral change (2)
B5	Some process skills were dissimilar to pre-existing clinical communication skills and challenging to use	Lack of knowledge or skills around LIs or promoting behavioral change (2)
B6	Time required to teach PCST skills to patients	Lack of time within patient consultations (5)
B7	Concern about capacity to recover costs of incorporating CBT into practice	Limited financial resources within organization (5)
B8	Lack of knowledge about CBT (necessary to participate in training/RCT to fully appreciate value of CBT to practice)	Lack of knowledge or skills around LIs or promoting behavioral change (2)
B9	Public expectation of what physical therapy treatment should be (e.g. didn't come to have thinking challenged)	Negative attitude toward LIs (3)

For peer review only

F1	Training workshop as good introduction to content and process of delivering PCST program	Ease for HCPs during delivery of LIs (1)
F2	Weekly group interaction crucial to being able to deliver intervention effectively/problem-solve issues	Ease for HCPs during delivery of LIs (1)
F3	Input from supervising psychologist crucial to being able to deliver intervention effectively/problem-solve issues	Ease for HCPs during delivery of LIs (1)
F4	Would have liked more role-playing experience prior to beginning trial treatments	Ease for HCPs during delivery of LIs (1)
F5	Favorably comments on program content (positive way to help people be proactive about their pain)	Positive experiences with or suggestions to improve the content or structure of LIs (1)
F6	Opportunity to review PCST skills and learn more structured/deliberate ways of incorporating these into practice	Having or improving knowledge or skills around LIs or promoting behavioral change (2)
F7	Some modules worked better than others (depending on the individual patient and context)	Ability and importance of providing personalized treatment within LIs (1)
F8	Importance of PCST component (cognitive restructuring techniques)	Positive experiences with or suggestions to improve the content or structure of LIs (1)
F9	Structure of PCST sessions (overview/practice review/covering new skill/practice planning) worked well	Positive experiences with or suggestions to improve the content or structure of LIs (1)
F10	Regular group meetings were considered very important (if not essential) for delivery of PCST program	Ease for HCPs during delivery of LIs (1)
F11	Value of having a psychologist involved throughout the program, their professional input was helpful	Ease for HCPs during delivery of LIs (1)
F12	Expecting to utilize/continue integrating PCST in general clinical work as physical therapist (beyond the study)	Positive attitude toward LIs (2)
F13	The belief that a more flexible approach responsive to patient needs was required in their practice	Ability and importance of providing personalized treatment within LIs (1)
F14	Increasing confidence in using PCST skills over the course of the study	Having or improving knowledge or skills around LIs or promoting behavioral change (2)
F15	Improved interpersonal skills with general clinical patients as a result of participating in the study	Having or improving knowledge or skills around LIs or promoting behavioral change (2)
F16	Value of increasing profession’s explicit understanding/use of PCST skills (practice model may be required)	Adequate professional paradigm or suggestions for expansion (6)
F17	Incorporating selected PCST components on as-needed basis most practical way within current environment	Adequate duration of specific interventions or protocols (5)
F18	Value of incorporating aspects of PCST mind-set into professional training (entry-level vs. postgraduate level)	Adequate professional paradigm or suggestions for expansion (6)

Okwera (2019) [46]

For peer review only - http://bmjopen.bmj.com/site/about/guidelines.xhtml

Number	Description	Subcategory (domain)
B1	Incurable nature and negative prognosis of OA	OA seen as untreatable and local condition (wear-and-tear) (9)
B2	Medical professionals saw OA as low priority with respect to managing their workload	OA seen as low priority (9)
B3	Frustrations about restrictive referral pathways	Challenges of communication and referral procedures (4)
B4	Frustrations about lack of autonomy with decision-making	Negative attitude toward guidelines or protocols (2)
B5	Only two GPs had clear understanding of clinical guidelines on OA	Lack of knowledge or skills around OA care in general (2)
B6	Negativity toward guidelines (clinical reasoning more important)	Negative attitude toward guidelines or protocols (2)
B7	Feeling that patients tended to prefer treatment administered to them	Negative attitude toward LIs (3)
B8	Lack of compliance with home exercise regimes and advice given to patients was common	Low patient adherence or engagement (3)
B9	Lack of confidence in clinical effectiveness of physiotherapy treatments	LIs have little or no effect on OA (1)
B10	Negative comments about patient reports of a lack of “hands-on” physiotherapy	Negative attitude toward LIs (3)
B11	Criticizing the decision to centralize musculoskeletal physiotherapy service (useful to have somebody in team)	Non-optimal interdisciplinary collaboration or healthcare provision (4)
B12	Frustrations about lack of continuity regarding team of physiotherapists within clinic	Non-optimal interdisciplinary collaboration or healthcare provision (4)
B13	Frustrations about lack of contact/communication involved in the referral and discharge process	Lack of communication between HCPs (4)
B14	Referral process was convoluted and at times irrelevant	Challenges of communication and referral procedures (4)
B15	Not working closely with physiotherapists/frustrations about working relationship	Non-optimal interdisciplinary collaboration or healthcare provision (4)
B16	Dissatisfaction about loss of communication since centralizing musculoskeletal physiotherapy service	Lack of communication between HCPs (4)
B17	Dissatisfaction about loss of coherent working since centralizing musculoskeletal physiotherapy service	Non-optimal interdisciplinary collaboration or healthcare provision (4)
F1	There is place for each (self-management programs/physiotherapy/orthopedic consultants) in OA management	Positive attitude toward LIs (2)
F2	Positivity toward private sector (patients will get seen a lot quicker)	Benefits of good health insurance (7)
F3	Reasonable understanding of role physiotherapy plays in management of lower-limb OA	Having or improving knowledge or skills around LIs or promoting behavioral change (2)
F4	Overall positive experience of physiotherapy service and therapists	Good interdisciplinary collaboration or healthcare provision, or suggestions for improvement (4)
F5	Need for improving communication (quality of referrals, information at discharge)	Improving communication between HCPs (4)
F6	In-house physiotherapy (as suggestion for physiotherapy service improvement)	Access to other HCPs (4)
F7	Streamlining the physiotherapy referral process (as suggestion for physiotherapy service improvement)	Needs regarding communication and referral procedures (4)
F8	Training sessions (as suggestion for physiotherapy service improvement)	Having or improving knowledge or skills around OA care in general (2)
F9	Triage service (as suggestion for physiotherapy service improvement)	LIs are available or accessible, or suggestions for improvement (1)
F10	Private healthcare supplementation (as suggestion for physiotherapy service improvement)	Benefits of good health insurance (7)

For peer review only

F11	A web-based physiotherapy service (as suggestion for physiotherapy service improvement)	LIs are available or accessible, or suggestions for improvement (1)
F12	Reduced waiting times (as suggestion for physiotherapy service improvement)	LIs are available or accessible, or suggestions for improvement (1)
U1	Management strategies depended on what the person wants	Patients' preferences (3)
U2	Management strategies depended on what the person can cope with	Health literacy (3)
U3	Management strategies depended on how bad the knee is	Severity of disease and symptoms (3)

Poitras (2010) [47]

Number	Description	Subcategory (domain)
B1	Knee OA seen as uninteresting health problem on which they had limited impact and could not cure	OA seen as untreatable and local condition (wear-and-tear) (9)
B2	Knee OA more often diagnosed as an unanticipated comorbidity (rarely primary reason for consultation)	OA seen as low priority (9)
B3	Not enough emphasis put on primary prevention of knee OA	OA seen as low priority (9)
B4	Most GPs believed their contribution was essentially limited to diagnosis of condition and medication	Perception of own role potentially impeding prescription or follow-up of LIs (2)
B5	Knee OA management seen as unchallenging routine	OA seen as low priority (9)
B6	Rehabilitation potential depended on length of disability (less potential with late management)	Severity of disease and symptoms (3)
B7	Benefits obtained in the long term, which often conflicted with patient expectations for short-term benefits	Negative attitude toward LIs (3)
B8	Paracetamol could mask pain/underlying physical problem (reducing opportunity to assess/manage problem)	Severity of disease and symptoms (3)
B9	Potential further damage to the knee due to activity	LIs are unsafe or have negative effects (1)
B10	Difficult to obtain effective analgesia with some patients	Severity of disease and symptoms (3)
B11	Lack of patient motivation in remaining active despite knee OA	Negative attitude toward LIs (3)
B12	Person had been sedentary throughout life	Other patient characteristics (3)
B13	Potential to create unrealistic expectations and discouragement in patients that were too disabled	Severity of disease and symptoms (3)
B14	Patient views and expectations rarely matched patient needs	Negative attitude toward LIs (3)
B15	Questioning capacity to perform regular exercise because of severity of disability	Severity of disease and symptoms (3)
B16	Questioning capacity to perform regular exercise because of age	Other patient characteristics (3)
B17	Questioning capacity to perform regular exercise because of general health	Negative impact of comorbidities (3)
B18	Questioning capacity to perform regular exercise because of motivation	Negative attitude toward LIs (3)
B19	Disagreement on effective exercise parameters	Non-optimal interdisciplinary collaboration or healthcare provision (4)
B20	Disagreement on optimal design of exercise programs to increase adherence	Non-optimal interdisciplinary collaboration or healthcare provision (4)

For peer review only

B21	Unclear on amount and type of activity necessary to obtain benefits without further damaging the knee	Lack of knowledge or skills around LIs or promoting behavioral change (2)
B22	Most patients demonstrated fatalism/inadequate knowledge and beliefs related to knee OA management	Low health literacy (3)
B23	Patients' adherence to management recommendations was limited (because of fatalism)	Low patient adherence or engagement (3)
B24	Limited impact of weight loss on established knee OA (more effective as a primary prevention strategy)	LIs have little or no effect on OA (1)
B25	Questioning direct relationship between weight and knee OA (numerous other factors associated)	LIs have little or no effect on OA (1)
B26	Knee pain restricts activities in general (which makes weight loss difficult)	Severity of disease and symptoms (3)
B27	Patients with knee OA tended to be older/less active/with slower metabolism (which makes weight loss difficult)	Other patient characteristics (3)
B28	Weight loss is difficult (multiplicity of factors need to be addressed, often involving change in lifestyle)	Potential effects of LIs are difficult to accomplish (1)
F1	Knee OA seen as technically challenging condition	Optimistic views toward OA (9)
F2	Necessity of physiotherapy to effectively rehabilitate knee OA patients (because of knowledge/availability)	Perception of own role potentially stimulating prescription or follow-up of LIs (2)
F3	Rehabilitation potential depended on length of disability (better outcomes with early management)	Severity of disease and symptoms (3)
F4	Many interventions should be used before resorting to medication (including physiotherapy)	Positive attitude toward LIs (2)
F5	Other interventions (including physiotherapy) should be used before paracetamol	Positive attitude toward LIs (2)
F6	NSAIDs alone are not sufficient to appropriately treat inflammation and have to be combined with physiotherapy	Positive attitude toward LIs (2)
F7	Importance of PT's role in educating patients with regards to NSAIDs/alternatives (including physiotherapy)	Perception of own role potentially stimulating prescription or follow-up of LIs (2)
F8	Benefits of activity on knee mobility	LIs have positive effects on affected joint(s) (1)
F9	Benefits of activity on general wellbeing	LIs have positive effects on general health (1)
F10	Effective analgesia necessary for patients to be able to accomplish activities	Severity of disease and symptoms (3)
F11	Patients should be encouraged to resume/maintain daily activities	Positive attitude toward LIs (2)
F12	Necessity of PT involvement in managing activity (because potentially detrimental if excessive)	Perception of own role potentially stimulating prescription or follow-up of LIs (2)
F13	GPs believed PT involvement was necessary to motivate the patient and manage the exercise program	Good interdisciplinary collaboration or healthcare provision, or suggestions for improvement (4)
F14	Exercise planning is usually PT's role (rather than GP's)	Perception of own role potentially stimulating prescription or follow-up of LIs (2)
F15	Exercise programs have to be individualized to each patient by the PT	Ability and importance of providing personalized treatment within LIs (1)

For peer review only - http://bmjopen.bmj.com/site/about/guidelines.xhtml

F16	Necessity of PT follow-up sessions to assess and encourage patient adherence	Perception of own role potentially stimulating prescription or follow-up of LIs (2)
F17	Activity necessary for the knee's health	LIs have positive effects on affected joint(s) (1)
F18	PT's role to individualize patients' activity according to needs and capacity	Perception of own role potentially stimulating prescription or follow-up of LIs (2)
F19	Necessity of patients' active participation in knee OA management (to achieve significant outcomes)	Importance of high patient adherence or engagement for effectiveness of LIs (3)
F20	Although agreeing with active patient participation, it is ultimately PT's role to appropriately manage patients	Perception of own role potentially stimulating prescription or follow-up of LIs (2)
F21	Weight loss effective at improving mobility in general	LIs have positive effects on general health (1)
F22	Weight loss improves pain and joint function	LIs have positive effects on affected joint(s) (1)
F23	Relationship with patients, developed through numerous sessions, facilitated influence for lifestyle modifications	Factors that could ease the way to discussing weight (8)
F24	Weight loss also benefits mobility in general	LIs have positive effects on general health (1)

Rosemann (2006) [48]

Number	Description	Subcategory (domain)
B1	Depression as important barrier to motivate patients to physical exercise	Negative impact of comorbidities (3)
B2	Feeling pressure by patients to refer them to specialist	Positive attitude toward TJA (3)
B3	Specialist did not take time to explain what they had examined/x-rays he had taken	Lack of communication between HCPs (4)
B4	Treatments (e.g. physiotherapy) prescribed less frequently due to decreasing financial resources	Limited financial resources within organization (5)
B5	Treatments (e.g. physiotherapy) prescribed less frequently due to increasing restrictions by health insurances	Restrictions due to health insurance (7)
B6	Not focusing on increasing patients' motivation for behavioural change, but just giving general recommendations	Perception of own role potentially impeding prescription or follow-up of LIs (2)
B7	Success rate in motivating patients too low (distinctly resignated regarding their impact on patients' life style)	Negative attitude toward LIs (3)
B8	Vicious circle (pain when exercising, people move less/eat more due to frustration/sometimes depression)	Potential effects of LIs are difficult to accomplish (1)
B9	Belief that nobody is willing to change lifestyle due to OA, disease has to be a lot worse	OA seen as low priority (9)
B10	Lack of information about self-help groups/offers on community level	Lack of information resources (5)
B11	Frustration about impact of information (e.g. self-help groups) (lot of patients find excuses not to participate)	Negative attitude toward LIs (3)
B12	Missing information about offers e.g. in the community	Lack of information resources (5)
B13	Lack of time	Lack of time due to other demands (or not further specified) (5)

For peer review only

B14	No knowledge about treatment	Lack of knowledge or skills around OA care in general (2)
F1	Gate keeper role for GPs could reduce patients' pressure to refer to orthopaedics/decrease performed x-rays	Good interdisciplinary collaboration or healthcare provision, or suggestions for improvement (4)
F2	Better communication with specialists could increase efficacy of treatment	Improving communication between HCPs (4)
F3	Payment system has to be changed to upgrade conservative treatments and conversation with patient	Financial reward for implementing LIs (5)
F4	Involvement of practice nurses is imaginable in the area of life style counselling and advice giving	Good interdisciplinary collaboration or healthcare provision, or suggestions for improvement (4)
F5	Interventions performed by practice nurses have to be reinsured sufficiently	Financial reward for implementing LIs (5)
F6	Desire to be more involved in life style counselling (upgrade of profession)	Perception of own role potentially stimulating prescription or follow-up of LIs (2)
F7	Knowledge about treatment	Having or improving knowledge or skills around OA care in general (2)
F8	Integrating patients' social system into treatment	Social support (3)
F9	More openly address psychological complaints of patients	Importance of communication and relationship (8)

Selten (2017) [49]

Number	Description	Subcategory (domain)
B1	Ambivalent about patients' ability to lose weight (not able to succeed in making lifestyle changes)	Low health literacy (3)
B2	Mistrust in interventions dieticians use to help patients' with weight reduction attempts	Non-optimal interdisciplinary collaboration or healthcare provision (4)
B3	Mentioning benefits of weight reduction, but not actively coaching or referring patients	Perception of own role potentially impeding prescription or follow-up of LIs (2)
B4	The belief that patients are not capable of losing weight	Low health literacy (3)
B5	Weight reduction advice takes too much time in a consultation	Lack of time within patient consultations (5)
B6	Not perceiving weight reduction advice as their responsibility	Perception of own role potentially impeding prescription or follow-up of LIs (2)
B7	Difficulties in communicating with patients about being overweight	Challenges of discussing weight (8)
B8	Uncertainties about dosage/frequency/type of physical activity	Lack of knowledge or skills around LIs or promoting behavioral change (2)
B9	Less certain about effectiveness of physical therapy (benefits variable or difficult to prove)	LIs have little or no effect on OA (1)
B10	Negative views about physical therapists who provided non-evidence-based treatments	Non-optimal interdisciplinary collaboration or healthcare provision (4)
B11	Mistrust because they observed huge differences in quality of care delivered by physical therapists	Non-optimal interdisciplinary collaboration or healthcare provision (4)
B12	Occupational therapists, podiatrists and physical therapists do not work together optimally in OA care	Non-optimal interdisciplinary collaboration or healthcare provision (4)
B13	Role of rheumatologist in knee/hip OA care perceived as unclear/limited	Non-optimal interdisciplinary collaboration or healthcare provision (4)

For peer review only

B14	Agreement that orthopedic surgeon’s primary task is to assess whether patient is eligible for surgery	Non-optimal interdisciplinary collaboration or healthcare provision (4)
B15	Orthopedic surgeons were perceived negatively by several healthcare providers	Non-optimal interdisciplinary collaboration or healthcare provision (4)
F1	Benefits of weight reduction for relieving symptoms of knee/hip OA	LIs have positive effects on affected joint(s) (1)
F2	Ambivalent about patients’ ability to lose weight (able)	High health literacy or importance of education (3)
F3	Dieticians are helpful for patients trying to lose weight	Good interdisciplinary collaboration or healthcare provision, or suggestions for improvement (4)
F4	Having a relationship with patient built on mutual trust/respect would ease way to discussing weight reduction	Factors that could ease the way to discussing weight (8)
F5	Value of lifestyle advice related to knee and hip OA	LIs have positive effects (not further specified) (1)
F6	Beneficial effects of physical therapy in reducing pain/stiffness and potential effects on cartilage	LIs have positive effects on affected joint(s) (1)
F7	Beneficial effects of physical therapy in reducing weight and for increasing mobility/posture/coordination	LIs have positive effects on general health (1)
F8	Physical therapy useful in increasing patients self-management in coping with/acceptance of symptoms	LIs have positive mental effects (1)
F9	Need for physical therapists to provide evidence-based exercises instead of non-evidence-based modalities	Good interdisciplinary collaboration or healthcare provision, or suggestions for improvement (4)
F10	Non-pharmacological, non-surgical treatment was considered useful to delay surgery	LIs have positive effects (not further specified) (1)
F11	Good communication with patient may help in delaying surgery	Importance of communication and relationship (8)
F12	Straightforward, easy and quick lines of communication among different disciplines in healthcare center	Needs regarding communication and referral procedures (4)
F13	Collaboration among multiple disciplines could be facilitated by working in a health center	Benefits of working in health centers (5)
F14	Non-pharmacological, non-surgical OA care can and should be provided in a primary care setting	Good interdisciplinary collaboration or healthcare provision, or suggestions for improvement (4)
F15	GPs have coordinating role (diagnose/monitor, refer when necessary, lifestyle education, long-term coach)	Good interdisciplinary collaboration or healthcare provision, or suggestions for improvement (4)
F16	Importance of having trust	Importance of communication and relationship (8)
F17	Physical therapists can guide patients/provide lifestyle advice (more time compared with GPs)	Good interdisciplinary collaboration or healthcare provision, or suggestions for improvement (4)
F18	Perceiving rheumatologists' role as valuable (giving injections, providing lifestyle/medication advice, refer)	Good interdisciplinary collaboration or healthcare provision, or suggestions for improvement (4)

Tang (2020) [50]

Number	Description	Subcategory (domain)
B1	Inability to discuss specific details of ACSM guideline	Lack of knowledge or skills around specific resources (2)

For peer review only

B2	Pain (main barrier resulting in reduced dosage prescription of strengthening exercises)	Severity of disease and symptoms (3)
B3	Patient's ability to exercise (main barrier resulting in reduced dosage prescription of strengthening exercises)	Severity of disease and symptoms (3)
B4	Unaware about practice guidelines in relation to aerobic exercise prescription/weight loss/pain management	Lack of knowledge or skills around LIs or promoting behavioral change (2)
B5	Knowledge about BMI/weight management was particularly poor (e.g. relying on visual estimations)	Lack of knowledge or skills around LIs or promoting behavioral change (2)
B6	Limited knowledge of how to address weight management	Lack of knowledge or skills around LIs or promoting behavioral change (2)
B7	Pain (key barrier to prescription of exercise as recommended by CPGs)	Severity of disease and symptoms (3)
B8	Less awareness about aerobic exercise prescription	Lack of knowledge or skills around LIs or promoting behavioral change (2)
B9	Pain (barrier to prescription of aerobic exercise)	Severity of disease and symptoms (3)
B10	Uncertainty over scope of practice/questioning whether weight and pain management fall outside scope	Perception of own role potentially impeding prescription or follow-up of LIs (2)
B11	Reduced confidence with recommending individual weight/pain management plans (discuss in general terms)	Lack of knowledge or skills around LIs or promoting behavioral change (2)
B12	Viewing weight as sensitive subject/feeling uncomfortable discussing it	Challenges of discussing weight (8)
F1	Knowledge/confidence in providing treatments related to strengthening and range of motion	Having or improving knowledge or skills around LIs or promoting behavioral change (2)
F2	Being aware about ACSM guidelines	Available resources might improve knowledge and decision-making (2)
F3	Confident in providing justifications for non-routinely adhering to guidelines (range of motion exercises)	Having or improving knowledge or skills around LIs or promoting behavioral change (2)
F4	Knowing the importance of weight management for knee OA	Positive attitude toward LIs (2)
F5	Being able to describe how they will manage pain during strengthening exercise	Having or improving knowledge or skills around LIs or promoting behavioral change (2)

Teo (2020) [51]

Number	Description	Subcategory (domain)
B1	Knee OA was perceived as a degenerative (wear and tear)	OA seen as untreatable and local condition (wear-and-tear) (9)
B2	Describing own role as prepping patients for knee surgery when they were referred for physiotherapy	Perception of own role potentially impeding prescription or follow-up of LIs (2)
B3	Comorbidities (often more severe pain, hampering ability to exercise or be physically active)	Negative impact of comorbidities (3)
B4	Patients' unsatisfactory adherence to exercise programs	Low patient adherence or engagement (3)
B5	Self-motivation (intrinsic barrier for patient adherence)	Negative attitude toward LIs (3)

For peer review only

B6	Fear of falling (intrinsic barrier for patient adherence)	Low health literacy (3)
B7	Fear of pain (intrinsic barrier for patient adherence)	Low health literacy (3)
B8	Costs (extrinsic barrier for patient adherence)	Costs of LIs to patients (1)
B9	Weather (extrinsic barrier for patient adherence)	Inconvenience to patients when accessing LIs (1)
B10	Patient expectations (not keen to participate in exercise/play active role in management, desire for quick fix)	Negative attitude toward LIs (3)
B11	Lack of confidence/knowledge/skills in implementing evidence into practice (e.g. weight management)	Lack of knowledge or skills around LIs or promoting behavioral change (2)
B12	Advice about how to lose weight was limited to brief general advice	Perception of own role potentially impeding prescription or follow-up of LIs (2)
B13	Considering weight loss to be outside own scope of practice (role of a dietician)	Perception of own role potentially impeding prescription or follow-up of LIs (2)
B14	Comfortable suggesting surgery to patients who responded poorly to conservative management	Perception of own role potentially impeding prescription or follow-up of LIs (2)
F1	Education focused on self-management strategies	High health literacy or importance of education (3)
F2	Importance of evaluating a patient's overall functional ability (rather than only knee signs/symptoms)	Other patient characteristics (3)
F3	Perceiving exercise prescription to be their main role	Perception of own role potentially stimulating prescription or follow-up of LIs (2)
F4	Importance of tailored exercise program	Ability and importance of providing personalized treatment within LIs (1)
F5	Advising patients against surgery for as long as possible (last option)	Perception of own role potentially stimulating prescription or follow-up of LIs (2)
F6	Implementing several strategies to boost adherence	Perception of own role potentially stimulating prescription or follow-up of LIs (2)
F7	Aware that being overweight/obese is risk factor for knee OA/losing weight is important	Positive attitude toward LIs (2)
F8	Not their role to advise the patient about knee surgery, opting not to discuss surgery at all	Perception of own role potentially stimulating prescription or follow-up of LIs (2)
F9	Advising patients against knee arthroscopy if specifically asked about this procedure	Perception of own role potentially stimulating prescription or follow-up of LIs (2)

Wallis (2020) [52]

Number	Description	Subcategory (domain)
B1	Cost (program access barrier)	Costs of LIs to patients (1)
B2	Transport, waiting time and parking related to attendance (program access barrier)	Inconvenience to patients when accessing LIs (1)
B3	Geography (program access barrier)	Inconvenience to patients when accessing LIs (1)
B4	Available session times (program access barrier)	Inconvenience to patients when accessing LIs (1)

For peer review only - http://bmjopen.bmj.com/site/about/guidelines.xhtml

B5	Using negative language to describe OA (wear-and-tear/joint damage/bone-on-bone/degenerative condition)	OA seen as untreatable and local condition (wear-and-tear) (9)
B6	Existing comorbidities (patient-related barrier)	Negative impact of comorbidities (3)
B7	Osteoarthritis severity (mild/severe) (patient-related barrier)	Severity of disease and symptoms (3)
B8	Lack of motivation to participate active lifestyle interventions (patient-related barrier)	Negative attitude toward LIs (3)
B9	Older age (patient-related barrier)	Other patient characteristics (3)
B10	Language/different cultural backgrounds (patient-related barrier)	Low health literacy (3)
B11	Work/other commitments precluding exercise-therapy (patient-related barrier)	Other responsibilities (3)
B12	Program factors (e.g. single discipline led intervention)	Non-optimal content or structure of LIs (1)
B13	Existing relationships with physiotherapists (as barrier to referral if patient already had treating physiotherapist)	Negative attitude toward LIs (3)
B14	Urging caution to patients about participating in higher impact exercise/activities	LIs are unsafe or have negative effects (1)
F1	A more holistic program as part of a multidisciplinary model of service was preferred	Positive experiences with or suggestions to improve the content or structure of LIs (1)
F2	Knowledge that program was delivered by well-trained and trusted physiotherapist	Good interdisciplinary collaboration or healthcare provision, or suggestions for improvement (4)
F3	Receiving communication back from program physiotherapist about patient outcomes	Improving communication between HCPs (4)
F4	Positive about program (alternative approach and opportunity to avoid a joint replacement)	LIs have positive effects (not further specified) (1)
F5	Exercise therapy may be effective by giving more muscular support for joints	LIs have positive effects on affected joint(s) (1)
F6	Exercise therapy may be effective by giving opportunity to improve confidence about activities/mobility	LIs have positive mental effects (1)
F7	Value of program's structure and peer (group) support	Positive experiences with or suggestions to improve the content or structure of LIs (1)
F8	Name of program (‘Good Life with OsteoArthritis’) implied optimism and positive outcome	Positive experiences with or suggestions to improve the content or structure of LIs (1)
F9	Received positive feedback from their patients about program	Positive experiences with or suggestions to improve the content or structure of LIs (1)
F10	Including links on websites of partners (suggestion for promotion and referrals)	Access to information resources (5)
F11	Simple, streamlined referral process (suggestion for promotion and referrals)	Needs regarding communication and referral procedures (4)
F12	Close, convenient locations (suggestion for promotion and referrals)	Convenience for patients when accessing LIs (1)
F13	Appropriate session times for working populations (suggestion for promotion and referrals)	Convenience for patients when accessing LIs (1)
F14	Specific information about program (suggestion for promotion and referrals)	Availability of information resources (5)
F15	Providing trial of sessions to assist patients to get started (suggestion for promotion and referrals)	Ease for patients during participation in LIs (1)
F16	Provision of free parking at health service (suggestion for promotion and referrals)	Convenience for patients when accessing LIs (1)

For peer review only

Cottrell (2016) [53]

Number	Description	Subcategory (domain)
B1	Insufficient time in consultations (*)	Lack of time within patient consultations (5)
B2	Limitations to accessing services (e.g. lack of facilities, costs) (*)	LIs are unavailable or inaccessible (1)
B3	Services do not meet expectations (*)	Non-optimal content or structure of LIs (1)
B4	Geographical problems (e.g. remote location, scared to walk in local area) (*)	Inconvenience to patients when accessing LIs (1)
B5	Cannot access necessary resources (*)	Challenges in accessing information resources (5)
B6	GP does not prioritise exercise (*)	Negative attitude toward LIs (2)
B7	Unclear what physio offers (*)	Non-optimal interdisciplinary collaboration or healthcare provision (4)
B8	Exercise does not match patient needs/expectations (*)	Negative attitude toward LIs (3)
B9	Achieving patient behavior change is difficult (*)	Potential effects of LIs are difficult to accomplish (1)
B10	GPs should (perhaps) not follow-up patients to monitor extent of continuation of exercises (*)	Perception of own role potentially impeding prescription or follow-up of LIs (2)
B11	It is the patient's own responsibility to continue doing their exercise programme (*)	Perception of own role potentially impeding prescription or follow-up of LIs (2)
B12	Increasing the overall activity levels does not/might not stop the knee problem getting worse (*)	LIs have little or no effect on OA (1)
B13	Time constraints prevent GPs from providing advice on individual exercises for CKP (*)	Lack of time within patient consultations (5)
F1	Physiotherapy (referral) needs to be prioritised	Positive attitude toward LIs (2)
F2	It is part of my job to reassure patients about the safety of exercise for CKP (*)	Perception of own role potentially stimulating prescription or follow-up of LIs (2)
F3	Exercise for CKP is most beneficial when it is tailored to meet individual patient needs (*)	Ability and importance of providing personalized treatment within LIs (1)
F4	A standard set of exercises is not/might not be sufficient for every patient with chronic knee problems (*)	Ability and importance of providing personalized treatment within LIs (1)
F5	GPs should educate patients with CKP about how to change their lifestyle for the better (*)	Perception of own role potentially stimulating prescription or follow-up of LIs (2)
F6	It is important that people with CKP increase their overall activity levels (*)	Positive attitude toward LIs (2)
F7	How well a patient complies with their exercise programme determines how effective it will be (*)	Importance of high patient adherence or engagement for effectiveness of LIs (3)
F8	GPs should prescribe quadriceps strengthening exercises to every patient with CKP (*)	Perception of own role potentially stimulating prescription or follow-up of LIs (2)
F9	GPs should prescribe general exercise (e.g. walking or swimming) for every patient with CKP (*)	Perception of own role potentially stimulating prescription or follow-up of LIs (2)
F10	Knee problems are improved by quadriceps strengthening exercises (*)	LIs have positive effects on affected joint(s) (1)
F11	Knee problems are improved by general exercise (e.g. walking or swimming) (*)	LIs have positive effects on affected joint(s) (1)

For peer review only

F12	Quadriceps strengthening exercises for the knee are safe for everybody to do (*)	LIs are safe (1)
F13	General exercise (e.g. walking or swimming) is safe for everybody to do (*)	LIs are safe (1)
F14	Exercise is effective for patients if an X-ray shows severe knee OA (*)	Severity of disease and symptoms (3)
F15	Increasing the strength of the muscles around the knee stops the knee problem getting worse (*)	LIs have positive effects on affected joint(s) (1)
F16	Exercise for CKP should (perhaps) preferably be used before drug treatment has been tried (*)	Positive attitude toward LIs (2)
F17	Exercise for CKP is more effectively provided by physiotherapists than GPs (*)	Good interdisciplinary collaboration or healthcare provision, or suggestions for improvement (4)
U1	Exercise does not/might not work just as well for everybody, regardless of the amount of pain they have (*)	Severity of disease and symptoms (3)

Duarte (2019) [54]

Number	Description	Subcategory (domain)
B1	Main concern was participant adherence to physical activity routines after end of program	Low patient adherence or engagement (3)
F1	Improvement in the physical condition of participants	LIs have positive effects (not further specified) (1)
F2	Enthusiastic participation of the participants	High patient adherence or engagement (3)

Hill (2018) [55]

Number	Description	Subcategory (domain)
B1	Not (or perhaps not) interested in being the orthopedic surgeon in an ortho-bariatric centre (*)	Perception of own role potentially impeding prescription or follow-up of LIs (2)
F1	There was a BMI threshold above which they would not perform a TKA at all (*)	Perception of own role potentially stimulating prescription or follow-up of LIs (2)
F2	There was a BMI threshold above which they would not perform a TKA until the patient had attended a weight management program (*)	Perception of own role potentially stimulating prescription or follow-up of LIs (2)
F3	Weight loss should be the first-line treatment in the management of obese patients with symptomatic knee OA (*)	Positive attitude toward LIs (2)
F4	Obese patients with symptomatic knee OA should be referred to a specialist weight management service before orthopaedic assessment (*)	Positive attitude toward LIs (2)
F5	Obese patients with symptomatic knee OA should be assessed by a specialist multidisciplinary service, which should include an orthopaedic surgeon (*)	Good interdisciplinary collaboration or healthcare provision, or suggestions for improvement (4)
F6	Support for creation of regional centres where orthopedic surgeons and bariatric surgeons, with their respective teams, could assess obese patients with symptomatic knee pain (*)	Good interdisciplinary collaboration or healthcare provision, or suggestions for improvement (4)

For peer review only

Hill (2018) [56]

Number	Description	Subcategory (domain)
B1	I don't have/might not have the required knowledge and training around obesity care (*)	Lack of knowledge or skills around LIs or promoting behavioral change (2)
B2	Weight management services are not/might not be adequately commissioned in my area (*)	LIs are unavailable or inaccessible (1)
F1	Weight loss should be the first-line treatment in the management of symptomatic knee OA in obesity (*)	Positive attitude toward LIs (2)
F2	Community interventions are effective at achieving sufficient and sustained weight loss (*)	LIs have positive effects on general health (1)
F3	Obese patients with symptomatic knee OA should be referred to a specialist weight management service before orthopaedic assessment (*)	Good interdisciplinary collaboration or healthcare provision, or suggestions for improvement (4)
F4	Obese patients with symptomatic knee OA should be assessed by a specialist multidisciplinary service (*)	Good interdisciplinary collaboration or healthcare provision, or suggestions for improvement (4)
F5	Support for creation of regional centres where orthopaedic surgeons and bariatric surgeons, with their respective teams, could assess obese patients with symptomatic knee pain (*)	Positive attitude toward LIs (2)
F6	Intention to refer patients to an ortho-bariatric centre if it existed (*)	Perception of own role potentially stimulating prescription or follow-up of LIs (2)

Hofstede (2016) [57]

Number	Description	Subcategory (domain)
F1	Clear referral criteria/guideline (*)	Availability of information resources (5)
F2	Important to follow guidelines (*)	Positive attitude toward guidelines or protocols (2)
F3	Important to try non-surgical treatments first (*)	Positive attitude toward LIs (2)
F4	Only few drawbacks for the use of non-surgical treatments (*)	LIs are safe (1)
F5	Patients benefit from weight loss (*)	LIs have positive effects on general health (1)
F6	Non-surgical treatments motivate patients to do things themselves (*)	LIs have positive mental effects (1)
F7	Good results of physical therapy (*)	LIs have positive effects (not further specified) (1)
F8	Important to delay a surgery as long as possible (*)	Positive attitude toward LIs (2)
F9	Agreements with colleagues about the content of the care trajectory (*)	Good interdisciplinary collaboration or healthcare provision, or suggestions for improvement (4)
F10	Peer review/audit of professional association (*)	Audit (6)
F11	Positive attitudes of colleagues about non-surgical treatments (*)	Good interdisciplinary collaboration or healthcare provision, or suggestions for improvement (4)
F12	Clarity on what the patient has done at the physical therapist (*)	Improving communication between HCPs (4)
F13	Agreements/ deliberations with primary care (GP, physical therapist, dietician) (*)	Good interdisciplinary collaboration or healthcare provision, or suggestions for improvement (4)

For peer review only - http://bmjopen.bmj.com/site/about/guidelines.xhtml

F14	Availability of non-surgical treatments (*)	LIs are available or accessible, or suggestions for improvement (1)

Lawford (2018) [58]

Number	Description	Subcategory (domain)
B1	Using the telephone to consult with an OA patient and prescribe an exercise program would not/might not be easy for me (*)	Other challenges for HCPs regarding feasibility of telehealth (1)
B2	I would not/might not be as satisfied talking to an OA patient over the telephone as I would be talking to the patient in person in my consulting room (*)	Other challenges for HCPs regarding feasibility of telehealth (1)
B3	An exercise program prescribed by a PT over the telephone would not/might not improve a patient's OA (*)	Disadvantages of telehealth in terms of effectiveness (1)
B4	I would not/might not be able to adequately monitor a patient's OA over the telephone (*)	Challenges for HCPs regarding lack of physical/visual contact (1)
B5	I do not/might not like that there would be no physical contact with an OA patient when consulting over the telephone (*)	Challenges for HCPs regarding lack of physical/visual contact (1)
B6	I do not/might not like that there would be no physical contact with an OA patient when consulting over the internet video (*)	Challenges for HCPs regarding lack of physical/visual contact (1)
B7	I would not/might not be interested in being involved in a service offering PT-prescribed exercise over the telephone for my people with OA (*)	Other challenges for HCPs regarding feasibility of telehealth (1)
B8	Using the telephone would not/might not be an acceptable way for me to deliver an exercise program to patients with OA (*)	Other challenges for HCPs regarding feasibility of telehealth (1)
B9	Using the telephone would not/might not be a useful (practical) way for me to deliver an exercise program to patients with OA (*)	Other challenges for HCPs regarding feasibility of telehealth (1)
B10	Using the telephone would not/might not be an effective way for me to deliver an exercise program to patients with OA (*)	Other challenges for HCPs regarding feasibility of telehealth (1)
B11	Using the telephone would not/might not be a safe way for patients to receive a PT-prescribed exercise program for their OA (*)	Telehealth is not safe for patients or patient/data privacy (1)
F1	Exercise is beneficial for OA (*)	LIs have positive effects on affected joint(s) (1)
F2	I would get a good understanding of a patient's OA over the telephone (*)	Lack of physical/visual contact not a major issue for HCPs (1)
F3	I would get a good understanding of a patient's OA over the internet video (*)	Lack of physical/visual contact not a major issue for HCPs (1)
F4	A patient's privacy would not be violated if I prescribed them an exercise program over the telephone (*)	Telehealth is safe for patients or patient/data privacy (1)
F5	A patient's privacy would not be violated if I prescribed them an exercise program over the internet video (*)	Telehealth is safe for patients or patient/data privacy (1)
F6	Using the internet video to consult with an OA patient and prescribe an exercise program would be easy for me (*)	Positive attitude or needs of HCPs regarding feasibility of telehealth (1)

For peer review only

F7	I would be as satisfied talking to an OA patient over the internet video as I would be talking to the patient in person in my consulting room (*)	Positive attitude or needs of HCPs regarding feasibility of telehealth (1)
F8	An exercise program prescribed by a PT over the internet video would improve a patient's OA (*)	Benefits of telehealth in terms of effectiveness (1)
F9	An exercise program prescribed by a PT over the telephone would save a patient money (*)	Patient-related benefits regarding feasibility of telehealth (1)
F10	An exercise program prescribed by a PT over the internet video would save a patient money (*)	Patient-related benefits regarding feasibility of telehealth (1)
F11	I would be able to adequately monitor a patient's OA over the internet video (*)	Lack of physical/visual contact not a major issue for HCPs (1)
F12	Receiving an exercise program from a PT over the telephone would be a convenient form of health care for an OA patient (*)	Patient-related benefits regarding feasibility of telehealth (1)
F13	Receiving an exercise program from a PT over the internet video would be a convenient form of health care for an OA patient (*)	Patient-related benefits regarding feasibility of telehealth (1)
F14	Receiving an exercise program from a PT over the telephone would save the patient time (*)	Patient-related benefits regarding feasibility of telehealth (1)
F15	Receiving an exercise program from a PT over the internet video would save the patient time (*)	Patient-related benefits regarding feasibility of telehealth (1)
F16	I would be interested in being involved in a service offering PT-prescribed exercise over the internet video for my people with OA (*)	Positive attitude or needs of HCPs regarding feasibility of telehealth (1)
F17	Using the internet video would be an acceptable way for me to deliver an exercise program to patients with OA (*)	Positive attitude or needs of HCPs regarding feasibility of telehealth (1)
F18	Using the internet video would be a useful (practical) way for me to deliver an exercise program to patients with OA (*)	Positive attitude or needs of HCPs regarding feasibility of telehealth (1)
F19	Using the internet video would be an effective way for me to deliver an exercise program to patients with OA (*)	Positive attitude or needs of HCPs regarding feasibility of telehealth (1)
F20	Using the telephone would be an affordable way for patients to receive a PT-prescribed exercise program for their OA (*)	Patient-related benefits regarding feasibility of telehealth (1)
F21	Using the internet video would be an affordable way for patients to receive a PT-prescribed exercise program for their OA (*)	Patient-related benefits regarding feasibility of telehealth (1)
F22	Using the internet video would be a safe way for patients to receive a PT-prescribed exercise program for their OA (*)	Telehealth is safe for patients or patient/data privacy (1)

Reid (2014) [59]

Number	Description	Subcategory (domain)
B1	Lack of availability of physiotherapy	LIs are unavailable or inaccessible (1)
B2	Poor rate of previous (physiotherapy) success (*)	Other patient characteristics (3)
B3	There is a paucity of evidence in regards to the effectiveness of physiotherapy treatment for	LIs have little or no effect on OA (1)

	OA hip and/or knee (*)	
B4	Past experience has shown physiotherapy to be ineffective (*)	LIs have little or no effect on OA (1)
F1	Referring patients to physiotherapy if they had high levels of pain/disability and where radiographic evidence of OA was present (*)	Severity of disease and symptoms (3)
F2	Referring patients to physiotherapy if they were of a younger age (*)	Other patient characteristics (3)
F3	Good access to physiotherapy in area (*)	LIs are available or accessible, or suggestions for improvement (1)
F4	Physiotherapists do not/might not lack expertise in OA management (*)	Good interdisciplinary collaboration or healthcare provision, or suggestions for improvement (4)
F5	Conservative treatment is (perhaps) an important part of OA management (*)	Positive attitude toward LIs (2)

De Rooij (2020) [60]

Number	Description	Subcategory (domain)
B1	In my daily clinical practice I can (perhaps) not integrate working according to the protocol well (*)	LIs are not feasible or sustainable (1)
B2	The lay out of the protocol does not/might not facilitate its usage in daily practice (*)	Challenges for HCPs during delivery of LIs (1)
B3	I do not/might not treat enough patients with knee OA and comorbidity to apply the protocol (*)	LIs are not feasible or sustainable (1)
B4	The protocol does not/might not fit well with my working methods of daily clinical practice (*)	LIs are not feasible or sustainable (1)
B5	I do not/might not have sufficient knowledge about knee OA exercise therapy and comorbidity to apply the protocol in daily clinical practice (*)	Lack of knowledge or skills around LIs or promoting behavioral change (2)
B6	I do not/might not have sufficient skills to apply the protocol in daily clinical practice (*)	Lack of knowledge or skills around specific resources (2)
B7	I do not/might not read the protocol sufficiently to remember any of its contents (*)	Lack of knowledge or skills around specific resources (2)
B8	The number of treatments that the patient receives from their insurance company is a barrier in using the protocol (*)	Restrictions due to health insurance (7)
B9	The patients are not/might not be cooperative in applying the protocol in daily clinical practice (*)	Negative attitude toward LIs (3)
B10	My colleagues in physiotherapy are not/might not be cooperative in applying the protocol in daily clinical practice (*)	Non-optimal interdisciplinary collaboration or healthcare provision (4)
B11	The management of my practice is not/might not be collaborative regarding the application of the protocol in daily clinical practice (*)	Management not supportive (6)
B12	The general practitioners or other physicians are not/might not be collaborative regarding the application of the protocol in daily clinical practice (*)	Non-optimal interdisciplinary collaboration or healthcare provision (4)
B13	Suboptimal collaboration with general practitioners and physicians	Non-optimal interdisciplinary collaboration or healthcare provision (4)
B14	Referring physicians do not always believe in/may lack knowledge about effectiveness of exercise therapy	Non-optimal interdisciplinary collaboration or healthcare provision (4)
B15	Patients do not always believe in/may lack knowledge about effectiveness of exercise therapy	Low health literacy (3)
B16	Total amount of knee OA patients with comorbidity was lower than expected	LIs are not feasible or sustainable (1)

For peer review only

B17	Number of treatment sessions patients receive from insurance companies restricted application of the strategy	Restrictions due to health insurance (7)
B18	Patients with knee OA and comorbidity are not always motivated to perform exercises	Negative attitude toward LIs (3)
B19	Requesting medical information about patients from specialists takes a lot of time	Challenges of communication and referral procedures (4)
B20	Physicians are not always collaborating in discussing medical conditions of patients	Lack of communication between HCPs (4)
F1	The protocol is feasible in daily clinical practice (*)	LIs are feasible or sustainable (1)
F2	The protocol supports me in clinical reasoning (*)	Available resources might improve knowledge and decision-making (2)
F3	The protocol gives the opportunity to make your own decisions regarding history taking, physical examination, and treatment (*)	Ability and importance of providing personalized treatment within LIs (1)
F4	Some contents of the protocol are not/might not be incorrect (*)	Positive experiences with or suggestions to improve the content or structure of LIs (1)
F5	In my daily clinical practice, I work with sufficient equipment (including blood pressure meter, saturation meter) to properly apply the protocol (*)	LIs are feasible or sustainable (1)
F6	The protocol is supporting the improvement of my knowledge regarding knee OA exercise therapy and comorbidity (*)	Available resources might improve knowledge and decision-making (2)
F7	The recommendations over adapting the diagnostic phase (history taking and physical examination) in the protocol are clear and understandable (*)	Ease for HCPs during delivery of LIs (1)
F8	The recommendations over adapting the OA exercise therapy in the protocol are clear and understandable (*)	Ease for HCPs during delivery of LIs (1)
F9	The protocol is supportive in which comorbidity-related symptoms I need to monitor before, during and after treatment (*)	Available resources might improve knowledge and decision-making (2)
F10	Working with the protocol invites me to discuss more with experts in the field of the comorbidity (*)	Good interdisciplinary collaboration or healthcare provision, or suggestions for improvement (4)
F11	In general, I do not/might not feel resistance towards working according to protocols (*)	Positive attitude toward guidelines or protocols (2)
F12	I have changed my working method (due to the protocol) (*)	LIs are feasible or sustainable (1)
F13	Working according to the protocol is not/might not be too time-consuming (*)	Adequate duration of specific interventions or protocols (5)
F14	Working according to the protocol should be financially rewarded (*)	Financial reward for implementing LIs (5)
F15	The protocol is applicable to OA patients with comorbidity that I see in my clinical practice (*)	Positive experiences with or suggestions to improve the content or structure of LIs (1)
F16	Intake procedure is feasible and implementable	LIs are feasible or sustainable (1)
F17	Important to extend the intake phase to at least to 45 min	Positive experiences with or suggestions to improve the content or structure of LIs (1)
F18	The more you apply the strategy in daily practice, the easier it is to integrate it in your daily working method	LIs are feasible or sustainable (1)
F19	More insight into exercise tolerance/more background knowledge to make clinical decision by using strategy	Available resources might improve knowledge and decision-making (2)
F20	Inform referrers better about benefits of exercise therapy in patients with knee OA and	Good interdisciplinary collaboration or healthcare provision, or

For peer review only - http://bmjopen.bmj.com/site/about/guidelines.xhtml

	comorbidity	suggestions for improvement (4)
F21	Inform patients with knee OA and comorbidity better about benefits of exercise therapy	High health literacy or importance of education (3)
F22	Optimize collaboration with orthopaedic surgeons and other health care providers	Good interdisciplinary collaboration or healthcare provision, or suggestions for improvement (4)
F23	In complex patients insurance companies should reimburse more treatment sessions	Benefits of good health insurance (7)
F24	Useful to plan follow up/refreshment training to repeat/discuss content of course/protocol and application	Ease for HCPs during delivery of LIs (1)
F25	Shortening the protocol would increase user-friendliness	Ease for HCPs during delivery of LIs (1)

Holden (2009) [61]

Number	Description	Subcategory (domain)
B1	Biomedical perspective on knee OA, attributing signs and symptoms to local knee pathology or wear and tear	OA seen as untreatable and local condition (wear-and-tear) (9)
B2	OA seen as chronic degenerative condition that would progressively worsen over time (only cure being surgery)	OA seen as untreatable and local condition (wear-and-tear) (9)
B3	Effectiveness related to severity of joint damage/pain level (less effective when more damage/pain)	Severity of disease and symptoms (3)
B4	Fear of increasing symptoms (as barrier to prescribing exercise)	LIs are unsafe or have negative effects (1)
B5	Causing disease progression, particularly through weight-bearing activities (as barrier to prescribing exercise)	LIs are unsafe or have negative effects (1)
B6	Exacerbating patient’s comorbidities (as barrier to prescribing exercise)	LIs are unsafe or have negative effects (1)
B7	Reluctant to promote exercise in the presence of pain	Severity of disease and symptoms (3)
B8	Negative perceptions of patients’ levels of exercise adherence	Low patient adherence or engagement (3)
B9	Lack of motivation or laziness (as patient-centered barrier to adherence)	Negative attitude toward LIs (3)
B10	Human nature (as patient-centered barrier to adherence)	Potential effects of LIs are difficult to accomplish (1)
B11	Pain (as patient-centered barrier to adherence)	Severity of disease and symptoms (3)
B12	Fear of harm (as patient-centered barrier to adherence)	Low health literacy (3)
B13	Negative treatment expectations (as patient-centered barrier to adherence)	Negative attitude toward LIs (3)
B14	Therapist’s role seen as assessment/exercise prescription/education (relatively short-term responsibilities)	Perception of own role potentially impeding prescription or follow-up of LIs (2)
B15	Patient’s role to follow prescribed exercise program over long term/get on board with treatment	Perception of own role potentially impeding prescription or follow-up of LIs (2)
B16	Limited time to review individual patients reduced opportunities to facilitate behavior change	Lack of time within patient consultations (5)
B17	Large caseloads and pressure of waiting lists reduced the number of treatment sessions provided	Lack of time within patient consultations (5)
B18	Limited opportunity to provide follow-up sessions after discharge	Lack of time within patient consultations (5)
B19	Poor links to community facilities such as local leisure centres	LIs are unavailable or inaccessible (1)

For peer review only

B20	Gaps in knowledge/skills (including how to facilitate behavior change, particularly with less motivated patients)	Lack of knowledge or skills around LIs or promoting behavioral change (2)
B21	Exercises are not/might not be effective for patients if an X-ray shows severe knee osteoarthritis (*)	Severity of disease and symptoms (3)
B22	Increasing overall activity levels does not/might not stop the knee problem getting worse (*)	LIs have little or no effect on OA (1)
B23	Increasing the strength of the muscles around the knee does not/might not stop the knee problem getting worse (*)	LIs have little or no effect on OA (1)
B24	General exercise is not/might not be safe for everybody to do (*)	LIs are unsafe or have negative effects (1)
B25	Local strengthening exercises for the knee are not/might not be safe for everybody to do (*)	LIs are unsafe or have negative effects (1)
B26	Knee problems are not/might not be improved by general exercise (*)	LIs have little or no effect on OA (1)
B27	Physical therapists should (perhaps) not prescribe general exercise for every patient with knee OA (*)	Perception of own role potentially impeding prescription or follow-up of LIs (2)
B28	It is the patient's own responsibility to continue doing their exercise program (*)	Perception of own role potentially impeding prescription or follow-up of LIs (2)
B29	It is not/might not be the physiotherapist's responsibility to make sure that the patient will continue doing their exercise program (*)	Perception of own role potentially impeding prescription or follow-up of LIs (2)
B30	It is not/might not be important that people with knee OA increase their overall activity levels (*)	Negative attitude toward LIs (2)
F1	Effectiveness related to severity of joint damage/pain level (more effective when less damage/pain)	Severity of disease and symptoms (3)
F2	Importance of exercise adherence/link between level of adherence and clinical outcomes (dose-response effect)	Importance of high patient adherence or engagement for effectiveness of LIs (3)
F3	Recognizing potential influence on exercise adherence, sharing responsibility of exercise adherence with patient	Perception of own role potentially stimulating prescription or follow-up of LIs (2)
F4	Exercises are effective for patients if an X-ray shows moderate knee osteoarthritis (*)	Severity of disease and symptoms (3)
F5	Exercises are effective for patients if an X-ray shows mild knee osteoarthritis (*)	Severity of disease and symptoms (3)
F6	Knee problems are improved by local strengthening exercises (*)	LIs have positive effects on affected joint(s) (1)
F7	Physical therapists should prescribe local strengthening exercise for every patient with knee OA (*)	Perception of own role potentially stimulating prescription or follow-up of LIs (2)
F8	How well a patient complies with their exercise program determines how effective it will be (*)	Importance of high patient adherence or engagement for effectiveness of LIs (3)
F9	Physiotherapists should educate chronic patients with knee OA about how to change their lifestyle for the better (*)	Perception of own role potentially stimulating prescription or follow-up of LIs (2)
F10	A standard set of exercises is not/might not be sufficient for every patient with knee OA (*)	Ability and importance of providing personalized treatment within LIs (1)
F11	Exercise for knee OA is most beneficial when it is tailored to meet individual patient needs (*)	Ability and importance of providing personalized treatment within LIs (1)
U1	Exercise does not/might not work just as well for everybody, regardless of the amount of pain they have (*)	Severity of disease and symptoms (3)

For peer review only

Kloek (2020) [62]

Number	Description	Subcategory (domain)
B1	Less satisfied about the applicability of e-Exercise for only one diagnosis	Insufficient ability to provide personalized treatment within LIs (1)
B2	Less satisfied about time needed to get used to e-Exercise during high work pressure/administrative burden	Lack of time due to other demands (or not further specified) (5)
B3	Lack of technology affinity (reason for patients' non-willingness to participate in e-Exercise)	Patient-related challenges regarding feasibility of telehealth (1)
B4	Patients preferred regular face-to-face contact	Patient-related challenges regarding feasibility of telehealth (1)
B5	Perceiving web-based application as an additional burden	Lack of time due to other demands (or not further specified) (5)
B6	Technical skills (lack of)	Other challenges for HCPs regarding feasibility of telehealth (1)
B7	Clarity of instruction manual and course (lack of)	Challenges for HCPs during delivery of LIs (1)
B8	Adaptive capacity to change treatment routines (lack of)	Other challenges for HCPs regarding feasibility of telehealth (1)
B9	Busy work schedules and administrative burden hindered testing/using e-Exercise in their practice	Lack of time due to other demands (or not further specified) (5)
B10	Reduced face-to-face contact interfered with professional autonomy	Challenges for HCPs regarding lack of physical/visual contact (1)
B11	Absence of national e-Health guideline or standard	Other challenges for HCPs regarding feasibility of telehealth (1)
B12	Loss of income due to substitution of face-to-face session	Other challenges for HCPs regarding feasibility of telehealth (1)
B13	E-Exercise does not/might not contain all essential elements for the treatment of hip/knee OA (*)	Non-optimal content or structure of LIs (1)
B14	I do not/might not have enough influence on the content of patients' individual e-Exercise program (*)	Insufficient ability to provide personalized treatment within LIs (1)
B15	The content of e-Exercise is not/might not be aligned with my opinion about treating patients with OA (*)	Non-optimal content or structure of LIs (1)
B16	The intervention provided through e-Exercise is not/might not be appropriate for the average patient with OA (*)	Non-optimal content or structure of LIs (1)
B17	I do not/might not experience that e-Exercise supports patients in doing their exercises at home (*)	Disadvantages of telehealth in terms of effectiveness (1)
B18	Patients who were treated with e-Exercise were (perhaps) not generally positive about the intervention (*)	Non-optimal content or structure of LIs (1)
F1	More flexibility in web-based application (intervention duration, number of sets/repetitions, type of exercises)	Ability and importance of providing personalized treatment within LIs (1)
F2	Completeness of web-based application (exercises/assignments/information)	Positive experiences with or suggestions to improve the content or structure of LIs (1)
F3	Perception that e-Exercise is an appropriate treatment option for subgroup of OA patients	Positive experiences with or suggestions to improve the content or structure of LIs (1)
F4	Added value in terms of exercise adherence (important factor to use web-based	Benefits of telehealth in terms of effectiveness (1)

	application)	
F5	Perceiving web-based application as time-saving	Adequate duration of specific interventions or protocols (5)
F6	More flexibility in intervention (more possibilities to personalize to individual needs)	Ability and importance of providing personalized treatment within LIs (1)
F7	Support from colleagues	Good interdisciplinary collaboration or healthcare provision, or suggestions for improvement (4)
F8	Advantage of reducing number of treatments	Positive attitude or needs of HCPs regarding feasibility of telehealth (1)
F9	Offering an innovative intervention attracted new patients	Positive attitude or needs of HCPs regarding feasibility of telehealth (1)
F10	The instruction course and manual assisted me so that I knew how to work with e-Exercise (*)	Ease for HCPs during delivery of LIs (1)
F11	That it results in less income is not/might not be a major disadvantage of e-Exercise (*)	Positive attitude or needs of HCPs regarding feasibility of telehealth (1)
F12	Our physiotherapy practice has the intention to use e-Health innovations (*)	Positive attitude or needs of HCPs regarding feasibility of telehealth (1)
F13	I do not/might not have insufficient time available to get familiar with e-Exercise and to use the web-application (*)	Adequate duration of specific interventions or protocols (5)
F14	I believe that patient data gathered at the e-Exercise web-application is stored safely (*)	Telehealth is safe for patients or patient/data privacy (1)

For peer review only

Preferred Reporting Items for Systematic reviews and Meta-Analyses extension for Scoping Reviews (PRISMA-ScR) Checklist

SECTION	ITEM	PRISMA-ScR CHECKLIST ITEM	REPORTED ON PAGE #
**TITLE**			
Title	1	Identify the report as a scoping review.	Page 1
**ABSTRACT**			
Structured summary	2	Provide a structured summary that includes (as applicable): background, objectives, eligibility criteria, sources of evidence, charting methods, results, and conclusions that relate to the review questions and objectives.	Page 2-3
**INTRODUCTION**			
Rationale	3	Describe the rationale for the review in the context of what is already known. Explain why the review questions/objectives lend themselves to a scoping review approach.	Page 4-5
Objectives	4	Provide an explicit statement of the questions and objectives being addressed with reference to their key elements (e.g., population or participants, concepts, and context) or other relevant key elements used to conceptualize the review questions and/or objectives.	Page 5
**METHODS**			
Protocol and registration	5	Indicate whether a review protocol exists; state if and where it can be accessed (e.g., a Web address); and if available, provide registration information, including the registration number.	Page 5
Eligibility criteria	6	Specify characteristics of the sources of evidence used as eligibility criteria (e.g., years considered, language, and publication status), and provide a rationale.	Page 6
Information sources*	7	Describe all information sources in the search (e.g., databases with dates of coverage and contact with authors to identify additional sources), as well as the date the most recent search was executed.	Page 6
Search	8	Present the full electronic search strategy for at least 1 database, including any limits used, such that it could be repeated.	Supplemental File 1
Selection of sources of evidence†	9	State the process for selecting sources of evidence (i.e., screening and eligibility) included in the scoping review.	Page 6-7
Data charting process‡	10	Describe the methods of charting data from the included sources of evidence (e.g., calibrated forms or forms that have been tested by the team before their use, and whether data charting was done independently or in duplicate) and any processes for obtaining and confirming data from investigators.	Page 7-9
Data items	11	List and define all variables for which data were sought and any assumptions and simplifications made.	Page 7-9
Critical appraisal of individual sources of evidence§	12	If done, provide a rationale for conducting a critical appraisal of included sources of evidence; describe the methods used and how this information was used in any data synthesis (if appropriate).	Page 7-8
Synthesis of results	13	Describe the methods of handling and summarizing the data that were charted.	Page 9

St. Michael's
Inspired Care.
Inspiring Science.

For peer review only - http://bmjopen.bmj.com/site/about/guidelines.xhtml

SECTION	ITEM	PRISMA-ScR CHECKLIST ITEM	REPORTED ON PAGE #
**RESULTS**			
Selection of sources of evidence	14	Give numbers of sources of evidence screened, assessed for eligibility, and included in the review, with reasons for exclusions at each stage, ideally using a flow diagram.	Figure 1 Page 9
Characteristics of sources of evidence	15	For each source of evidence, present characteristics for which data were charted and provide the citations.	Table 1 Page 10
Critical appraisal within sources of evidence	16	If done, present data on critical appraisal of included sources of evidence (see item 12).	Supplemental File 2 Page 11
Results of individual sources of evidence	17	For each included source of evidence, present the relevant data that were charted that relate to the review questions and objectives.	Supplemental File 4
Synthesis of results	18	Summarize and/or present the charting results as they relate to the review questions and objectives.	Table 2-5 Supplemental File 3 Page 11-13
**DISCUSSION**			
Summary of evidence	19	Summarize the main results (including an overview of concepts, themes, and types of evidence available), link to the review questions and objectives, and consider the relevance to key groups.	Page 13-16
Limitations	20	Discuss the limitations of the scoping review process.	Page 17-18
Conclusions	21	Provide a general interpretation of the results with respect to the review questions and objectives, as well as potential implications and/or next steps.	Page 18-19
**FUNDING**			
Funding	22	Describe sources of funding for the included sources of evidence, as well as sources of funding for the scoping review. Describe the role of the funders of the scoping review.	Page 19-20

JBI = Joanna Briggs Institute; PRISMA-ScR = Preferred Reporting Items for Systematic reviews and Meta-Analyses extension for Scoping Reviews.

* Where *sources of evidence* (see second footnote) are compiled from, such as bibliographic databases, social media platforms, and Web sites.

† A more inclusive/heterogeneous term used to account for the different types of evidence or data sources (e.g., quantitative and/or qualitative research, expert opinion, and policy documents) that may be eligible in a scoping review as opposed to only studies. This is not to be confused with *information sources* (see first footnote).

‡ The frameworks by Arksey and O'Malley (6) and Levac and colleagues (7) and the JBI guidance (4, 5) refer to the process of data extraction in a scoping review as data charting.

§ The process of systematically examining research evidence to assess its validity, results, and relevance before using it to inform a decision. This term is used for items 12 and 19 instead of "risk of bias" (which is more applicable to systematic reviews of interventions) to include and acknowledge the various sources of evidence that may be used in a scoping review (e.g., quantitative and/or qualitative research, expert opinion, and policy document).

From: Tricco AC, Lillie E, Zarin W, O'Brien KK, Colquhoun H, Levac D, et al. PRISMA Extension for Scoping Reviews (PRISMAScR): Checklist and Explanation. Ann Intern Med. 2018;169:467–473. doi: 10.7326/M18-0850.

For peer review only - http://bmjopen.bmj.com/site/about/guidelines.xhtml
